# Predicting the chemical equilibrium point of reacting components in gaseous mixtures through a novel Hierarchical Manta-Ray Foraging Optimization Algorithm

**DOI:** 10.1038/s41598-025-93524-1

**Published:** 2025-04-01

**Authors:** Oguz Emrah Turgut, Hadi Genceli, Mustafa Asker, Mustafa Turhan Çoban, Mohammad Akrami

**Affiliations:** 1 Department of Industrial Engineering, İzmir Bakircay University, Izmir, 35665 Turkey; 2 Faculty of Mechanical Engineering, Yıldız Technical University, 34349 Istanbul, Turkey; 3 Department of Mechanical Engineering, Aydın Adnan Menderes University, 09010 Aydın, Turkey; 4 Department of Mechanical Engineering, Piri Reis University, 34940 Istanbul, Turkey; 5 Department of Engineering, University of Exeter, Exeter, EX4 4QF UK

**Keywords:** Constrained optimization, Manta Ray Foraging Optimization, Opposition-based learning, Quantum search operator, Reactive chemical equilibrium, Structured population, Engineering, Mathematics and computing

## Abstract

This study proposes a Hierarchical Manta-Ray Foraging Optimization (HMRFO) algorithm for calculating the equilibrium points of chemical reactions. To improve the solution diversity in the trial Manta-Ray population and enhance the general optimization effectivity of the algorithm, an ordered hierarchy is integrated into the original algorithm, taking into account the efficient search strategies of Elite-Opposition learning, Dynamic Opposition Learning, and Quantum search operator. Within this proposed concept, the Manta-ray population is divided into three main sub-populations: the Elite Oppositional learning scheme manipulates top elite individuals, Dynamic Oppositional learning search equations update average population members, and quantum-based learning equations process the worst members. The improved MRFO is applied to a hundred 30D and 500D optimization benchmark functions, and results have been compared to those obtained from state-of-art metaheuristic optimizers. Then, the proposed optimizer solved twenty-eight test problems previously employed in CEC-2013 competitions, and corresponding results were benchmarked against well-reputed metaheuristics. This research study also suggests a novel mathematical model for solving chemical equilibrium problems for ideal gas mixtures. Four challenging case studies related to chemical equilibrium problems have been performed by the HMRFO for varying test conditions, and it is observed that HMRFO can effectively cope with the tedious nonlinearities and complexities of the governing thermodynamic models associated with solving chemical equilibrium problems for gaseous reacting mixture components.

## Introduction

Rapid and innovative advancements in microchip technology have led to a quick growth in the computation capability of concurrently processing tasks, which results in the accurate extracting of valuable information from the amounted dataset, paving the way for generating alternative options for solving real-world design problems with varying difficulties and complexities^1^. Optimization problems in real-life applications involve restrictive features such as high dimensionality, non-differentiability, and multidimensionality, which are some of the critical factors jeopardizing the ongoing iterative process to obtain the global answer to the problem at hand, making it extremely difficult to circumvent the problem-specific obstacles faced during iterations^2^. It is found that solving real-world NP-hard optimization problems is a complex task and requires a meticulously devised methodological procedure to arrive at successful conclusions. Numerous optimization approaches have been proposed to overcome the inherent complexities of available problems and improve the general solution quality and accuracy. Traditional methods, including derivative-based optimizers, suffer from the non-differentiable regions of the search space and excessive expenditure of computational resources, collectively hampering the progressive agent-based actions to obtain the optimal solution. Metaheuristic algorithms come to the rescue when conventional optimizers collapse in troublesome situations where no apparent improvement in objective function values is observed within a certain number of iterations due to the incapabilities of the governing analytic-based search equations.

Metaheuristic algorithms are practical and flexible tools for generating near-optimal solutions for hard-to-solve complex optimization problems. Nature-inspired metaheuristic optimizers are one of the promising branches of the general class of metaheuristic approaches; the vast majority comprises intelligently devised search methods simulating commonly encountered natural activities such as food-searching behaviors of population individuals or reproduction characteristics of living organisms. A considerable amount of improvement has been made in the scientific field of nature-inspired algorithms within the recent two decades. Researchers have put various natural phenomena into a well-defined optimization perspective to enhance the algorithm variety in the existing alternatives. Developed algorithms can be categorized into four main branches: evolutionary optimization algorithms, swarm-based optimization algorithms, physics-based algorithms, and human-based algorithms. The paragraph below provides some examples of cutting-edge, state-of-the-art optimizers for these four sub-branches.

The natural evolution process of living individuals mainly inspires evolutionary algorithms. Population members evolve into optimal values through biological operators, which maintain evolutionary actions such as mutation, selection, and reproduction. Genetic Algorithm^3^ is one of the pioneers of this category and conceptualized Darwin’s famous survival of the fittest principle. Differential Evolution^4^ is another representative method inspired by the progressive evolution of the individuals in the population. It is administrated by the three complementary search operators of recombination, mutation, and selection. Evolutionary Strategies^5^, Memetic Algorithms^6^, and Biogeography-based Optimization^7^ are some of the prevalent examples of this category. Swarm intelligence or swarm-based algorithms, in other words, are inspired by the collective behaviors of living organisms during their interactions with the surrounding environment to achieve the global optimum solution of the predefined optimization problem. Particle Swarm Optimization (PSO)^8^ is one of the founders of swarm intelligence methods simulating the food search characteristics of flocking birds or fishes. They randomly initialized trial swarm individuals relocating across the defined search limits throughout the iterations. They obey the representative probing mechanics relying on the governing manipulative equations to conquer the local pitfalls over the domain and reach the optimal solution. Ant Colony Optimization (ACO)^9^ is one of the early successful attempts belonging to the swarm-based algorithms mimicking the extensive exploration of the artificial ants, tracing the released pheromones to direct each other to available food resources. Salp Swarm Optimization^10^, Whale Optimization^11^, Harris Hawks Optimization^12^, Cuckoo Search^13^, and Bat Optimizer^14^ algorithms can be given as exemplary alternatives to the swarm intelligent-based optimizers, having plenty of applications in the existing literature^15–20^.

The governing laws of physics have been successfully translated into a concept of metaheuristic optimizer, which can be observed for many pioneering different algorithms, including the Gravitational Search Algorithm (GSA)^21^, Simulated Annealing (SA)^22^, Big-Bang Big-Crunch (BB-BC) Optimizer^23^, etc. Between these mentioned algorithms, the SA algorithm makes one of the first commanding efforts to convert the physics-based activity into a stochastic metaheuristic algorithm by simulating the metallurgical annealing process. GSA is founded upon the laws of gravity that influence the mass interactions between neighbouring bodies. Within this method, each search agent is affected by the law of motion that forms a systematic approach to organizing the manipulation equations of the algorithm. BB-BC algorithm follows the evolutionary steps of the universe, which is established on two complementary fundamental search mechanisms called Big Bang and Big–Crunch. The Big Bang phase generates the required randomness in producing trial candidate solutions. In contrast, the Big-Crunch phase systematically draws these randomly made solutions into a representative center point to improve diversity within the evolving population.

Human-based metaheuristic optimizers have been developed from various fields of inspiration within two decades. It is unsurprising to witness the surplus of emerging human-inspired optimization methods as humans have excellent thought supremacy and undisputable intelligence superiority over the world’s entire range of living creatures. They simulate these intelligently devised human behaviors into a well-organized algorithm concept to solve the defined optimization problem. Teaching–learning-based optimization (TLBO)^24^, Political Optimizer^25^, and Seeker Optimization^26^ algorithms are three prominent members of human-based optimizers.

Many metaheuristic approaches have been developed to solve global optimization problems^27–30^. Among the multitude of available alternative algorithms, the Manta Ray Foraging Optimization (MRFO) algorithm^31^ is one of the recently developed swarm-based metaheuristic algorithms simulating the intelligent foraging behaviors of manta rays roaming around in the deepest level of the oceans. Despite its relatively new emergence, this algorithm has been applied to various engineering fields and artificially generated optimization benchmark functions to scrutinize its widespread applicability. However, the numerical outcomes of past literature studies reveal that there is still much room to explore how to improve the general search’s effectiveness by employing intelligently devised manipulation schemes in the original algorithm. This study aims to overcome this research gap by introducing a structured hierarchy into MRFO to increase solution diversity within the manta ray population as much as possible. The iteratively adjusted trial manta ray population is subdivided into three main subgroups composed of elite, average, and worst individuals. The elite members of the subset with the best fitness values update their current position through the Elite opposition-based learning search equation^32^, which is prolific in exploiting the promising regions explored in the previous iterations. A subpopulation comprised of average individuals improves their respective fitness qualities by using the responsible search equation of Dynamic-Opposition based Learning^33^, thanks to its asymmetric and dynamically adjusting search domain, which enables unvisited regions throughout the iterative process, augmenting the exploration capability of the algorithm. Finally, the locations of the worst population members are re-positioned within the defined search space through a Quantum-based Learning mechanism^34^, which can diversify the solution domain as much as possible to reach the global optimum solution of the optimization problem.

The current literature has witnessed the development of hierarchical metaheuristic algorithms that have been consistently applied to many optimization cases from diverse fields. Yen and Lu^35^ developed a hierarchical genetic algorithm for adjusting the model parameters of multi-layer feedforward neural networks. Khishe et al.^36^ proposed using a Hierarchical Population-Based Differential Evolution algorithm for parameter identification of PEM Fuel Cells. Bao et al.^37^ hybridized a Student Psychology Optimization algorithm with Differential Evolution and hierarchical learning mechanism for solving data clustering problems with varying difficulties. Wei et al.^38^ proposed an improved version of the Sparrow Search Algorithm enhanced with an adaptive multi-strategy hierarchical mechanism for high-dimensional complex global optimization problems and constrained engineering design problems. Zhong et al.^39^ developed a Hierarchical RIME algorithm using multiple search operators to tune the model parameters of an extreme learning machine algorithm. Here in this study, a Hierarchical Manta-Ray Foraging algorithm is developed for solving chemical equilibrium problems, another contribution to the current literature regarding multi-population metaheuristic algorithms. After exhaustive investigations of the existing literature approaches, it is comprehended that no available accomplished study is devoted to finding the equilibrium point of gas mixtures in the reactive chamber. Therefore, a hierarchically structured MRFO algorithm is utilized to obtain the chemical equilibrium point of gas phase reacting mixtures, whose governing mathematical model is highly complex and challenging, rendering this problem an efficient test bed to assess the predictive performance of the proposed algorithm.

The authors’ past experiences suggest that the MRFO algorithm has some algorithmic disadvantages, such as its being trapped in local solutions when diversity within the population is lost. Furthermore, premature convergence to the local answers can also be observed when similarities between population members are high, and there is no chance to improve the general diversity within the population when hyperdimensional problems are solved. Motivated to conquer these inherent disadvantages of the MRFO algorithm, a hierarchically ordered population is introduced to improve the algorithm’s global performance. The main contributions of this study can be summarized in four aspects.

- Constructing a multi-population metaheuristic method in which each sub-group employs different mutation equations to enhance the overall search efficiency of the ruling MRFO algorithm
- Evaluating the optimization performance of the hierarchical learning strategy-based MRFO (HMRFO) algorithm over 30D and 500D optimization benchmark problems along with artificially produced CEC—2013 test cases
- Different oppositional learning-based MRFO algorithms have been developed, and their resulting performances over 1000D standard test problems have been compared to those obtained by the HMRFO algorithm
- Proposing a novel mathematical model for solving reactive chemical equilibrium problems for vapor mixtures at the ideal gas state
- Solving the reactive chemical phase equilibrium problems through the proposed HMRFO, whose functional characteristic is also highly challenging and involves extreme non-linearities and whose accurate and global solution has yet to be successfully obtained by the traditional optimization solvers.

## The proposed Hierarchical Manta-Ray Foraging Optimization (HMRFO) Algorithm Manta-Ray Foraging Optimization Algorithm

MRFO algorithm mathematically models the intelligent foraging behaviors of manta rays into a swarm-based metaheuristic optimizer. Manta ray individuals feed on plankton that can be abundantly found in deep regions of oceans containing plants and sea animals loosely swimming due to ocean currents. In this algorithm concept, swarming manta rays aim to maximize their food share by employing three distinctive food search mechanisms: chain foraging, cyclone foraging, and somersault foraging. Manta rays form long chains while practicing their intrinsic foraging skills that can reach 50 individuals, forming a relatively crowded group. Chain foraging relates to this hunting behavior in which 50 or more manta rays line up in the form of a chain so that the following one behind them can scoop up the missed plankton by the previous manta ray in the chain. This favorable cooperation between the manta individuals forming the chain group minimizes the possibility of missing the available plankton resources, increasing the chance to funnel most food rewards. The second intelligent food search mechanism is cyclone foraging. Manta rays gather together to form a spiral-shaped chain when they detect a fertile food region with a high concentration of plankton. A spiral vertex is collectively formed in the eye of a cyclone by linking head-to-tail sequence of manta rays, which moves the filtered water up towards the surface, entailing a sudden and forceful pull of the planktons into the open mouths of the foraging manta rays. The third and last foraging mechanism is somersault foraging, which differs from other food search methods employed by manta rays. When food search is detected, foraging manta rays perform a series of backward somersaults to attract the available plankton and draw them into a swarm of manta rays. The subsequent sections will explain these foraging mechanisms’ respective mathematical modeling formulations.

### Chain foraging

In the MRFO algorithm, mantra rays move toward the areas where food plankton are resourceful. The higher the concentration of plankton, the better their current location is. MRFO algorithm supposes that the best solution obtained so far is the plankton location where highly concentrated food resources reside. Manta rays aim to approach these fertile food regions by lining up a heat-to-tail foraging chain. The following mathematical formulation simulates this intrinsic foraging mechanism.

1 $$x_{i}^{t + 1} = \left\{ \begin{gathered} x_{i}^{t} + rd.\left( {x_{best}^{t} - x_{i}^{t} } \right) + \alpha .\left( {x_{best}^{t} - x_{i}^{t} } \right)\quad i = 1 \hfill \\ x_{i}^{t} + rd.\left( {x_{i - 1}^{t} - x_{i}^{t} } \right) + \alpha .\left( {x_{best}^{t} - x_{i}^{t} } \right)\quad i = 2,3, \ldots ,N \hfill \\ \end{gathered} \right.$$

2 $$\alpha = 2.rd.\sqrt {\log \left( {rd} \right)}$$

where $x_{i}^{t}$ is the current location of the plankton at the current iteration t; rd is a uniformly distributed random number in the range [0,1]; α is the weighing coefficient calculated by Eq. (2); $x_{best}^{t}$ is the position of planktons with the highest concentration.

### Cyclone foraging

Flocking mantra rays form a foraging chain when they recognize the fruitful locations of plankton with higher concentrations in deep water and move towards this region by creating a spiral chain. Each manta ray constituting the chain swims over in front of it during cyclone foraging strategy while moving towards the food resources following a spiral path. The following equation can express mathematical modeling representing the cyclone foraging mechanism.

3 $$x_{i}^{t + 1} = \left\{ \begin{gathered} x_{best}^{t} + rd.\left( {x_{best}^{t} - x_{i}^{t} } \right) + \beta .\left( {x_{best}^{t} - x_{i}^{t} } \right)\quad i = 1 \hfill \\ x_{best}^{t} + rd.\left( {x_{i - 1}^{t} - x_{i}^{t} } \right) + \beta .\left( {x_{best}^{t} - x_{i}^{t} } \right)\quad i = 2,3, \ldots ,N \hfill \\ \end{gathered} \right.$$

4 $$\beta = 2exp\left( {rd.\left( {\frac{T - t + 1}{T}} \right)} \right).\sin \left( {2.\pi .rd} \right)$$

where *β* is the weight coefficient; *T* is the maximum number of iterations defined for the termination criterion; *rd* is a random number in the range [0,1]. The cyclone foraging mechanism steers the direction of the stochastic search process towards the plankton-abundant regions, which empowers the algorithm’s exploitation capability. The cyclone foraging mechanism also emphasizes the exploration phase by positioning the swimming mantra rays in random search space locations. Each individual in the swarm probes around the search domain to arrive at a random location, facilitating the diversification of the candidate solutions within the manta ray population as much as possible. The formulation below aims to generate sufficient diversity among the population by employing random locations within the search domain, enabling a well-organized global search mechanism.

5 $$x_{rand} = LB + rd.\left( {UB - LB} \right)$$

6 $$x_{i}^{t + 1} = \left\{ \begin{gathered} x_{rand}^{t} + rd.\left( {x_{rand}^{t} - x_{i}^{t} } \right) + \beta .\left( {x_{rand}^{t} - x_{i}^{t} } \right)\quad i = 1 \hfill \\ x_{rand}^{t} + rd.\left( {x_{i - 1}^{t} - x_{i}^{t} } \right) + \beta .\left( {x_{rand}^{t} - x_{i}^{t} } \right)\quad \;i = 2,3, \ldots ,N \hfill \\ \end{gathered} \right.$$

here *x*_*rand*_ is the random position defined in the search space computed by Eq. (5), and *LB* and *UB* are the lower and upper bounds of the solution domain.

### Somersault foraging

This foraging phase of the algorithm considers previously explored fruitful food locations as a pivot point to reach more fertile areas. Manta rays update their current sites around the best positions by practicing somersault flip movements through the following equation.

7 $$x_{i}^{t + 1} = x_{i}^{t} + S.rd.\left( {x_{best}^{t} - x_{i}^{t} } \right)\quad i = 1, \ldots ,N$$

*S* is the model parameter that decides the somersault range of the swimming manta rays and is set to *S* = 2; *rd* is a random number between 0 and 1. Table 1 provides the pseudo-code of the MRFO algorithm.

**Table 1:**
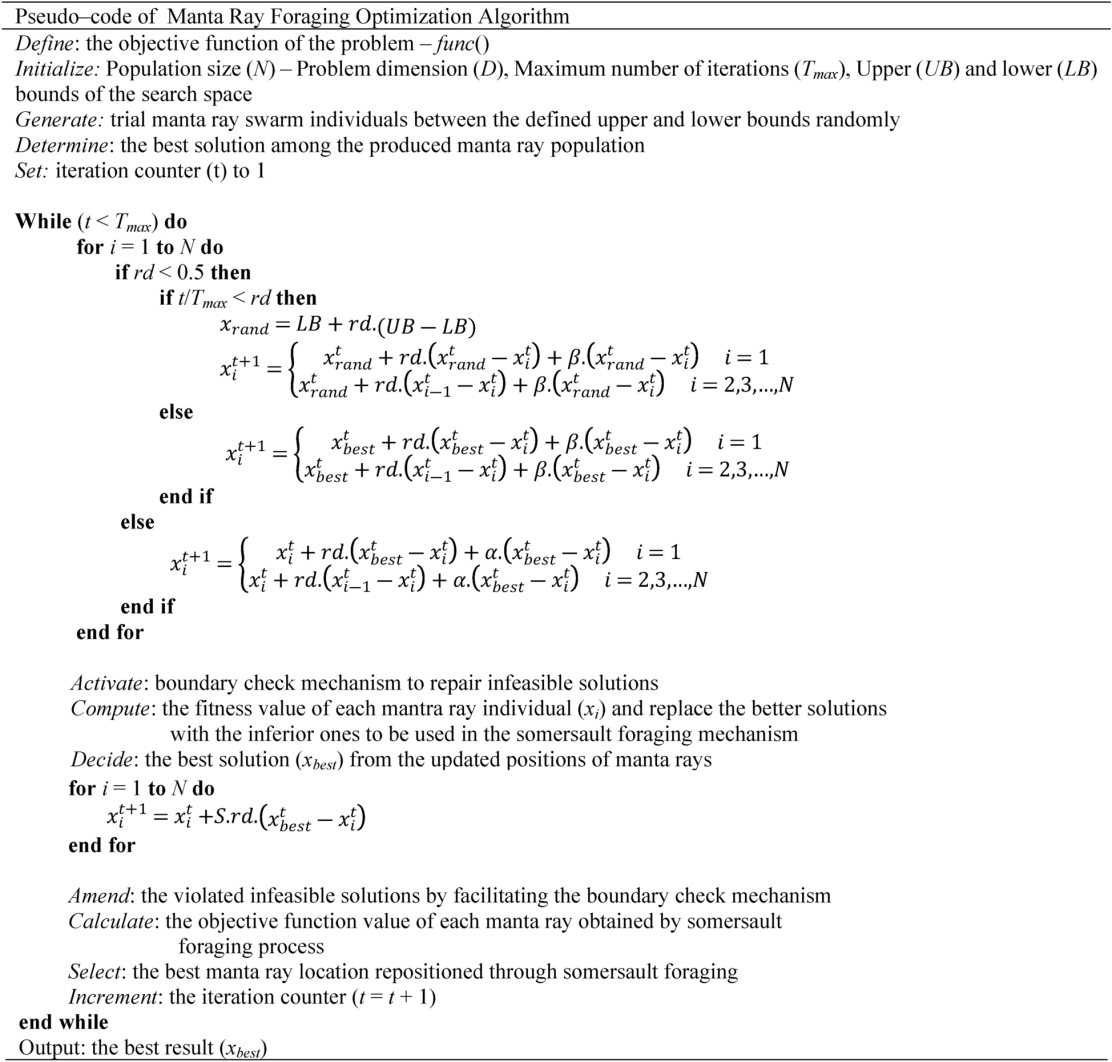
Pseudo-code of Manta-Ray Foraging Optimization Algorithm.

## Hierarchical Manta-Ray Foraging Optimization Algorithm

Previous extensive experiences with the general optimization performance of the Manta Ray Foraging Optimization Algorithm reveal its sufficient exploration and exploitation ability based on prediction results obtained from different kinds of constrained and unconstrained multidimensional problems. However, it suffers from premature convergence to local solutions and poor global exploration when facing highly nonlinear-constrained optimization problems. Manta rays in the population employ three complementary foraging mechanisms, taking advantage of various manipulation schemes. When encountering challenging optimization problems, most manta ray individuals are prone to accumulate around the best solution obtained so far, jeopardizing the iterative process of global exploration. With the increasing number of iterations, search agents tend to gather around local solutions residing in the infertile areas, leading to inferior prediction results and hampering the probing efficiency of the general search process, particularly for high dimensional optimization problems. Therefore, this study introduces a novel hierarchical learning mechanism into the original Manta Ray Optimization algorithm to overcome the issue of local solution entrapment and enhance the algorithm’s overall search quality algorithm to achieve higher performance levels.

As it is known, standard metaheuristic methods rely on the interactive accumulation of domain information among population members to evolve into new offspring individuals iteratively. However, this solution update mechanism often makes the algorithm susceptible to stagnation during solution convergence. As the iterations proceed, the ruling search mechanism between the alternative hunting skills of the manta ray is dominated by the chain foraging scheme, in which the relocating of manta ray individuals is based on the vectorial difference between the current best manta ray position and the actual position of other members, emphasizing on the local search phase of the algorithm. Although it can be evaluated that the transition between global and regional search mechanisms is well organized within the MRFO algorithm, it may lead to deceptive prediction results as spatial differences between the current best and actual solution become so small with increasing function evaluations and resulting in poor exploitation for making better estimations. Numerical experiments based on multidimensional and hyperdimensional test problems solved by the MRFO algorithm also suggest there is considerable room for improving the local search capability of the original algorithm by introducing diversified solutions extracted from various manipulation equations. Considering these algorithmic disadvantages of MRFO, a hierarchical population is implemented into basic MRFO to alleviate their detrimental influence and enhance its overall probing efficiency.

**Fig. 1:**
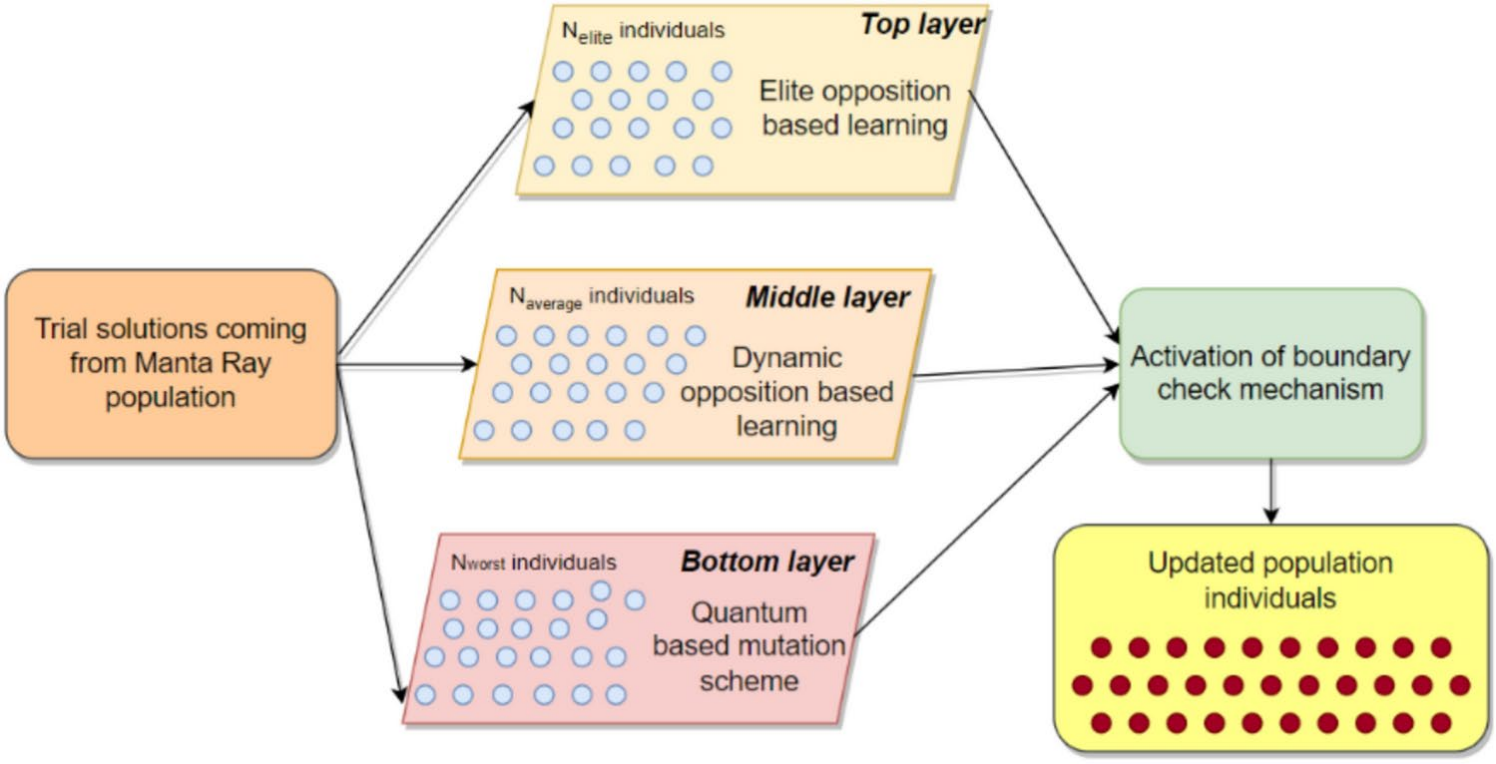
Three-layered hierarchically structured Manta Ray population.

A hierarchical structure is constructed and implemented into the base MRFO algorithm in such a well-adapted fashion that contributing population individuals are ordered based on their respective fitness rates, and each constituent individual occupying different layers has distinctive functional characteristics. Interaction between the linking layers can provide a practical guideline throughout the iterations for evolving individuals within the population to improve their fitness accuracies efficiently. Several hierarchical layers are defined from top to bottom, forming a tree-like structure based on the search equations employed for each layer and the competitive individuals’ influential contributions. Connecting layers comprised of two or more structural elements carrying characteristic sub-population information form a hierarchical structure to establish a beneficial interactive relationship between them. It is worth mentioning that enhancing the diversity of the whole population can be more easily maintained by the exchange of hierarchical domain information between the layered subgroups, each of which has been manipulated by their assigned manipulative scheme. This kind of interaction between the sub-population members not only alleviates the local minima entrapment arising from insufficient exploration but also conquers the annoying premature convergence problem, adversely influencing pinpointing the promising regions explored during the iterative process. This study proposes a three-layered hierarchical structure consisting of top, medium, and bottom layers to address this issue. Figure 1 schematically visualizes the three-layered hierarchically structured population of manta rays, employing different search mechanisms in each layer.

The upper class of the hierarchy occupies the elite individuals updated by the ruling search mechanism of elite oppositional-based learning^40^, which has been widely employed in literature for concurrently enhancing the global and local search effectiveness of the base metaheuristic algorithm^41,42^. Most metaheuristic algorithms must improve their probing of the entire solution domain. The elite opposition-based learning mechanism assists the governing base algorithm produce accurate estimations. This position update strategy is relatively new in computational intelligence algorithms, whose central ideology relies on computing the current and opposition solutions simultaneously, evaluating their respective fitness value, and selecting the best individuals to be used for the upcoming generations. A candidate solution with the best objective function value is considered the elite individual, and the ruling learning mechanism pivots around this elite solution. The definition given below can briefly explain the Elite oppositional-based learning search strategy. Suppose that the so far obtained elite individual of the population is the solution vector expressed by *i*th individual. Then the elite oppositional-based solution is defined as ${\overrightarrow{X}}_{eopp}=({x}_{eopp,1},{x}_{eopp,2},\dots ,{x}_{eopp,D})$ and can be calculated by the following formulation^40^

8 $$x_{eopp,i,j} = rand_{i,j} \left( {0,1} \right).\left( {dlow_{j} + dup_{j} } \right) - x_{elite,j }\quad i = 1,2, \ldots ,N, j = 1,2, \ldots ,D$$

where *N* is the size of the population; *D* is the problem dimension; *rand*(0,1) is a random number drawn from Gaussian distribution in the range [0,1]; *dlow*_j_ and *dup*_j_ are dynamic lower and upper bounds of *j*th decision variable and obtained by the following

9 $$dlow_{j} = \min \left( {x_{i,j} } \right),dup_{j} = {\text{max}}\left( {x_{i,j} } \right)$$

The dynamic search range is good at carrying the accumulated search experiences into the upcoming generations. However, generated solutions through this model may pose a risk of jumping out of the dynamic bound. If this occurs, the below-given expression amends and repairs the violated infeasible solutions.

10 $$x_{eopp,i,j} = rand\left( {dlow_{j} ,dup_{j} } \right)\quad if\,x_{eopp,j} \left\langle {dlow_{j}\;or\;x_{eopp,j} } \right\rangle dup_{j}$$

**Fig. 2:**
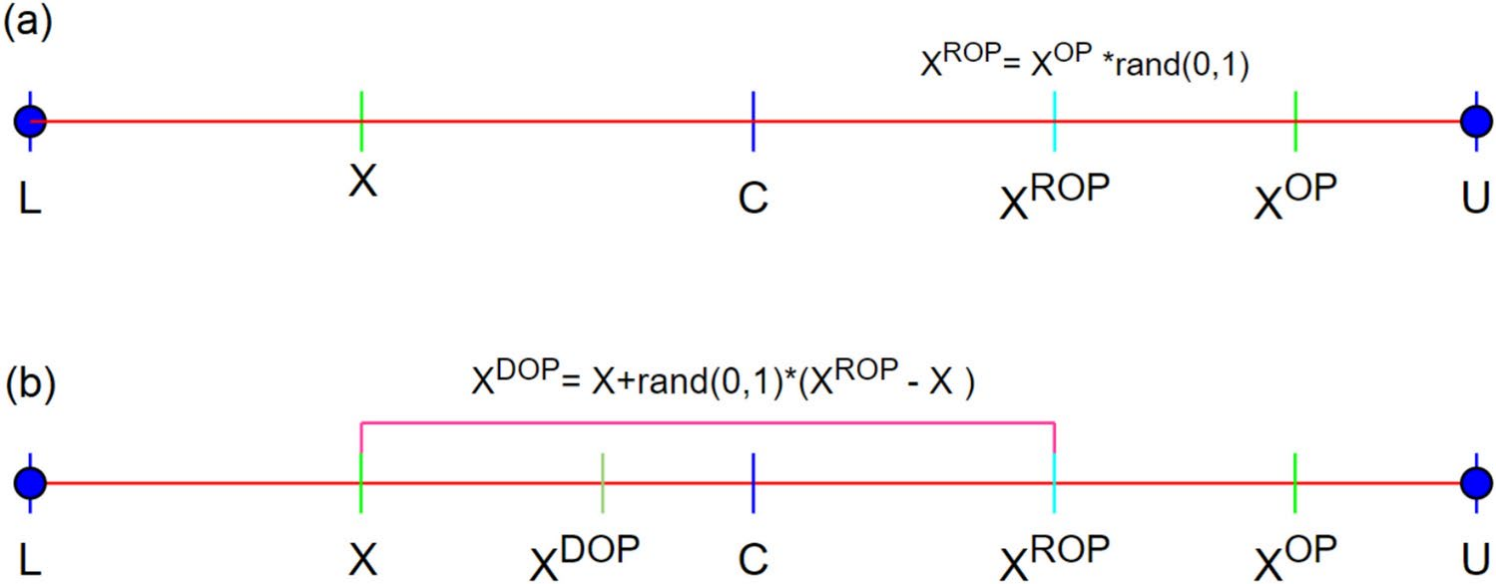
Asymmetric search space of Dynamic Opposition Learning. (**a**) Generation of Random Opposite Number (X^ROP^) (**b**) Generation of Dynamic Opposite Number (X^DOP^).

*N*_*elite*_ is the total number of elite individuals in the top hierarchical layer manipulated by the above-explained elite oppositional learning mechanism in the constructed hierarchy. In the medium level of the hierarchy, the dynamical oppositional learning search mechanism is the dominant mutation strategy used to alter the residing individuals’ general solution quality. Despite its relatively new emergence, this oppositional-based learning variant draws satisfactory attention from the research community. The asymmetric search space of the Dynamic Oppositional Learning mechanism enables the ruling algorithm to arrive at the unexplored regions previously, enriching the diversity of the population and enhancing the overall exploitation ability. Dynamic Oppositional Learning was proposed by Xu et al.^33^ and implemented within the Teaching–Learning-Based Optimization algorithm. Its primary inspiration was to expand the search space as much as possible to avoid local solutions and reach the global answer to the problem more efficiently. As evident from Quasi oppositional-based learning^43^ and Quasi reflection-based learning^44^ solution update mechanisms, oppositional learning-based solution space expansion is conducive to getting closer to the areas where the optimum points are possibly located. Within the concept of Dynamic Oppositional base learning (*DOP*), a random opposite number (*X*^*ROP*^) is generated between the defined upper and lower bounds, represented by the multiplication of the produced opposite number (*X*^*OP*^) and a uniformly distributed random number *rand*(0,1) to alleviate detrimental effects of the premature convergence problem. Figure 2 shows that a symmetric search space is changed into an asymmetric space using *X*^*RO*^ instead of *X*^*OP*^. Then, the Dynamic Opposition point is randomly generated between the current point (*X*) and the randomized oppositional point (*X*^*ROP*^), as described in Fig. 2.

A brief set of formulations describing the dynamic opposition learning defined for a *D*-dimensional space can be given as follows:

11 $$\begin{gathered} X_{j}^{OP} = L_{j} + U_{j} - X_{j} \hfill \\ X_{j}^{ROP} = X_{j}^{OP} .rand\left( {0,1} \right)\quad j = { 1},{2}, \ldots ,D \hfill \\ X_{j}^{DOP} = X_{j} + \alpha .rand\left( {0,1} \right).\left( {X_{j}^{ROP} - X_{j} } \right) \hfill \\ \end{gathered}$$

here *L* and *U* are the lower and upper search bounds of the *j*th design variable; *D* is the problem dimension; *rand*(0,1) is a random value defined between 0 and 1; *X* is a real-valued number representing the current passion of the search agents; *X*^*OP*^ is the opposite solution; *X*^*ROP*^ is the randomly repositioned opposite solution; and *X*^*DOP*^ is the spatial location of a dynamically adjusted opposite point in *D*-dimensional solution domain, and α is a model parameter defined for scaling the positional distance between current (*X*) and dynamic opposition point (*X*^*DOP*^). In the original paper where dynamical oppositional-based learning was first coined, the upper and lower bounds of the search space are also updated at a specific iteration interval. However, in this study, we proposed using a static boundary range rather than dynamically changing upper and lower limits, relying on the prediction success of the dynamic opposition-based learning mechanism by employing stationary search limits. In addition, the numerical value of model parameter α is taken as 1.0. The total number of *N*_*aver*_ population members is updated by a dynamical oppositional-based earning mechanism to alter their current positions in the *D*-dimensional search space. The individuals accumulated in the bottom layer have the worst fitness values. Therefore, their respective objective function values should be ameliorated through a well-devised mutation scheme. Quantum-behaved solution manipulation procedure is applied to the bottom layer residents of the population. Past literature studies regarding quantum-behaved metaheuristic algorithms assertively state that integrating this dexterous scheme into the base algorithm significantly improves the algorithm’s performance regarding probing capability and local minima avoidance^45,46^. To improve the overall fitness quality of subpopulation members taking place in the bottom layer, quantum behaved theory is applied to the candidate solution of this layer, whose explicit formulation can be given as:

12 $$X_{i,j}^{t + 1} \leftarrow \left\{ \begin{gathered} P_{i,j}^{t} + \beta^{t} \cdot \left| {Mean_{j} - X_{i,j}^{t} } \right| \cdot \ln \left( {\frac{1}{{rand\left( {0,1} \right)}}} \right)\quad rand\left( {0,1} \right) < 0.5 \hfill \\ P_{i,j}^{t} - \beta^{t} \cdot \left| {Mean_{j} - X_{i,j}^{t} } \right| \cdot \ln \left( {\frac{1}{{rand\left( {0,1} \right)}}} \right)\quad otherwise \hfill \\ \end{gathered} \right.$$

where *t* is the iteration counter; *rand*(0,1) is a random number defined between 0 and 1; model parameter *β* is a real-valued number decreased from 1.0 to 0.5 with an increasing number of iterations and calculated by the following.

13 $$\beta = 1 - 0.5\frac{t}{T}$$

where *T* represents the defined maximum number of iterations to terminate the iterative process; *P* is the average position between the current solution (*X*) and the best solution obtained so far (*X*_*best*_) and calculated by the following.

14 $$P_{{i,j}}^{t} = \frac{{r_{1} .X_{{i,j}}^{t} + r_{2} .X_{{best,j}}^{t} }}{{r_{1} + r_{2} }}x_{{elite,j}} \quad i = 1,2, \ldots ,N,j = 1,2, \ldots ,D$$

where *r*_1_ and *r*_2_ are uniform random numbers between 0 and 1, which are also different from each other; average values of the population members for each different problem dimension are denoted by the parameter “Mean” and computed by

15 $$Mean^{t} = \left( {m_{1}^{t} ,m_{2}^{t} , \ldots ,m_{D}^{t} } \right) = \left( {\frac{1}{N}\mathop \sum \limits_{i = 1}^{N} x_{i,1,}^{t} \frac{1}{N}\mathop \sum \limits_{i = 1}^{N} x_{i,2,}^{t} \ldots ,\frac{1}{N}\mathop \sum \limits_{i = 1}^{N} x_{i,D}^{t} } \right)$$

where *N* is the population size, the quantum-behaved mutation scheme is a convenient and practical tool for generating diversity within the population. This mutation scheme takes advantage of the exponential distribution obtained by the global convergence between the population individuals residing in the bottom layer while exploring new locations. In addition, employing the mean position of the individuals provides a favorable pathway to conquer the convergence stagnation problem. It enables open doors to enhance the algorithm’s general search performance. *N*_*worst*_ individuals residing in the bottom layer update their current locations by a quantum-behaved mutation scheme. One last point that should be precisely clarified is deciding the total number of population members within each hierarchical layer. Exhaustive numerical experiments on multidimensional constrained and unconstrained test problems reveal that using the below-given set of formulations associated with assigning the number of subpopulation members to different hierarchical layers provides the most accurate predictions.

16 $$\begin{gathered} N_{elite} = fix\left( {N.0.5.rand\left( {0,1} \right)} \right) \hfill \\ N_{over} = fix\left( {N - N_{elite} ).rand\left( {0,1} \right)} \right) \hfill \\ N_{worst} = N - N_{elite} - N_{over} \hfill \\ \end{gathered}$$

**Fig. 3:**
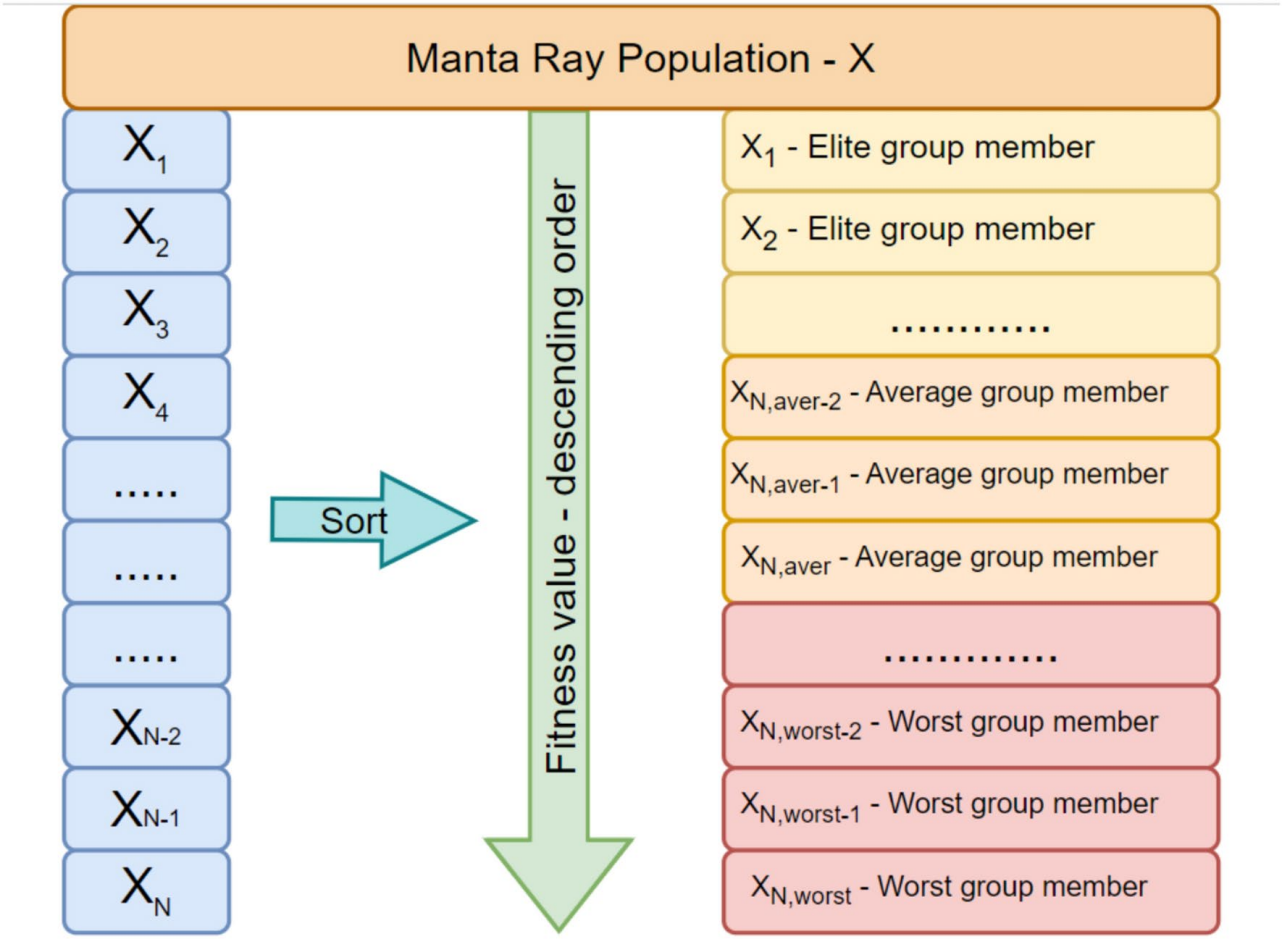
Organization of the manta ray individuals within the proposed hierarchy.

**Fig. 4:**
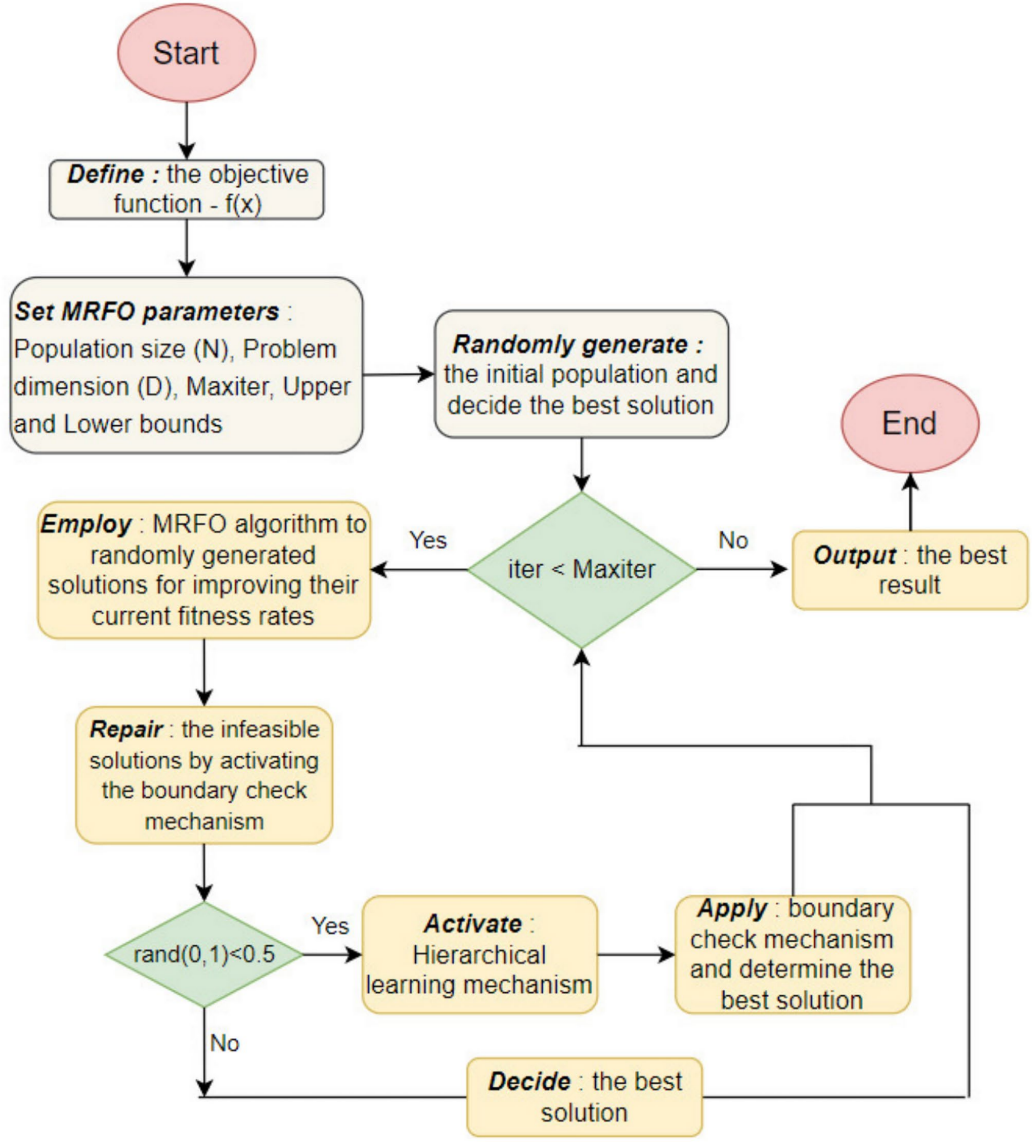
Visual algorithmic description of the proposed HMRFO.

In the above formulations, *fix*() function is used for converting the continuous value of the obtained population member into the nearest integer. As seen from the set of equations, the number of individuals in each layer varies and does not stay constant as iterations proceed, thanks to the contribution of a random number within [0,1] into the decisive formulations. Table 2 describes the proposed hierarchical learning scheme in a pseudo-code form. Figure 3 shows the organization of the manta ray individuals within the hierarchically ordered manta ray population and how the respective fitness values of the population members influence their current position within the structured hierarchy. Figure 4 visualizes the algorithmic steps of the proposed HMRFO algorithm. The following section discusses the predictive results of multimodal and unimodal test functions and their respective convergence analysis.

**Table 2:**
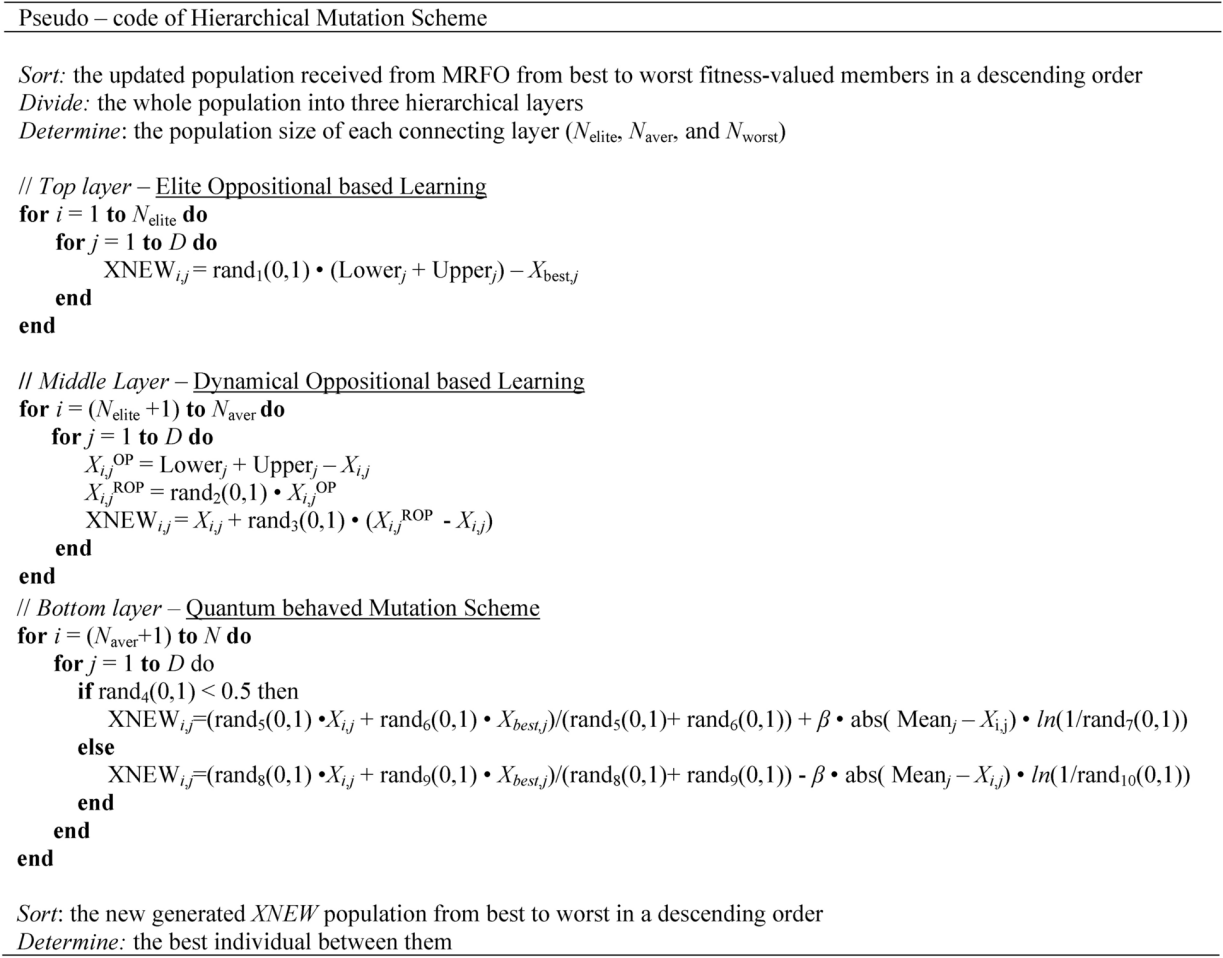
Representative pseudo-code of the proposed learning scheme.

### Numerical experiments made on the proposed HMRFO

The proposed algorithm has been employed on well-known artificially generated unimodal and multimodal multidimensional test functions to evaluate its general optimization performance in this section. These benchmark problems have been considered for their challenging and complex functional characteristics. They are efficient test beds for assessing the search efficiency of any proposed metaheuristic algorithm widely used in most past literature applications. Further evaluations extend to comparative performance analysis between the Hierarchical Manta Ray Foraging Optimizer and (HMRFO) and well-reputed metaheuristic of the original Manta Ray foraging Optimization Algorithm (MRFO)^31^, Multiverse Optimization Algorithm (MVO)^47^, Particle Swarm Optimization (PSO)^8^, Sine–Cosine Optimization (SINECOS)^48^, Jaya Optimization algorithm (JAYA)^49^, Equilibrium based Optimizer (EQUIL)^50^, and Moth-Flame Optimization (MOTH)^51^. All these algorithms are developed in MATLAB and run on a desktop computer with 8.0 GB RAM running an Intel Core processor @ 2.50 MHz. Exhaustive and tedious experiments made over benchmark functions show that setting the fixed population size to *N* = 20 for each compared metaheuristic algorithm yields the most reliable predictions. Furthermore, 100 iterations have been considered for each algorithm to maintain a fair comparative study. Table 3 reports the numerical values of the model parameters of each metaheuristic optimizer considered in the benchmarking event. All algorithms are run 50 times, considering 2000 function evaluations for each solved benchmark problem.

**Table 3:** Numerical values of algorithm parameters.

Algorithm	Model parameters
HMRFO	Somersault parameter (*S* = 2)
MRFO	Somersault parameter (*S* = 2)
MVO	Upper limit of Wormhole Existence Probability (*WEP*_*max*_ = 1.2)
	Lower limit of Wormhole Existence Probability (*WEP*_*min*_ = 0.2)
PSO	Social parameter (*c*_1_ = 2.0)
	Cognitive parameter (*c*_2_ = 2.0)
	Maximum value of inertia coefficient (*w*_*max*_ = 0.6)
	Minimum value of inertia coefficient (*w*_*min*_ = 0.1)
SINECOS	No tunable parameter involved
JAYA	No tunable parameter involved
EQUIL	No tunable parameter involved
MOTH	No tunable parameter involved

The optimization efficiency of the Hierarchical Mantra Ray Foraging Optimization algorithm has been investigated over a hundred test functions comprised of 30D and 500D unimodal and multimodal benchmark problems. As it is known, unimodal functions have only one global optimum point that makes them beneficial for assessing the exploitative ability of the running algorithm. Having more than one local optimum point scattered around the search space, multi-modal optimization problems are widely used tools for evaluating the exploration performance of the algorithm. The optimization efficiency of each compared metaheuristic optimizer is assessed using the outcomes of their statistical analysis obtained after 50 independent algorithm runs. Statistical performance parameters such as best, mean, worst, and standard deviation results are critical for evaluating their prediction success. Between these statistical parameters, average values and standard deviation rates are effective indicators in assessing the reliability of the algorithms. Furthermore, the estimation results pool’s minimum and maximum fitness values represent the best possible computational cost for a given optimization problem. Comparative statistical tests ensure that predictive results are not produced by chance. To validate the significance rate of the performed predictions, a non-parametric Wilcoxon statistical test analysis is carried out, and the obtained p-values are reported as having a degree of significance for a given set of estimate solutions.

### Experiment case—1: performance assessment of HMRFO algorithm for 30D global optimization problems

**Fig. 5:**
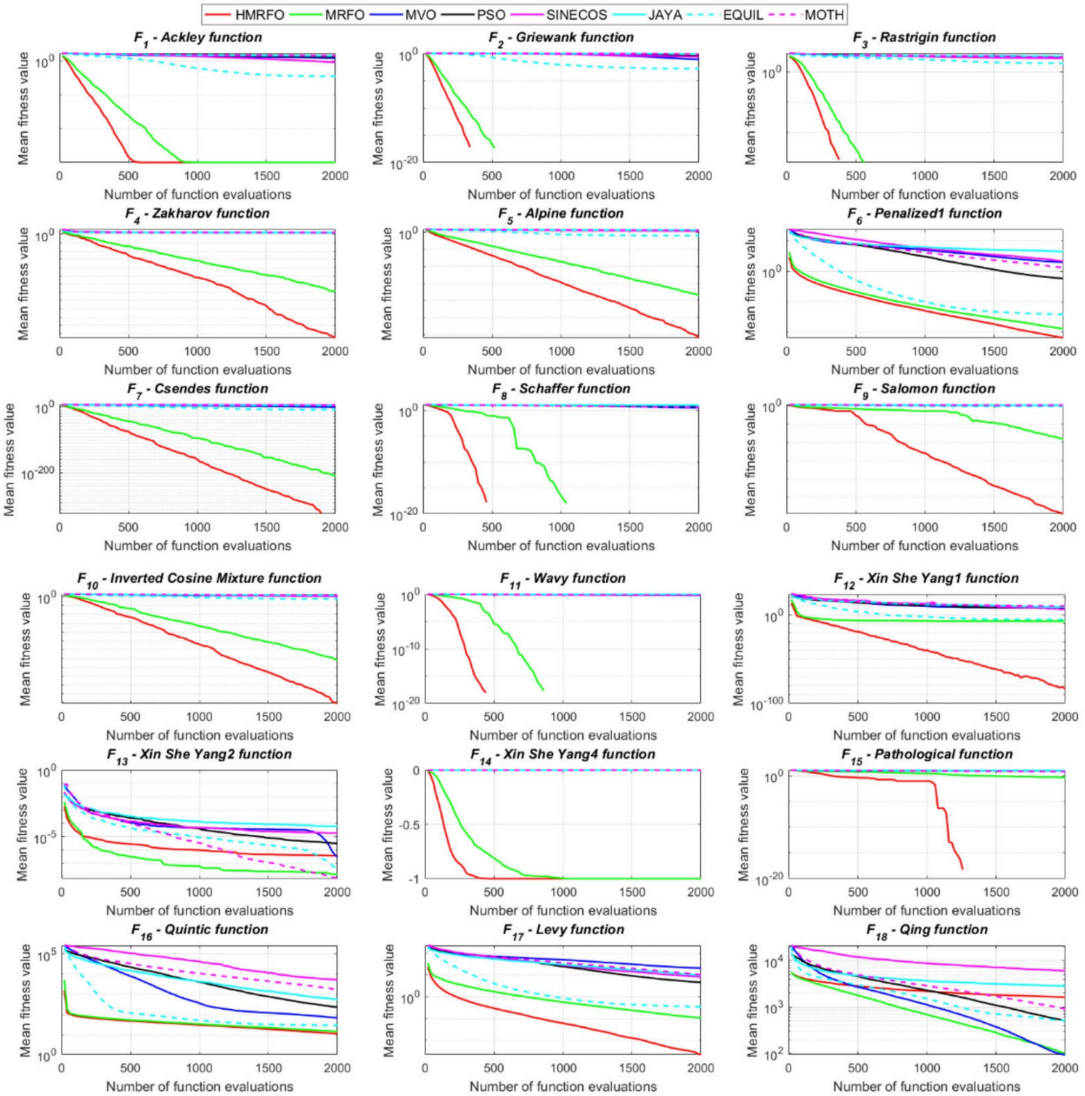
Convergence graphs for the multimodal problems from F_1_–F_18_.

**Fig. 6:**
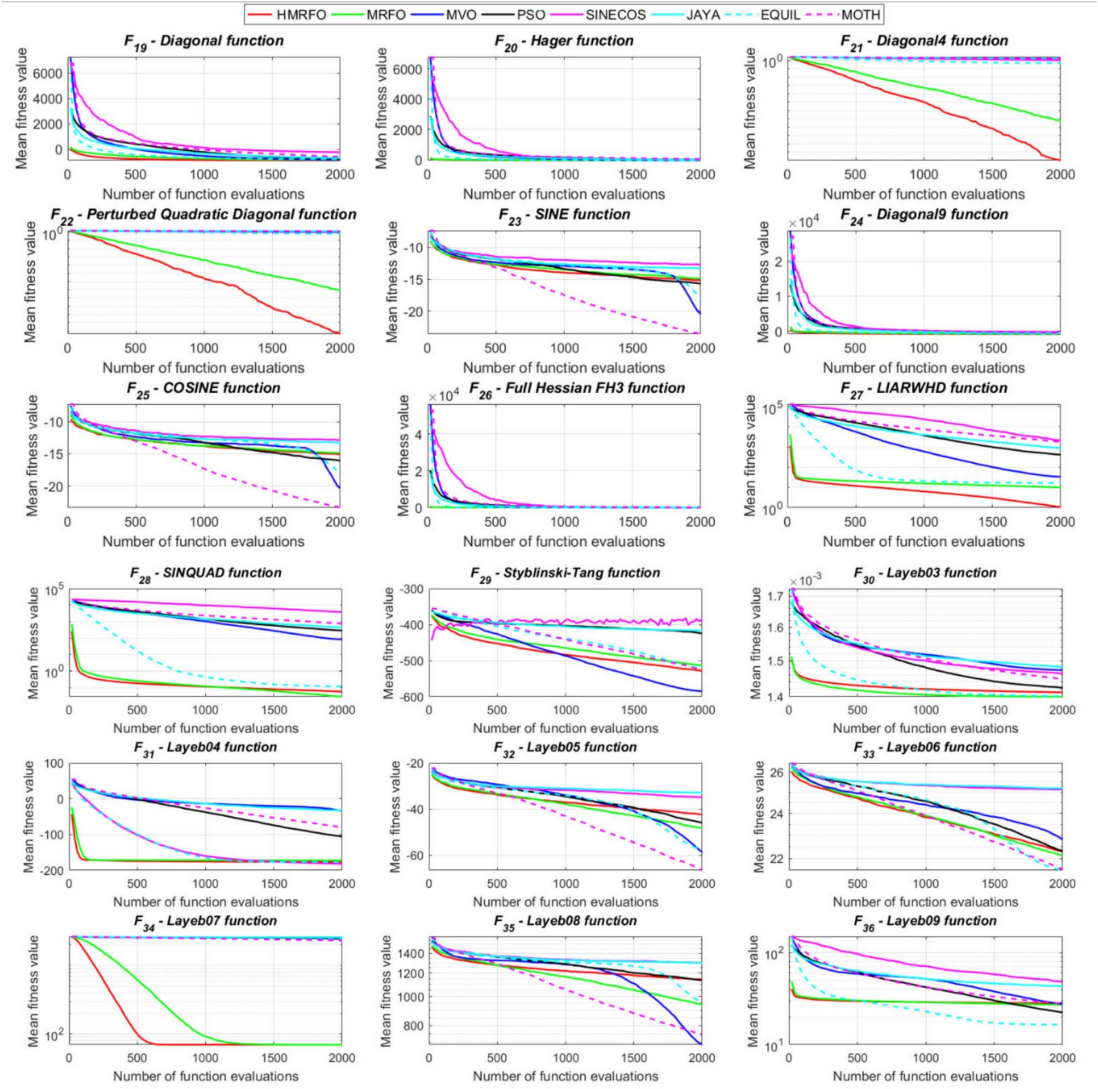
Evolution plots for the multimodal problems from F_19_–F_36_.

**Fig. 7:**
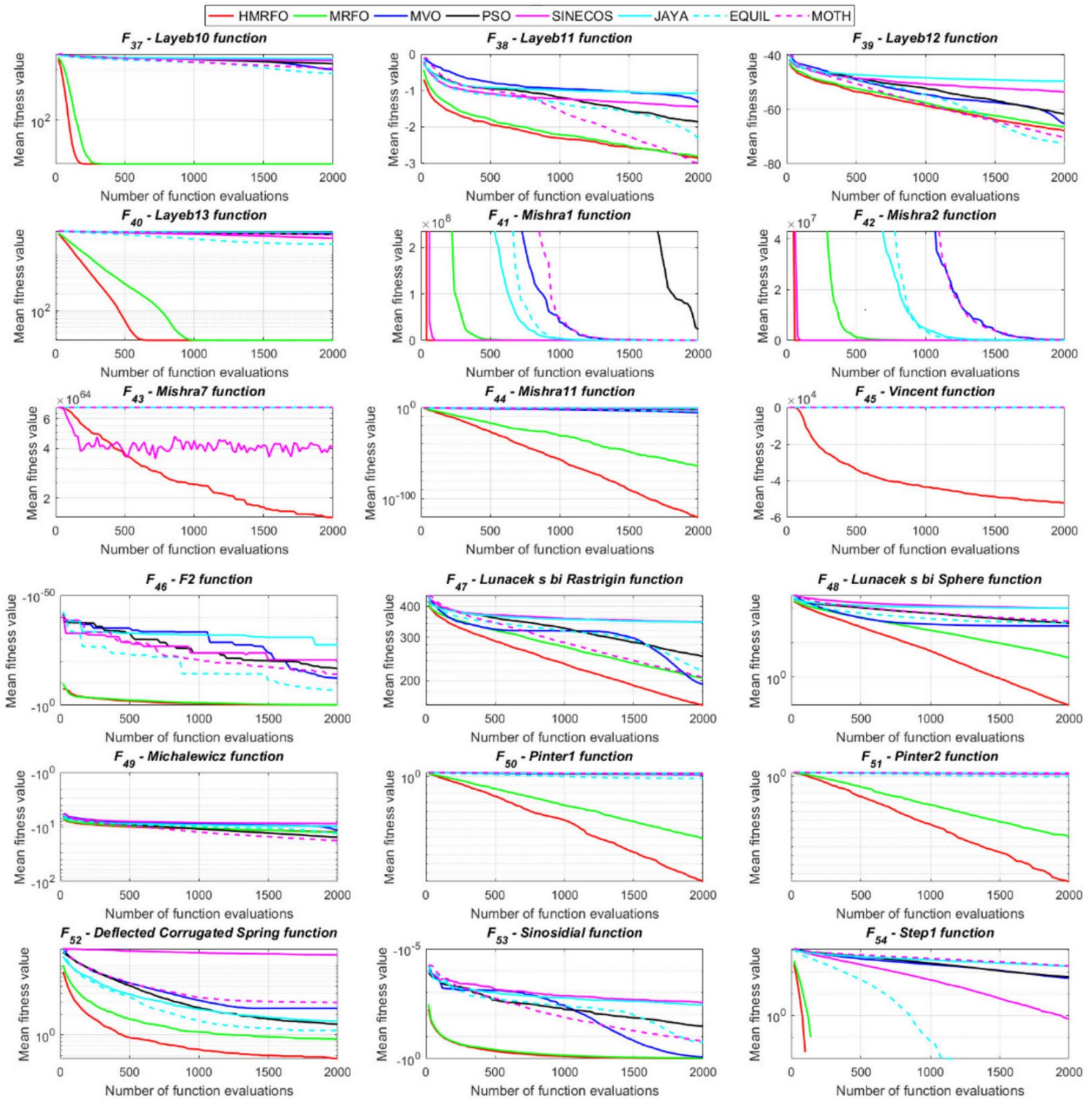
Convergence histories of the compared algorithms for the multimodal problems from F_37_–F_54_.

**Fig. 8:**
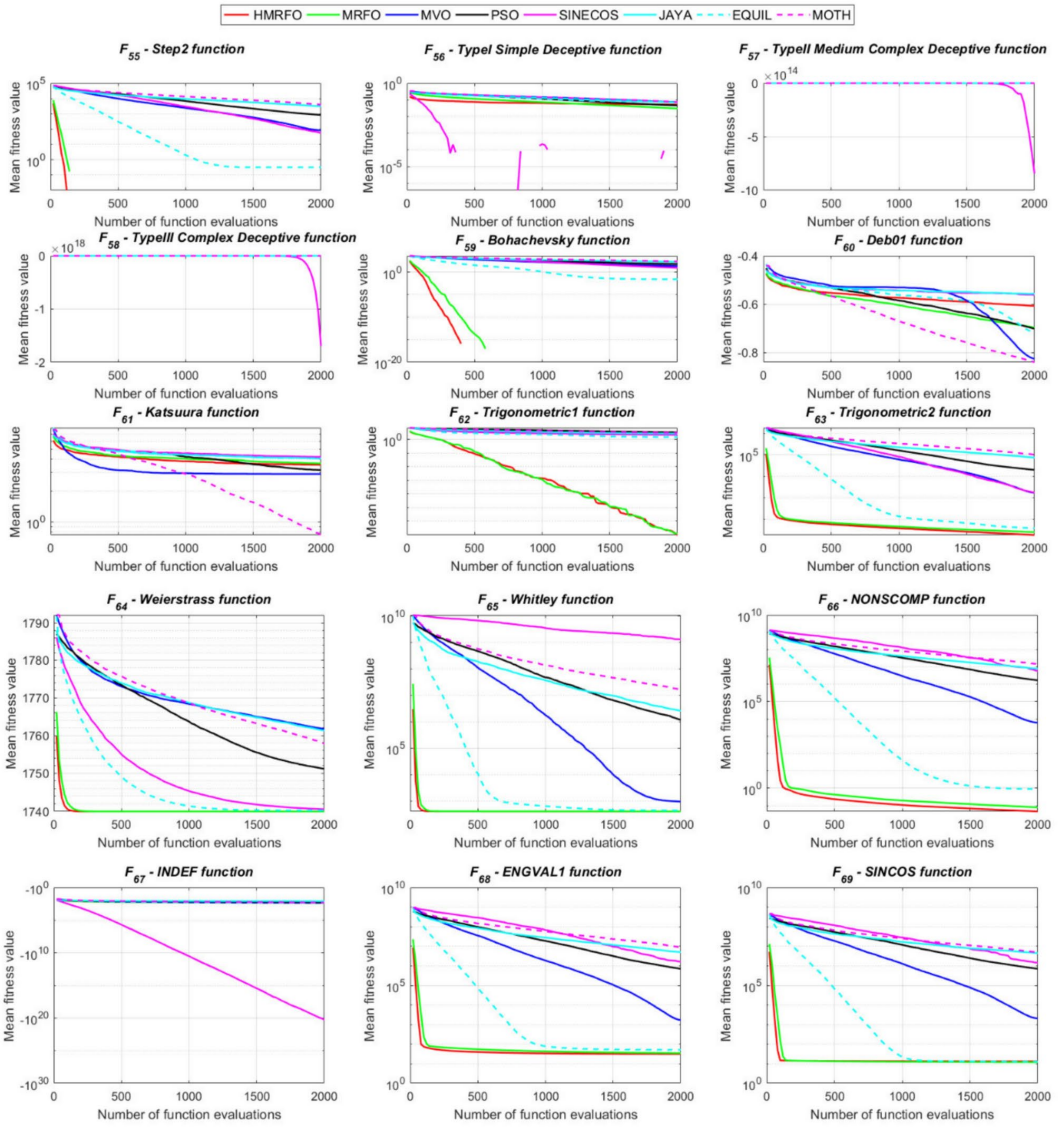
Evolution histories of the compared algorithms for the multimodal problems from F_55_–F_69_.

**Fig. 9:**
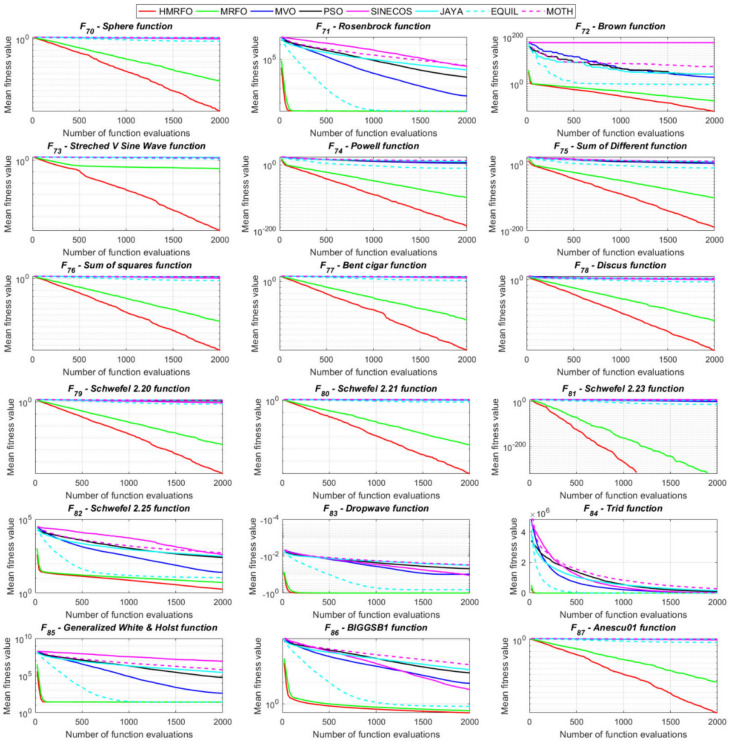
Convergence performances of the competitive algorithms for 30D unimodal test problems from F_70_ to F_87_.

**Fig. 10:**
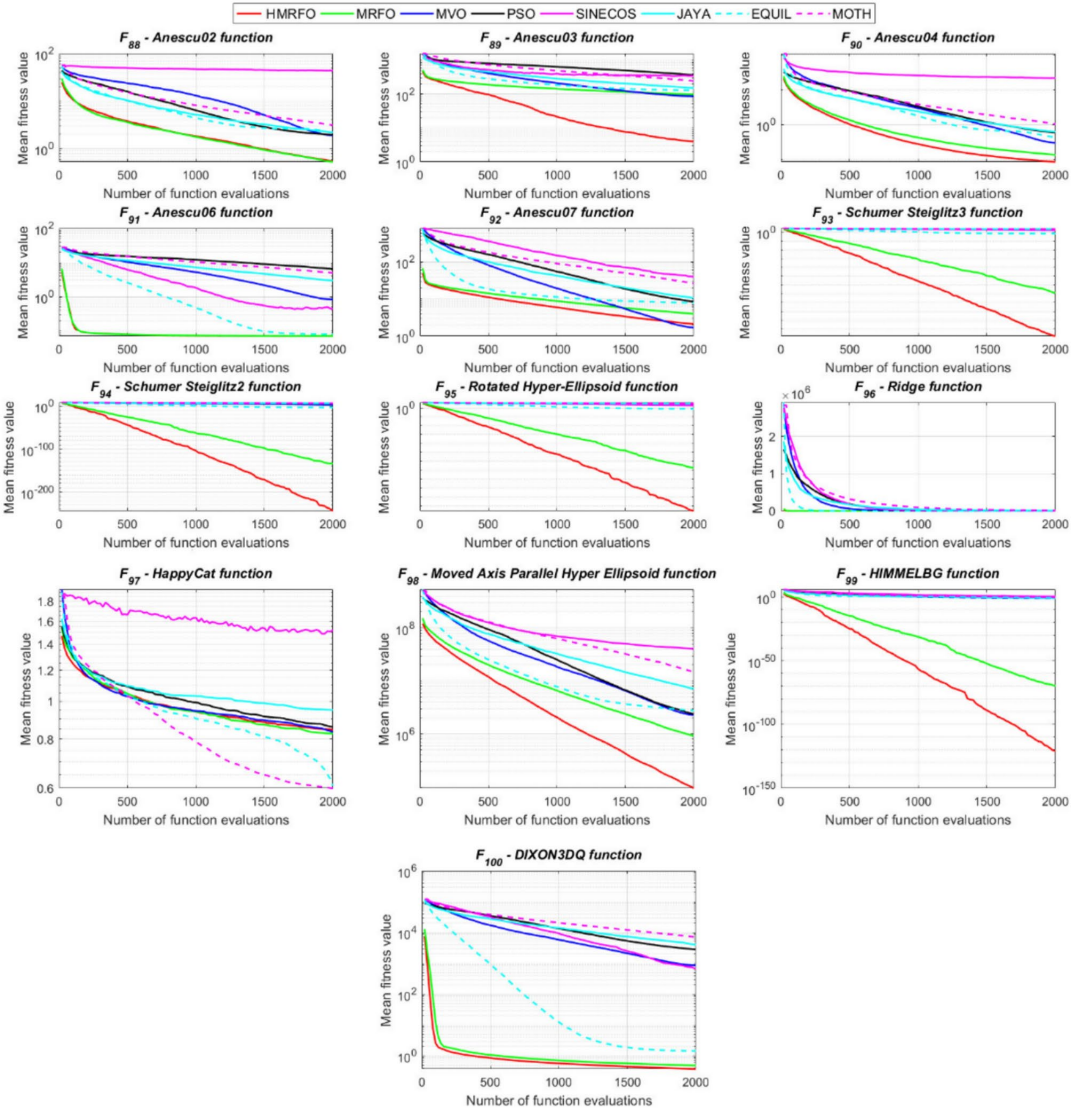
Optimal convergence of the contestant metaheuristic algorithms for 30D unimodal test problems from F_88_ to F_100_.

Tables 4 and 5 briefly describe the artificially generated multimodal and unimodal test functions utilized for the respective prediction performances of the compared algorithms. Table 6 reports the statistical results of 30D sixty-nine multimodal test functions for the compared algorithms. From Table 6, it can be observed that the HMRFO algorithm can find the global optimum solutions of F_1_, F_2_, F_3_, F_7_, F_8_, F_11_, F_14_, F_15_, F_54_, F_55_, F_59_, and F_64_ test functions within each algorithm run after 2000 function evaluations. When comparative performances of the tabulated algorithms are investigated in detail, it is seen that a considerable improvement in solution accuracies and persistency is seen in the condition where the hierarchical mutation scheme is embedded into the original MRFO algorithm. MRFO algorithm also provides better predictions than the literature optimizers, yet outperformed by the HMRFO algorithm in terms of mean and best results. HMRFO algorithm becomes the best-performing method in 46 out of 69 test problems and secures an undisputable lead for the 30-dimensional algorithms. The second best-performing optimizer between them is the original MRFO algorithm, which takes the leading method for 14 test problems. The JAYA optimizer is the metaheuristic algorithm yielding the worst prediction because of its insufficient exploration and exploitation abilities, which becomes obvious when lower functional evaluations are practiced. Other metaheuristics used in the comparative study also suffer from this algorithmic efficiency, making them ineffective in producing accurate predictions. Among the literature algorithms, the EQUIL optimizer gives the most accurate estimations by yielding the best prediction results for F_33_, F_36_, F_39_, and F_54_ test functions. Regarding the best fitness values, HMRFO is unequivocally superior to the remaining algorithms, taking the lead for fifty-two test problems. MRFO becomes the second-best performing algorithm between them when best values are considered, while EQUIL is the third best method. Finally, conclusive remarks can be given as introducing the hierarchical search scheme into the original MRFO algorithm significantly augments the probing capability, thanks to the useful contributions of different oppositional-based variants as well as quantum behaved mutation scheme, entailing a high-level improvement in producing the solution diversity to circumvent local solutions faced during iterations. Figures 5, 6, 7, and 8 visualize the convergence histories of the objective functions obtained for the competitive algorithms, including HMRFO. Two complementary search mechanisms govern convergence to the optimal solution during the iterative process. At the early phase of the iterations, carefully and meticulously probe around the solution domain to arrive at undiscovered regions where fruitful areas possibly reside. Then, after a fertile solution space is detected by the running algorithm during the exploration phase, a gradual decline to the best solution is observed by activating the exploitation mechanism. This convergence behavior is valid for most metaheuristics in the literature, which is conducive to reaching the global answer to the optimization problem. The decline in fitness rates shown in the figures also indicates that decision variables iteratively evolve into their optimal values. Contrary to the above-mentioned prevalent convergence behavior, HMRFO shows a gradual and stepwise decrease from the first to the last iteration in most cases. This is mostly because of the competent integrated mutation scheme of hierarchical learning, which enables the activation of more than one manipulative equation within a single iteration, emphasizing exploration and exploitation phases. However, this intelligently devised mechanism collapse for test functions of F_57_ and F_58_ does not make any progress until the end of the iterations. For the F_15_—Pathological 30D test function, gradual decreases with rapid declines are observed for HMRFO, obeying the general convenient convergence behavior. Rapid decreases at the early iterations following linear and gradual declines are observed for F_16_, F_27_, F_28_, F_31_, F_41_, F_42_, F_53_, F_63_, F_64_, F_65_, F_66_, F_68_, and F_69_ test functions contrary to the general convergence behavior. This tendency results from the functional characteristics of the applied test functions and the unexpected stagnation in progressing into the global optimum solution. In their seminal work, Derrac et al.^52^ stated that evaluating the statistical analysis of the collected results is essential to compare the given algorithms. Therefore, a non-parametric statistical test between the proposed HMRFO and other algorithms has been performed in this section to investigate the improvement made in HMFRO. It is accepted that the median value of the obtained prediction results is considered a relatively better statistical measure for assessing the comparative performance of two or more search methods. The statistical test has been performed at a 5% significance level, and retained results are reported in Table 7. This table provides the respective *p* values acquired by the Wilcoxon test. The symbols presented in this table have a particular meaning such that the symbol “+” indicates the proposed HMRFO is better than the compared algorithm, the symbol “−” represents that the compared algorithm shows better performance than HMRFO, lastly, “=” symbol stands for the expression that both algorithms give statistically same performance. According to the results given in Table 7, HMRFO outperforms the remaining algorithm in both terms of statistical significance and prediction accuracy for multimodal test problems. Table 8 reports the predictive results of 30D unimodal test problems for the competitive algorithms. HMRFO algorithm reaches the global best answer of the benchmark functions of F81 and F83 in each algorithm run. However, very close predictions of the best-known optimum solution are often observed. Interestingly, none of the algorithms reach the global best solution for any problem except HMRFO. Considering the average results, HMRFO becomes the dominant method in 27 out of 31 test problems among the given algorithms in Table 8. Standard MRFO is the second-best algorithm between them when average solutions are considered. EQUIL algorithm yields the most accurate predictions among the literature optimizers yet is significantly surpassed by HMRFO and MRFO algorithms. Figures 9 and 10 show the evolution of the fitness function values with increasing function evaluations for each algorithm for 30D unimodal test functions. In most test problems, linear decreases are observed rather than stepwise declines, mostly due to the balanced diversification intensification mechanism making it possible to evade the challenging local pitfalls and deny solution stagnation. Table 9 reports the Wilcoxon sum rank results on a classical 30D unimodal test function at a 5% significance level, which is made to ensure the algorithmic improvements made in HMRFO. The conclusion can be given that a significant enhancement was made in the search ability of HMRFO so that it can easily escape from the infertile areas and arrive in the promising regions, which made it successful in generating more diverse solutions compared to the remaining algorithms given in Table 9.

**Table 4:** Description of the multimodal test functions.

Function name	Dimension (D)	Range
F_1_—Ackley	30, 500	[− 32,32]^D^
F_2_—Griewank	30, 500	[− 600,600]^D^
F_3_—Rastrigin	30, 500	[− 5.12,5.12]^D^
F_4_—Zakharov	30, 500	[− 5.0,10.0]^D^
F_5_—Alpine	30, 500	[0,10]^D^
F_6_—Penalized1	30, 500	[− 50.0,50.0]^D^
F_7_—Csendes	30, 500	[− 5.0,5.0]^D^
F_8_—Schaffer	30, 500	[− 100.0,100.0]
F_9_—Salomon	30, 500	[− 50.0,50.0]^D^
F_10_—Inverted cosine mixture	30, 500	[− 10.0,10.0]^D^
F_11_—Wavy	30, 500	[− 3.14,3.14]^D^
F_12_—Xin She Yang1	30, 500	[− 5.0,5.0]^D^
F_13_—Xin She Yang2	30, 500	[− 6.28,6.28]^D^
F_14_—Xin She Yang4	30, 500	[− 10.0,10.0]^D^
F_15_—Pathological	30, 500	[− 10.0,10.0]^D^
F_16_—Quintic	30, 500	[− 10.0,10.0]^D^
F_17_—Levy	30, 500	[− 10.0,10.0]^D^
F_18_—Qing	30, 500	[− 500.0,500.0]^D^
F_19_—Diagonal1	30, 500	[− 10.0,10.0]^D^
F_20_—Hager	30, 500	[− 10.0,10.0]^D^
F_21_—Diagonal4	30, 500	[− 10.0,10.0]^D^
F_22_—Perturbed Quadratic Diagonal	30, 500	[− 10.0,10.0]^D^
F_23_—SINE	30, 500	[− 10.0,10.0]^D^
F_24_—Diagonal9	30, 500	[− 10.0,10.0]^D^
F_25_—COSINE	30, 500	[− 10.0,10.0]^D^
F_26_—Full Hessian FH3	30, 500	[− 10.0,10.0]^D^
F_27_—LIARWHD	30, 500	[− 10.0,10.0]^D^
F_28_—SINQUAD	30, 500	[− 10.0,10.0]^D^
F_29_—Styblinski –Tang	30, 500	[− 2.0,2.0]^D^
F_30_—Layeb03	30, 500	[− 10.0,10.0]^D^
F_31_—Layeb04	30, 500	[− 10.0,10.0]^D^
F_32_—Layeb05	30, 500	[− 10.0,10.0]^D^
F_33_—Layeb06	30, 500	[− 10.0,10.0]^D^
F_34_—Layeb07	30, 500	[− 10.0,10.0]^D^
F_35_—Layeb08	30, 500	[− 10.0,10.0]^D^
F_36_—Layeb09	30, 500	[− 10.0,10.0]^D^
F_37_—Layeb10	30, 500	[− 10.0,10.0]^D^
F_38_—Layeb11	30, 500	[− 10.0,10.0]^D^
F_39_—Layeb12	30, 500	[− 5.0,5.0]^D^
F_40_—Layeb13	30, 500	[− 10.0,10.0]^D^
F_41_—Mishra1	30, 500	[0.0,1.0]^D^
F_42_—Mishra2	30, 500	[0.0,1.0]^D^
F_43_—Mishra7	30, 500	[− 10.0,10.0]^D^
F_44_—Mishra11	30, 500	[− 10.0,10.0]^D^
F_45_—Vincent	30, 500	[0.0,10.0]^D^
F_46_—F2	30, 500	[0.0,1.0]^D^
F_47_—Lunacek’s bi Rastigin	30, 500	[− 5.12,5.12]^D^
F_48_—Lunacek’s bi Sphere	30, 500	[− 10.0,10.0]^D^
F_49_—Michalewicz	30, 500	[0.0,3.14]^D^
F_50_—Pinter1	30, 500	[− 10.0,10.0]^D^
F_51_—Pinter2	30, 500	[− 10.0,10.0]^D^
F_52_—Deflected Corrugated Spring	30, 500	[0.0,10.0]^D^
F_53_—Sinosidial	30, 500	[0.0,3.14]^D^
F_54_—Step1	30, 500	[− 100.0,100.0]^D^
F_55_—Step2	30, 500	[− 100.0,100.0]^D^
F_56_—Type—I Simple Deceptive	30, 500	[0.0,1.0]^D^
F_57—_Type—II Medium-Complex Deceptive	30, 500	[0.0,1.0]^D^
F_58_—Type—III Complex Deceptive	30, 500	[0.0,1.0]^D^
F_59_—Bohachevsky	30, 500	[− 15.0,15.0]^D^
F_60_—Deb01	30, 500	[− 1.0,1.0]^D^
F_61_—Katsuura	30, 500	[0.0,100.0]^D^
F_62_—Trigonometric1	30, 500	[0.0,3.14]^D^
F_63_—Trigonometric2	30, 500	[− 500.0,500.0]^D^
F_64_—Weierstrass	30, 500	[− 5.0,5.0]^D^
F_65_—Whitley	30, 500	[− 10.0,10.0]^D^
F_66_—NONSCOMP	30, 500	[− 100.0,100.0]^D^
F_67_—INDEF	30, 500	[− 10.0,10.0]^D^
F_68_—ENGVAL1	30, 500	[− 100.0,100.0]^D^
F_69_—SINCOS	30, 500	[− 100.0,100.0]^D^

**Table 5:** 30D and 500D unimodal test function used for performance evaluation of the algorithms.

Function name	Dimension	Range
F_70_—Sphere	30, 500	[− 5.12,5.12]^D^
F_71_—Rosenbrock	30, 500	[− 30.0,30.0]^D^
F_72_—Brown	30, 500	[− 1.0,4.0]^D^
F_73_—Streched V Sine Wave	30, 500	[− 10.0,10.0]^D^
F_74_—Powell	30, 500	[0.0,10.0]^D^
F_75_—Sum of Different Powers	30, 500	[− 1.0,1.0]^D^
F_76_—Sum of Squares	30, 500	[− 10.0,10.0]^D^
F_77_—Bent cigar	30, 500	[− 5.0,5.0]^D^
F_78_—Discus	30, 500	[− 100.0,100.0]^D^
F_79_—Schwefel 2.20	30, 500	[− 100.0,100.0]^D^
F_80_—Schwefel 2.21	30, 500	[− 100.0,100.0]^D^
F_81_—Schwefel 2.23	30, 500	[− 10.0,10.0]^D^
F_82_—Schwefel 2.25	30, 500	[0.0,10.0]^D^
F_83_—Dropwave	30, 500	[− 5.12,5.12]^D^
F_84_—Trid	30, 500	[D^2^,D^2^]^D^
F_85_—Generalized White & Holst	30, 500	[− 10.0,10.0]^D^
F_86_—BIGGSB1	30, 500	[− 10,10]^D^
F_87_—Anescu01	30, 500	[− 2.0,2.0]^D^
F_88_—Anescu02	30, 500	[1.39,4.0]^D^
F_89_—Anescu03	30, 500	[− 4.0,1.39]^D^
F_90_—Anescu04	30, 500	[0.001,2.0]^D^
F_91_—Anescu06	30, 500	[0.001,2.0]^D^
F_92_—Anescu07	30, 500	[− 2.0,2.0]^D^
F_93_—Schumer-Steiglitz 3	30, 500	[− 100.0,100.0]^D^
F_94_—Schumer-Steiglitz 2	30, 500	[− 100.0,100.0]^D^
F_95_—Rotated Hyper-Ellipsoid	30, 500	[− 100.0,100.0]^D^
F_96_—Ridge	30, 500	[− 5.0,5.0]^D^
F_97_—HappyCat	30, 500	[− 2.0,2.0]^D^
F_98_—Moved-Axis Parallel Hyperellipsoid	30, 500	[− 100.0,100.0]^D^
F_99_—HIMMELBG	30, 500	[− 10.0,10.0]^D^
F_100_—DIXON3DQ	30, 500	[− 100.0,100.0]^D^

**Table 6:** Statistical comparison between the competitive algorithms for 30D multimodal functions.

		HMRFO	MRFO	MVO	PSO	SINECOS	JAYA	EQUIL	MOTH
F_1_	Min	**8.88E−16**	**8.88E−16**	1.76E+00	2.41E+00	5.48E−02	4.54E+00	1.71E−03	4.25E+00
	Mean	8.88E−16	8.88E−16	2.73E+00	3.92E+00	6.42E−01	5.57E+00	5.27E−03	5.84E+00
	Std. dev	0.00E+00	0.00E+00	5.09E−01	6.51E−01	6.60E−01	4.58E−01	2.24E−03	5.96E−01
	Max	8.88E−16	8.88E−16	4.15E+00	5.67E+00	3.09E+00	6.84E+00	1.33E−02	7.43E+00
F_2_	Min	**0.00E+00**	**0.00E+00**	3.76E−02	8.00E−02	1.66E−04	6.21E−01	6.81E−07	3.36E−01
	Mean	0.00E+00	0.00E+00	8.83E−02	3.38E−01	2.60E−01	8.66E−01	1.84E−03	8.16E−01
	Std. dev	0.00E+00	0.00E+00	2.86E−02	1.62E−01	2.96E−01	8.52E−02	9.35E−03	1.09E−01
	Max	0.00E+00	0.00E+00	1.64E−01	8.06E−01	9.24E−01	1.00E+00	7.94E−02	1.07E+00
F_3_	Min	**0.00E+00**	**0.00E+00**	1.28E+02	9.07E+01	2.39E+01	2.21E+02	1.42E+00	1.03E+02
	Mean	0.00E+00	0.00E+00	2.31E+02	1.91E+02	1.51E+02	3.10E+02	2.27E+01	2.34E+02
	Std. dev	0.00E+00	0.00E+00	5.01E+01	4.08E+01	6.42E+01	3.05E+01	1.31E+01	4.43E+01
	Max	0.00E+00	0.00E+00	3.99E+02	2.82E+02	3.34E+02	3.98E+02	6.28E+01	3.92E+02
F_4_	Min	**1.83E−173**	1.20E−85	8.61E+01	2.45E+02	1.01E+02	1.96E+02	2.68E+01	2.01E+02
	Mean	5.65E−126	1.50E−69	2.59E+02	3.77E+02	3.36E+02	4.01E+02	1.10E+02	4.11E+02
	Std. dev	4.04E−125	1.00E−68	8.44E+01	6.41E+01	1.15E+02	8.27E+01	3.90E+01	8.55E+01
	Max	3.32E−86	9.13E−68	4.53E+02	5.31E+02	7.11E+02	6.57E+02	2.34E+02	5.81E+02
F_5_	Min	**3.89E−80**	2.07E−44	2.61E+00	2.58E+00	1.39E−02	1.67E+01	2.30E−03	5.65E+00
	Mean	1.22E−62	1.81E−37	1.25E+01	8.70E+00	7.03E+00	2.45E+01	1.10E−02	1.15E+01
	Std. dev	9.28E−62	8.92E−37	3.36E+00	3.52E+00	6.80E+00	3.51E+00	1.60E−02	2.42E+00
	Max	9.18E−61	7.12E−36	2.30E+01	1.87E+01	3.40E+01	3.18E+01	1.23E−01	1.72E+01
F_6_	Min	**6.33E−04**	1.12E−03	4.95E−01	4.12E−02	4.02E−01	2.33E+00	9.61E−03	3.42E−01
	Mean	6.40E−03	1.28E−02	2.03E+00	5.83E−01	2.19E+00	4.46E+00	3.84E−02	1.31E+00
	Std. dev	6.10E−03	1.24E−02	8.86E−01	3.21E−01	2.15E+00	1.1E+00	1.76E−02	5.26E−01
	Max	2.88E−02	1.11E−01	4.74E+00	1.67E+00	1.08E+01	7.57E+00	9.07E−02	2.84E+00
F_7_	Min	**0.00E+00**	3.1E−275	7.6E−04	4.78E+01	5.08E−02	7.9E+02	3.09E−13	1.06E+03
	Mean	0.00E+00	1.9E−209	1.98E−01	1.64E+03	2.17E+05	7.32E+03	1.88E−09	1.43E+04
	Std. dev	0.00E+00	0.00E+00	3.31E−01	2.59E+03	5.53E+05	5.86E+03	6.52E−09	1.18E+04
	Max	0.00E+00	1.8E−207	2.02E+00	1.62E+04	3.81E+06	3.11E+04	5.71E−08	6.66E+04
F_8_	Min	**0.00E+00**	0.00E+00	1.59E+00	2.71E+00	5.11E+00	6.07E+00	2.11E+00	1.95E+00
	Mean	0.00E+00	0.00E+00	2.72E+00	5.62E+00	7.91E+00	8.13E+00	3.84E+00	3.33E+00
	Std. dev	0.00E+00	0.00E+00	5.70E−01	1.28E+00	7.40E−01	5.84E−01	8.58E−01	6.96E−01
	Max	0.00E+00	0.00E+00	3.96E+00	8.00E+00	9.39E+00	9.18E+00	6.04E+00	5.61E+00
F_9_	Min	**2.15E−81**	1.32E−41	5.99E−01	7.16E−01	4.24E−01	1.00E+00	1.99E−01	9.99E−01
	Mean	1.41E−59	1.05E−18	1.09E+00	1.10E+00	1.02E+00	1.29E+00	3.34E−01	1.40E+00
	Std. dev	1.39E−58	1.05E−17	2.02E−01	1.22E−01	2.74E−01	1.40E−01	7.43E−02	1.71E−01
	Max	1.39E−57	1.05E−16	1.49E+00	1.49E+00	1.99E+00	1.66E+00	4.99E−01	1.79E+00
F_10_	Min	**6.65E−168**	2.29E−90	2.28E+00	4.70E+00	1.60E−02	1.48E+01	1.76E−04	1.70E+01
	Mean	1.69E−121	3.53E−72	3.75E+00	1.16E+01	2.03E+00	3.34E+01	2.48E−03	4.12E+01
	Std. dev	1.67E−120	2.89E−71	7.05E−01	5.45E+00	2.66E+00	9.05E+00	1.50E−02	1.23E+01
	Max	1.67E−119	2.87E−70	6.02E+00	3.31E+01	2.11E+01	6.16E+01	1.51E−01	8.53E+01
F_11_	Min	**0.00E+00**	**0.00E+00**	6.19E−01	6.06E−01	2.31E−01	7.72E−01	3.38E−01	5.76E−01
	Mean	0.00E+00	0.00E+00	7.46E−01	7.32E−01	5.82E−01	8.26E−01	5.49E−01	6.52E−01
	Std. dev	0.00E+00	0.00E+00	3.69E−02	4.15E−02	1.13E−01	2.04E−02	8.03E−02	4.02E−02
	Max	0.00E+00	0.00E+00	8.09E−01	8.28E−01	7.76E−01	8.64E−01	7.10E−01	7.40E−01
F_12_	Min	**2.64E−108**	3.12E−39	1.42E+02	2.54E+03	4.66E−03	1.61E+04	2.49E−10	2.76E+04
	Mean	2.30E−84	2.74E−08	9.17E+07	4.72E+07	3.15E+06	4.84E+08	6.09E−06	8.81E+09
	Std. dev	2.28E−83	2.74E−07	4.85E+08	1.55E+08	2.04E+07	2.69E+09	4.11E−05	5.84E+10
	Max	2.28E−82	2.74E−06	3.94E+09	1.08E+09	1.91E+08	2.62E+10	3.88E−04	5.57E+11
F_13_	Min	6.46E−10	**4.09E−12**	7.47E−10	1.84E−09	8.94E−07	3.18E−07	2.41E−10	6.34E−11
	Mean	3.75E−07	1.54E−08	2.93E−07	3.07E−06	1.87E−05	6.08E−05	4.22E−08	6.99E−09
	Std. dev	1.59E−06	1.36E−07	8.02E−07	8.60E−06	2.28E−05	5.69E−05	1.23E−07	1.27E−08
	Max	1.49E−05	1.35E−06	6.16E−06	6.46E−05	1.68E−04	2.92E−04	1.09E−06	9.86E−08
F_14_	Min	**− 1.00E+00**	**− 1.00E+00**	1.27E−13	4.31E−13	2.96E−11	2.28E−11	5.85E−13	1.05E−12
	Mean	− 1.00E+00	− 1.00E+00	3.58E−13	9.70E−13	1.33E−10	1.27E−10	1.05E−12	3.06E−12
	Std. dev	0.00E+00	0.00E+00	1.95E−13	4.25E−13	7.17E−11	7.39E−11	2.74E−13	1.24E−12
	Max	− 1.00E+00	− 1.00E+00	1.25E−12	2.91E−12	3.50E−10	4.00E−10	2.14E−12	8.03E−12
F_15_	Min	**0.00E+00**	**0.00E+00**	6.07E+00	6.73E+00	8.32E+00	8.59E+00	6.13E+00	3.91E+00
	Mean	0.00E+00	4.72E−01	8.18E+00	8.88E+00	9.74E+00	1.01E+01	8.25E+00	6.34E+00
	Std. dev	0.00E+00	1.93E+00	7.07E−01	8.08E−01	5.27E−01	4.69E−01	8.21E−01	8.93E−01
	Max	0.00E+00	9.19E+00	9.98E+00	1.03E+01	1.08E+01	1.09E+01	1.03E+01	8.81E+00
F_16_	Min	**1.91E+00**	4.32E+00	3.79E+01	7.07E+01	9.58E+01	1.64E+02	1.15E+01	1.96E+02
	Mean	1.10E+01	1.43E+01	6.83E+01	2.32E+02	5.46E+03	5.70E+02	2.88E+01	1.68E+03
	Std. dev	5.07E+00	5.23E+00	1.45E+01	2.85E+02	3.22E+04	3.56E+02	7.03E+00	9.11E+02
	Max	2.66E+01	2.92E+01	1.09E+02	2.64E+03	3.19E+05	2.43E+03	4.81E+01	5.76E+03
F_17_	Min	**2.55E−04**	1.07E−02	3.94E+00	2.68E−01	2.54E+00	3.07E+00	7.24E−02	3.22E+00
	Mean	2.14E−03	1.05E−01	2.12E+01	4.55E+00	8.34E+00	9.44E+00	3.49E−01	1.02E+01
	Std. dev	6.16E−03	6.84E−02	9.38E+00	2.81E+00	6.75E+00	4.56E+00	1.61E−01	4.13E+00
	Max	6.19E−02	3.25E−01	4.97E+01	1.34E+01	4.44E+01	3.38E+01	9.00E−01	2.45E+01
F_18_	Min	6.98E+02	**1.54E+01**	1.92E+01	1.09E+02	3.67E+03	1.08E+03	1.28E+02	3.86E+02
	Mean	1.64E+03	9.87E+01	9.31E+01	4.96E+02	6.06E+03	2.84E+03	5.12E+02	9.03E+02
	Std. dev	5.96E+02	1.09E+02	6.02E+01	2.49E+02	2.19E+03	5.63E+02	2.40E+02	2.89E+02
	Max	3.02E+03	8.12E+02	3.71E+02	1.25E+03	2.00E+04	4.05E+03	1.25E+03	1.72E+03
F_19_	Min	**− 8.91E+02**	− 8.88E+02	− 8.82E+02	− 8.62E+02	− 4.32E+02	− 8.07E+02	− 8.71E+02	− 7.48E+02
	Mean	− 8.90E+02	− 8.64E+02	− 8.38E+02	− 7.74E+02	− 2.48E+02	− 7.12E+02	− 7.86E+02	− 6.07E+02
	Std. dev	1.33E+00	2.04E+01	2.69E+01	5.52E+01	9.25E+01	4.97E+01	4.15E+01	7.35E+01
	Max	− 8.83E+02	− 7.83E+02	− 7.56E+02	− 6.15E+02	1.49E+02	− 4.98E+02	− 6.81E+02	− 3.95E+02
F_20_	Min	**− 4.25E+01**	− 4.22E+01	− 4.12E+01	− 2.99E+01	− 3.84E+00	− 2.57E+01	− 3.97E+01	− 3.62E+00
	Mean	− 4.23E+01	− 4.02E+01	− 3.50E+01	− 3.85E+00	1.63E+01	− 1.30E+00	− 3.20E+01	3.82E+01
	Std. dev	3.94E−01	2.03E+00	4.86E+00	1.71E+01	1.18E+01	1.28E+01	3.68E+00	2.03E+01
	Max	− 3.94E+01	− 3.13E+01	− 1.51E+01	4.91E+01	6.79E+01	3.03E+01	− 2.03E+01	8.72E+01
F_21_	Min	**6.39E−157**	1.49E−89	4.33E+01	6.75E+00	2.02E−02	7.79E+01	4.81E−05	7.45E+01
	Mean	1.74E−120	1.02E−72	1.22E+02	2.76E+01	1.53E+00	1.63E+02	6.27E−04	1.83E+02
	Std. dev	1.74E−119	8.68E−72	3.24E+01	1.33E+01	2.52E+00	4.58E+01	5.12E−04	6.10E+01
	Max	1.74E−118	8.62E−71	2.19E+02	7.32E+01	2.01E+01	3.15E+02	2.75E−03	4.34E+02
F_22_	Min	**1.13E−163**	2.25E−88	4.28E−01	4.94E+00	1.07E−01	1.39E+01	1.49E−03	6.69E+00
	Mean	8.54E−125	2.35E−71	1.87E+00	1.32E+01	7.19E+00	2.86E+01	2.45E−02	1.68E+01
	Std. dev	6.16E−124	2.24E−70	7.64E−01	4.11E+00	5.68E+00	5.95E+00	2.18E−02	5.04E+00
	Max	5.26E−123	2.24E−69	4.34E+00	2.51E+01	2.25E+01	4.51E+01	1.65E−01	3.14E+01
F_23_	Min	− 2.40E+01	− 2.25E+01	**− 2.45E+01**	− 2.23E+01	− 1.73E+01	− 1.78E+01	− 2.41E+01	− 2.73E+01
	Mean	− 1.51E+01	− 1.48E+01	− 2.04E+01	− 1.56E+01	− 1.27E+01	− 1.32E+01	− 1.78E+01	− 2.35E+01
	Std. dev	2.52E+00	1.91E+00	1.87E+00	1.81E+00	1.29E+00	1.33E+00	2.42E+00	1.53E+00
	Max	− 1.18E+01	− 1.19E+01	− 1.52E+01	− 1.21E+01	− 1.02E+01	− 1.09E+01	− 1.33E+01	− 1.92E+01
F_24_	Min	**− 8.19E+02**	− 8.15E+02	− 6.01E+02	− 7.83E+02	− 4.14E+02	− 7.24E+02	− 7.87E+02	− 6.50E+02
	Mean	− 8.17E+02	− 7.71E+02	− 3.13E+02	− 6.76E+02	− 2.30E+02	− 5.99E+02	− 7.16E+02	− 4.89E+02
	Std. dev	3.87E+00	3.59E+01	1.45E+02	6.09E+01	9.22E+01	5.21E+01	4.40E+01	7.69E+01
	Max	− 7.91E+02	− 6.40E+02	1.51E+02	− 5.04E+02	3.28E+01	− 4.20E+02	− 6.21E+02	− 3.38E+02
F_25_	Min	− 2.46E+01	− 2.21E+01	**− 2.52E+01**	− 2.13E+01	− 1.77E+01	− 1.77E+01	− 2.42E+01	− 2.66E+01
	Mean	− 1.50E+01	− 1.48E+01	− 2.03E+01	− 1.60E+01	− 1.27E+01	− 1.32E+01	− 1.81E+01	− 2.33E+01
	Std. dev	2.38E+00	1.96E+00	1.91E+00	1.94E+00	1.43E+00	1.30E+00	2.28E+00	1.53E+00
	Max	− 1.16E+01	− 1.18E+01	− 1.54E+01	− 1.18E+01	− 1.03E+01	− 1.07E+01	− 1.35E+01	− 1.94E+01
F_26_	Min	− 2.37E+02	− 2.24E+02	**− 2.48E+02**	− 1.54E+02	− 1.92E+03	− 1.41E+02	− 2.03E+02	− 1.18E+02
	Mean	− 1.77E+02	− 1.65E+02	− 1.67E+02	− 6.88E+01	− 6.88E+01	− 3.55E+01	− 1.48E+02	− 2.61E+01
	Std. dev	2.73E+01	3.03E+01	3.08E+01	3.21E+01	2.16E+02	3.52E+01	2.67E+01	6.13E+01
	Max	− 8.84E+01	− 8.74E+01	− 9.98E+01	8.96E+00	9.77E+01	9.32E+01	− 8.55E+01	2.81E+02
F_27_	Min	**3.13E−02**	3.5E+00	1.49E+01	9.85E+01	2.78E+01	1.48E+02	1.30E+01	4.14E+02
	Mean	1.04E+00	9.9E+00	3.38E+01	4.10E+02	2.13E+03	8.87E+02	1.65E+01	1.74E+03
	Std. dev	1.52E+00	2.0E+00	1.20E+01	2.06E+02	5.46E+03	4.81E+02	1.56E+00	7.96E+02
	Max	6.26E+00	1.46E+01	8.41E+01	1.03E+03	3.53E+04	2.67E+03	2.10E+01	5.20E+03
F_28_	Min	**6.56E−04**	2.52E−03	1.07E+00	3.42E+01	4.46E+00	5.74E+01	1.45E−02	1.67E+02
	Mean	5.58E−02	2.75E−02	8.25E+01	2.82E+02	3.74E+03	4.38E+02	1.16E−01	7.33E+02
	Std. dev	5.21E−02	2.56E−02	1.07E+02	1.95E+02	4.96E+03	2.60E+02	5.64E−02	3.41E+02
	Max	1.28E−01	1.20E−01	7.12E+02	1.03E+03	1.96E+04	1.53E+03	3.47E−01	1.53E+03
F_29_	Min	− 6.39E+02	− 6.31E+02	**− 6.57E+02**	− 4.94E+02	− 4.87E+02	− 4.67E+02	− 5.97E+02	− 6.06E+02
	Mean	− 5.28E+02	− 5.13E+02	− 5.84E+02	− 4.24E+02	− 3.84E+02	− 4.16E+02	− 5.20E+02	− 5.26E+02
	Std. dev	4.03E+01	3.85E+01	3.95E+01	2.47E+01	4.30E+01	1.98E+01	3.04E+01	3.25E+01
	Max	− 4.59E+02	− 4.25E+02	− 4.61E+02	− 3.60E+02	− 3.10E+02	− 3.59E+02	− 4.40E+02	− 4.27E+02
F_30_	Min	1.40E−03	**1.39E−03**	1.40E−03	1.40E−03	1.43E−03	1.44E−03	1.39E−03	1.42E−03
	Mean	1.41E−03	1.39E−03	1.47E−03	1.42E−03	1.46E−03	1.48E−03	1.40E−03	1.44E−03
	Std. dev	5.23E−06	2.38E−06	2.94E−05	1.17E−05	1.27E−05	1.60E−05	1.22E−06	1.68E−05
	Max	1.42E−03	1.41E−03	1.55E−03	1.45E−03	1.49E−03	1.52E−03	1.40E−03	1.49E−03
F_31_	Min	**− 1.87E+02**	− 1.86E+02	− 6.41E+01	− 1.41E+02	− 1.91E+02	− 5.27E+01	− 1.86E+02	− 1.08E+02
	Mean	− 1.76E+02	− 1.72E+02	− 3.44E+01	− 1.05E+02	− 1.81E+02	− 3.29E+01	− 1.76E+02	− 8.10E+01
	Std. dev	3.85E+00	2.70E+00	1.41E+01	1.68E+01	3.55E+00	8.56E+00	3.90E+00	1.12E+01
	Max	− 1.71E+02	− 1.71E+02	− 3.21E+00	− 6.15E+01	− 1.70E+02	− 1.05E+01	− 1.71E+02	− 4.99E+01
F_32_	Min	− 6.17E+01	**− 1.01E+02**	− 8.27E+01	− 7.76E+01	− 4.62E+01	− 4.32E+01	− 7.57E+01	− 9.05E+01
	Mean	− 4.23E+01	− 4.82E+01	− 5.86E+01	− 4.59E+01	− 3.48E+01	− 3.28E+01	− 5.79E+01	− 6.65E+01
	Std. dev	5.76E+00	1.20E+01	1.03E+01	1.01E+01	3.48E+00	2.71E+00	9.10E+00	8.68E+00
	Max	− 3.28E+01	− 3.06E+01	− 3.49E+01	− 2.97E+01	− 2.85E+01	− 2.85E+01	− 4.04E+01	− 4.98E+01
F_33_	Min	1.94E+01	**1.88E+01**	2.04E+01	1.88E+01	2.38E+01	2.46E+01	1.93E+01	2.00E+01
	Mean	2.23E+01	2.21E+01	2.28E+01	2.23E+01	2.51E+01	2.51E+01	2.15E+01	2.15E+01
	Std. dev	1.31E+00	1.51E+00	9.30E−01	1.39E+00	3.81E−01	2.37E−01	8.83E−01	7.04E−01
	Max	2.51E+01	2.55E+01	2.47E+01	2.50E+01	2.58E+01	2.57E+01	2.37E+01	2.29E+01
F_34_	Min	**6.93E+01**	**6.93E+01**	2.33E+03	2.35E+03	2.52E+03	2.51E+03	2.35E+03	2.20E+03
	Mean	6.93E+01	6.93E+01	2.45E+03	2.53E+03	2.58E+03	2.58E+03	2.47E+03	2.32E+03
	Std. dev	8.56E−14	8.56E−14	4.96E+01	4.23E+01	2.07E+01	1.73E+01	4.47E+01	5.25E+01
	Max	6.93E+01	6.93E+01	2.56E+03	2.60E+03	2.62E+03	2.62E+03	2.57E+03	2.46E+03
F_35_	Min	8.33E+02	4.73E+02	**4.34E+02**	7.40E+02	1.11E+03	1.06E+03	6.75E+02	4.87E+02
	Mean	1.14E+03	9.38E+02	6.88E+02	1.13E+03	1.30E+03	1.29E+03	9.61E+02	7.41E+02
	Std. dev	1.06E+02	2.00E+02	1.17E+02	1.27E+02	6.34E+01	5.92E+01	1.14E+02	1.09E+02
	Max	1.33E+03	1.30E+03	1.03E+03	1.34E+03	1.41E+03	1.41E+03	1.21E+03	9.78E+02
F_36_	Min	2.46E+01	2.05E+01	2.24E+01	**1.81E+01**	2.77E+01	2.85E+01	2.02E+01	2.23E+01
	Mean	2.82E+01	2.72E+01	2.77E+01	2.24E+01	4.80E+01	4.33E+01	1.64E+01	2.77E+01
	Std. dev	2.14E+00	3.93E+00	2.66E+00	6.94E+00	1.78E+01	7.45E+00	5.46E+00	2.62E+00
	Max	3.39E+01	3.39E+01	3.49E+01	3.26E+01	1.18E+02	6.26E+01	7.11E+00	3.59E+01
F_37_	Min	**1.39E+01**	**1.39E+01**	7.37E+02	8.30E+02	9.20E+02	1.42E+03	7.42E+01	8.07E+02
	Mean	1.39E+01	1.39E+01	9.82E+02	1.28E+03	1.48E+03	1.60E+03	8.29E+02	1.03E+03
	Std. dev	5.35E−15	5.35E−15	1.08E+02	1.75E+02	1.33E+02	5.84E+01	2.17E+02	1.09E+02
	Max	1.39E+01	1.39E+01	1.34E+03	1.58E+03	1.70E+03	1.73E+03	1.20E+03	1.33E+03
F_38_	Min	**− 6.42E+00**	− 5.51E+00	− 2.91E+00	− 3.96E+00	− 2.44E+00	− 1.90E+00	− 4.02E+00	− 4.78E+00
	Mean	− 2.86E+00	− 2.81E+00	− 1.32E+00	− 1.87E+00	− 1.44E+00	− 1.08E+00	− 2.32E+00	− 3.02E+00
	Std. dev	8.98E−01	8.18E−01	4.46E−01	5.89E−01	3.27E−01	2.22E−01	6.01E−01	7.31E−01
	Max	− 1.74E+00	− 1.72E+00	− 8.60E−01	− 9.35E−01	− 9.67E−01	− 8.57E−01	− 1.00E+00	− 1.61E+00
F_39_	Min	**− 8.41E+01**	− 8.31E+01	− 7.90E+01	− 7.89E+01	− 6.15E+01	− 5.49E+01	− 8.50E+01	− 8.29E+01
	Mean	− 6.77E+01	− 6.63E+01	− 6.51E+01	− 6.16E+01	− 5.34E+01	− 4.95E+01	− 7.26E+01	− 7.03E+01
	Std. dev	9.47E+00	6.43E+00	5.41E+00	5.61E+00	3.67E+00	1.97E+00	5.09E+00	4.49E+00
	Max	− 5.01E+01	− 5.18E+01	− 5.36E+01	− 5.06E+01	− 4.52E+01	− 4.56E+01	− 6.23E+01	− 6.02E+01
F_40_	Min	**2.90E+00**	**2.90E+00**	2.34E+03	2.22E+03	1.28E+03	2.51E+03	1.32E+03	2.26E+03
	Mean	2.90E+00	2.90E+00	2.51E+03	2.37E+03	2.02E+03	2.60E+03	1.59E+03	2.36E+03
	Std. dev	0.00E+00	0.00E+00	5.86E+01	7.01E+01	3.20E+02	2.58E+01	1.11E+02	5.23E+01
	Max	2.90E+00	2.90E+00	2.62E+03	2.53E+03	2.60E+03	2.65E+03	1.87E+03	2.51E+03
F_41_	Min	**1.14E−204**	2.49E+01	1.37E+02	6.05E+04	0.00E+00	3.32E+02	3.58E+02	2.03E+03
	Mean	1.45E−28	4.18E+02	3.99E+04	2.32E+07	0.00E+00	8.34E+03	6.03E+03	6.27E+04
	Std. dev	2.11E−26	5.90E+02	1.29E+05	5.28E+07	0.00E+00	1.36E+04	8.06E+03	1.46E+05
	Max	1.36E−26	3.88E+03	1.01E+06	3.66E+08	0.00E+00	1.16E+05	5.76E+04	1.07E+06
F_42_	Min	**1.12E−175**	1.94E+01	1.26E+02	4.36E+04	0.00E+00	8.27E+02	3.96E+02	1.26E+03
	Mean	9.22E−32	4.25E+02	1.95E+05	5.90E+07	0.00E+00	1.12E+04	7.88E+03	1.15E+05
	Std. dev	9.26E−31	9.03E+02	8.58E+05	1.86E+08	0.00E+00	1.30E+04	1.11E+04	2.68E+05
	Max	9.22E−30	7.81E+03	7.70E+06	1.16E+09	0.00E+00	9.22E+04	5.91E+04	1.66E+06
F_43_	Min	**1.15E+59**	7.03E+64	7.03E+64	7.03E+64	1.55E+59	7.03E+64	7.03E+64	7.03E+64
	Mean	1.53E+64	7.03E+64	7.03E+64	7.03E+64	4.04E+64	7.03E+64	7.03E+64	7.03E+64
	Std. dev	2.57E+64	6.94E+59	8.57E+60	3.44E+56	2.63E+64	4.14E+56	1.10E+58	3.39E+58
	Max	7.03E+64	7.03E+64	7.03E+64	7.03E+64	7.03E+64	7.03E+64	7.03E+64	7.03E+64
F_44_	Min	**3.61E−164**	8.08E−92	3.79E−07	1.53E−04	2.61E−02	5.93E−02	8.80E−07	6.08E−04
	Mean	3.43E−121	9.36E−65	2.84E−06	4.16E−03	2.12E−01	1.38E−01	4.37E−05	3.77E−03
	Std. dev	3.42E−120	9.19E−64	2.41E−06	4.97E−03	7.64E−02	3.90E−02	4.94E−05	2.50E−03
	Max	3.42E−119	9.19E−63	1.32E−05	3.15E−02	4.47E−01	2.44E−01	2.06E−04	1.38E−02
F_45_	Min	**− 2.83E+04**	− 9.98E−01	− 9.94E−01	− 9.94E−01	− 5.71E−02	− 9.45E−01	− 9.88E−01	− 9.79E−01
	Mean	− 5.20E+04	− 9.81E−01	− 9.54E−01	− 9.75E−01	− 8.63E−03	− 8.43E−01	− 9.73E−01	− 9.37E−01
	Std. dev	1.79E+04	4.16E−02	3.64E−02	1.50E−02	2.29E−02	6.81E−02	9.12E−03	2.46E−02
	Max	− 7.54E+04	− 6.79E−01	− 7.76E−01	− 9.25E−01	4.94E−02	− 6.73E−01	− 9.34E−01	− 7.88E−01
F_46_	Min	**− 9.94E−01**	− 9.51E−01	− 4.90E−11	− 1.74E−15	− 2.00E−19	− 3.13E−26	− 1.40E−05	− 8.02E−13
	Mean	− 6.81E−01	− 6.20E−01	− 5.05E−13	− 2.59E−17	− 2.20E−21	− 3.13E−28	− 1.45E−07	− 9.41E−15
	Std. dev	3.30E−01	2.80E−01	4.90E−12	1.85E−16	2.01E−20	3.13E−27	1.39E−06	8.07E−14
	Max	− 4.64E−04	− 6.18E−06	− 3.42E−28	− 1.89E−32	− 2.68E−39	− 1.08E−42	− 1.10E−25	− 1.30E−26
F_47_	Min	**6.34E+01**	8.73E+01	1.39E+02	1.22E+02	2.95E+02	2.98E+02	1.58E+02	1.47E+02
	Mean	1.58E+02	2.04E+02	1.93E+02	2.50E+02	3.45E+02	3.44E+02	2.20E+02	2.05E+02
	Std. dev	5.38E+01	3.98E+01	2.44E+01	3.65E+01	1.61E+01	1.58E+01	2.39E+01	2.36E+01
	Max	3.33E+02	3.20E+02	2.51E+02	3.23E+02	3.78E+02	3.78E+02	2.75E+02	2.68E+02
F_48_	Min	**2.28E−02**	2.64E−01	3.00E+01	3.14E+01	8.23E+01	7.16E+01	3.09E+01	3.51E+01
	Mean	1.52E−01	3.60E+00	3.00E+01	3.68E+01	9.96E+01	9.92E+01	3.71E+01	4.16E+01
	Std. dev	3.24E−01	2.95E+00	3.05E−02	2.95E+00	8.18E+00	9.83E+00	3.74E+00	3.83E+00
	Max	3.25E+00	1.16E+01	3.01E+01	4.45E+01	1.25E+02	1.20E+02	5.10E+01	5.65E+01
F_49_	Min	− 1.81E+01	− 1.90E+01	− 1.54E+01	− 2.09E+01	− 1.08E+01	− 1.20E+01	− 1.66E+01	**− 2.10E+01**
	Mean	− 1.22E+01	− 1.25E+01	− 1.14E+01	− 1.54E+01	− 8.63E+00	− 9.68E+00	− 1.32E+01	− 1.77E+01
	Std. dev	1.69E+00	2.03E+00	1.39E+00	2.30E+00	6.34E−01	7.36E−01	1.38E+00	1.24E+00
	Max	− 9.78E+00	− 9.13E+00	− 8.43E+00	− 9.74E+00	− 6.99E+00	− 8.48E+00	− 9.46E+00	− 1.44E+01
F_50_	Min	**4.53E−156**	5.37E−87	1.60E+01	1.41E+02	2.75E−01	7.79E+02	8.32E−04	8.09E+02
	Mean	1.65E−121	2.54E−72	1.18E+02	4.22E+02	2.90E+02	1.46E+03	5.46E−03	1.42E+03
	Std. dev	1.65E−120	1.54E−71	5.41E+01	1.70E+02	4.99E+02	4.64E+02	4.22E−03	4.22E+02
	Max	1.65E−119	1.16E−70	2.81E+02	9.74E+02	2.92E+03	2.71E+03	2.26E−02	2.84E+03
F_51_	Min	**1.51E−156**	1.97E−84	6.26E+02	7.15E+02	1.04E+00	1.56E+03	1.48E−02	1.50E+03
	Mean	7.18E−120	1.36E−68	1.33E+03	1.30E+03	1.02E+02	2.12E+03	2.80E−01	2.31E+03
	Std. dev	7.18E−119	1.36E−67	3.38E+02	3.18E+02	1.35E+02	2.67E+02	2.86E−01	3.44E+02
	Max	7.18E−118	1.36E−66	2.39E+03	2.03E+03	7.26E+02	2.66E+03	1.85E+00	3.15E+03
F_52_	Min	**− 3.73E−01**	4.09E−01	1.50E+00	**− 3.73E−01**	1.05E+01	4.09E−01	4.09E−01	4.09E−01
	Mean	4.46E−01	8.56E−01	2.40E+00	1.41E+00	1.45E+01	1.56E+00	1.14E+00	2.94E+00
	Std. dev	4.20E−01	6.02E−01	9.76E−01	7.07E−01	2.08E+00	7.89E−01	6.67E−01	1.22E+00
	Max	1.50E+00	2.91E+00	4.63E+00	2.91E+00	1.96E+01	4.63E+00	2.91E+00	6.67E+00
F_53_	Min	**− 9.98E−01**	− 9.94E−01	− 9.34E−01	− 1.68E−01	− 2.09E−02	− 2.20E−02	− 4.39E−01	− 4.22E−01
	Mean	− 9.79E−01	− 9.56E−01	− 8.29E−01	− 3.36E−02	− 2.79E−03	− 3.50E−03	− 1.81E−01	− 1.63E−01
	Std. dev	6.43E−02	7.13E−02	5.59E−02	3.12E−02	3.25E−03	3.60E−03	1.04E−01	9.85E−02
	Max	− 4.46E−01	− 6.11E−01	− 6.31E−01	− 8.90E−04	− 1.64E−04	− 2.25E−04	− 2.89E−02	− 1.20E−02
F_54_	Min	**0.00E+00**	**0.00E+00**	2.00E+01	1.40E+01	**0.00E+00**	1.26E+02	**0.00E+00**	1.06E+02
	Mean	0.00E+00	0.00E+00	5.36E+01	5.97E+01	6.80E−01	1.85E+02	0.00E+00	1.85E+02
	Std. dev	0.00E+00	0.00E+00	2.10E+01	2.34E+01	1.21E+00	2.81E+01	0.00E+00	3.35E+01
	Max	0.00E+00	0.00E+00	1.35E+02	1.28E+02	7.00E+00	2.72E+02	0.00E+00	2.63E+02
F_55_	Min	**0.00E+00**	**0.00E+00**	3.10E+01	2.12E+02	1.00E+00	1.30E+03	**0.00E+00**	1.48E+03
	Mean	0.00E+00	0.00E+00	8.22E+01	8.41E+02	5.58E+01	3.12E+03	3.20E−01	3.91E+03
	Std. dev	0.00E+00	0.00E+00	3.25E+01	4.49E+02	7.25E+01	9.07E+02	5.66E−01	1.22E+03
	Max	0.00E+00	0.00E+00	1.99E+02	2.83E+03	3.33E+02	5.59 E+00	3.00E+00	7.33E+03
F_56_	Min	**0.00E+00**	1.75E−02	3.86E−02	2.62E−02	**0.00E+00**	5.10E−02	5.09E−02	5.45E−02
	Mean	5.20E−02	3.05E−02	7.40E−02	4.65E−02	0.00E+00	6.98E−02	7.50E−02	7.42E−02
	Std. dev	3.58E−02	8.16E−03	1.55E−02	9.55E−03	0.00E+00	9.68E−03	1.21E−02	1.08E−02
	Max	1.46E−01	5.83E−02	1.16E−01	7.50E−02	0.00E+00	9.93E−02	1.06E−01	1.02E−01
F_57_	Min	− 2.82E+00	1.79E−01	1.86E−01	1.89E−01	**− 1.56E+16**	2.05E−01	1.64E−01	1.84E−01
	Mean	− 1.49E+00	2.11E−01	2.15E−01	2.17E−01	− 8.42E+14	3.10E−01	2.10E−01	2.14E−01
	Std. dev	5.02E−01	1.11E−02	1.28E−02	1.24E−02	2.09E+15	3.66E−02	1.23E−02	1.16E−02
	Max	− 4.98E−01	2.32E−01	2.42E−01	2.42E−01	− 1.26E+12	4.12E−01	2.35E−01	2.38E−01
F_58_	Min	− 5.33E+00	1.92E−02	6.58E−02	3.27E−02	**− 1.71E+19**	7.22E−02	5.00E−02	5.27E−02
	Mean	− 3.41E+00	4.38E−02	1.14E−01	5.67E−02	− 1.69E+18	1.21E−01	7.86E−02	7.94E−02
	Std. dev	5.92E−01	1.33E−02	2.17E−02	1.29E−02	3.01E+18	2.11E−02	1.29E−02	1.19E−02
	Max	− 2.13E+00	9.10E−02	1.67E−01	9.54E−02	− 5.82E+15	1.90E−01	1.11E−01	1.07E−01
F_59_	Min	**0.00E+00**	**0.00E+00**	1.84E+01	2.18E+01	1.10E−01	9.51E+01	4.63E−04	1.29E+02
	Mean	0.00E+00	0.00E+00	2.57E+01	7.01E+01	1.01E+01	2.15E+02	2.92E−02	2.62E+02
	Std. dev	0.00E+00	0.00E+00	4.24E+00	2.95E+01	1.05E+01	5.64E+01	1.29E−01	6.91E+01
	Max	0.00E+00	0.00E+00	4.29E+01	1.79E+02	8.12E+01	4.00E+02	1.12E+00	5.18E+02
F_60_	Min	− 7.90E−01	**− 9.61E−01**	− 9.17E−01	− 8.48E−01	− 7.02E−01	− 6.37E−01	− 8.40E−01	− 9.41E−01
	Mean	− 6.07E−01	− 6.97E−01	− 8.26E−01	− 7.02E−01	− 5.61E−01	− 5.57E−01	− 7.17E−01	− 8.41E−01
	Std. dev	4.58E−02	1.07E−01	4.04E−02	6.70E−02	3.00E−02	2.42E−02	5.55E−02	3.56E−02
	Max	− 5.35E−01	− 5.37E−01	− 7.19E−01	− 5.54E−01	− 5.06E−01	− 5.12E−01	− 6.03E−01	− 7.47E−01
F_61_	Min	5.25E−11	**2.46E−14**	1.64E+00	1.52E+00	2.82E+00	2.19E+00	2.11E+00	1.99E−01
	Mean	3.55E+00	3.66E+00	2.89E+00	3.16E+00	4.23E+00	4.07E+00	4.09E+00	7.50E−01
	Std. dev	6.55E−01	9.28E−01	5.26E−01	7.93E−01	5.72E−01	5.35E−01	5.78E−01	3.56E−01
	Max	4.51E+00	5.03E+00	3.90E+00	4.89E+00	5.31E+00	5.20E+00	5.16E+00	2.64E+00
F_62_	Min	2.16E−50	**4.50E−51**	3.24E+01	1.67E+02	1.08E−01	9.01E+01	1.58E+00	1.40E+02
	Mean	6.85E−36	2.60E−35	6.49E+01	4.89E+02	5.40E+01	1.68E+02	8.91E+00	3.24E+02
	Std. dev	3.88E−35	2.58E−34	1.82E+01	2.54E+02	4.28E+01	3.37E+01	6.51E+00	1.22E+02
	Max	3.64E−34	2.58E−33	1.16E+02	1.77E+03	1.81E+02	3.00E+02	4.72E+01	8.68E+02
F_63_	Min	**− 8.65E−01**	8.43E+00	6.08E+02	2.28E+03	1.47E+02	3.16E+04	1.50E+01	4.43E+04
	Mean	1.85E+01	2.52E+01	1.68E+03	1.97E+04	1.70E+03	7.38E+04	3.86E+01	9.89E+04
	Std. dev	1.15E+01	1.01E+01	5.21E+02	1.22E+04	2.60E+03	2.27E+04	1.19E+01	3.25E+04
	Max	5.70E+01	5.32E+01	3.26E+03	8.42E+04	1.70E+04	1.77E+05	8.03E+01	2.46E+05
F_64_	Min	**1.74E+03**	**1.74E+03**	1.75E+03	**1.74E+03**	**1.74E+03**	1.75E+03	**1.74E+03**	1.75E+03
	Mean	1.74E+03	1.74E+03	1.76E+03	1.75E+03	1.74E+03	1.76E+03	1.74E+03	1.75E+03
	Std. dev	0.00E+00	0.00E+00	3.55E+00	2.04E+00	2.99E−01	1.97E+00	7.11E−02	2.05E+00
	Max	1.74E+03	1.74E+03	1.77E+03	1.75E+03	1.74E+03	1.76E+03	1.74E+03	1.76E+03
F_65_	Min	**3.72E+02**	3.92E+02	7.70E+02	1.86E+04	7.40E+02	1.16E+05	3.97E+02	3.59E+05
	Mean	4.05E+02	4.06E+02	9.53E+02	1.18E+06	1.33E+09	2.59E+06	4.41E+02	1.57 E+07
	Std. dev	4.26E+00	1.43E+00	4.42E+02	1.72E+06	3.06E+09	2.81 E+06	3.41E+01	1.65 E+07
	Max	4.06E+02	4.07E+02	5.22E+03	9.68E+06	1.87E+10	2.25 E+07	5.64E+02	9.29 E+07
F_66_	Min	**2.84E−02**	4.32E−02	7.71E+02	6.39E+04	6.97E+01	1.41 E+06	2.62E−01	2.92 E+06
	Mean	4.57E−02	7.82E−02	6.05E+03	1.70E+06	5.78E+06	8.04 E+06	9.10E−01	1.43 E+07
	Std. dev	1.23E−02	2.75E−02	6.09E+03	1.52E+06	1.61E+07	4.45 E+06	5.41E−01	8.62 E+06
	Max	9.32E−02	1.99E−01	3.06E+04	7.84E+06	1.09E+08	2.11E+07	3.50E+00	4.12 E+07
F_67_	Min	− 2.69E+02	− 2.65E+02	− 2.57E+02	− 1.97E+02	**− 2.03E+21**	− 1.51E+02	− 2.47E+02	− 2.22E+02
	Mean	− 2.34E+02	− 2.28E+02	− 2.17E+02	− 1.68E+02	− 1.75E+20	− 1.22E+02	− 2.24E+02	− 2.01E+02
	Std. dev	1.45E+01	1.23E+01	1.82E+01	1.31E+01	3.44E+20	9.35E+00	1.04E+01	1.18E+01
	Max	− 1.92E+02	− 1.95E+02	− 1.69E+02	− 1.37E+02	− 7.60E+16	− 1.01E+02	− 1.88E+02	− 1.46E+02
F_68_	Min	**3.13E+01**	3.19E+01	3.08E+02	4.12E+04	8.10E+01	7.43 E+05	4.07E+01	1.67 E+06
	Mean	3.16E+01	3.46E+01	1.69E+03	7.03E+05	1.62E+06	4.97E+06	5.13E+01	8.75E+06
	Std. dev	3.75E−01	1.58E+00	1.30E+03	6.67E+05	7.50E+06	2.78E+06	4.74E+00	4.12E+06
	Max	3.29E+01	3.82E+01	7.67E+03	4.64E+06	7.29E+07	1.81E+07	6.44E+01	2.18E+07
F_69_	Min	1.23E+01	**1.16E+01**	1.17E+02	4.71E+04	1.61E+01	9.82E+05	1.21E+01	9.29 E+05
	Mean	1.32E+01	1.19E+01	2.03E+03	7.09E+05	1.44E+06	4.45E+06	1.28E+01	4.74E+06
	Std. dev	3.05E−01	1.77E−01	1.56E+03	8.06E+05	6.25E+06	2.11E+06	3.04E−01	3.01E+06
	Max	1.40E+01	1.24E+01	8.52E+03	5.79E+06	5.93E+07	1.19E+07	1.35E+01	1.87E+07

**Table 7:** Wilcoxon sum rank test analysis results for multimodal test functions.

	HMRFO	MRFO	MVO	PSO	SINECOS	JAYA	EQUIL	MOTH
F_1_	N/A	1.00	< 0.05	< 0.05	< 0.05	< 0.05	< 0.05	< 0.05
	1	1(=)	5(+)	6(+)	4(+)	7(+)	3(+)	8(+)
F_2_	N/A	1.00	< 0.05	< 0.05	< 0.05	< 0.05	< 0.05	< 0.05
	1	1(=)	4(+)	6(+)	5(+)	8(+)	3(+)	7(+)
F_3_	N/A	1.00	< 0.05	< 0.05	< 0.05	< 0.05	< 0.05	< 0.05
	1	1(=)	6(+)	5(+)	4(+)	8(+)	3(+)	7(+)
F_4_	N/A	< 0.05	< 0.05	< 0.05	< 0.05	< 0.05	< 0.05	< 0.05
	1	2(+)	4(+)	6(+)	5(+)	7(+)	3(+)	8(+)
F_5_	N/A	< 0.05	< 0.05	< 0.05	< 0.05	< 0.05	< 0.05	< 0.05
	1	2(+)	7(+)	5(+)	4(+)	8(+)	3(+)	6(+)
F_6_	N/A	< 0.05	< 0.05	< 0.05	< 0.05	< 0.05	< 0.05	< 0.05
	1	2(+)	6(+)	4(+)	7(+)	8(+)	3(+)	5(+)
F_7_	N/A	< 0.05	< 0.05	< 0.05	< 0.05	< 0.05	< 0.05	< 0.05
	1	2(+)	4(+)	5(+)	8(+)	6(+)	3(+)	7(+)
F_8_	N/A	1.00	< 0.05	< 0.05	< 0.05	< 0.05	< 0.05	< 0.05
	1	1(=)	3(+)	6(+)	7(+)	8(+)	5(+)	4(+)
F_9_	N/A	< 0.05	< 0.05	< 0.05	< 0.05	< 0.05	< 0.05	< 0.05
	1	2(+)	5(+)	6(+)	4(+)	7(+)	3(+)	8(+)
F_10_	N/A	< 0.05	< 0.05	< 0.05	< 0.05	< 0.05	< 0.05	< 0.05
	1	2(+)	5(+)	6(+)	4(+)	7(+)	3(+)	8(+)
F_11_	N/A	1.00	< 0.05	< 0.05	< 0.05	< 0.05	< 0.05	< 0.05
	1	1(=)	7(+)	6(+)	4(+)	8(+)	3(+)	5(+)
F_12_	N/A	< 0.05	< 0.05	< 0.05	< 0.05	< 0.05	< 0.05	< 0.05
	1	2(+)	6(+)	5(+)	4(+)	7(+)	3(+)	8(+)
F_13_	N/A	< 0.05	0.321	< 0.05	< 0.05	< 0.05	< 0.05	< 0.05
	5	2(−)	4(−)	6(+)	7(+)	8(+)	3(−)	1(−)
F_14_	N/A	1.00	< 0.05	< 0.05	< 0.05	< 0.05	< 0.05	< 0.05
	1	1(=)	3(+)	4(+)	8(+)	7(+)	4(+)	6(+)
F_15_	N/A	< 0.05	< 0.05	< 0.05	< 0.05	< 0.05	< 0.05	< 0.05
	1	2(+)	4(+)	6(+)	7(+)	8(+)	5(+)	3(+)
F_16_	N/A	< 0.05	< 0.05	< 0.05	< 0.05	< 0.05	< 0.05	< 0.05
	1	2(+)	4(+)	5(+)	8(+)	6(+)	3(+)	7(+)
F_17_	N/A	< 0.05	< 0.05	< 0.05	< 0.05	< 0.05	< 0.05	< 0.05
	1	2(+)	8(+)	4(+)	5(+)	6(+)	3(+)	7(+)
F_18_	N/A	< 0.05	< 0.05	< 0.05	< 0.05	< 0.05	< 0.05	< 0.05
	6	2(−)	1(−)	3(−)	8(+)	7(+)	4(−)	5(−)
F_19_	N/A	< 0.05	< 0.05	< 0.05	< 0.05	< 0.05	< 0.05	< 0.05
	1	2(+)	3(+)	5(+)	8(+)	6(+)	4(+)	7(+)
F_20_	N/A	< 0.05	< 0.05	< 0.05	< 0.05	< 0.05	< 0.05	< 0.05
	1	2(+)	3(+)	5(+)	7(+)	6(+)	4(+)	8(+)
F_21_	N/A	< 0.05	< 0.05	< 0.05	< 0.05	< 0.05	< 0.05	< 0.05
	1	2(+)	6(+)	5(+)	4(+)	7(+)	3(+)	8(+)
F_22_	N/A	< 0.05	< 0.05	< 0.05	< 0.05	< 0.05	< 0.05	< 0.05
	1	2(+)	4(+)	6(+)	5(+)	8(+)	3(+)	7(+)
F_23_	N/A	0.775	< 0.05	< 0.05	< 0.05	< 0.05	< 0.05	< 0.05
	5	6(+)	2(−)	4(−)	8(+)	7(+)	3(−)	1(−)
F_24_	N/A	< 0.05	< 0.05	< 0.05	< 0.05	< 0.05	< 0.05	< 0.05
	1	2(+)	7(+)	4(+)	8(+)	5(+)	3(+)	6(+)
F_25_	N/A	0.754	< 0.05	< 0.05	< 0.05	< 0.05	< 0.05	< 0.05
	5	6(+)	2(−)	4(−)	8(+)	7(+)	3(−)	1(−)
F_26_	N/A	< 0.05	< 0.05	< 0.05	< 0.05	< 0.05	< 0.05	< 0.05
	1	3(+)	2(+)	6(+)	5(+)	7(+)	4(+)	8(+)
F_27_	N/A	< 0.05	< 0.05	< 0.05	< 0.05	< 0.05	< 0.05	< 0.05
	1	2(+)	4(+)	5(+)	8(+)	6(+)	3(+)	7(+)
F_28_	N/A	< 0.05	< 0.05	< 0.05	< 0.05	< 0.05	< 0.05	< 0.05
	2	1(−)	4(+)	5(+)	8(+)	6(+)	3(+)	7(+)
F_29_	N/A	0.06	< 0.05	< 0.05	< 0.05	< 0.05	0.236	0.891
	2	5(+)	1(−)	6(+)	8(+)	7(+)	4(+)	3(+)
F_30_	N/A	< 0.05	< 0.05	< 0.05	< 0.05	< 0.05	< 0.05	< 0.05
	3	1(−)	7(+)	4(+)	6(+)	8(+)	2(−)	5(+)
F_31_	N/A	< 0.05	< 0.05	< 0.05	< 0.05	< 0.05	0.672	< 0.05
	3	4(+)	7(+)	5(+)	1(−)	8(+)	2(−)	6(+)
F_32_	N/A	< 0.05	< 0.05	< 0.05	< 0.05	< 0.05	< 0.05	< 0.05
	6	4(−)	2(−)	5(−)	7(+)	8(+)	3(−)	1(−)
F_33_	N/A	< 0.365	< 0.05	< 0.901	< 0.05	< 0.05	< 0.05	< 0.05
	4	3(−)	6(+)	5(+)	7(+)	8(+)	1(−)	2(−)
F_34_	N/A	1.00	< 0.05	< 0.05	< 0.05	< 0.05	< 0.05	< 0.05
	1	1(=)	4(+)	6(+)	8(+)	7(+)	5(+)	3(+)
F_35_	N/A	< 0.05	< 0.05	0.762	< 0.05	< 0.05	< 0.05	< 0.05
	6	3(−)	1(−)	5(−)	8(+)	7(+)	4(−)	2(−)
F_36_	N/A	< 0.05	0.185	< 0.05	< 0.05	< 0.05	< 0.05	0.183
	6	3(−)	5(−)	2(−)	8(+)	7(+)	1(−)	4(−)
F_37_	N/A	1.00	< 0.05	< 0.05	< 0.05	< 0.05	< 0.05	< 0.05
	1	2(+)	4(+)	6(+)	7(+)	8(+)	3(+)	5(+)
F_38_	N/A	0.679	< 0.05	< 0.05	< 0.05	< 0.05	< 0.05	< 0.06
	2	3(+)	7(+)	5(+)	6(+)	8(+)	4(+)	1(−)
F_39_	N/A	0.294	< 0.05	< 0.05	< 0.05	< 0.05	< 0.05	< 0.05
	3	4(+)	5(+)	6(+)	7(+)	8(+)	1(−)	2(−)
F_40_	N/A	1.00	< 0.05	< 0.05	< 0.05	< 0.05	< 0.05	< 0.05
	1	1(=)	7(+)	6(+)	4(+)	8(+)	3(+)	5(+)
F_41_	N/A	< 0.05	< 0.05	< 0.05	< 0.05	< 0.05	< 0.05	< 0.05
	1	3(+)	5(+)	8(+)	2(+)	5(+)	4(+)	7(+)
F_42_	N/A	< 0.05	< 0.05	< 0.05	0.483	< 0.05	< 0.05	< 0.05
	2	3(+)	7(+)	8(+)	1(−)	5(+)	4(+)	6(+)
F_43_	N/A	< 0.05	< 0.05	< 0.05	< 0.05	< 0.05	< 0.05	< 0.05
	1	4(+)	3(+)	5(+)	2(+)	6(+)	7(+)	8(+)
F_44_	N/A	< 0.05	< 0.05	< 0.05	< 0.05	< 0.05	< 0.05	< 0.05
	1	2(+)	3(+)	6(+)	8(+)	7(+)	4(+)	5(+)
F_45_	N/A	< 0.05	< 0.05	< 0.05	< 0.05	< 0.05	< 0.05	< 0.05
	1	2(+)	5(+)	3(+)	8(+)	7(+)	4(+)	6(+)
F_46_	N/A	0.167	< 0.05	< 0.05	< 0.05	< 0.05	< 0.05	< 0.05
	1	2(+)	4(+)	6(+)	7(+)	8(+)	3(+)	5(+)
F_47_	N/A	< 0.05	< 0.05	< 0.05	< 0.05	< 0.05	< 0.05	< 0.05
	1	3(+)	2(+)	6(+)	8(+)	7(+)	5(+)	4(+)
F_48_	N/A	< 0.05	< 0.05	< 0.05	< 0.05	< 0.05	< 0.05	< 0.05
	1	2(+)	3(+)	4(+)	8(+)	7(+)	5(+)	6(+)
F_49_	N/A	0.198	< 0.05	< 0.05	< 0.05	< 0.05	< 0.05	< 0.05
	5	4(+)	6(+)	2(+)	8(+)	7(+)	3(+)	1(−)
F_50_	N/A	< 0.05	< 0.05	< 0.05	< 0.05	< 0.05	< 0.05	< 0.05
	1	2(+)	4(+)	6(+)	5(+)	8(+)	3(+)	7(+)
F_51_	N/A	< 0.05	< 0.05	< 0.05	< 0.05	< 0.05	< 0.05	< 0.05
	1	2(+)	6(+)	5(+)	4(+)	7(+)	3(+)	8(+)
F_52_	N/A	< 0.05	< 0.05	< 0.05	< 0.05	< 0.05	< 0.05	< 0.05
	1	2(+)	6(+)	4(+)	8(+)	5(+)	3(+)	7(+)
F_53_	N/A	< 0.05	< 0.05	< 0.05	< 0.05	< 0.05	< 0.05	< 0.05
	1	2(+)	3(+)	6(+)	8(+)	7(+)	4(+)	5(+)
F_54_	N/A	1.00	< 0.05	< 0.05	< 0.05	< 0.05	1.00	< 0.05
	1	1(=)	5(+)	6(+)	4(+)	7(+)	1(=)	8(+)
F_55_	N/A	1.00	< 0.05	< 0.05	< 0.05	< 0.05	< 0.05	< 0.05
	1	1(=)	5(+)	6(+)	4(+)	7(+)	3(+)	8(+)
F_56_	N/A	< 0.05	< 0.05	0.362	< 0.05	< 0.05	< 0.05	< 0.05
	4	2(−)	6(−)	3(−)	1(−)	5(+)	8(+)	7(+)
F_57_	N/A	< 0.05	< 0.05	< 0.05	< 0.05	< 0.05	< 0.05	< 0.05
	2	4(+)	6(+)	7(+)	1(−)	8(+)	3(+)	5(+)
F_58_	N/A	< 0.05	< 0.05	< 0.05	< 0.05	< 0.05	< 0.05	< 0.05
	2	3(+)	7(+)	4(+)	1(−)	8(+)	5(+)	6(+)
F_59_	N/A	1.00	< 0.05	< 0.05	< 0.05	< 0.05	< 0.05	< 0.05
	1	1(=)	5(+)	6(+)	4(+)	7(+)	3(+)	8(+)
F_60_	N/A	< 0.05	< 0.05	< 0.05	< 0.05	< 0.05	< 0.05	< 0.05
	6	5(−)	2(−)	4(−)	7(+)	8(+)	3(−)	1(−)
F_61_	N/A	< 0.05	< 0.05	< 0.05	< 0.05	< 0.05	< 0.05	< 0.05
	4	5(+)	2(−)	3(−)	8(+)	6(+)	7(+)	1(−)
F_62_	N/A	< 0.05	< 0.05	< 0.05	< 0.05	< 0.05	< 0.05	< 0.05
	1	2(+)	5(+)	8(+)	4(+)	6(+)	3(+)	7(+)
F_63_	N/A	< 0.05	< 0.05	< 0.05	< 0.05	< 0.05	< 0.05	< 0.05
	1	2(+)	4(+)	6(+)	5(+)	7(+)	3(+)	8(+)
F_64_	N/A	1.00	< 0.05	< 0.05	< 0.05	< 0.05	< 0.05	< 0.05
	1	1(=)	8(+)	5(+)	4(+)	7(+)	3(+)	6(+)
F_65_	N/A	< 0.05	< 0.05	< 0.05	< 0.05	< 0.05	< 0.05	< 0.05
	1	2(+)	4(+)	5(+)	8(+)	6(+)	3(+)	7(+)
F_66_	N/A	< 0.05	< 0.05	< 0.05	< 0.05	< 0.05	< 0.05	< 0.05
	1	2(+)	4(+)	5(+)	6(+)	7(+)	3(+)	8(+)
F_67_	N/A	< 0.05	< 0.05	< 0.05	< 0.05	< 0.05	< 0.05	< 0.05
	2	3(+)	5(+)	7(+)	1(−)	8(+)	4(+)	6(+)
F_68_	N/A	< 0.05	< 0.05	< 0.05	< 0.05	< 0.05	< 0.05	< 0.05
	1	2(+)	4(+)	5(+)	6(+)	7(+)	3(+)	8(+)
F_69_	N/A	< 0.05	< 0.05	< 0.05	< 0.05	< 0.05	< 0.05	< 0.05
	3	1(−)	4(+)	5(+)	6(+)	7(+)	2(−)	8(+)
Aver. rank	1.94	2.34	4.52	5.18	5.73	7.02	3.40	5.59
Ranking	1	2	4	5	7	8	3	6
+/=/−	//	48/11/10	58/0/11	60/0/9	63/0/6	69/0/0	57/1/11	59/0/10

**Table 8:** Predictive results obtained for the compared algorithms for 30D unimodal test problems.

		HMRFO	MRFO	MVO	PSO	SINECOS	JAYA	EQUIL	MOTH
F_70_	Min	**1.49E−156**	5.88E−90	2.51E−01	1.05E+00	2.32E−03	1.17E+01	8.66E−06	1.90E+01
	Mean	4.26E−119	5.60E−74	5.90E−01	7.68E+00	5.06E−01	3.22E+01	7.28E−05	3.86E+01
	Std. dev	4.26E−118	3.71E−73	2.02E−01	4.71E+00	9.44E−01	1.02E+01	5.85E−05	1.22E+01
	Max	4.26E−117	3.56E−72	1.26E+00	2.89E+01	6.94E+00	6.09E+01	3.76E−04	7.41E+01
F_71_	Min	**2.54E+01**	2.61E+01	8.38E+01	7.76E+02	3.01E+01	4.42E+03	2.75E+01	7.32E+03
	Mean	2.63E+01	2.71E+01	3.08E+02	5.81E+03	3.38E+04	1.90E+04	2.85E+01	3.57E+04
	Std. dev	3.73E−01	5.59E−01	2.31E+02	4.47E+03	1.55E+05	1.09E+04	3.80E−01	2.43E+04
	Max	2.78E+01	2.87E+01	1.71E+03	2.67E+04	1.52E+06	6.54E+04	2.97E+01	1.85E+05
F_72_	Min	**2.29E−171**	3.94E−89	7.57E+00	7.44E+04	4.72E+00	4.48E+08	5.00E−05	1.47E+11
	Mean	1.13E−123	2.36E−73	1.71E+28	9.35E+41	2.18E+176	1.69E+42	2.12E−03	1.54E+74
	Std. dev	1.12E−122	1.38E−72	1.70E+29	9.35E+42	2.75E+177	1.69E+43	2.76E−03	1.53E+75
	Max	1.12E−121	1.24E−71	1.70E+30	9.35E+43	8.29E+178	1.69E+44	2.37E−02	1.54E+76
F_73_	Min	**2.39E−40**	1.12E−20	8.20E+00	1.05E+01	1.07E+01	1.86E+01	1.42E+00	8.74E+00
	Mean	5.83E−31	2.24E−04	1.35E+01	1.72E+01	1.83E+01	2.25E+01	4.13E+00	1.17E+01
	Std. dev	4.87E−30	7.33E−04	2.06E+00	3.00E+00	2.71E+00	1.65E+00	1.45E+00	1.61E+00
	Max	4.86E−29	2.95E−03	1.92E+01	2.33E+01	2.49E+01	2.63E+01	9.59E+00	1.57E+01
F_74_	Min	**3.02E−228**	4.65E−120	1.78E−01	7.48E+02	2.55E−03	3.25E+05	2.53E−18	1.22E+06
	Mean	3.88E−188	1.60E−100	3.68E+04	1.03E+07	6.10E+08	3.24E+09	2.25E−11	4.26E+12
	Std. dev	4.91E−187	1.55E−99	1.14E+05	4.66E+07	3.53E+09	1.78E+10	8.55E−11	4.01E+13
	Max	7.85E−186	1.55E−98	7.52E+05	3.44E+08	2.94E+10	1.73E+11	5.48E−10	4.01E+14
F_75_	Min	**1.79E−227**	1.31E−115	1.34E+01	3.02E+02	4.50E−02	2.53E+05	7.12E−18	2.95E+05
	Mean	2.23E−193	2.03E−101	6.24E+04	2.81E+07	1.53E+08	3.35E+09	5.05E−10	1.07E+11
	Std. dev	0.00E+00	9.76E−101	3.15E+05	1.43E+08	7.57E+08	2.19E+10	4.52E−09	6.01E+11
	Max	1.98E−191	8.51E−100	2.93E+06	1.32E+09	6.05E+09	2.15E+11	4.52E−08	5.44E+12
F_76_	Min	**1.44E−166**	1.55E−89	6.53E+00	2.22E+01	1.44E−02	1.65E+02	9.60E−05	1.82E+02
	Mean	5.14E−118	8.13E−72	2.96E+01	8.70E+01	5.27E+00	3.67E+02	8.72E−04	4.34E+02
	Std. dev	5.09E−117	6.18E−71	1.61E+01	4.37E+01	8.93E+00	1.00E+02	6.46E−04	1.27E+02
	Max	5.09E−116	6.12E−70	9.37E+01	2.32E+02	5.96E+01	6.76E+02	3.36E−03	7.64E+02
F_77_	Min	**2.42E−162**	1.89E−80	2.31E+05	5.23E+05	4.48E+03	1.30E+07	5.45E+00	1.46E+07
	Mean	1.81E−117	2.49E−65	5.14E+05	5.61E+06	4.94E+05	2.61E+07	6.32E+01	3.64E+07
	Std. dev	1.80E−116	2.48E−64	1.63E+05	3.65E+06	1.36E+06	7.98 E+06	5.62E+01	1.11E+07
	Max	1.80E−115	2.48E−63	1.07E+06	1.80E+07	1.29E+07	5.70 E+07	3.24E+02	6.91E+07
F_78_	Min	**6.45E−161**	2.30E−87	3.98E+01	3.98E+00	1.47E−03	2.30E+01	3.27E−05	2.17E+01
	Mean	4.34E−119	3.25E−69	1.26E+02	1.60E+01	5.83E−01	4.90E+01	2.18E−04	5.11E+01
	Std. dev	3.17E−118	3.16E−68	4.64E+01	8.57E+00	1.11E+00	1.33E+01	1.72E−04	1.47E+01
	Max	2.78E−117	3.16E−67	3.01E+02	4.54E+01	8.80E+00	9.52E+01	9.80E−04	9.12E+01
F_79_	Min	**9.68E−84**	3.73E−44	2.86E+00	2.75E+00	1.75E−02	1.34E+01	5.50E−03	9.71E+00
	Mean	5.43E−61	6.33E−37	6.27E+00	7.58E+00	1.95E−01	1.99E+01	1.65E−02	1.91E+01
	Std. dev	4.36E−60	3.83E−36	2.05E+00	2.25E+00	1.63E−01	3.16E+00	6.72E−03	3.36E+00
	Max	4.30E−59	3.26E−35	1.17E+01	1.60E+01	8.47E−01	2.95E+01	4.16E−02	2.77E+01
F_80_	Min	**7.10E−83**	1.09E−45	1.46E+00	2.81E+00	2.03E+00	2.52E+00	1.31E−02	3.71E+00
	Mean	1.83E−61	1.58E−37	2.59E+00	4.08E+00	7.45E+00	3.91E+00	6.17E−02	5.15E+00
	Std. dev	1.72E−60	1.03E−36	7.26E−01	5.09E−01	1.71E+00	5.16E−01	3.06E−02	5.61E−01
	Max	1.72E−59	1.01E−35	5.16E+00	5.39E+00	9.42E+00	5.24E+00	2.13E−01	6.50E+00
F_81_	Min	**0.00E+00**	**0.00E+00**	3.21E−04	1.49E+03	1.32E−03	1.08E+04	5.22E−21	3.73E+04
	Mean	0.00E+00	0.00E+00	4.27E+00	3.08E+05	1.15E+09	8.56E+05	4.57E−13	3.37E+06
	Std. dev	0.00E+00	0.00E+00	1.15E+01	4.96E+05	2.64E+09	1.18E+06	3.91E−12	4.33E+06
	Max	0.00E+00	0.00E+00	6.90E+01	3.12E+06	1.43E+10	6.60E+06	3.90E−11	2.40E+07
F_82_	Min	**9.18E−02**	1.51E+00	3.98E+00	4.36E+01	2.52E+01	9.60E+01	7.64E+00	1.36E+02
	Mean	1.80E+00	5.16E+00	2.54E+01	2.59E+02	4.05E+02	3.13E+02	1.12E+01	5.24E+02
	Std. dev	1.38E+00	1.85E+00	3.04E+01	1.25E+02	8.84E+02	1.51E+02	1.72E+00	2.00E+02
	Max	6.64E+00	9.48E+00	2.20E+02	7.64E+02	4.86E+03	8.81E+02	1.46E+01	1.14E+03
F_83_	Min	**0.00E+00**	**0.00E+00**	3.28E−04	1.49E+03	1.32E−03	1.08E+04	5.22E−21	3.73E+04
	Mean	0.00E+00	0.00E+00	4.27E+00	3.08E+05	1.15E+09	8.56E+05	4.57E−13	3.37E+06
	Std. dev	0.00E+00	0.00E+00	1.15E+01	4.96E+05	2.64E+09	1.18E+06	3.91E−12	4.33E+06
	Max	0.00E+00	0.00E+00	6.90E+01	3.12E+06	1.43E+10	6.60E+06	3.90E−11	2.40E+07
F_84_	Min	**− 9.33E+02**	− 9.17E+02	− 6.29E+02	2.06E+04	9.86E+01	6.46E+04	− 3.47E+02	9.64E+04
	Mean	− 3.52E+02	− 2.49E+02	2.96E+04	1.07E+05	2.00E+04	1.56E+05	− 3.55E+01	2.77E+05
	Std. dev	1.48E+02	1.19E+02	1.87E+04	4.73E+04	4.06E+04	6.41E+04	5.87E+01	8.57E+04
	Max	− 1.64E+02	− 8.98E+01	9.46E+04	2.47E+05	2.55E+05	3.46E+05	4.80E+01	5.44E+05
F_85_	Min	**2.60E+01**	2.63E+01	1.14E+02	2.01E+03	4.06E+01	2.95E+04	2.76E+01	1.07E+04
	Mean	2.69E+01	2.72E+01	4.32E+02	5.55E+04	8.30E+06	2.63E+05	2.85E+01	5.77E+05
	Std. dev	5.38E−01	4.46E−01	2.32E+02	5.89E+04	2.64E+07	2.24E+05	3.45E−01	5.13E+05
	Max	2.87E+01	2.87E+01	1.46E+03	2.99E+05	1.59E+08	1.19E+06	2.90E+01	2.95E+06
F_86_	Min	**2.79E−01**	3.07E−01	2.82E+00	9.30E+00	1.09E+00	1.47E+01	4.32E−01	2.52E+01
	Mean	3.75E−01	4.72E−01	9.27E+00	2.88E+01	4.75E+00	4.05E+01	7.55E−01	6.83E+01
	Std. dev	5.75E−02	1.16E−01	3.59E+00	1.29E+01	5.00E+00	1.48E+01	2.09E−01	2.07E+01
	Max	6.44E−01	1.14E+00	2.05E+01	7.75E+01	3.06E+01	9.12E+01	1.48E+00	1.20E+02
F_87_	Min	**1.13E−166**	8.43E−90	5.14E−02	5.51E−01	4.44E−04	1.50E+00	7.08E−07	2.64E+00
	Mean	6.13E−126	1.06E−73	5.96E−01	1.82E+00	3.59E−01	4.43E+00	2.34E−05	6.70E+00
	Std. dev	5.20E−125	1.01E−72	2.36E+00	1.00E+00	9.33E−01	1.36E+00	2.17E−05	1.96E+00
	Max	5.11E−124	1.01E−71	1.66E+01	7.90E+00	7.35E+00	8.79E+00	1.09E−04	1.16E+01
F_88_	Min	**8.78E−02**	1.27E−01	1.16E+00	1.10E+00	2.40E+01	1.55E+00	1.40E+00	1.97E+00
	Mean	5.47E−01	5.29E−01	1.89E+00	1.96E+00	4.36E+01	2.20E+00	2.17E+00	3.10E+00
	Std. dev	7.75E−01	2.20E−01	2.92E−01	4.03E−01	8.35E+00	3.70E−01	4.36E−01	6.69E−01
	Max	5.44E+00	1.15E+00	2.65E+00	3.11E+00	6.41E+01	3.75E+00	3.61E+00	5.69E+00
F_89_	Min	**1.92E−02**	5.74E+01	5.59E+01	2.37E+02	8.29E+01	1.14E+02	8.45E+01	1.71E+02
	Mean	3.98E+00	9.64E+01	8.55E+01	3.61E+02	3.43E+02	1.49E+02	1.28E+02	2.39E+02
	Std. dev	8.12E+00	1.68E+01	1.63E+01	6.64E+01	7.97E+01	1.82E+01	1.76E+01	3.58E+01
	Max	5.40E+01	1.50E+02	1.52E+02	5.44E+02	5.85E+02	1.97E+02	1.71E+02	3.54E+02
F_90_	Min	**1.71E−02**	4.34E−02	9.22E−02	1.11E−01	6.62E+00	2.61E−01	1.47E−01	4.05E−01
	Mean	8.42E−02	1.34E−01	2.98E−01	5.87E−01	2.13E+01	6.14E−01	4.35E−01	1.02E+00
	Std. dev	4.52E−02	6.67E−02	1.26E−01	3.71E−01	7.44E+00	2.41E−01	1.75E−01	3.76E−01
	Max	3.07E−01	3.65E−01	6.79E−01	2.18E+00	5.54E+01	1.62E+00	1.04E+00	1.91E+00
F_91_	Min	**7.13E−02**	**7.13E−02**	3.45E−01	3.65E+00	1.30E−01	1.69E+00	7.27E−02	3.10E+00
	Mean	7.13E−02	7.16E−02	8.30E−01	6.47E+00	4.39E−01	2.96E+00	8.02E−02	4.91E+00
	Std. dev	3.88E−05	2.09E−04	3.31E−01	1.19E+00	6.99E−01	5.87E−01	3.92E−03	9.08E−01
	Max	7.15E−02	7.27E−02	2.79E+00	9.25E+00	5.65E+00	5.28E+00	9.54E−02	7.93E+00
F_92_	Min	**5.27E−01**	1.03E+00	8.49E−01	2.74E+00	1.94E+01	4.36E+00	5.16E+00	6.76E+00
	Mean	2.11E+00	3.97E+00	1.70E+00	8.40E+00	3.96E+01	1.05E+01	7.94E+00	2.60E+01
	Std. dev	1.08E+00	1.59E+00	5.39E−01	3.89E+00	7.21E+01	5.87E−01	1.24E+00	8.12E+00
	Max	6.06E+00	8.48E+00	3.63E+00	2.04E+01	7.16E+02	5.28E+00	1.11E+01	5.09E+01
F_93_	Min	**1.35E−159**	3.77E−87	3.39E+02	6.19E+02	2.50E+00	1.41E+03	2.36E−03	1.05E+03
	Mean	6.72E−119	1.14E−69	3.50E+03	2.69E+03	1.92E+02	3.67E+03	3.23E−02	3.46E+03
	Std. dev	6.58E−118	7.37E−69	2.59E+03	1.11E+03	3.04E+02	1.15E+03	2.54E−02	1.24E+03
	Max	6.58E−117	5.83E−68	1.36E+04	6.16E+03	1.56E+03	7.74E+03	1.42E−01	7.27E+03
F_94_	Min	**0.00E+00**	2.54E−169	1.09E+02	5.34E+04	7.53E+01	4.00E+05	5.38E−06	1.09E+06
	Mean	5.99E−245	1.41E−134	9.91E+02	5.05E+05	4.47E+06	2.40E+06	7.87E−04	3.97E+06
	Std. dev	0.00E+00	1.41E−133	8.35E+02	4.00E+05	2.12E+07	1.30E+06	2.13E−03	1.98E+06
	Max	3.67E−243	1.41E−132	4.72E+03	2.11E+06	1.97E+08	7.41E+06	1.53E−02	1.07E+07
F_95_	Min	**2.38E−161**	6.26E−84	4.69E+02	1.01E+03	8.62E−01	1.53E+04	7.04E−03	1.99E+04
	Mean	1.78E−116	2.47E−68	2.89E+03	8.54E+03	4.47E+02	3.61E+04	8.36E−02	4.40E+04
	Std. dev	1.75E−115	2.44E−67	1.51E+03	4.95E+03	7.40E+02	9.58E+03	6.34E−02	1.21E+04
	Max	1.75E−114	2.44E−66	6.87E+03	2.57E+04	4.45E+03	6.54E+04	3.55E−01	8.71E+04
F_96_	Min	− 4.23E+02	− 5.00E+00	− 5.00E+00	1.58E+01	− 4.67E+04	8.14E+02	**− 4.9E+00**	1.88E+03
	Mean	− 5.49E+01	− 4.99E+00	− 4.99E+00	3.99E+02	− 1.00E+03	5.22E+03	− 4.89E+00	8.44E+03
	Std. dev	8.14E+01	1.16E−03	1.16E−03	5.21E+02	5.48E+03	3.03E+03	6.58E−01	5.25E+03
	Max	− 5.47E+00	− 4.99E+00	− 4.99E+00	3.44E+03	9.95E+01	1.57E+04	− 2.80E−01	3.16E+04
F_97_	Min	4.65E−01	3.64E−01	5.47E−01	5.41E−01	9.69E−01	6.26E−01	2.46E−01	**2.21E−01**
	Mean	8.44E−01	8.26E−01	8.33E−01	8.59E−01	1.51E+00	9.50E−01	6.08E−01	5.99E−01
	Std. dev	1.54E−01	1.52E−01	1.66E−01	1.28E−01	2.86E−01	1.14E−01	1.31E−01	1.33E−01
	Max	1.22E+00	1.19E+00	1.28E+00	1.14E+00	2.19E+00	1.15E+00	9.78E−01	9.58E−01
F_98_	Min	**5.83E+03**	1.21E+05	9.01E+05	5.69E+05	1.79E+07	3.41E+06	4.92E+05	5.83E+06
	Mean	9.44E+04	9.25E+05	2.28E+06	2.36E+06	4.07E+07	7.11E+06	2.79E+06	1.43E+07
	Std. dev	1.37E+05	1.08E+06	9.13E+05	1.28E+06	8.88E+06	2.42E+06	2.23E+06	4.67E+06
	Max	1.03E+06	7.31E+06	5.59E+06	7.30E+06	6.74E+07	1.47E+07	1.59E+07	2.75E+07
F_99_	Min	**5.08E−160**	9.11E−89	4.95E−04	1.04E−01	8.53E−02	2.72E−02	2.60E−02	1.70E−02
	Mean	1.34E−121	2.48E−70	1.65E−01	1.52E+00	2.97E+00	3.19E−01	1.91E−01	7.57E−01
	Std. dev	9.55E−121	2.48E−69	2.36E−01	1.83E+00	2.47E+00	4.31E−01	1.13E−01	7.65E−01
	Max	7.92E−120	2.48E−68	1.34E+00	1.35E+01	1.29E+01	1.98E+00	6.89E−01	4.43E+00
F_100_	Min	**2.92E−01**	3.35E−01	2.85E+02	8.37E+02	2.00E+00	1.57E+03	4.03E−01	2.85E+03
	Mean	3.73E−01	4.83E−01	8.48E+02	2.79E+03	6.58E+02	3.94E+03	1.44E+00	6.89E+03
	Std. dev	5.37E−02	9.71E−02	5.05E+02	1.35E+03	1.22E+03	1.32E+03	8.71E−01	2.15E+03
	Max	6.27E−01	7.38E−01	3.77E+03	7.33E+03	6.28E+03	8.38E+03	8.21E+00	1.51E+04

Significant values are in [bold].

**Table 9:** Results of the Wilcoxon sum rank test on 30D unimodal problems at 5% significance level.

	HMRFO	MRFO	MVO	PSO	SINECOS	JAYA	EQUIL	MOTH
F_69_	N/A	< 0.05	< 0.05	< 0.05	< 0.05	< 0.05	< 0.05	< 0.05
	1	2(+)	5(+)	6(+)	4(+)	7(+)	3(+)	8(+)
F_70_	N/A	< 0.05	< 0.05	< 0.05	< 0.05	< 0.05	< 0.05	< 0.05
	1	2(+)	4(+)	5(+)	7(+)	6(+)	3(+)	8(+)
F_71_	N/A	< 0.05	< 0.05	< 0.05	< 0.05	< 0.05	< 0.05	< 0.05
	1	2(+)	4(+)	5(+)	8(+)	6(+)	3(+)	7(+)
F_72_	N/A	< 0.05	< 0.05	< 0.05	< 0.05	< 0.05	< 0.05	< 0.05
	1	2(+)	5(+)	6(+)	7(+)	8(+)	3(+)	4(+)
F_73_	N/A	< 0.05	< 0.05	< 0.05	< 0.05	< 0.05	< 0.05	< 0.05
	1	2(+)	4(+)	5(+)	6(+)	7(+)	3(+)	8(+)
F_74_	N/A	< 0.05	< 0.05	< 0.05	< 0.05	< 0.05	< 0.05	< 0.05
	1	2(+)	4(+)	5(+)	6(+)	7(+)	3(+)	8(+)
F_75_	N/A	< 0.05	< 0.05	< 0.05	< 0.05	< 0.05	< 0.05	< 0.05
	1	2(+)	5(+)	6(+)	4(+)	7(+)	3(+)	8(+)
F_76_	N/A	< 0.05	< 0.05	< 0.05	< 0.05	< 0.05	< 0.05	< 0.05
	1	2(+)	5(+)	6(+)	4(+)	7(+)	3(+)	8(+)
F_77_	N/A	< 0.05	< 0.05	< 0.05	< 0.05	< 0.05	< 0.05	< 0.05
	1	2(+)	8(+)	5(+)	4(+)	6(+)	3(+)	7(+)
F_78_	N/A	< 0.05	< 0.05	< 0.05	< 0.05	< 0.05	< 0.05	< 0.05
	1	2(+)	5(+)	6(+)	4(+)	8(+)	3(+)	7(+)
F_79_	N/A	< 0.05	< 0.05	< 0.05	< 0.05	< 0.05	< 0.05	< 0.05
	1	2(+)	4(+)	6(+)	8(+)	5(+)	3(+)	7(+)
F_80_	N/A	1.00	< 0.05	< 0.05	< 0.05	< 0.05	< 0.05	< 0.05
	1	1(=)	4(+)	5(+)	8(+)	6(+)	3(+)	7(+)
F_81_	N/A	< 0.05	< 0.05	< 0.05	< 0.05	< 0.05	< 0.05	< 0.05
	1	2(+)	4(+)	5(+)	7(+)	6(+)	3(+)	8(+)
F_82_	N/A	1.00	< 0.05	< 0.05	< 0.05	< 0.05	< 0.05	< 0.05
	1	1(=)	5(+)	6(+)	4(+)	7(+)	3(+)	8(+)
F_83_	N/A	< 0.05	< 0.05	< 0.05	< 0.05	< 0.05	< 0.05	< 0.05
	1	2(+)	5(+)	6(+)	4(+)	7(+)	3(+)	8(+)
F_84_	N/A	< 0.05	< 0.05	< 0.05	< 0.05	< 0.05	< 0.05	< 0.05
	1	2(+)	4(+)	5(+)	8(+)	6(+)	3(+)	7(+)
F_85_	N/A	< 0.05	< 0.05	< 0.05	< 0.05	< 0.05	< 0.05	< 0.05
	1	2(+)	5(+)	6(+)	4(+)	7(+)	3(+)	8(+)
F_86_	N/A	< 0.05	< 0.05	< 0.05	< 0.05	< 0.05	< 0.05	< 0.05
	1	2(+)	5(+)	6(+)	4(+)	7(+)	3(+)	8(+)
F_87_	N/A	< 0.05	< 0.05	< 0.05	< 0.05	< 0.05	< 0.05	< 0.05
	2	1(−)	3(+)	4(+)	8(+)	6(+)	5(+)	7(+)
F_88_	N/A	< 0.05	< 0.05	< 0.05	< 0.05	< 0.05	< 0.05	< 0.05
	1	3(+)	2(+)	8(+)	7(+)	5(+)	4(+)	6(+)
F_89_	N/A	< 0.05	< 0.05	< 0.05	< 0.05	< 0.05	< 0.05	< 0.05
	1	2(+)	3(+)	5(+)	8(+)	6(+)	4(+)	7(+)
F_90_	N/A	< 0.05	< 0.05	< 0.05	< 0.05	< 0.05	< 0.05	< 0.05
	1	2(+)	5(+)	8(+)	4(+)	6(+)	3(+)	7(+)
F_91_	N/A	< 0.05	< 0.05	< 0.05	< 0.05	< 0.05	< 0.05	< 0.05
	2	3(+)	1(−)	5(+)	8(+)	6(+)	4(+)	7(+)
F_92_	N/A	< 0.05	< 0.05	< 0.05	< 0.05	< 0.05	< 0.05	< 0.05
	1	2(+)	7(+)	5(+)	4(+)	8(+)	3(+)	6(+)
F_93_	N/A	< 0.05	< 0.05	< 0.05	< 0.05	< 0.05	< 0.05	< 0.05
	1	2(+)	4(+)	5(+)	8(+)	6(+)	3(+)	7(+)
F_94_	N/A	< 0.05	< 0.05	< 0.05	< 0.05	< 0.05	< 0.05	< 0.05
	1	2(+)	4(+)	5(+)	8(+)	6(+)	3(+)	7(+)
F_95_	N/A	< 0.05	< 0.05	< 0.05	< 0.05	< 0.05	< 0.05	< 0.05
	1	2(+)	5(+)	6(+)	4(+)	7(+)	3(+)	8(+)
F_96_	N/A	< 0.05	< 0.05	< 0.05	< 0.05	< 0.05	< 0.05	< 0.05
	2	3(+)	5(+)	6(+)	1(−)	7(+)	4(+)	8(+)
F_97_	N/A	0.312	0.483	0.520	< 0.05	< 0.05	< 0.05	< 0.05
	5	3(−)	4(−)	6(+)	8(+)	7(+)	2(−)	1(−)
F_98_	N/A	< 0.05	< 0.05	< 0.05	< 0.05	< 0.05	< 0.05	< 0.05
	1	2(+)	3(+)	4(+)	8(+)	6(+)	5(+)	7(+)
F_99_	N/A	< 0.05	< 0.05	< 0.05	< 0.05	< 0.05	< 0.05	< 0.05
	1	2(+)	3(+)	7(+)	8(+)	5(+)	4(+)	6(+)
F_100_	N/A	< 0.05	< 0.05	< 0.05	< 0.05	< 0.05	< 0.05	< 0.05
	1	2(+)	5(+)	6(+)	4(+)	7(+)	3(+)	8(+)
Aver. rank	1.21	2.06	4.34	5.62	5.90	6.50	3.25	7.06
Ranking	1	2	4	5	6	8	3	7
+/=/−		27/2/2	29/0/2	31/0/0	30/0/1	31/0/0	30/0/1	30/0/1

### Experiment case—2: performance assessment made on 500D hyperdimensional classical optimization problems

**Fig. 11:**
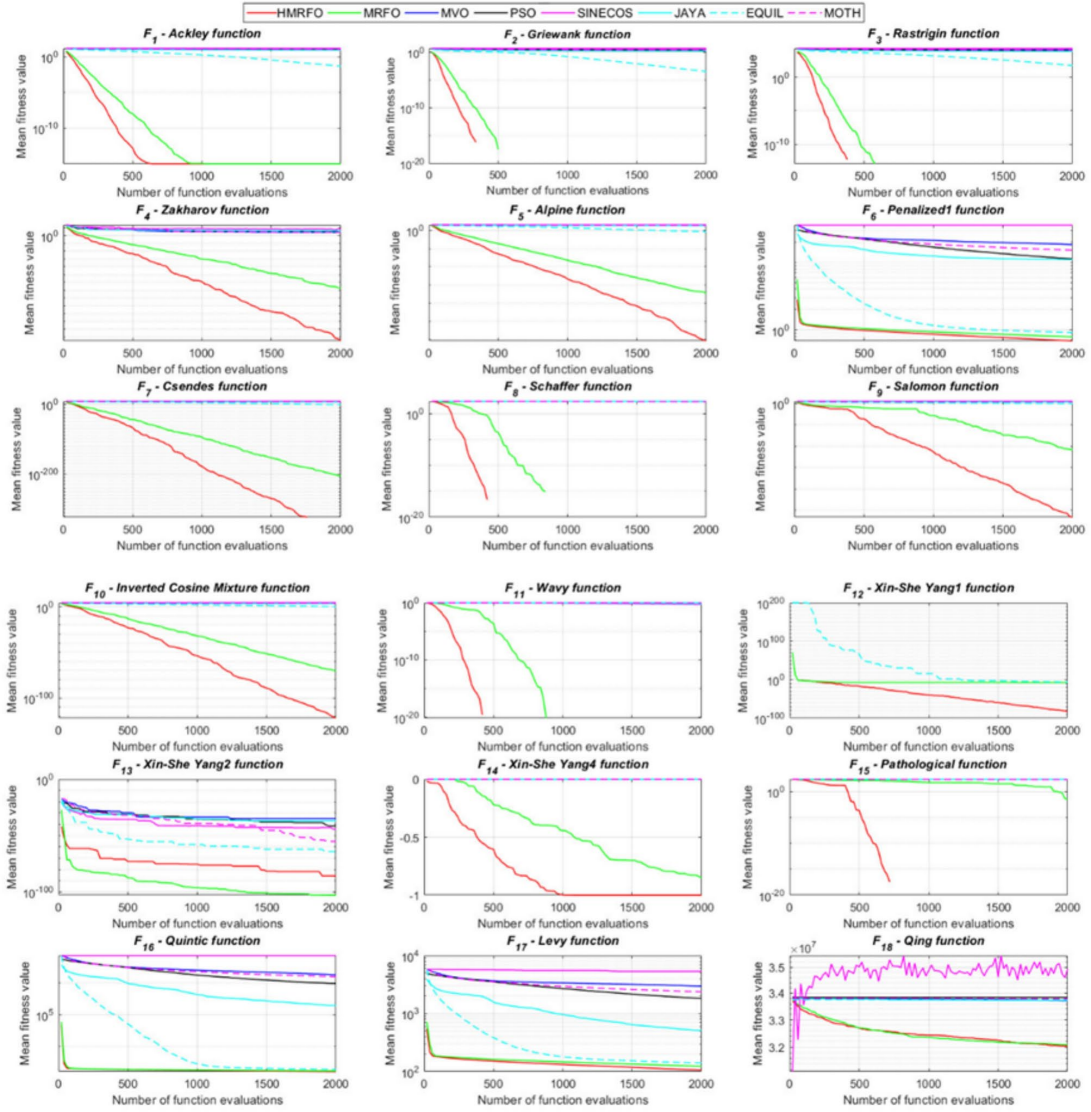
Evolution plots for 500D multimodal test functions from F_1_ to F_18_.

**Fig. 12:**
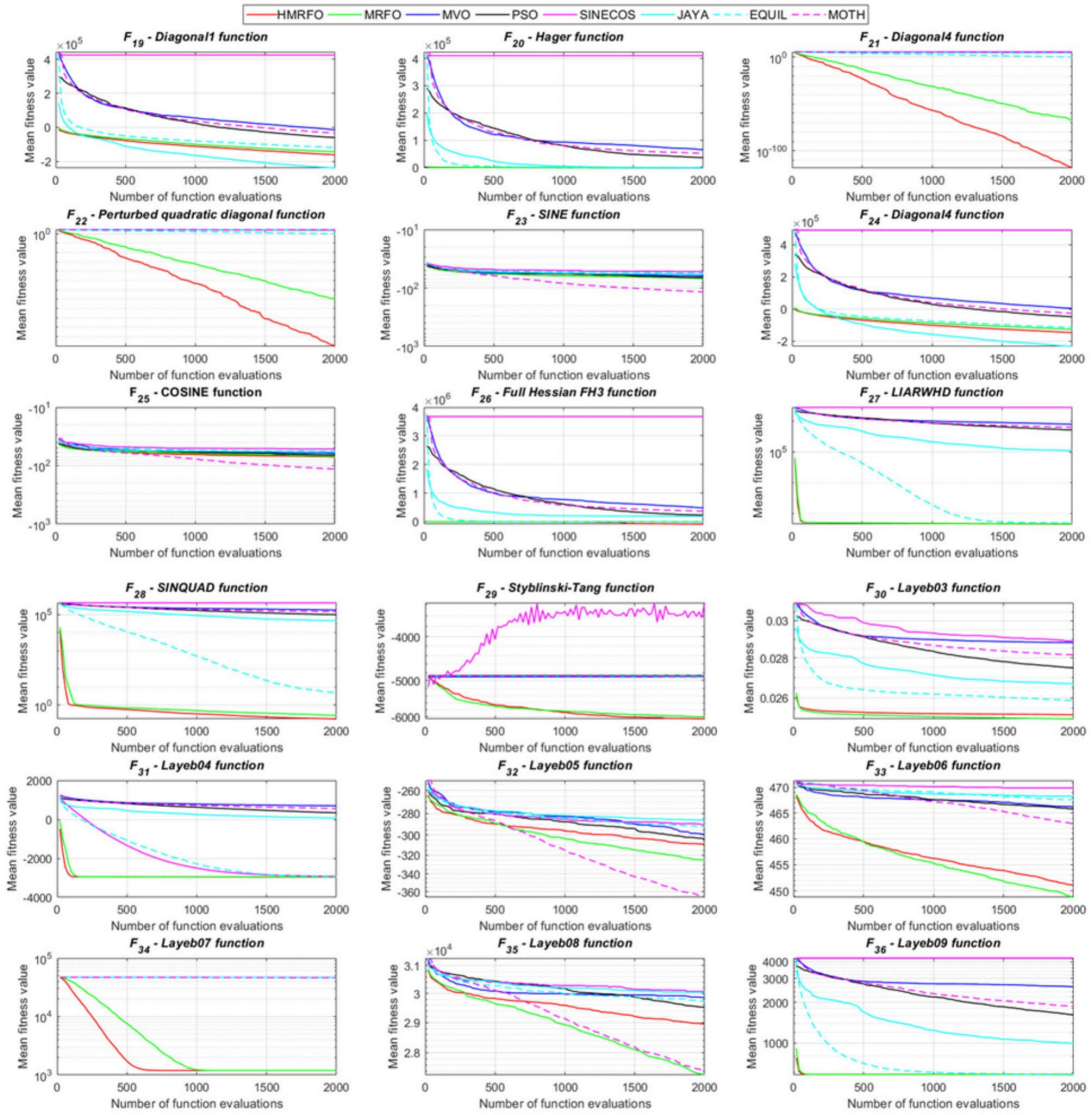
Evolution of the fitness values with the increasing number of iterations for 500D multimodal test problems from F_19_ to F_36_.

**Fig. 13:**
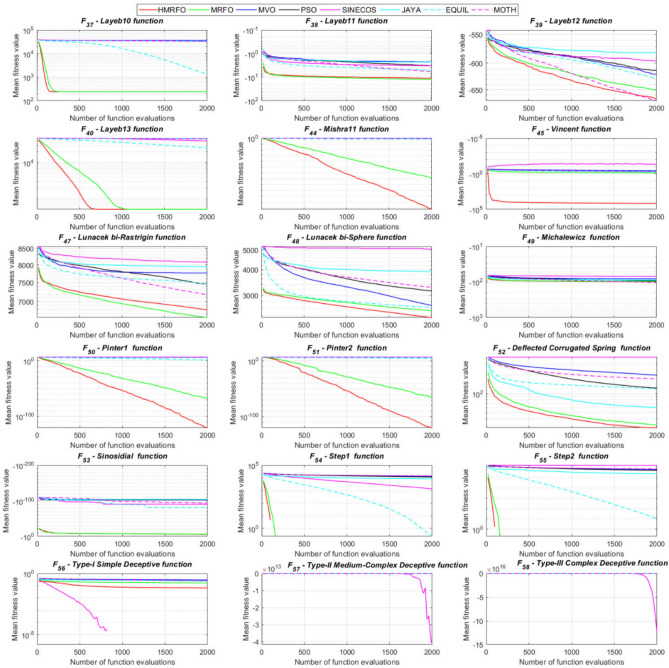
Evolution curves of the compared metaheuristic optimizers for 500D multimodal test problems from F_37_ to F_58_.

**Fig. 14:**
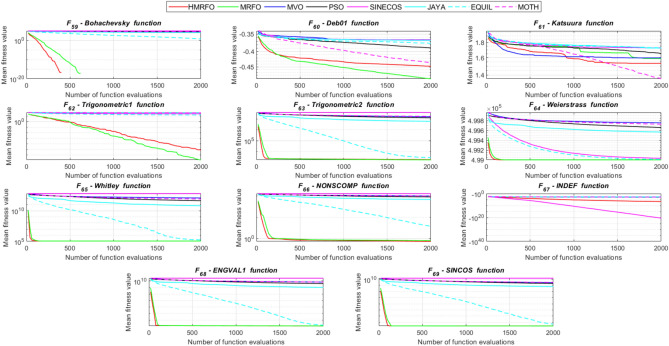
Optimal convergence curves for 500D multimodal test problems from F_59_ to F_69_.

**Fig. 15:**
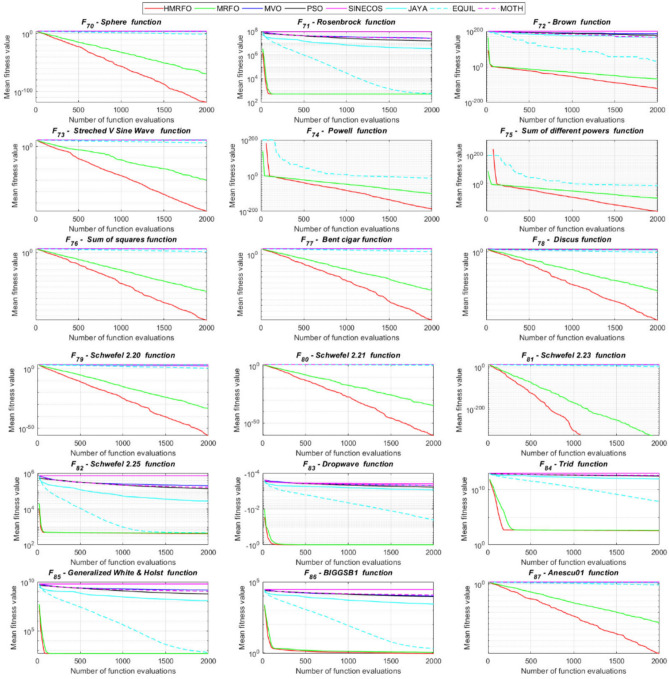
Iterative declines in objective function values for 500D unimodal problems from F_70_ to F_87_.

**Fig. 16:**
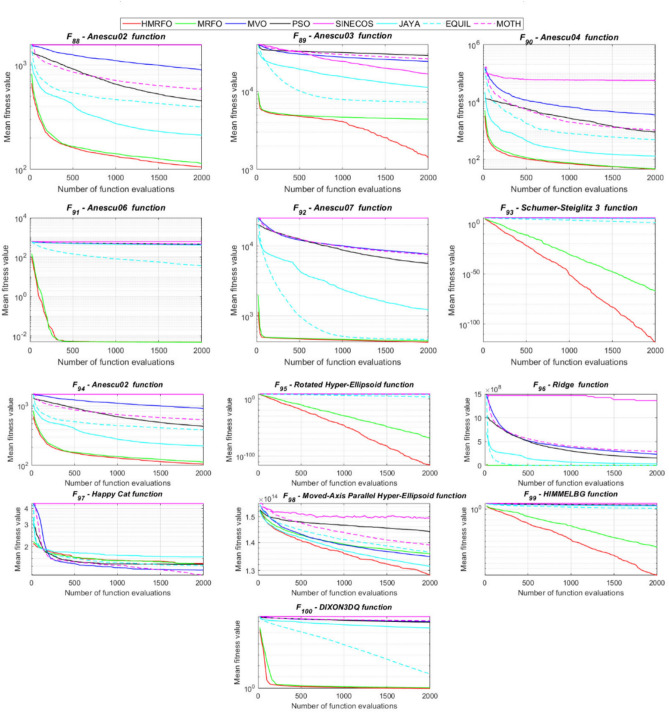
Optimal evolution of the convergence curves for the compared algorithms for 500D unimodal test functions from F_88_ to F_100_.

The optimization efficiency of the proposed algorithm will be evaluated in this section based on its prediction accuracy over 500D global benchmark functions. A comparative study between the developed HMFRO and the remaining algorithms used in the previous case is made to investigate how the hierarchical mutation scheme influences the overall probing effectivity of the proposed method and decide if it is feasible to integrate the hierarchical mutation scheme into the original MRFO algorithm to conquer the annoying effects of “curse of dimensionality.” Table 10 reports the predictive results obtained for 500D multimodal test functions for each compared algorithm. It is seen that MRFO reaches the global optimum answer of F_1_, F_2_, F_3_, F_4_, F_7_, F_8_, F_11_, F_15_, F_34_, F_37_, F_40_, F_51_, F_54_, F_55_, F_59_, and F_64_ test functions in each run, which is quite an achievement in solving hyperdimensional optimization problems within 2000 function evaluations. Furthermore, HMRFO obtains close predictions to global optimum solutions of the test functions, including F_5_, F_9_, F_10_, F_12_, F_13_, F_14_, F_21_, F_22_, F_44_, and F_50_. When average prediction values are in consideration, HMRFO is the superior algorithm in terms of solution accuracy in 41 out of 69 benchmark cases and proves its effectiveness in solving hyperdimensional problems. However, this algorithm surrenders to the high dimensionality of the employed problems since it fails to retain feasible predictions for test problems of F_41_, F_42_, F_43_, and F_46_. MRFO algorithm becomes the second-best performer when average estimations are taken into account. EQUIL again yields the best predictions between the literature metaheuristic algorithms. PSO, SINECOS, JAYA, and MOTH algorithm collapse at finding feasible results for F_12_ test functions. Convergence histories of the compared algorithms plotted for 500D multimodal test problems are shown in Figs. 11, 12, 13, and 14. Similar evolution tendencies are evident for 500D multimodal test problems as previously observed for 30D multi-modal problems. Gradual declines in objective function rates are observed in most cases, yet quick decreases followed by smooth declines are also seen in many instances. This tendency in the evolution of fitness values depends on the characteristics of the defined optimization problem, and no clear conclusion can be drawn as to a general convergence behavior of any problems since each test function has its solution domain characterizing its functional proclivities. Table 11 reports the Wilcoxon sum rank test results of 500D multimodal test problems. The dominance of the HMRFO algorithm is evident concerning the robustness and accuracy of the estimations, as understood from Table 11. Table 12 provides the predictions performed by the comparative algorithms for 500D unimodal benchmark problems. HMRFO arrives at the global optimum answer of F_74_, F_75_, F_81_, F_83_, and F_94_ test functions in each independent run. Furthermore, very accurate predictions close to the optimum point are observed for the F_87_, F_93_, F_95_, and F_99_ test functions for the HMRFO algorithm. Figures 15 and 16 visualize the convergence plots of the compared algorithms for 500D unimodal problems. Linear declines are observed with increasing iterations for the MRFO algorithm for most cases. It is also seen that compared algorithms are far from their optimal point while HMRFO is gradually closing to its optimum answer, which is valid for most test cases. Table 13 gives the statistical outcomes of the Wilcoxon sum rank test analysis performed at a 5% significance level. The indisputable superiority of the proposed HMRFO is verified by its average ranking performance, which is much better than the other remaining algorithms.

**Table 10:** Statistical results for 500D classical optimization problems.

		HMRFO	MRFO	MVO	PSO	SINECOS	JAYA	EQUIL	MOTH
F_1_	Min	**8.88E−16**	**8.88E−16**	1.08E+01	9.75E+00	1.32E+01	7.94E+00	1.58E−03	1.13E+01
	Mean	8.88E−16	8.88E−16	1.14E+01	1.03E+01	1.50E+01	8.31E+00	2.57E−03	1.15E+01
	Std. dev	0.00E+00	0.00E+00	3.29E−01	1.73E−01	3.93E+01	2.01E–01	6.53E−04	1.73E−01
	Max	8.88E−16	8.88E−16	1.19E+01	1.05E+01	1.52E+01	8.75E+00	4.69E−03	1.20E+01
F_2_	Min	**0.00E+00**	**0.00E+00**	1.73E+00	1.85E+00	3.90E+00	1.44E+00	2.70E−07	2.32E+00
	Mean	0.00E+00	0.00E+00	1.89E+00	1.94E+00	4.83E+00	1.50E+00	1.33E−06	2.43E+00
	Std. dev	0.00E+00	0.00E+00	4.89E−02	5.31E−02	1.93E−01	4.51E−02	1.00E−06	4.99E−02
	Max	0.00E+00	0.00E+00	1.99E+00	2.04E+00	5.00E+00	1.62E+00	5.25E−06	2.53E+00
F_3_	Min	**0.00E+00**	**0.00E+00**	1.00E+05	7.73E+03	1.43E+04	6.19E+03	2.47E−02	9.94E+03
	Mean	0.00E+00	0.00E+00	1.31E+04	8.39E+03	1.97E+04	6.81E+03	7.70E+00	1.03E+04
	Std. dev	0.00E+00	0.00E+00	5.36E+02	2.81E+02	1.64E+03	2.29E+02	6.87E+00	2.70E+02
	Max	0.00E+00	0.00E+00	1.23E+04	8.86E+03	2.09E+04	7.11E+03	2.82E+01	1.11E+04
F_4_	Min	**0.00E+00**	2.81E−159	1.15E+04	9.44E+03	7.09E+03	1.03E+04	3.42E+03	1.01E+04
	Mean	0.00E+00	4.10E−137	1.28E+04	1.99E+04	1.32E+06	1.50E+04	5.31E+03	1.24E+04
	Std. dev	0.00E+00	2.24E−136	7.98E+02	3.13E+04	3.82E+06	7.93E+03	1.15E+03	1.43E+03
	Max	0.00E+00	1.23E−135	1.45E+04	1.71E+05	1.59E+07	4.74E+04	8.86E+03	1.71E+04
F_5_	Min	**5.67E−150**	2.30E−83	7.26E+02	5.52E+02	9.38E+01	4.97E+02	7.34E−03	6.82E+02
	Mean	2.95E−123	6.00E−74	7.84E+82	5.95E+02	1.12E+03	5.46E+02	2.17E−02	7.36E+02
	Std. dev	1.61E−122	2.86E−72	3.59E+01	2.47E+01	3.75E+02	2.08E+01	7.78E−03	2.78E+01
	Max	8.83E−122	1.51E−71	8.72E+02	6.36E+02	1.45E+03	5.82E+02	4.53E−02	7.90E+02
F_6_	Min	**3.97E−01**	4.73E−01	1.00E+01	6.71E+00	3.36E+01	9.20E+00	7.67E−01	1.05E+01
	Mean	4.58E−01	5.95E−01	1.10E+01	7.79E+00	3.60E+01	1.05E+01	8.23E−01	1.16E+01
	Std. dev	3.28E−02	4.03E−02	6.67E−01	5.20E−01	1.08E+00	7.41E−01	3.07E−02	8.11E−01
	Max	5.10E−01	6.76E−01	1.24E+01	8.70E+00	3.83E+01	1.23E+01	9.06E−01	1.32E+01
F_7_	Min	**0.00E+00**	**0.00E+00**	7.19E+06	5.53E+06	1.12E+08	1.02E+06	2.71E−09	1.22E+07
	Mean	0.00E+00	0.00E+00	9.42E+06	7.76E+06	1.23E+08	1.63E+06	2.24E−07	1.75E+07
	Std. dev	0.00E+00	0.00E+00	1.44E+06	1.24E+06	5.19E+06	5.08E+05	4.54E−07	2.06E+06
	Max	0.00E+00	0.00E+00	1.29E+07	1.07E+08	1.31E+08	2.98E+06	2.49E−06	2.14E+07
F_8_	Min	**0.00E+00**	**0.00E+00**	1.48E+02	1.70E+02	2.15E+02	2.11E+02	1.75E+02	1.62E+02
	Mean	0.00E+00	0.00E+00	1.55E+02	1.97E+02	2.20E+02	2.18E+02	1.91E+02	1.74E+02
	Std. dev	0.00E+00	0.00E+00	3.72E+00	1.31E+01	2.91E+00	2.96E+00	6.42E+00	7.28E+00
	Max	0.00E+00	0.00E+00	1.62E+02	2.20E+02	2.25E+02	2.32E+02	2.02E+02	1.89E+02
F_9_	Min	**4.28E−157**	9.78E−85	8.39E+00	7.10E+00	7.77E+00	5.20E+00	5.99E−01	8.09E+00
	Mean	8.57E−127	2.21E−64	8.75E+00	7.49E+00	1.25E+01	5.72E+00	7.56E−01	8.58E+00
	Std. dev	4.69E−126	1.21E−63	2.43E−01	1.96E−01	9.19E−01	2.53E−01	1.04E−01	3.12E−01
	Max	2.57E−125	6.64E−63	9.49E+00	7.99E+00	1.30E+01	6.30E+00	8.99E−01	9.39E+00
F_10_	Min	**1.15E−313**	1.89E−162	3.19E+03	3.52E+03	1.21E+04	1.69E+03	9.90E−04	5.24E+03
	Mean	3.75E−256	3.39E−146	3.62E+03	3.90E+03	1.53E+04	2.02E+03	3.40E−03	5.71E+03
	Std. dev	0.00E+00	1.42E−145	1.67E+02	2.00E+02	8.50E+02	1.84E+02	1.83E−03	2.40E+02
	Max	1.12E−254	7.43E−145	3.97E+03	4.25E+03	1.61E+04	2.46E+02	8.26E−03	6.29E+03
F_11_	Min	**0.00E+00**	**0.00E+00**	9.05E−01	8.85E−01	2.16E−02	9.44E−01	1.27E−02	8.76E−01
	Mean	0.00E+00	0.00E+00	9.19E−01	9.21E−01	1.15E−01	9.57E−01	2.94E−01	8.89E−01
	Std. dev	0.00E+00	0.00E+00	5.71E−03	1.69E−02	1.66E−01	5.45E−03	2.50E−01	7.35E−03
	Max	0.00E+00	0.00E+00	9.31E−01	9:45E−01	8.98E−01	9.64E−01	7.44E−01	9.04E−01
F_12_	Min	**3.95E−203**	9.50E−66	2.45E+121	N/A	N/A	N/A	9.42E−21	N/A
	Mean	1.66E−176	4.33E−23	5.52E+227	N/A	N/A	N/A	7.57E−12	N/A
	Std. dev	0.00E+00	2.36E−22	3.01E+228	N/A	N/A	N/A	3.56E−11	N/A
	Max	2.73E−175	1.29E−21	1.65E+229	N/A	N/A	N/A	1.93E−10	N/A
F_13_	Min	1.47E−120	**1.74E−132**	4.56E−70	1.14E−85	2.53E−55	1.25E−54	1.68E−06	1.59E−86
	Mean	3.12E−89	2.97E−112	1.10E−55	4.55E−52	1.29E−48	3.81E−41	1.52E−78	6.53E−73
	Std. dev	1.70E−88	1.51E−111	4.60E−55	2.49E−51	3.77E−48	1.76E−40	8.33E−78	3.04E−72
	Max	9.36E−88	8.27E−111	2.47E−54	1.36E−50	1.76E−47	9.57E−40	4.56E−77	1.66E−71
F_14_	Min	1.47E−120	**1.74E−132**	4.56E−70	1.14E−85	2.53E−55	1.25E−54	1.68E−96	1.59E−86
	Mean	3.12E−89	2.97E−112	1.10E−55	4.54E−52	1.29E−48	3.81E−41	1.52E−78	6.53E−73
	Std. dev	1.70E−88	1.51E−111	4.60E−55	2.49E−51	3.77E−48	1.76E−40	8.33E−78	3.04E−72
	Max	9.36E−88	8.27E−111	2.47E−54	1.36E−50	1.76E−47	9.57E−40	4.56E−77	1.66E−71
F_15_	Min	**0.00E+00**	**0.00E+00**	2.08E+02	2.10E+02	2.24E+02	2.26E+02	2.02E+02	1.87E+02
	Mean	0.00E+00	0.00E+00	2.16E+02	2.22E+02	2.29E+02	2.29E+02	2.18E+02	1.97E+02
	Std. dev	0.00E+00	0.00E+00	2.75E+00	5.29E+00	1.97E+00	1.69E+00	4.96E+00	4.21E+00
	Max	0.00E+00	0.00E+00	2.21E+02	2.28E+02	2.33E+02	2.32E+02	2.28E+02	2.04E+02
F_16_	Min	**1.15E+03**	1.30E+03	4.76E+05	5.13E+05	7.15E+06	6.16E+04	1.64E+03	1.05E+06
	Mean	1.25E+03	1.42E+02	6.67E+05	6.71E+05	7.69E+06	1.11E+05	1.69E+03	1.25E+06
	Std. dev	4.89E+01	4.52E+01	1.11E+05	8.35E+04	2.66E+05	3.02E+04	2.77E+01	1.40E+05
	Max	1.34E+03	1.47E+03	9.37E+05	9.10E+05	8.21E+06	1.70E+05	1.74E+03	1.55E+06
F_17_	Min	**6.00E+01**	8.29E+01	1.53E+03	1.05E+03	3.39E+03	2.19E+02	1.18E+02	1.53E+03
	Mean	7.01E+01	9.42E+01	1.73E+03	1.23E+03	5.14E+03	2.96E+02	1.28E+02	1.81E+03
	Std. dev	6.11E+00	6.04E+00	1.19E+02	1.10E+02	8.09E+02	4.37E+01	4.12E+00	1.09E+02
	Max	8.50E+01	1.05E+02	2.01E+03	1.44E+03	5.96E+03	4.10E+02	1.37E+02	2.10E+03
F_18_	Min	**3.03E+07**	3.07E+07	3.25E+07	3.32E+07	3.28E+07	3.32E+07	3.32E+07	3.34E+07
	Mean	3.16E+07	3.19E+07	3.32E+07	3.38E+07	3.48E+07	3.36E+07	3.37E+07	3.37E+07
	Std. dev	6.58E+05	5.41E+05	3.26E+05	1.89E+05	1.09E+06	1.36E+05	1.66E+05	1.77E+05
	Max	3.29E+07	3.28E+07	3.37E+07	3.41E+07	3.67E+07	3.39E+07	3.39E+07	3.41E+07
F_19_	Min	− 2.75E+05	− 2.41E+05	− 1.91E+05	− 1.87E+05	**3.47E+05**	− 3.28E+05	− 1.69E+05	− 1.35E+05
	Mean	− 2.42E+05	− 2.01E+05	− 1.51E+05	− 1.43E+05	4.24E+05	− 3.08E+02	− 1.46E+05	− 1.12E+05
	Std. dev	1.51E+04	1.61E+04	1.72E+04	2.01E+04	2.98E+04	1.01E+04	9.97E+03	1.55E+04
	Max	− 2.17E+05	− 1.72E+05	− 1.11E+05	− 1.01E+05	4.80E+05	− 2.80E+05	− 1.27E+05	− 7.84E+04
F_20_	Min	− 5.65E+03	− 4.50E+03	1.08E+04	1.25E+04	3.17E+05	**− 7.02E+03**	− 2.47E+03	2.76E+04
	Mean	− 5.11E+03	− 3.92E+03	1.41E+04	2.03E+04	4.10E+05	− 5.62E+03	− 2.05E+03	3.66E+04
	Std. dev	3.18E+02	3.25E+02	1.98E+03	3.47E+03	3.71E+04	8.35E+02	1.82E+02	5.86E+03
	Max	− 4.45E+03	− 3.03E+03	1.96E+04	2.84E+04	4.71E+05	− 2.37E+03	− 1.72E+03	4.85E+04
F_21_	Min	**7.73E−312**	9.73E−166	3.32E+04	6.11E+04	4.52E+04	3.13E+04	1.24E−03	9.59E+04
	Mean	3.54E−260	1.91E−144	3.72E+04	6.86E+04	2.20E+05	4.25E+04	3.39E−03	1.09E+05
	Std. dev	0.00E+00	1.03E−143	2.55E+03	5.01E+03	1.06E+05	4.57E+03	1.54E−03	7.68E+03
	Max	9.86E−259	5.68E−143	4.18E+04	7.96E+04	3.77E+05	5.61E+04	6.62E−03	1.26E+05
F_22_	Min	**1.49E−315**	1.84E−169	7.25E+03	9.49E+03	6.58E+03	1.54E+04	1.54E−03	1.29E−04
	Mean	1.53E−260	1.93E−148	8.80E+03	1.02E+04	2.56E+04	2.15E+03	6.71E−03	1.48E+04
	Std. dev	0.00E+00	8.36E−148	6.40E+02	5.37E+02	8.93E+03	3.53E+03	4.22E−03	8.83E+02
	Max	4.60E−259	4.46E−147	9.78E+03	1.17E+04	3.93E+04	2.81E+04	1.74E−02	1.67E+04
F_23_	Min	**− 1.46E+02**	− 1.39E+02	− 1.36E+02	− 1.29E+02	− 6.77E+01	− 8.22E+01	− 1.19E+02	− 1.91E+02
	Mean	− 8.21E+01	− 8.40E+01	− 1.14E+02	− 8.18E+01	− 5.44E+01	− 6.29E+01	− 9.73E+01	− 1.59E+02
	Std. dev	2.40E+01	2.33E+01	9.87E+00	1.63E+01	5.96E+00	6.03E+00	1.17E+01	1.61E+01
	Max	− 6.13E+01	− 5.95E+01	− 9.43E+01	− 6.37E+01	− 4.61E+01	− 5.24E+01	− 7.73E+01	− 1.30E+02
F_24_	Min	− 2.56E+05	− 2.07E+05	− 1.67E+05	− 1.69E+05	3.98E+05	**− 3.27E+05**	− 1.55E+05	− 1.40E+05
	Mean	− 2.31E+05	− 1.88E+05	− 1.30E+05	− 1.34E+05	4.90E+05	− 3.05E+05	− 1.43E+05	− 1.06E+05
	Std. dev	1.15E+04	1.04E+04	2.25E+04	1.62E+04	4.59E+04	1.18E+04	7.08E+03	1.98E+04
	Max	− 2.11E+05	− 1.64E+05	− 7.71E+04	− 1.00E+05	5.99E+05	− 2.77E+05	− 1.24E+05	− 7.28E+04
F_25_	Min	− 1.78E+02	− 1.10E+02	− 1.37E+02	− 1.18E+02	− 7.63E+01	− 6.89E+01	− 1.24E+02	**− 1.8E+02**
	Mean	− 8.66E+01	− 7.68E+01	− 1.19E+02	− 8.24E+01	− 5.41E+01	− 6.18E+01	− 9.45E+01	− 1.61E+02
	Std. dev	2.97E+01	1.24E+01	8.80E+00	1.57E+01	6.88E+00	3.46E+00	1.52E+01	1.40E+01
	Max	− 5.92E+01	− 6.40E+01	− 1.01E+02	− 6.17E+01	− 4.57E+01	− 5.54E+01	− 6.76E+01	− 1.33E+02
F_26_	Min	**− 1.50E+07**	− 5.78E+02	4.79E+04	7.62E+04	3.03E+06	1.15E+05	− 4.70E+02	1.62E+05
	Mean	− 8.65E+05	− 4.63E+02	7.02E+04	1.18E+05	3.67E+06	1.85E+05	− 3.80E+02	2.51E+05
	Std. dev	2.85E+06	5.85E+01	1.57E+04	2.76E+04	3.44E+05	4.09E+04	4.91E+01	4.86E+04
	Max	− 8.53E+03	− 3.77E+02	1.15E+05	1.76E+05	4.31E+06	2.87E+05	− 2.56E+02	3.54E+05
F_27_	Min	**3.58E+02**	4.03E+02	2.60E+05	2.48E+05	2.53E+06	5.33E+04	4.63E+02	4.01E+05
	Mean	4.04E+02	4.24E+02	3.61E+05	3.35E+05	2.83E+06	8.08E+04	4.68E+02	4.92E+05
	Std. dev	8.06E+00	8.20E+00	4.57E+04	5.83E+04	1.57E+05	1.40E+04	2.95E+00	5.39E+04
	Max	4.20E+02	4.37E+02	4.61E+05	4.75E+05	3.09E+06	1.08E+05	4.77E+02	6.21E+05
F_28_	Min	7.90E−02	**6.81E−02**	7.44E+04	5.77E+04	3.88E+05	2.09E+04	1.90E−01	1.01E+05
	Mean	1.12E−01	1.44E−01	9.46E+04	6.76E+04	4.36E+05	3.32E+04	1.37E+00	1.16E+05
	Std. dev	1.68E−02	5.17E−02	1.14E+04	6.64E+03	1.94E+04	1.12E+04	2.90E+00	1.01E+04
	Max	1.50E−01	2.56E−01	1.18E+05	7.91E+04	4.72E+05	7.42E+04	1.65E+01	1.49E+05
F_29_	Min	**− 7.10E+03**	− 6.96E+03	− 5.36E+03	− 5.02E+03	− 4.27E+03	− 5.17E+03	− 5.10E+03	− 5.08E+03
	Mean	− 6.31E+03	− 6.16E+03	− 5.12E+03	− 4.83E+03	− 3.63E+03	− 4.92E+03	− 4.89E+03	− 4.92E+03
	Std. dev	3.90E+02	3.79E+02	1.28E+02	7.79E+01	4.61E+02	1.02E+02	1.01E+02	8.91E+01
	Max	− 5.67E+03	− 5.42E+03	− 4.86E+03	− 4.72E+03	− 2.95E+03	− 4.75E+03	− 4.72E+03	− 4.75E+03
F_30_	Min	2.49E−02	**2.47E−02**	2.78E−02	2.66E−02	2.79E−02	2.60E−02	2.53E−02	2.72E−02
	Mean	2.51E−02	2.48E−02	2.82E−02	2.68E−02	2.84E−02	2.64E−02	2.55E−02	2.76E−02
	Std. dev	9.75E−05	6.92E−05	1.98E−04	1.32E−04	3.65E−04	1.63E−04	8.75E−05	1.63E−04
	Max	2.53E−02	2.50E−02	2.85E−02	2.70E−02	2.91E−02	2.67E−02	2.56E−02	2.80E−02
F_31_	Min	**− 2.99E+03**	**− 2.99E+03**	3.99E+02	− 8.24E+01	− 2.99E+03	− 1.76E+02	− 2.97E+03	2.25E+02
	Mean	− 2.96E+03	− 2.95E+03	5.79E+02	− 2.24E−01	− 2.98E+03	− 9.06E+01	− 2.96E+03	2.95E+02
	Std. dev	1.13E+01	1.58E+01	6.88E+01	4.81E+01	9.00E+00	5.56E+01	4.76E+00	4.20E+01
	Max	− 2.94E+03	− 2.94E+03	7.11E+02	7.85E+01	− 2.95E+03	6.54E+01	− 2.95E+03	3.69E+02
F_32_	Min	− 3.78E+02	**− 5.04E+02**	− 4.11E+02	− 4.08E+02	− 3.11E+02	− 3.05E+02	− 4.15E+02	− 4.98E+02
	Mean	− 3.23E+02	− 3.83E+02	− 3.80E+02	− 3.43E+02	− 2.97E+02	− 2.91E+02	− 3.66E+02	− 4.53E+02
	Std. dev	2.16E+01	5.23E+01	2.23E+01	2.79E+01	8.90E+00	7.29E+00	1.82E+01	1.87E+01
	Max	− 2.96E+02	− 3.01E+02	− 3.34E+02	− 3.04E+02	− 2.80E+02	− 2.81E+02	− 3.23E+02	− 4.21E+02
F_33_	Min	4.25E+02	**4.22E+02**	4.51E+02	4.44E+02	4.57E+02	4.65E+02	4.47E+02	4.47E+02
	Mean	4.37E+02	4.38E+02	4.57E+02	4.59E+02	4.69E+02	4.67E+02	4.56E+02	4.53E+02
	Std. dev	8.40E+00	6.49E+00	3.22E+00	7.12E+00	1.17E+00	6.81E−01	3.75E+00	3.48E+00
	Max	4.61E+02	4.53E+02	4.64E+02	4.68E+02	4.71E+02	4.68E+02	4.63E+02	4.60E+02
F_34_	Min	**1.19E+03**	**1.19E+03**	4.55E+04	4.59E+04	1.67E+04	4.66E+04	4.55E+04	4.48E+04
	Mean	1.19E+03	1.19E+03	4.50E+04	4.64E+04	4.53E+04	4.67E+04	4.62E+04	4.53E+04
	Std. dev	2.31E−13	2.31E−13	2.62E+02	2.06E+02	5.88E+03	5.34E+01	2.20E+02	1.94E+02
	Max	1.19E+03	1.19E+03	4.66E+04	4.67E+04	4.68E+04	4.68E+04	4.66E+04	4.58E+04
F_35_	Min	2.63E+04	**2.17E+04**	2.38E+04	2.62E+04	2.84E+04	2.93E+04	2.52E+03	2.38E+04
	Mean	2.81E+04	2.42E+04	2.49E+04	2.82E+04	2.99E+04	2.99E+04	2.66E+04	2.49E+04
	Std. dev	8.42E+02	2.00E+03	3.63E+02	9.79E+02	3.52E+02	1.94E+02	7.06E+02	5.80E+02
	Max	2.95E+04	2.95E+04	2.56E+04	2.99E+04	3.03E+04	3.02E+04	2.77E+04	2.66E+04
F_36_	Min	5.83E+02	**5.68E+02**	1.20E+03	1.10E+03	3.89E+03	8.41E+02	5.49E+02	1.32E+03
	Mean	5.83E+02	5.83E+02	1.38E+03	1.26E+03	4.25E+03	9.05E+02	5.57E+02	1.49E+03
	Std. dev	2.31E−13	2.74E+00	1.16E+02	9.35E+01	1.60E+02	4.46E+01	4.62E+00	8.08E+01
	Max	5.83E+02	5.83E+02	1.68E+03	1.48E+03	4.56E+03	9.91E+02	5.64E+02	1.70E+03
F_37_	Min	**2.39E+02**	**2.39E+02**	2.98E+04	2.73E+04	3.68E+03	3.25E+04	2.60E+02	2.87E+04
	Mean	2.39E+02	2.39E+02	3.08E+04	2.97E+04	3.06E+04	3.37E+04	3.50E+02	3.01E+04
	Std. dev	1.73E−13	1.73E−13	6.25E+02	9.33E+02	8.77E+03	4.64E+02	1.43E+02	6.14E+02
	Max	2.39E+02	2.39E+02	3.21E+04	3.17E+04	3.67E+04	3.46E+04	9.41E+02	3.15E+04
F_38_	Min	− 3.29E+01	**− 3.77E+01**	− 4.59E+00	− 6.12E+00	− 5.56E+00	− 3.21E+00	− 1.43E+01	− 1.50E+01
	Mean	− 1.38E+01	− 1.69E+01	− 3.05E+00	− 3.93E+00	− 3.79E+00	− 2.61E+00	− 9.07E+00	− 1.10E+01
	Std. dev	6.62E+00	6.66E+00	5.86E−01	7.79E−01	6.35E−01	2.76E−01	1.82E+00	1.27E+00
	Max	− 7.74E+00	− 8.37E+00	− 2.09E+00	− 2.51E+00	− 2.88E+00	− 2.01E+00	− 5.93E+00	− 9.21E+00
F_39_	Min	**− 8.46E+02**	− 7.96E+02	− 7.37E+02	− 7.55E+02	− 6.28E+02	− 6.00E+02	− 7.99E+02	− 8.05E+02
	Mean	− 7.63E+02	− 7.17E+02	− 6.94E+02	− 6.76E+02	− 6.05E+02	− 5.85E+02	− 7.39E+02	− 7.53E+02
	Std. dev	5.49E+01	5.16E+01	3.03E+01	3.83E+01	1.04E+01	5.93E+00	2.31E+01	2.32E+01
	Max	− 6.36E+02	− 6.18E+02	− 6.36E+02	− 6.20E+02	− 5.85E+02	− 5.73E+02	− 6.92E+02	− 6.92E+02
F_40_	Min	**4.99E+02**	**4.99E+02**	4.62E+04	4.48E+04	9.68E+03	4.63E+04	1.78E+04	4.53E+04
	Mean	4.99E+02	4.99E+02	4.66E+04	4.52E+04	2.61E+04	4.65E+04	1.90E+04	1.45E+04
	Std. dev	0.00E+00	0.00E+00	2.19E+02	2.12E+02	1.54E+04	1.33E+02	5.45E+02	1.71E+02
	Max	4.99E+02	4.99E+02	4.70E+04	4.57E+04	4.82E+04	4.68E+04	1.99E+04	4.61E+04
F_41_	Min	N/A	N/A	N/A	N/A	N/A	N/A	N/A	N/A
	Mean	N/A	N/A	N/A	N/A	N/A	N/A	N/A	N/A
	Std. dev	N/A	N/A	N/A	N/A	N/A	N/A	N/A	N/A
	Max	N/A	N/A	N/A	N/A	N/A	N/A	N/A	N/A
F_42_	Min	N/A	N/A	N/A	N/A	N/A	N/A	N/A	N/A
	Mean	N/A	N/A	N/A	N/A	N/A	N/A	N/A	N/A
	Std. dev	N/A	N/A	N/A	N/A	N/A	N/A	N/A	N/A
	Max	N/A	N/A	N/A	N/A	N/A	N/A	N/A	N/A
F_43_	Min	N/A	N/A	N/A	N/A	N/A	N/A	N/A	N/A
	Mean	N/A	N/A	N/A	N/A	N/A	N/A	N/A	N/A
	Std. dev	N/A	N/A	N/A	N/A	N/A	N/A	N/A	N/A
	Max	N/A	N/A	N/A	N/A	N/A	N/A	N/A	N/A
F_44_	Min	**5.07E−295**	5.06E−169	1.57E−01	1.68E−01	1.18E+00	2.54E−01	2.11E−02	2.58E−01
	Mean	1.56E−243	2.12E−142	2.17E−01	2.11E−01	1.32E+00	4.72E−01	2.72E−02	3.18E−01
	Std. dev	0.00E+00	1.16E−141	3.92E−02	2.60E−02	8.27E−02	1.25E−01	4.07E−03	4.40E−02
	Max	4.70E−242	6.38E−141	3.14E−01	2.59E−01	1.46E+00	8.82E−01	3.75E−02	4.03E−01
F_45_	Min	**− 9.73E+03**	− 8.60E−01	− 5.92E−01	− 6.85E−01	− 7.18E−02	− 6.45E−01	− 6.56E−01	− 6.09E−01
	Mean	− 1.60E+04	− 8.20E−01	− 5.27E−01	− 6.43E−01	− 4.28E−02	− 5.91E−01	− 6.21E−01	− 5.71E−01
	Std. dev	5.04E+03	3.67E−02	3.23E−02	2.40E−02	1.44E−02	2.62E−02	1.46E−02	1.71E−02
	Max	− 2.04E+04	− 6.97E−01	− 4.28E−01	− 5.82E−01	1.39E−02	− 5.20E−01	− 5.95E−01	− 5.40E−01
F_46_	Min	N/A	N/A	N/A	N/A	N/A	N/A	N/A	N/A
	Mean	N/A	N/A	N/A	N/A	N/A	N/A	N/A	N/A
	Std. dev	N/A	N/A	N/A	N/A	N/A	N/A	N/A	N/A
	Max	N/A	N/A	N/A	N/A	N/A	N/A	N/A	N/A
F_47_	Min	**5.68E+03**	5.74E+03	6.36E+03	6.29E+03	7.74E+03	7.72E+03	6.22E+03	6.35E+03
	Mean	6.28E+03	6.08E+03	6.62E+03	6.64E+03	8.05E+03	7.83E+03	6.66E+03	6.59E+03
	Std. dev	3.65E+02	1.61E+02	1.33E+02	1.62E+02	1.07E+02	9.34E+01	1.74E+02	1.18E+02
	Max	6.98E+03	6.37E+03	6.86E+03	7.02E+03	8.27E+03	8.04E+03	7.10E+03	6.84E+03
F_48_	Min	1.75E+03	2.60E+03	**1.69E+03**	2.46E+03	4.00E+03	3.54E+03	2.40E+03	2.64E+03
	Mean	1.86E+03	2.16E+03	1.87E+03	2.63E+03	4.90E+03	3.82E+03	2.51E+03	2.81E+03
	Std. dev	5.31E+01	6.35E+01	7.87E+01	7.95E+01	4.00E+02	1.16E+02	5.24E+01	9.25E+01
	Max	1.93E+03	2.29E+03	2.05E+03	2.79E+03	5.36E+03	4.05E+03	2.61E+03	3.04E+03
F_49_	Min	− 1.08E+02	− 1.17E+02	− 9.22E+01	− 1.17E+02	− 7.88E+01	− 9.87E+01	− 1.06E+02	**− 1.40E+02**
	Mean	− 1.00E+02	− 1.00E+02	− 8.48E+01	− 1.03E+02	− 7.18E+01	− 9.13E+01	− 9.78E+01	− 1.27E+02
	Std. dev	2.93E+00	4.69E+00	2.90E+00	6.55E+00	4.13E+00	3.48E+00	4.10E+00	5.61E+00
	Max	− 9.57E+01	− 9.43E+01	− 7.94E+00	− 9.30E+01	− 6.37E+01	− 8.37E+01	− 9.19E+01	− 1.16E+02
F_50_	Min	**7.38E−306**	5.16E−159	7.57E+05	9.32E+05	4.58E+06	4.53E+05	3.95E−02	1.38E+06
	Mean	3.63E−241	1.20E−146	9.19E+05	1.05E+06	4.78E+06	5.58E+05	8.94E−02	1.55E+06
	Std. dev	0.00E+00	3.61E−146	8.45E+04	6.28E+04	1.10E+05	5.85E+04	3.64E−02	7.84E+04
	Max	1.09E−239	1.67E−145	1.08E+06	1.19E+06	4.99E+06	6.94E+05	1.96E−01	1.73E+06
F_51_	Min	**0.00E+00**	1.39E−160	1.94E+06	2.02E+06	2.75E+06	1.47E+06	2.20E+01	2.46E+06
	Mean	1.30E−246	2.92E−144	2.07E+06	2.14E+06	4.88E+06	1.59E+06	6.29E+01	2.64E+06
	Std. dev	0.00E+00	1.47E−143	6.31E+04	6.73E+04	7.61E+06	6.90E+04	3.16E+01	9.75E+04
	Max	3.96E−245	8.08E−143	2.17E+06	2.30E+06	5.61E+06	1.82E+06	1.80E+02	2.86E+06
F_52_	Min	1.79E+01	2.15E+01	1.06E+02	8.72E+01	3.69E+02	4.42E+01	8.45E+01	**1.30E+02**
	Mean	2.29E+01	2.48E+01	1.17E+02	9.62E+01	3.86E+02	5.35E+01	1.00E+02	1.43E+02
	Std. dev	2.72E+00	2.56E+00	5.24E+00	5.35E+00	9.70E+00	4.59E+00	6.34E+00	6.85E+00
	Max	2.96E+01	2.96E+01	1.25E+02	1.08E+02	4.06E+02	6.87E+01	1.12E+02	1.58E+02
F_53_	Min	**− 3.45E−04**	− 7.55E−05	− 1.78E−71	− 2.44E−98	− 2.34E−91	− 3.90E−103	− 1.90E−61	− 1.53E−82
	Mean	− 8.59E−05	− 1.34E−05	− 6.02E−73	− 8.14E−100	− 1.26E−92	− 4.44E−104	− 6.77E−63	− 6.69E−84
	Std. dev	1.10E−04	1.96E−05	3.25E−72	4.46E−99	4.70E−92	1.04E−103	3.47E−62	2.88E−83
	Max	− 6.45E−11	− 2.89E−13	− 1.53E−95	− 3.92E−112	− 8.18E−111	− 5.37E−111	− 1.20E−96	− 1.54E−102
F_54_	Min	**0.00E+00**	**0.00E+00**	9.38E+03	8.97E+03	7.00E+00	6.80E+03	0.00E+00	1.17E+04
	Mean	0.00E+00	0.00E+00	1.01E+04	9.83E+03	3.67E+01	7.61E+03	0.00E+00	1.24E+04
	Std. dev	0.00E+00	0.00E+00	3.50E+02	3.39E+02	2.30E+01	3.30E+02	0.00E+00	4.36E+02
	Max	0.00E+00	0.00E+00	1.08E+04	1.04E+04	1.06E+02	8.41E+03	0.00E+00	1.34E+04
F_55_	Min	**0.00E+00**	**0.00E+00**	3.28E+05	3.43E+05	1.10E+06	1.70E+05	0.00E+00	5.17E+05
	Mean	0.00E+00	0.00E+00	3.57E+05	3.95E+05	1.52E+06	2.00E+05	3.00E−01	5.65E+50
	Std. dev	0.00E+00	0.00E+00	1.54E+04	2.48E+04	1.11E+05	1.41E+04	5.34E−01	3.03E+04
	Max	0.00E+00	0.00E+00	3.88E+05	4.51E+05	1.61E+06	2.29E+05	2.00E+00	6.45E+05
F_56_	Min	2.43E−02	1.22E−01	2.20E−01	2.09E−01	**0.00E+00**	2.24E−01	2.32E−01	2.26E−01
	Mean	5.87E−02	1.45E−01	2.36E−01	2.22E−01	0.00E+00	2.38E−01	2.47E−01	2.38E−01
	Std. dev	4.98E−02	1.19E−02	8.06E−03	6.55E−03	0.00E+00	5.22E−03	7.12E−03	6.44E−03
	Max	2.40E−01	1.71E−01	2.56E−01	2.35E−01	0.00E+00	2.45E−01	2.62E−01	2.50E−01
F_57_	Min	− 9.12E+06	2.72E−01	3.07E−01	2.88E−01	**− 5.94E+30**	2.97E−01	2.78E−01	2.95E−01
	Mean	− 4.85E+05	2.81E−01	3.18E−01	2.95E−01	− 4.68E−29	3.07E−01	2.87E−01	3.03E−01
	Std. dev	1.66E+06	3.16E−03	5.62E−03	4.91E−03	1.23E+30	6.02E−03	3.28E−03	4.00E−03
	Max	− 1.99E+03	2.85E−01	3.34E−01	3.06E−01	− 2.19E+26	3.21E−01	2.94E−01	3.10E−01
F_58_	Min	− 7.41E+04	1.22E−01	2.38E−01	1.90E−01	**− 1.24E+38**	1.79E−01	2.13E−01	2.26E−01
	Mean	− 2.11E+04	1.36E−01	2.56E−01	2.05E−01	− 7.44E+36	1.93E−01	2.24E−01	2.40E−01
	Std. dev	1.77E+04	6.15E−03	7.90E−03	7.21E−03	2.31E+37	8.29E−03	7.18E−03	6.81E−03
	Max	− 3.53E+03	1.48E−01	2.68E−01	2.19E−01	− 3.53E+33	2.09E−01	2.36E−01	2.51E−01
F_59_	Min	**0.00E+00**	**0.00E+00**	2.12E+03	2.49E+04	7.38E+04	1.17E+04	9.73E−03	3.61E+04
	Mean	0.00E+00	0.00E+00	2.41E+04	2.67E+04	1.02E+05	1.36E+04	2.23E−02	3.88E+04
	Std. dev	0.00E+00	0.00E+00	1.42E+03	1.45E+03	7.34E+03	8.33E+02	9.59E−03	1.67E+03
	Max	0.00E+00	0.00E+00	2.70E+04	2.99E+04	1.07E+05	1.51E+04	5.24E−02	4.36E+04
F_60_	Min	− 5.25E−01	**− 6.15E−01**	− 5.08E−01	− 4.93E−01	− 3.80E−01	− 3.80E−01	− 4.68E−01	− 5.16E−01
	Mean	− 4.65E−01	− 5.37E−01	− 4.88E−01	− 4.46E−01	− 3.69E−01	− 3.70E−01	− 4.37E−01	− 4.93E−01
	Std. dev	2.36E−02	3.03E−02	1.07E−02	2.79E−02	5.38E−03	4.65E−03	1.61E−02	1.20E−02
	Max	− 4.16E−01	− 4.68E−01	− 4.65E−01	− 3.91E−01	− 3.58E−01	− 3.62E−01	− 4.12E−01	− 4.64E−01
F_61_	Min	**0.00E+00**	**0.00E+00**	1.48E+00	1.40E+00	1.58E+00	1.61E+00	1.60E+00	8.29E−01
	Mean	1.37E+00	1.45E+00	1.59E+00	1.58E+00	1.69E+00	1.71E+00	1.70E+00	1.00E+00
	Std. dev	6.30E−01	5.80E−01	4.47E−02	7.16E−02	4.67E−02	3.90E−02	4.95E−02	9.95E−02
	Max	1.73E+00	1.74E+00	1.66E+00	1.70E+00	1.76E+00	1.76E+00	1.80E+00	1.27E+00
F_62_	Min	5.78E−84	**7.79E−88**	3.73E+07	5.62E+07	9.43E+05	4.39E+07	3.78E+05	5.70E+07
	Mean	1.01E−64	1.32E−77	4.19E+07	6.67E+07	6.61E+06	5.18E+07	5.09E+05	6.66E+07
	Std. dev	5.38E−64	4.57E−77	2.81E+06	5.39E+06	3.13E+06	3.62E+06	8.17E+04	4.20E+06
	Max	2.95E−63	2.37E−76	4.71E+07	7.87E+07	1.39E+07	5.79E+07	7.07E+05	7.27E+07
F_63_	Min	**1.14E+03**	1.27E+03	7.75E+06	8.62E+06	2.35E+07	4.20E+06	2.14E+03	1.29E+07
	Mean	1.48E+03	1.46E+03	9.04E+06	9.65E+06	3.81E+07	5.05E+06	2.24E+03	1.46E+07
	Std. dev	1.66E+02	9.50E+01	5.75E+05	5.93E+05	3.24E+06	4.19E+05	6.97E+01	8.69E+05
	Max	1.82E+03	1.64E+03	1.01E+07	1.09E+07	4.03E+07	5.85E+06	2.41E+03	1.69E+07
F_64_	Min	**4.99E+05**	**4.99E+05**	**4.99E+05**	**4.99E+05**	**4.99E+05**	**4.99E+05**	**4.99E+05**	**4.99E+05**
	Mean	4.99E+05	4.99E+05	4.99E+05	4.99E+05	4.99E+05	4.99E+05	4.99E+05	4.99E+05
	Std. dev	0.00E+00	0.00E+00	1.82E+01	1.26E+01	6.49E−01	1.19E+01	2.80E−01	1.14E+01
	Max	4.99E+00	4.99E+00	4.99E+05	4.99E+05	4.99E+05	4.99E+05	4.99E+05	4.99E+05
F_65_	Min	**1.14E+05**	**1.14E+05**	1.70E+11	1.73E+11	5.10E+12	1.50E+10	1.15E+05	3.87E+11
	Mean	1.14E+05	1.14E+05	3.04E+11	2.36E+11	6.05E+12	3.16E+10	1.21E+05	5.88E+11
	Std. dev	4.05E+01	2.99E+01	5.13E+10	4.16E+10	3.33E+11	1.19E+10	6.33E+03	1.01E+11
	Max	1.14E+05	1.14E+05	4.11E+11	3.79E+11	6.74E+12	5.87E+10	1.49E+05	8.16E+11
F_66_	Min	**5.63E−02**	1.04E−01	3.26E+09	3.39E+09	3.27E+10	7.86E+08	1.57E+00	6.50E+09
	Mean	1.02E−01	2.24E−01	4.19E+09	4.30E+09	3.53E+10	1.07E+09	2.71E+00	7.76E+09
	Std. dev	4.90E−02	1.20E−01	4.88E+08	4.55E+08	1.20E+09	2.08E+08	1.41E+00	5.68E+08
	Max	2.10E−01	4.71E−03	5.27E+09	5.01E+09	3.78E+10	1.62E+09	8.39E+00	8.67E+09
F_67_	Min	**− 9.41E+07**	− 1.41E+03	− 1.50E+03	− 1.03E+03	− 7.47E+40	− 6.20E+02	− 1.28E+03	− 1.24E+03
	Mean	− 2.98E+07	− 1.23E+03	1.22E+03	− 9.06E+02	− 7.01E+39	− 5.29E+02	− 1.20E+03	− 1.10E+03
	Std. dev	2.09E+07	9.76E+01	1.26E+02	8.11E+01	1.58E+40	4.84E+01	4.69E+01	7.11E+01
	Max	− 6.14E+06	− 1.02E+03	− 9.85E+02	− 7.22E+02	− 9.41E+36	− 4.24E+02	− 1.07E+03	− 8.92E+02
F_68_	Min	**1.09E+03**	1.16E+03	2.11E+09	2.17E+09	2.39E+10	4.99E+08	1.36E+03	4.63E+09
	Mean	1.12E+03	1.19E+03	2.61E+09	2.72E+09	2.70E+10	7.62E+08	1.40E+03	5.35E+09
	Std. dev	1.84E+01	1.50E+01	2.92E+08	3.04E+08	1.35E+09	1.45E+08	1.39E+01	4.68E+08
	Max	1.16E+03	1.21E+03	3.19E+09	3.42E+09	2.92E+10	1.10E+09	1.42E+03	7.13E+09
F_69_	Min	2.42E+02	**2.33E+02**	8.09E+08	9.61E+08	1.30E+10	3.77E+08	2.43E+02	1.84E+09
	Mean	2.44E+02	2.35E+02	1.03E+09	1.22E+09	1.49E+10	5.26E+08	2.44E+02	2.29E+09
	Std. dev	9.00E−01	1.03E+00	1.07E+08	1.56E+08	9.55E+08	6.44E+07	6.17E−01	2.74E+08
	Max	2.46E+02	2.37E+02	1.21E+09	1.78E+09	1.68E+10	6.71E+08	2.45E+02	2.83E+09

Significant values are in [bold].

**Table 11:** Wilcoxon rank test results at 5% significance level for the competitive algorithms.

	HMRFO	MRFO	MVO	PSO	SINECOS	JAYA	EQUIL	MOTH
F_1_	N/A	1.00	< 0.05	< 0.05	< 0.05	< 0.05	< 0.05	< 0.05
	1	1(=)	6(+)	5(+)	8(+)	4(+)	3(+)	7(+)
F_2_	N/A	1.00	< 0.05	< 0.05	< 0.05	< 0.05	< 0.05	< 0.05
	1	1(=)	5(+)	6(+)	7(+)	4(+)	3(+)	7(+)
F_3_	N/A	1.00	< 0.05	< 0.05	< 0.05	< 0.05	< 0.05	< 0.05
	1	1(=)	7(+)	5(+)	8(+)	4(+)	3(+)	7(+)
F_4_	N/A	< 0.05	< 0.05	< 0.05	< 0.05	< 0.05	< 0.05	< 0.05
	1	2(+)	5(+)	7(+)	8(+)	6(+)	3(+)	4(+)
F_5_	N/A	< 0.05	< 0.05	< 0.05	< 0.05	< 0.05	< 0.05	< 0.05
	1	2(+)	7(+)	5(+)	8(+)	4(+)	3(+)	6(+)
F_6_	N/A	< 0.05	< 0.05	< 0.05	< 0.05	< 0.05	< 0.05	< 0.05
	1	2(+)	6(+)	4(+)	8(+)	5(+)	3(+)	7(+)
F_7_	N/A	1.00	< 0.05	< 0.05	< 0.05	< 0.05	< 0.05	< 0.05
	1	1(=)	6(+)	5(+)	8(+)	4(+)	3(+)	7(+)
F_8_	N/A	1.00	< 0.05	< 0.05	< 0.05	< 0.05	< 0.05	< 0.05
	1	1(=)	3(+)	6(+)	8(+)	7(+)	5(+)	4(+)
F_9_	N/A	< 0.05	< 0.05	< 0.05	< 0.05	< 0.05	< 0.05	< 0.05
	1	2(+)	7(+)	5(+)	8(+)	4(+)	3(+)	6(+)
F_10_	N/A	< 0.05	< 0.05	< 0.05	< 0.05	< 0.05	< 0.05	< 0.05
	1	2(+)	5(+)	6(+)	8(+)	4(+)	3(+)	7(+)
F_11_	N/A	1.00	< 0.05	< 0.05	< 0.05	< 0.05	< 0.05	< 0.05
	1	1(=)	6(+)	7(+)	3(+)	8(+)	4(+)	5(+)
F_12_	N/A	1.00	< 0.05	< 0.05	< 0.05	< 0.05	< 0.05	< 0.05
	1	1(=)	6(+)	7(+)	3(+)	8(+)	4(+)	5(+)
F_13_	N/A	< 0.05	< 0.05	< 0.05	< 0.05	< 0.05	< 0.05	< 0.05
	2	1(−)	5(+)	6(+)	7(+)	8(+)	3(+)	4(+)
F_14_	N/A	< 0.05	< 0.05	< 0.05	< 0.05	< 0.05	< 0.05	< 0.05
	2	1(−)	5(+)	6(+)	7(+)	8(+)	3(+)	4(+)
F_15_	N/A	1.00	< 0.05	< 0.05	< 0.05	< 0.05	< 0.05	< 0.05
	1	1(=)	4(+)	6(+)	8(+)	7(+)	5(+)	3(+)
F_16_	N/A	< 0.05	< 0.05	< 0.05	< 0.05	< 0.05	< 0.05	< 0.05
	1	2(+)	5(+)	6(+)	8(+)	4(+)	3(+)	7(+)
F_17_	N/A	< 0.05	< 0.05	< 0.05	< 0.05	< 0.05	< 0.05	< 0.05
	1	2(+)	6(+)	5(+)	8(+)	4(+)	3(+)	7(+)
F_18_	N/A	0.124	< 0.05	< 0.05	< 0.05	< 0.05	< 0.05	< 0.05
	1	2(+)	3(+)	7(+)	8(+)	4(+)	6(+)	5(+)
F_19_	N/A	< 0.05	< 0.05	< 0.05	< 0.05	< 0.05	< 0.05	< 0.05
	2	3(+)	4(+)	6(+)	8(+)	1(−)	5(+)	7(+)
F_20_	N/A	< 0.05	< 0.05	< 0.05	< 0.05	< 0.05	< 0.05	< 0.05
	2(+)	3(+)	5(+)	6(+)	8(+)	1(−)	4(+)	7(+)
F_21_	N/A	< 0.05	< 0.05	< 0.05	< 0.05	< 0.05	< 0.05	< 0.05
	1	2(+)	4(+)	6(+)	7(+)	5(+)	3(+)	7(+)
F_22_	N/A	< 0.05	< 0.05	< 0.05	< 0.05	< 0.05	< 0.05	< 0.05
	1	2(+)	4(+)	5(+)	8(+)	7(+)	3(+)	6(+)
F_23_	N/A	< 0.05	< 0.05	< 0.05	< 0.05	< 0.05	< 0.05	< 0.05
	5	4(−)	2(−)	6(+)	8(+)	7(+)	3(−)	1(−)
F_24_	N/A	< 0.05	< 0.05	< 0.05	< 0.05	< 0.05	< 0.05	< 0.05
	2	3(+)	5(+)	6(+)	8(+)	1(−)	4(+)	7(+)
F_25_	N/A	0.183	< 0.05	0.781	< 0.05	< 0.05	0.082	< 0.05
	4	6(+)	2(−)	5(+)	8(+)	7(+)	3(−)	1(−)
F_26_	N/A	< 0.05	< 0.05	< 0.05	< 0.05	< 0.05	< 0.05	< 0.05
	1	2(+)	4(+)	5(+)	8(+)	6(+)	3(+)	7(+)
F_27_	N/A	< 0.05	< 0.05	< 0.05	< 0.05	< 0.05	< 0.05	< 0.05
	1	2(+)	6(+)	5(+)	8(+)	4(+)	3(+)	7(+)
F_28_	N/A	< 0.05	< 0.05	< 0.05	< 0.05	< 0.05	< 0.05	< 0.05
	1	2(+)	6(+)	5(+)	8(+)	4(+)	3(+)	7(+)
F_29_	N/A	0.093	< 0.05	< 0.05	< 0.05	< 0.05	< 0.05	< 0.05
	1	2(+)	3(+)	7(+)	8(+)	4(+)	6(+)	5(+)
F_30_	N/A	0.093	< 0.05	< 0.05	< 0.05	< 0.05	< 0.05	< 0.05
	2(+)	1(−)	7(+)	5(+)	8(+)	4(+)	3(+)	6(+)
F_31_	N/A	< 0.05	< 0.05	< 0.05	< 0.05	< 0.05	< 0.05	< 0.05
	2(+)	4(+)	8(+)	6(+)	1(−)	5(+)	3(+)	7(+)
F_32_	N/A	< 0.05	< 0.05	< 0.05	< 0.05	< 0.05	< 0.05	< 0.05
	6	2(−)	3(−)	5(−)	7(+)	8(+)	4(−)	1(−)
F_33_	N/A	0.544	< 0.05	< 0.05	< 0.05	< 0.05	< 0.05	< 0.05
	1	2(+)	5(+)	6(+)	8(+)	7(+)	4(+)	3(+)
F_34_	N/A	< 0.05	< 0.05	< 0.05	< 0.05	< 0.05	< 0.05	< 0.05
	1	2(+)	5(+)	7(+)	3(+)	8(+)	6(+)	4(+)
F_35_	N/A	< 0.05	< 0.05	0.532	< 0.05	< 0.05	< 0.05	< 0.05
	5	1(−)	3(−)	6(+)	7(+)	8(+)	4(−)	2(−)
F_36_	N/A	0.500	< 0.05	0.532	< 0.05	< 0.05	< 0.05	< 0.05
	3	2(−)	6(+)	5(+)	8(+)	4(+)	1(−)	7(+)
F_37_	N/A	1.00	< 0.05	0.532	< 0.05	< 0.05	< 0.05	< 0.05
	1	2(+)	7(+)	4(+)	6(+)	8(+)	3(+)	5(+)
F_38_	N/A	< 0.05	< 0.05	0.532	< 0.05	< 0.05	< 0.05	0.308
	2	1(−)	7(+)	5(+)	6(+)	8(+)	4(+)	3(+)
F_39_	N/A	< 0.05	< 0.05	0.532	< 0.05	< 0.05	< 0.05	0.349
	1	4(+)	5(+)	6(+)	7(+)	8(+)	3(+)	2(+)
F_40_	N/A	< 0.05	< 0.05	0.532	< 0.05	< 0.05	< 0.05	< 0.05
	1	2(+)	8(+)	5(+)	4(+)	7(+)	3(+)	6(+)
F_41_	N/A	N/A	N/A	N/A	N/A	N/A	N/A	N/A
	1	1(=)	1(=)	1(=)	1(=)	1(=)	1(=)	1(=)
F_42_	N/A	N/A	N/A	N/A	N/A	N/A	N/A	N/A
	1	1(=)	1(=)	1(=)	1(=)	1(=)	1(=)	1(=)
F_43_	N/A	N/A	N/A	N/A	N/A	N/A	N/A	N/A
	1	1(=)	1(=)	1(=)	1(=)	1(=)	1(=)	1(=)
F_44_	N/A	< 0.05	< 0.05	< 0.05	< 0.05	< 0.05	< 0.05	< 0.05
	1	2(+)	5(+)	4(+)	8(+)	7(+)	3(+)	6(+)
F_45_	N/A	< 0.05	< 0.05	< 0.05	< 0.05	< 0.05	< 0.05	< 0.05
	1	2(+)	7(+)	3(+)	8(+)	5(+)	4(+)	6(+)
F_46_	N/A	N/A	N/A	N/A	N/A	N/A	N/A	N/A
	1	1(=)	1(=)	1(=)	1(=)	1(=)	1(=)	1(=)
F_47_	N/A	< 0.05	< 0.05	< 0.05	< 0.05	< 0.05	< 0.05	< 0.05
	2	1(−)	4(+)	5(+)	8(+)	7(+)	6(+)	3(+)
F_48_	N/A	< 0.05	0.931	< 0.05	< 0.05	< 0.05	< 0.05	< 0.05
	1	3(+)	2(+)	5(+)	8(+)	7(+)	4(+)	6(+)
F_49_	N/A	0.221	< 0.05	0.213	< 0.05	< 0.05	< 0.05	< 0.05
	3	4(+)	7(+)	2(−)	8(+)	6(+)	5(+)	1(−)
F_50_	N/A	< 0.05	< 0.05	< 0.05	< 0.05	< 0.05	< 0.05	< 0.05
	1	2(+)	5(+)	6(+)	7(+)	4(+)	3(+)	7(+)
F_51_	N/A	< 0.05	< 0.05	< 0.05	< 0.05	< 0.05	< 0.05	< 0.05
	1	2(+)	5(+)	6(+)	8(+)	4(+)	3(+)	7(+)
F_52_	N/A	< 0.05	< 0.05	< 0.05	< 0.05	< 0.05	< 0.05	< 0.05
	1	2(+)	6(+)	4(+)	8(+)	3(+)	5(+)	7(+)
F_53_	N/A	< 0.05	< 0.05	< 0.05	< 0.05	< 0.05	< 0.05	< 0.05
	1	2(+)	4(+)	7(+)	6(+)	8(+)	3(+)	5(+)
F_54_	N/A	1.00	< 0.05	< 0.05	< 0.05	< 0.05	1.00	< 0.05
	1	1(=)	7(+)	6(+)	4(+)	5(+)	1(=)	8(+)
F_55_	N/A	1.00	< 0.05	< 0.05	< 0.05	< 0.05	< 0.05	< 0.05
	1	2(+)	5(+)	6(+)	8(+)	4(+)	3(+)	7(+)
F_56_	N/A	< 0.05	< 0.05	< 0.05	< 0.05	< 0.05	< 0.05	< 0.05
	2	3(+)	6(+)	4(+)	1(−)	6(+)	8(+)	7(+)
F_57_	N/A	< 0.05	< 0.05	< 0.05	< 0.05	< 0.05	< 0.05	< 0.05
	2	3(+)	8(+)	5(+)	1(−)	7(+)	4(+)	6(+)
F_58_	N/A	< 0.05	< 0.05	< 0.05	< 0.05	< 0.05	< 0.05	< 0.05
	2	3(+)	8(+)	5(+)	1(−)	4(+)	6(+)	7(+)
F_59_	N/A	< 0.05	< 0.05	< 0.05	< 0.05	< 0.05	< 0.05	< 0.05
	1	1(=)	5(+)	6(+)	8(+)	4(+)	3(+)	7(+)
F_60_	N/A	< 0.05	< 0.05	< 0.05	< 0.05	< 0.05	< 0.05	< 0.05
	4	1(−)	3(−)	5(+)	8(+)	7(+)	6(+)	2(−)
F_61_	N/A	0.328	0.314	0.416	< 0.05	< 0.05	< 0.05	0.057
	2	3(+)	5(+)	4(+)	6(+)	8(+)	7(+)	1(−)
F_62_	N/A	< 0.05	< 0.05	< 0.05	< 0.05	< 0.05	< 0.05	< 0.05
	2	1(−)	5(+)	8(+)	4(+)	6(+)	3(+)	7(+)
F_63_	N/A	0.557	< 0.05	< 0.05	< 0.05	< 0.05	< 0.05	< 0.05
	2	1(−)	5(+)	6(+)	8(+)	4(+)	3(+)	7(+)
F_64_	N/A	1.00	< 0.05	< 0.05	< 0.05	< 0.05	< 0.05	< 0.05
	1	2(+)	8(+)	6(+)	3(+)	5(+)	4(+)	7(+)
F_65_	N/A	< 0.05	< 0.05	< 0.05	< 0.05	< 0.05	< 0.05	< 0.05
	1	2(+)	6(+)	5(+)	8(+)	4(+)	3(+)	7(+)
F_66_	N/A	< 0.05	< 0.05	< 0.05	< 0.05	< 0.05	< 0.05	< 0.05
	1	2(+)	5(+)	6(+)	8(+)	4(+)	3(+)	7(+)
F_67_	N/A	< 0.05	< 0.05	< 0.05	< 0.05	< 0.05	< 0.05	< 0.05
	2	3(+)	4(+)	7(+)	1(−)	8(+)	5(+)	6(+)
F_68_	N/A	< 0.05	< 0.05	< 0.05	< 0.05	< 0.05	< 0.05	< 0.05
	1	2(+)	5(+)	6(+)	8(+)	4(+)	3(+)	7(+)
F_69_	N/A	< 0.05	< 0.05	< 0.05	< 0.05	< 0.05	0.618	< 0.05
	3	1(−)	5(+)	6(+)	8(+)	4(+)	2(−)	7(+)
Aver. rank	1.57	1.95	5.11	5.31	6.44	5.22	3.59	5.37
Ranking	1	2	4	6	8	5	3	7
+/=/−		46/14/9	61/4/4	64/3/2	60/4/5	61/4/4	59/5/5	60/4/5

**Table 12:** Comparison of the metaheuristic algorithms based on 500D unimodal test problems.

		HMRFO	MRFO	MVO	PSO	SINECOS	JAYA	EQUIL	MOTH
F_84_	Min	**9.85E+01**	2.32E+02	1.41E+12	1.65E+12	6.82E+12	3.92E+11	5.54E+04	2.72E+12
	Mean	2.07E+02	2.98E+02	1.64E+12	2.01E+12	9.06E+12	4.90E+11	2.70E+05	2.97E+12
	Std. dev	3.95E+01	2.09E+01	1.04E+11	1.61E+11	7.53E+11	5.99E+10	1.58E+05	1.73E+11
	Max	2.69E+02	3.36E+02	1.91E+12	2.47E+12	9.71E+12	6.14E+11	7.21E+05	3.50E+12
F_85_	Min	**4.95E+02**	4.97E+02	3.27E+08	3.16E+08	5.50E+09	3.88E+07	4.98E+02	7.02E+08
	Mean	4.97E+02	4.97E+02	4.70E+08	3.95E+08	6.18E+09	7.77E+07	4.99E+02	8.99E+08
	Std. dev	3.69E−01	2.58E−01	8.27E+07	5.64E+07	2.69E+08	2.48E+07	4.79E−01	1.37E+08
	Max	4.97E+02	4.97E+02	7.16E+08	5.15E+08	6.62E+09	1.33E+08	5.00E+02	1.28E+09
F_86_	Min	2.21E+04	1.26E+03	4.54E+03	5.62E+03	2.21E+04	1.26E+03	**8.16E−01**	8.56E+03
	Mean	2.95E+04	1.55E+03	5.13E+03	6.35E+03	2.95E+04	1.55E+03	1.50E+00	9.42E+03
	Std. dev	1.64E+03	1.80E+02	2.59E+02	3.91E+02	1.64E+03	1.80E+02	3.47E−01	5.02E+02
	Max	3.11E+04	1.97E+03	5.77E+03	7.22E+03	3.11E+04	1.97E+03	2.00E+00	1.03E+04
F_87_	Min	**0.00E+00**	1.05E−168	5.76E+02	5.95E+02	2.96E+03	1.96E+02	3.28E−05	8.69E+02
	Mean	1.26E−253	1.31E−147	6.98E+02	6.91E+02	3.23E+03	2.22E+02	7.89E−05	1.01E+03
	Std. dev	0.00E+00	7.09E−147	5.61E+01	4.17E+01	9.34E+01	2.11E+01	3.24E−05	5.38E+01
	Max	3.78E−252	3.88E−146	7.95E+02	7.84E+02	3.38E+03	2.64E+02	1.56E−04	1.13E+03
F_88_	Min	**6.40E+01**	6.69E+01	4.49E+02	2.94E+02	1.42E+03	1.50E+02	3.06E+02	4.23E+02
	Mean	8.00E+01	8.93E+01	4.94E+02	3.28E+02	1.55E+03	1.77E+02	3.45E+02	4.75E+02
	Std. dev	1.09E+01	9.41E+00	3.12E+01	1.72E+01	5.32E+01	1.32E+01	2.16E+01	2.76E+01
	Max	1.10E+02	1.08E+02	6.03E+02	3.63E+02	1.66E+03	2.06E+02	3.85E+02	5.25E+02
F_89_	Min	**5.19E+00**	3.45E+03	1.65E+04	2.26E+04	8.03E+03	6.38E+03	6.82E+03	2.01E+04
	Mean	4.99E+02	4.12E+03	1.88E+04	2.43E+04	9.58E+03	7.13E+03	6.95E+03	2.23E+04
	Std. dev	1.24E+03	3.82E+02	1.08E+03	8.78E+02	1.21E+03	2.80E+02	6.64E+01	1.08E+03
	Max	4.35E+03	4.80E+03	2.06E+04	2.66E+04	1.20E+04	7.61E+03	7.07E+03	2.44E+04
F_90_	Min	**1.42E+01**	2.05E+01	4.17E+02	3.54E+02	3.00E+04	7.55E+01	2.12E+02	3.92E+02
	Mean	2.09E+01	2.58E+01	7.50E+02	4.82E+02	5.28E+04	9.49E+01	3.35E+02	6.51E+02
	Std. dev	2.92E+00	3.37E+00	2.35E+02	9.76E+01	1.35E+04	1.30E+01	7.19E+01	1.25E+02
	Max	2.68E+01	3.15E+01	1.63E+03	6.61E+02	8.29E+04	1.22E+02	5.07E+02	8.82E+02
F_91_	Min	4.32E−03	**4.28E−03**	3.24E+02	3.62E+02	5.83E+02	3.42E+02	3.68E+00	3.73E+02
	Mean	4.63E−03	4.60E−03	3.45E+02	3.91E+02	6.11E+02	3.59E+02	7.16E+00	3.98E+02
	Std. dev	3.97E−04	3.47E−04	1.33E+01	1.22E+01	1.10E+01	1.00E+01	2.59E+00	1.36E+01
	Max	6.40E−03	5.54E−03	3.76E+02	4.19E+02	6.32E+02	3.88E+02	1.36E+01	4.21E+02
F_92_	Min	**3.56E+02**	3.81E+02	2.82E+03	3.25E+03	2.20E+04	5.58E+02	4.40E+02	5.15E+03
	Mean	3.74E+02	4.04E+02	3.73E+03	4.02E+03	2.49E+04	7.46E+02	4.49E+02	5.89E+03
	Std. dev	9.38E+00	9.89E+00	4.18E+02	3.74E+02	1.24E+03	1.01E+02	4.26E+00	4.36E+02
	Max	3.89E+02	4.23E+02	4.64E+03	4.81E+03	2.73E+04	9.42E+02	4.56E+02	6.72E+03
F_93_	Min	**1.69E−292**	3.88E−166	3.73E+05	2.23E+05	7.93E+04	9.44E+04	3.48E−02	3.16E+05
	Mean	8.77E−240	3.36E−145	4.13E+05	2.67E+05	4.76E+05	1.11E+05	1.34E−01	3.38E+05
	Std. dev	0.00E+00	1.31E−144	2.40E+04	1.87E+04	2.56E+05	1.00E+04	8.30E−02	1.60E+04
	Max	2.61E−238	6.81E−144	4.55E+05	3.00E+06	9.05E+05	1.38E+05	3.88E−01	3.79E+05
F_94_	Min	**0.00E+00**	2.33E−321	8.28E+08	8.46E+08	8.40E+09	2.25E+08	2.67E−04	1.70E+09
	Mean	0.00E+00	1.82E−274	1.06E+09	1.05E+09	8.90E+09	2.97E+08	4.36E−03	2.93E+09
	Std. dev	0.00E+00	0.00E+00	1.15E+08	1.25E+08	2.42E+08	4.78E+07	4.59E−03	1.30E+08
	Max	0.00E+00	5.47E−273	1.38E+09	1.35E+09	9.25E+09	4.50E+08	2.22E−02	2.34E+09
F_95_	Min	**5.21E−302**	9.34E−161	6.43E+07	7.67E+07	1.51E+08	3.71E+07	2.54E+00	1.18E+08
	Mean	1.95E−259	7.87E−147	7.23E+07	8.84E+07	3.45E+08	4.81E+07	8.71E+00	1.32E+08
	Std. dev	0.00E+00	4.04E−146	3.86E+06	4.65E+06	6.25E+07	4.80E+06	5.38E+00	6.57E+06
	Max	4.20E−258	2.21E−145	8.08E+07	9.80E+07	3.98E+08	5.74E+07	2.33E+01	1.45E+08
F_96_	Min	**− 1.77E+03**	− 4.99E+00	4.76E+07	6.71E+07	5.40E+08	1.93E+07	− 4.99E+00	1.61E+08
	Mean	− 4.48E+02	− 4.95E+00	7.74E+07	9.27E+07	1.35E+09	2.46E+07	− 2.05E+00	2.01E+08
	Std. dev	4.80E+02	2.69E−02	1.19E+07	1.28E+07	2.85E+08	3.90E+06	2.28E+00	1.82E+07
	Max	− 5.18E+01	− 4.89E+00	9.85E+07	1.19E+08	1.56E+09	3.52E+07	9.77E−07	2.42E+08
F_97_	Min	9.21E−01	1.05E+00	9.56E−01	1.10E+00	4.19E+00	1.32E+00	1.03E+00	**8.87E−01**
	Mean	1.36E+00	1.34E+00	1.12E+00	1.38E+00	4.36E+00	1.63E+00	1.23E+00	9.89E−01
	Std. dev	1.49E−01	9.57E−02	8.36E−02	1.21E−01	8.48E−02	2.13E−01	9.03E−02	4.32E−02
	Max	1.67E+00	1.60E+00	1.30E+00	1.62E+00	4.58E+00	2.13E+00	1.36E+00	1.06E+00
F_98_	Min	**9.59E+13**	1.28E+14	1.25E+14	1.35E+14	1.43E+14	1.22E+14	1.30E+14	1.29E+14
	Mean	1.04E+14	1.31E+14	1.28E+14	1.37E+14	1.39E+14	1.24E+14	1.32E+14	1.32E+14
	Std. dev	3.68E+12	1.99E+12	1.94E+12	1.65E+12	1.85E+12	9.43E+11	1.23E+12	1.56E+12
	Max	1.10E+14	1.35E+14	1.32E+14	1.40E+14	1.51E+14	1.26E+14	1.35E+14	1.36E+14
F_99_	Min	**3.06E−305**	4.81E−167	4.64E+04	8.84E+05	2.80E+08	1.01E+03	1.82E−02	5.71E+05
	Mean	2.97E−253	2.55E−146	2.02E+05	4.38E+06	1.56E+10	5.08E+04	1.26E−01	5.00E+06
	Std. dev	0.00E+00	1.31E−145	1.03E+05	2.61E+06	1.28E+10	8.23E+04	9.88E−02	3.04E+06
	Max	8.91E+252	7.19E−145	4.52E+05	1.23E+07	5.28E+10	4.24E+05	3.85E−01	1.27E+07
F_100_	Min	**5.23E−01**	6.20E−01	4.51E+05	5.37E+05	1.48E+06	1.30E+05	1.56E+00	8.66E+05
	Mean	7.09E−01	8.72E−01	5.16E+05	6.40E+05	2.85E+06	1.54E+05	1.86E+00	9.51E+05
	Std. dev	1.92E−01	2.05E−01	3.67E+04	4.86E+04	3.75E+05	1.60E+04	2.19E−01	5.77E+04
	Max	1.25E+00	1.35E+00	6.12E+05	7.53E+05	3.15E+06	1.95E+05	2.16E+00	1.05E+06

Significant values are in [bold].

**Table 13:** Wilcoxon sum rank test results for 500D unimodal problems.

	HMRFO	MRFO	MVO	PSO	SINECOS	JAYA	EQUIL	MOTH
F_70_	N/A	< 0.05	< 0.05	< 0.05	< 0.05	< 0.05	< 0.05	< 0.05
	1	2(+)	6(+)	5(+)	8(+)	4(+)	3(+)	7(+)
F_71_	N/A	< 0.05	< 0.05	< 0.05	< 0.05	< 0.05	< 0.05	< 0.05
	1	2(+)	5(+)	6(+)	8(+)	4(+)	3(+)	7(+)
F_72_	N/A	< 0.05	< 0.05	< 0.05	< 0.05	< 0.05	< 0.05	< 0.05
	1	2(+)	5(+)	6(+)	8(+)	4(+)	3(+)	7(+)
F_73_	N/A	< 0.05	< 0.05	< 0.05	< 0.05	< 0.05	< 0.05	< 0.05
	1	2(+)	8(+)	5(+)	4(+)	6(+)	3(+)	7(+)
F_74_	N/A	< 0.05	< 0.05	< 0.05	< 0.05	< 0.05	< 0.05	< 0.05
	1	2(+)	4(+)	5(+)	6(+)	7(+)	3(+)	8(+)
F_75_	N/A	< 0.05	< 0.05	< 0.05	< 0.05	< 0.05	< 0.05	< 0.05
	1	2(+)	4(+)	5(+)	6(+)	7(+)	3(+)	8(+)
F_76_	N/A	< 0.05	< 0.05	< 0.05	< 0.05	< 0.05	< 0.05	< 0.05
	1	2(+)	5(+)	6(+)	8(+)	4(+)	3(+)	7(+)
F_77_	N/A	< 0.05	< 0.05	< 0.05	< 0.05	< 0.05	< 0.05	< 0.05
	1	2(+)	5(+)	6(+)	8(+)	4(+)	3(+)	7(+)
F_78_	N/A	< 0.05	< 0.05	< 0.05	< 0.05	< 0.05	< 0.05	< 0.05
	1	2(+)	7(+)	5(+)	8(+)	4(+)	3(+)	6(+)
F_79_	N/A	< 0.05	< 0.05	< 0.05	< 0.05	< 0.05	< 0.05	< 0.05
	1	2(+)	7(+)	6(+)	4(+)	5(+)	3(+)	8(+)
F_80_	N/A	< 0.05	< 0.05	< 0.05	< 0.05	< 0.05	< 0.05	< 0.05
	1	2(+)	6(+)	5(+)	8(+)	4(+)	3(+)	7(+)
F_81_	N/A	< 0.05	< 0.05	< 0.05	< 0.05	< 0.05	< 0.05	< 0.05
	1	2(+)	5(+)	6(+)	8(+)	4(+)	3(+)	7(+)
F_82_	N/A	< 0.05	< 0.05	< 0.05	< 0.05	< 0.05	< 0.05	< 0.05
	1	2(+)	6(+)	5(+)	8(+)	4(+)	3(+)	7(+)
F_83_	N/A	1.00	< 0.05	< 0.05	< 0.05	< 0.05	< 0.05	< 0.05
	1	1(=)	7(+)	5(+)	8(+)	4(+)	3(+)	6(+)
F_84_	N/A	< 0.05	< 0.05	< 0.05	< 0.05	< 0.05	< 0.05	< 0.05
	1	2(+)	5(+)	6(+)	8(+)	4(+)	3(+)	7(+)
F_85_	N/A	< 0.05	< 0.05	< 0.05	< 0.05	< 0.05	< 0.05	< 0.05
	1	2(+)	6(+)	5(+)	8(+)	4(+)	3(+)	7(+)
F_86_	N/A	< 0.05	< 0.05	< 0.05	< 0.05	< 0.05	< 0.05	< 0.05
	1	2(+)	5(+)	6(+)	8(+)	4(+)	3(+)	7(+)
F_87_	N/A	< 0.05	< 0.05	< 0.05	< 0.05	< 0.05	< 0.05	< 0.05
	1	2(+)	6(+)	5(+)	8(+)	4(+)	3(+)	7(+)
F_88_	N/A	< 0.05	< 0.05	< 0.05	< 0.05	< 0.05	< 0.05	< 0.05
	1	2(+)	7(+)	4(+)	8(+)	3(+)	5(+)	6(+)
F_89_	N/A	< 0.05	< 0.05	< 0.05	< 0.05	< 0.05	< 0.05	< 0.05
	1	2(+)	6(+)	8(+)	5(+)	4(+)	3(+)	7(+)
F_90_	N/A	< 0.05	< 0.05	< 0.05	< 0.05	< 0.05	< 0.05	< 0.05
	1	2(+)	7(+)	5(+)	8(+)	3(+)	4(+)	6(+)
F_91_	N/A	< 0.05	< 0.05	< 0.05	< 0.05	< 0.05	< 0.05	< 0.05
	2	1(−)	4(+)	6(+)	8(+)	5(+)	3(+)	7(+)
F_92_	N/A	< 0.05	< 0.05	< 0.05	< 0.05	< 0.05	< 0.05	< 0.05
	1	2(+)	5(+)	6(+)	8(+)	4(+)	3(+)	7(+)
F_93_	N/A	< 0.05	< 0.05	< 0.05	< 0.05	< 0.05	< 0.05	< 0.05
	1	2(+)	7(+)	5(+)	8(+)	4(+)	3(+)	6(+)
F_94_	N/A	< 0.05	< 0.05	< 0.05	< 0.05	< 0.05	< 0.05	< 0.05
	1	2(+)	6(+)	5(+)	8(+)	4(+)	3(+)	7(+)
F_95_	N/A	< 0.05	< 0.05	< 0.05	< 0.05	< 0.05	< 0.05	< 0.05
	1	2(+)	5(+)	6(+)	8(+)	4(+)	3(+)	7(+)
F_96_	N/A	< 0.05	< 0.05	< 0.05	< 0.05	< 0.05	< 0.05	< 0.05
	1	2(+)	5(+)	6(+)	8(+)	4(+)	3(+)	7(+)
F_97_	N/A	0.658	< 0.05	0.498	< 0.05	< 0.05	< 0.05	< 0.05
	5	4(−)	2(−)	6(+)	8(+)	7(+)	3(−)	1(−)
F_98_	N/A	< 0.05	< 0.05	< 0.05	< 0.05	< 0.05	< 0.05	< 0.05
	1	4(+)	3(+)	7(+)	8(+)	2(+)	5(+)	6(+)
F_99_	N/A	< 0.05	< 0.05	< 0.05	< 0.05	< 0.05	< 0.05	< 0.05
	1	2(+)	5(+)	6(+)	8(+)	4(+)	3(+)	7(+)
F_100_	N/A	< 0.05	< 0.05	< 0.05	< 0.05	< 0.05	< 0.05	< 0.05
	1	2(+)	5(+)	6(+)	8(+)	4(+)	3(+)	7(+)
Aver. rank	1.15	2.06	5.43	5.62	7.53	4.28	3.15	6.71
Ranking	1	2	5	6	8	4	3	7
+/=/−		28/1/2	30/0/1	31/0/0	31/0/0	31/0/0	30/0/1	30/0/1

### Experiment case—3: comparative analysis between the proposed method and opposition-based MRFO variants

**Fig. 17:**
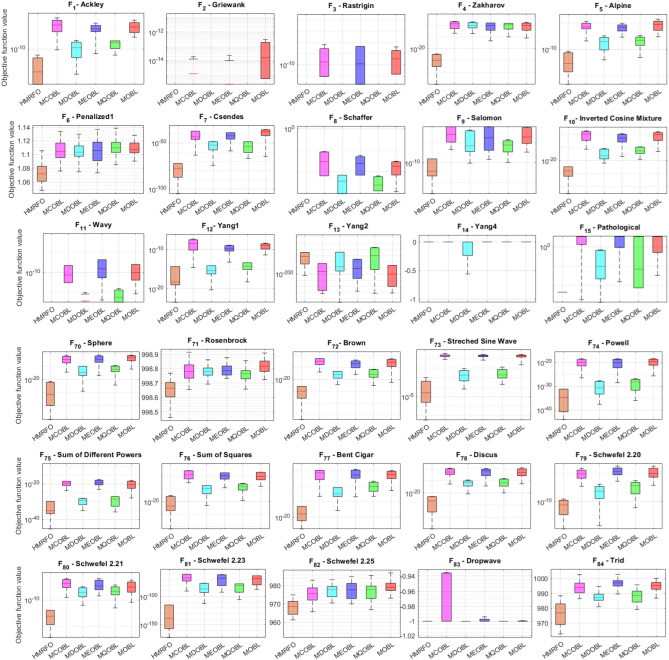
Statistical comparison between different oppositional-based MRFO algorithms and the proposed HMRFO in terms of box plot representation.

**Fig. 18:**
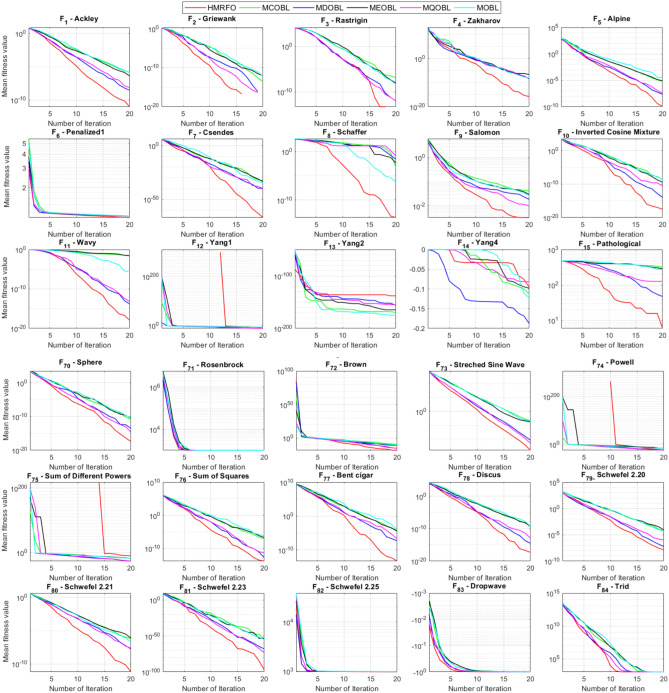
Convergence curves drawn for 1000D multidimensional and unimodal test problems.

This section briefly analyzes HMRFO and various oppositional-based Manta-Ray Foraging Optimization algorithms concerning the first fifteen 1000D multimodal and unimodal test problems provided in Tables 4 and 5. Oppositional-based algorithms significantly enhance the solution quality of the base metaheuristic algorithm in which they are embedded, thanks to the expanded search space from ubiquitously utilizing the current and opposite solutions to approach fertile areas in the solution domain. Although there are many oppositional-based learning strategies previously proposed in the current literature, this study considers these five methods of standard Oppositional-based Learning (OBL)^53^, Central Oppositional-based Learning (COBL)^54^, Quasi Oppositional-based Learning (QOBL)^55^, Elite Oppositional based Learning (EOBL)^32,40^, and Dynamic Oppositional based Learning (DOBL)^33^ to be integrated into standard MRFO algorithms. All algorithms are run 50 times for 20 iterations with a corresponding population size of N = 20, and respective results are evaluated regarding boxplots and convergence curves. Figure 17 visualizes the boxplot results for 1000D multimodal and unimodal test problems obtained by the competitive different opposition-based MRFO algorithms along the proposed HMRFO optimization method. It is observed that HMRFO finds the global optimum solution of f(x) = 0 in each independent run for F_2_-Griewank, F_3_-Rastrigin, F_8_-Schaffer, and F_11_—Wavy multimodal 1000D test problems. Its dominance prevails over the remaining cases as HMRFO is only outperformed for the F_13_-Yang2 function by the remaining OBL variants and becomes the superior algorithm for multimodal test problems. Convergence curves constructed for the respective performance of 1000D multimodal and unimodal problems shown in Fig. 18 also verify the success of the HMRFO in terms of quick and accurate evolution to the optimum solution. Gradual decreases in fitness values indicate the maintained balance between exploration and exploitation mechanisms occurred from the constructed hierarchy within the population that enables them to generate new offspring from the created synergy between quantum-behaved mutation, dynamic opposition-based learning, and elite opposition-based learning schemes; those three having different search tendencies and capabilities to explore various promising regions around the defined search space. When considering 1000D unimodal test functions, HMRFO continues its superior success in these benchmark cases and surpasses all remaining competitive oppositional-based MRFO algorithms concerning the best and mean predictive results despite failing to reach the global optimum solution of any unimodal test function within 400 function evaluations. Similar convergence performances are also observed for 1000D unimodal test problems to those obtained for the multimodal problems by the proposed HMRFO since it quickly reaches its optimum solution. At the same time, other oppositional-based learning variants struggle to overcome the local points scattered over the search space to arrive at the global optimum solution of the defined benchmark problem.

### Experiment case—4: statistical analysis on CEC—2013 benchmark problems

**Fig. 19:**
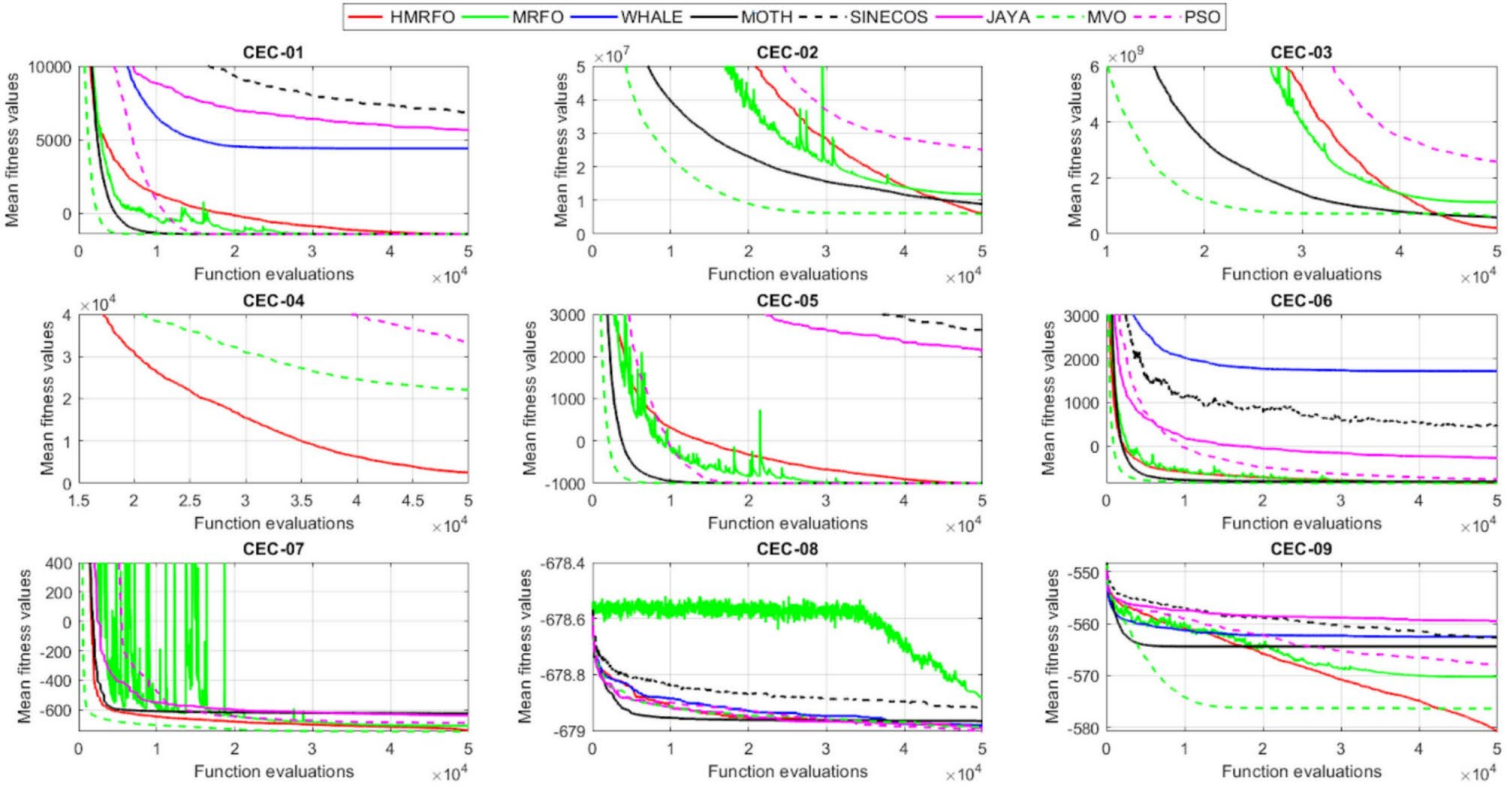
Evolution tendencies of the fitness values for the compared optimizers for benchmark functions from CEC01 to CEC09.

**Fig. 20:**
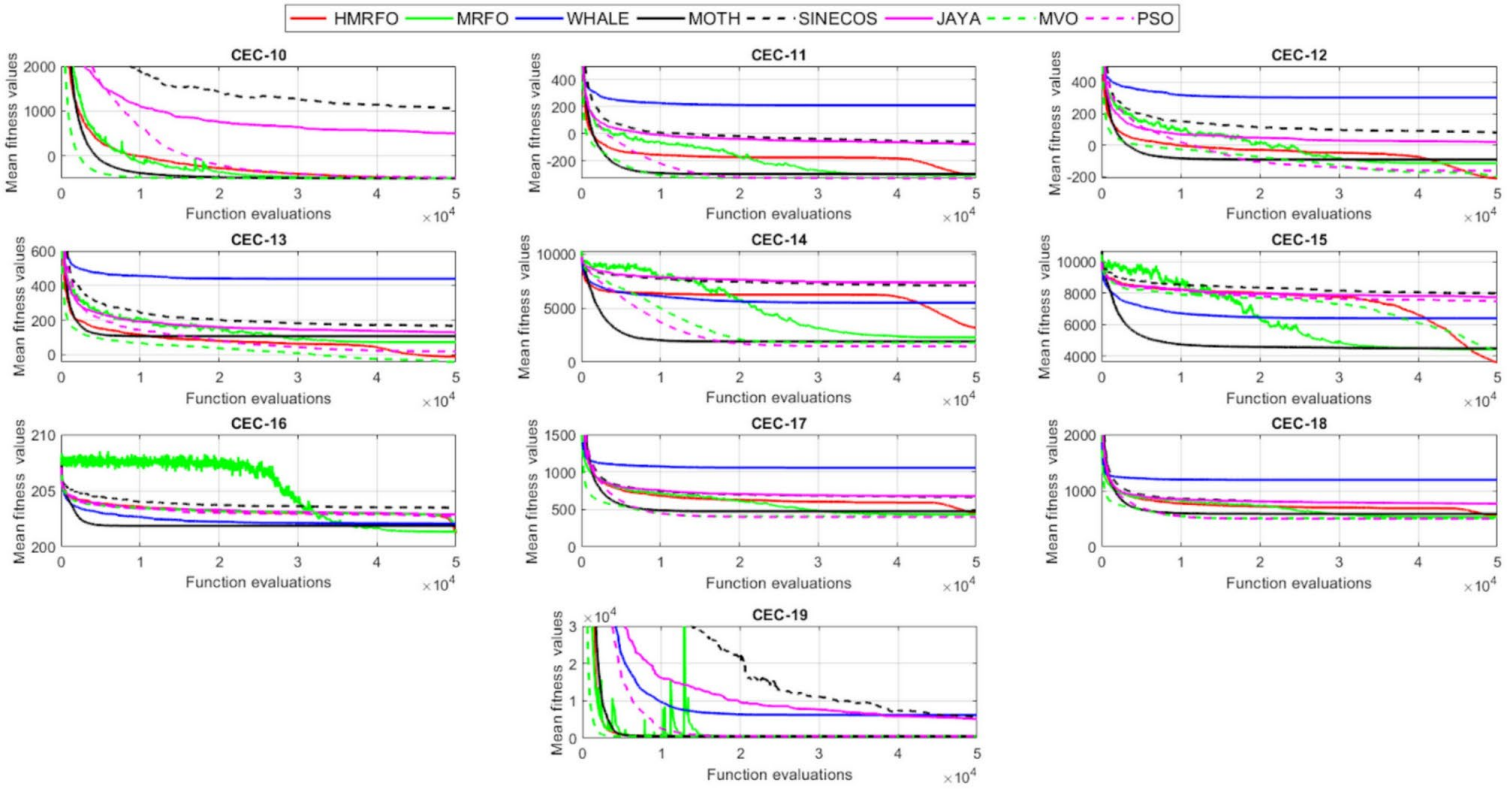
Convergence performance of the compared algorithms for multimodal benchmark problems.

**Fig. 21:**
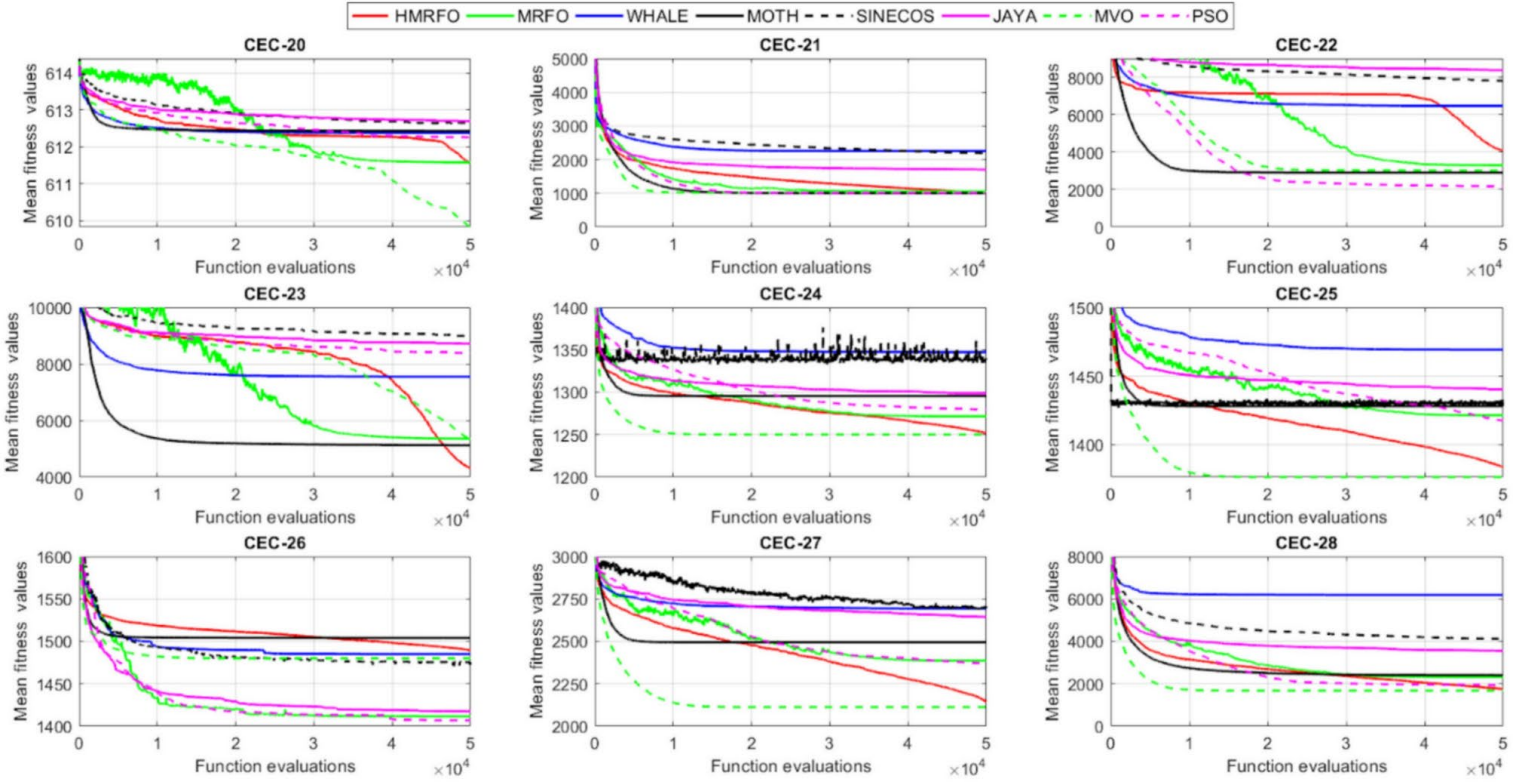
Evolution histories of the objective function values with the elapsed number of iterations for composite test functions.

This section evaluates the optimization performance of the developed HMRFO algorithm over twenty-eight artificially generated 30D test problems utilized in the CEC2013 Special Session on real parameter optimization. Respective acquired results from the HMRFO algorithm are assessed against those obtained from famous literature metaheuristic optimizers of MRFO, MOTH, SINECOS, JAYA, MVO, PSO, and WHALE using the merits of statistical analysis, considering the measures of best, mean, worst, and standard deviation results. All these algorithms are run 50 times for 50,000 function evaluations, and their optimization success is analyzed through the outcomes of the statistical analysis. These challenging benchmark problems have been frequently utilized in evaluating the developed metaheuristic algorithms’ search capabilities and are considered favorable test beds for algorithm benchmarking. Twenty-eight functions in the CEC 2013 test suite include three different types of problems, which can be categorized into unimodal functions (CEC01–CEC05), multi-modal functions (CEC06–CEC20), and composition functions (CEC21–CEC28). Table 14 reports the optimal results obtained for unimodal test functions of CEC2013 test problems for the compared algorithm, including the proposed HMRFO algorithm. When considering the best results, HMRFO outperforms all the compared methods in Table 14 except for the case CEC03, in which MOTH is the dominant method. MRFO surpasses HMRFO for the test function of CEC02, considering the mean results. However, HMRFO is the best-performing method for unimodal cases when evaluations are based on mean results. Table 15 reports the statistical analysis results of multimodal test functions employed in the CEC 2013 competition. There is no clear dominancy between the competitive algorithms for multi-modal test problems since HMRFO rules dominancy over the remaining algorithm in only five test instances when mean results are considered. The original MRFO algorithm performs better for multimodal test problems by taking the lead in eight out of fifteen benchmark cases when overall performance is evaluated in terms of mean results. Table 16 provides the statistical results for composite test functions, which integrate two or more test functions used between CEC01 and CEC20. Locating the optimal regions of this type of problem requires a prolific skill for the employed optimization algorithm as it is needed for well-established probing within the defined search space to successfully avoid plenty of sub-optimal local solutions to reach the global optimum solution of the prescribed optimization problem HMRFO gets the best mean results CEC21, CEC23, CEC24, CEC26 test problems and dominates the remaining methods concerning solution consistency and accuracy for composite test problems. Figures 19, 20, and 21 visualize the convergence histories of the compared test functions for twenty-eight CEC2013 test problems.

**Table 14:** Statistical results obtained for unimodal test problems of CEC2013 for the contestant algorithms.

	HMRFO	MRFO	WHALE	MOTH	SINECOS	JAYA	MVO	PSO
CEC01								
Min	**− 1.40E+03**	**− 1.40E+03**	− 3.78E+02	**− 1.40E+03**	4.18E+03	3.45E+03	**− 1.40E+03**	**− 1.40E+03**
Mean	− 1.40E+03	− 1.40E+03	4.41E+03	− 1.40E+03	6.79E+02	5.59E+03	− 1.40E+03	− 1.40E+03
Std. dev	2.59E−02	3.77E−10	2.68E+03	6.26E−13	1.65E+03	1.62E+03	4.95E−07	2.32E−06
Max	− 1.40E+03	− 1.40E+03	1.03E+04	− 1.40E+03	1.08E+04	9.12E+03	− 1.40E+03	− 1.40E+03
CEC02								
Min	**1.42E+06**	2.31E+06	3.77E+07	1.94E+06	7.11E+07	5.12E+07	6.00E+06	2.92E+06
Mean	6.14E+06	5.83E+06	2.67E+08	8.90E+06	1.32E+08	1.16E+08	1.18E+07	2.50E+07
Std. dev	2.91E+06	1.81E+06	1.37E+08	6.55E+06	3.50E+07	2.93E+07	4.46E+06	1.72E+07
Max	1.27E+07	1.13E+07	6.08E+08	3.38E+07	1.87E+08	1.55E+08	2.25E+07	5.66E+07
CEC03								
Min	**1.52E+07**	2.58E+07	5.34E+10	1.32E+07	3.04E+10	1.20E+10	6.62E+07	4.74E+07
Mean	2.21E+08	6.11E+08	9.67E+14	5.89E+08	1.03E+15	2.86E+10	1.13E+09	2.58E+09
Std. dev	2.74E+08	5.93E+08	3.71E+15	6.42E+08	3.71E+15	9.94E+09	9.44E+08	3.59E+09
Max	1.05E+09	2.30E+09	1.99E+16	3.04E+09	1.67E+16	5.61E+10	3.33E+09	1.71E+10
CEC04								
Min	**− 1.85E+02**	1.31E+04	5.62E+04	4.93E+04	3.88E+04	2.96E+04	4.36E+04	2.09E+04
Mean	2.52E+03	2.21E+04	6.91E+04	7.44E+04	5.76E+04	5.03E+04	5.58E+04	3.31E+04
Std. dev	1.86E+03	4.66E+03	6.34E+03	1.65E+04	1.08E+04	7.91E+03	4.31E+03	6.48E+03
Max	7.07E+03	3.11E+04	8.71E+04	1.26E+05	9.08E+04	6.90E+04	6.20E+04	4.71E+04
CEC05								
Min	**− 1.00E+03**	**− 1.00E+03**	3.75E+02	− 1.00E+03	1.28E+03	9.80E+02	**− 1.00E+03**	**− 1.00E+00**
Mean	− 1.00E+03	− 1.00E+03	3.34E+03	− 1.00E+03	2.60E+03	2.13E+03	− 9.99E+02	− 1.00E+00
Std. dev	1.91E−04	5.62E−07	2.34E+03	9.84E−08	1.06E+03	6.33E+02	2.49E+00	9.24E−07
Max	− 1.00E+03	− 1.00E+03	8.81E+03	− 1.00E+03	7.01E+03	3.4E+03	− 9.86E+02	− 1.00E+00

Significant values are in [bold].

**Table 15:** Optimal results for multimodal problems of CEC2013 test suite.

	HMRFO	MRFO	WHALE	MOTH	SINECOS	JAYA	MVO	PSO
CEC12								
Min	− 2.53E+02	− 2.41E+02	1.40E+02	− 1.96E+02	1.57E+01	**− 2.75E+01**	− 2.08E+02	− 2.17E+02
Mean	− 2.09E+02	− 1.97E+02	3.02E+02	− 9.17E+01	8.01E+01	2.10E+01	− 1.12E+02	− 1.60E+02
Std. dev	2.27E+01	2.98E+01	9.61E+01	5.86E+01	3.11E+01	2.71E+01	6.31E+01	3.55E+01
Max	− 1.62E+02	− 1.45E+02	5.13E+02	1.06E+02	1.38E+02	8.77E+01	2.93E+01	− 9.45E+01
CEC13								
Min	− 9.16E+01	**− 1.15E+02**	2.36E+02	7.42E+00	9.19E+01	8.11E+01	− 2.42E+01	− 8.08E+01
Mean	− 1.02E+01	− 4.21E+01	4.38E+02	1.07E+02	1.67E+02	1.30E+02	7.33E+01	1.89E+01
Std. dev	4.41E+01	4.16E+01	9.71E+01	5.82E+01	4.34E+01	2.90E+01	4.48E+01	3.66E+01
Max	7.61E+01	7.67E+01	6.08E+02	2.32E+02	2.76E+02	1.81E+02	1.57E+02	7.25E+01
CEC14								
Min	1.79E+03	7.76E+02	3.86E+03	9.50E+02	6.61E+03	6.64E+03	6.95E+02	**6.75E+02**
Mean	3.18E+03	1.79E+03	5.50E+03	1.91E+03	7.03E+03	7.38E+03	2.31E+03	1.44E+03
Std. dev	6.02E+02	5.62E+02	7.47E+02	5.04E+02	3.01E+02	3.27E+02	6.77E+02	3.57E+02
Max	4.53E+03	2.82E+03	6.69E+03	3.05E+03	7.97E+03	8.00E+03	3.62E+03	2.29E+03
CEC15								
Min	2.19E+03	**2.52E+03**	4.72E+03	3.10E+03	7.14E+03	6.62E+03	2.79E+03	5.82E+03
Mean	3.60E+03	4.32E+03	6.39E+03	4.47E+03	7.99E+03	7.74E+03	4.43E+03	7.47E+03
Std. dev	8.07E+02	6.89E+02	7.22E+02	7.43E+02	3.78E+02	3.74E+02	6.56E+02	4.60E+02
Max	5.27E+03	5.44E+03	7.42E+03	5.91E+03	8.86E+03	8.37E+03	5.63E+03	8.12E+03
CEC16								
Min	**2.00E+02**	2.00E+02	2.00E+02	2.00E+02	2.02E+02	2.02E+02	2.00E+02	2.01E+02
Mean	2.01E+02	2.01E+02	2.02E+02	2.01E+02	2.03E+02	2.02E+02	2.01E+01	2.02E+02
Std. dev	5.98E−02	5.45E−01	7.15E−02	1.01E+00	4.09E−01	3.31E−01	6.92E−01	4.62E−02
Max	2.02E+02	2.02E+02	2.03E+02	2.04E+02	2.04E+02	2.03E+02	2.03E+02	2.03E+02
CEC17								
Min	3.91E+02	**3.66E+02**	8.35E+02	4.16E+02	6.10E+02	6.03E+02	3.96E+02	3.71E+02
Mean	4.53E+02	4.06E+02	1.05E+03	4.74E+02	6.66E+02	6.76E+02	4.31E+02	3.98E+02
Std. dev	3.36E+01	3.61E+01	1.06E+02	4.07E+01	2.75E+01	3.33E+01	2.05E+01	1.44E+01
Max	5.26E+02	5.19E+02	1.23E+03	6.17E+02	7.25E+02	7.32E+02	4.87E+02	4.29E+02
CEC18								
Min	4.83E+02	4.63E+02	9.54E+02	5.02E+02	7.23E+02	7.18E+02	4.76E+02	**4.62E+02**
Mean	5.45E+02	5.08E+02	1.19E+03	5.87E+02	7.70E+02	7.70E+02	5.30E+02	4.99E+02
Std. dev	3.88E+01	3.03E+01	1.10E+02	5.52E+01	3.08E+01	3.59E+01	3.16E+01	1.84E+01
Max	6.05E+02	5.96E+02	1.32E+03	6.89E+02	8.51E+02	8.58E+02	6.02E+02	5.39E+02
CEC19								
Min	5.04E+02	**5.02E+02**	1.08E+03	5.07E+02	2.44E+03	1.37E+03	5.02E+02	5.05E+02
Mean	5.09E+02	5.07E+02	6.21E+03	5.38E+02	5.86E+03	5.16E+03	5.06E+02	5.11E+02
Std. dev	2.86E+00	3.89E+00	4.61E+03	7.14E+01	2.89E+03	3.22E+03	2.47E+01	4.42E+00
Max	5.15E+02	5.22E+02	1.93E+04	9.02E+02	1.52E+04	1.57E+04	5.12E+02	5.23E+02
CEC20								
Min	6.10E+02	**6.08E+02**	6.11E+02	6.10E+02	6.11E+02	6.12E+02	6.10E+02	6.11E+02
Mean	6.11E+02	6.09E+02	6.12E+02	6.12E+02	6.12E+02	6.12E+02	6.11E+02	6.12E+02
Std. dev	4.65E−01	6.04E−01	3.38E−01	6.26E−01	3.59E−01	2.35E−01	3.37E−01	3.07E−01
Max	6.12E+02	6.10E+02	6.14E+02	6.13E+02	6.13E+02	6.13E+02	6.11E+02	6.12E+02

Significant values are in [bold].

**Table 16:** Optimal results found by the competitive methods for composite functions.

	HMRFO	MRFO	WHALE	MOTH	SINECOS	JAYA	MVO	PSO
CEC21								
Min	9.04E+02	**8.00E+02**	1.22E+03	8.00E+02	1.57E+03	1.31E+03	**8.00E+02**	**8.00E+02**
Mean	1.00E+03	1.02E+03	2.25E+03	1.06E+03	2.19E+03	1.70E+03	1.05E+03	1.01E+03
Std. dev	5.71E+01	6.23E+01	4.74E+02	6.91E+01	2.15E+02	3.65E+02	6.81E+01	7.46E+01
Max	1.09E+03	1.09E+03	2.75E+03	1.09E+03	2.61E+03	2.33E+03	1.09E+03	1.09E+03
CEC22								
Min	2.80E+03	1.92E+03	4.69E+03	1.78E+03	6.96E+03	7.67E+03	1.87E+03	**1.60E+03**
Mean	4.08E+03	3.01E+03	6.46E+03	2.90E+03	7.82E+03	8.38E+03	3.30E+03	2.18E+03
Std. dev	6.00E+02	5.04E+02	6.53E+02	5.20E+02	4.94E+02	2.79E+02	7.09E+02	3.24E+02
Max	5.52E+03	4.36E+03	8.02E+03	3.87E+03	8.85E+03	8.77E+03	4.85E+03	2.90E+03
CEC23								
Min	**3.20E+03**	3.91E+03	6.30E+03	3.82E+03	7.85E+03	7.86E+03	4.24E+03	5.28E+03
Mean	4.32E+03	5.34E+03	7.55E+03	5.12E+03	9.00E+03	8.71E+03	5.35E+03	8.39E+03
Std. dev	6.98E+02	5.82E+02	7.35E+02	6.49E+02	4.21E+02	2.72E+02	6.95E+02	7.47E+02
Max	5.56E+03	6.30E+03	8.72E+03	6.36E+03	9.72E+03	9.05E+03	6.67E+03	8.99E+03
CEC24								
Min	**1.22E+03**	1.22E+03	1.30E+03	1.27E+03	1.32E+03	1.27E+03	1.25E+03	1.24E+03
Mean	1.23E+03	1.25E+03	1.34E+03	1.29E+03	1.33E+03	1.29E+03	1.27E+03	1.27E+03
Std. dev	1.32E+01	1.04E+01	2.96E+01	1.22E+01	8.41E+00	1.20E+01	1.38E+01	1.25E+01
Max	1.27E+03	1.27E+03	1.43E+03	1.33E+03	1.35E+03	1.32E+03	1.31E+03	1.30E+03
CEC25								
Min	1.36E+03	**1.32E+03**	1.42E+03	1.40E+03	1.42E+03	1.42E+03	1.39E+03	1.39E+03
Mean	1.38E+03	1.37E+03	1.46E+03	1.42E+03	1.43E+03	1.44E+03	1.42E+03	1.41E+03
Std. dev	1.15E+01	2.08E+01	2.16E+01	1.34E+01	5.90E+00	5.71E+00	1.55E+01	1.31E+00
Max	1.40E+03	1.40E+03	1.52E+03	1.45E+03	1.44E+03	1.45E+03	1.45E+03	1.44E+03
CEC26								
Min	**1.40E+03**	**1.40E+03**	1.40E+03	1.40E+03	1.40E+03	1.40E+03	1.40E+03	1.40E+03
Mean	1.41E+03	1.47E+03	1.48E+03	1.50E+03	1.47E+03	1.41E+03	1.41E+03	1.40E+03
Std. dev	7.42E+01	7.61E+01	8.26E+01	9.25E+01	8.69E+01	3.61E+01	4.25E+01	3.01E+01
Max	1.46E+03	1.56E+03	1.61E+03	1.60E+03	1.60E+03	1.60E+03	1.57E+03	1.56E+03
CEC27								
Min	1.96E+03	**1.84E+03**	2.48E+03	2.31E+03	2.45E+03	2.45E+03	2.11E+03	2.11E+03
Mean	2.10E+03	2.11E+03	2.69E+03	2.49E+03	2.70E+03	2.64E+03	2.38E+03	2.36E+03
Std. dev	8.16E+01	9.21E+01	9.61E+03	9.53E+01	1.70E+02	7.21E+02	1.40E+02	1.09E+02
Max	2.32E+03	2.29E+03	2.88E+03	2.69E+03	3.06E+03	2.74E+03	2.66E+03	2.43E+03
CEC28								
Min	1.70E+03	**1.50E+03**	5.08E+03	1.70E+03	3.36E+03	3.23E+03	1.50E+03	1.50E+03
Mean	1.74E+03	1.67E+03	6.18E+03	2.40E+03	4.09E+03	3.55E+03	2.32E+03	1.93E+03
Std. dev	2.11E+02	6.91E+01	5.56E+02	9.20E+02	4.61E+02	1.69E+02	1.06E+03	5.34E+02
Max	2.86E+03	1.70E+03	7.18E+03	4.56E+03	5.25E+03	3.99E+03	2.98E+03	3.46E+03

Significant values are in [bold].

### Solving chemical equilibrium problems through the proposed HMRFO algorithm

Here in this section, the optimization effectivity of the proposed HMRFO method is benchmarked against solving reactive chemical equilibrium problems, which are highly nonlinear and non-convex and can be considered favorable test beds to assess the efficiency of the developed HMRFO algorithm. The following paragraph briefly discusses the fundamental characteristics of the reacting chemical systems, their importance in the accurate modeling of chemical equilibrium formulations, and existing past literature devoted to utilizing the proper procedure to solve chemical equilibrium problems.

In chemical engineering, understanding a system’s phase behavior of various components is crucial in designing and simulating complex separation processes, such as reactive distillation and supercritical extraction^56^. The accuracy and reliability of phase behavior calculations significantly impact the results of simulation models characterizing complex and intricate chemical reactions. Chemical equilibrium is used to simulate the behavior of a chemical mixture at a fixed temperature (*T*), pressure (*P*), and overall composition^57^. One significant challenge that results in computational difficulties during chemical equilibrium calculations is determining the unknown quantity of reacting molecules at the equilibrium point. To effectively tackle this problem, many intelligently devised computational procedures for calculating the accurate mole numbers of the reactive components at a specific equilibrium condition are designed to search through all potential phases systematically. Optimization algorithms used for identifying the global optimum point in phase equilibrium problems can be divided into two main categories: deterministic and stochastic algorithms. Between them, stochastic-based metaheuristic algorithms have been applied to various cases of chemical equilibrium problems and found to be very successful in capturing the accurate estimations of mole numbers in chemical products. One of the early attempts at solving the phase equilibrium problems through a metaheuristic algorithm was accomplished by Pan and Firoozabadi^58^ using the Simulated Annealing (SA) optimizer to solve vapor–liquid equilibria near phase boundaries. Rangaiah^59^ evaluated the effectiveness of Genetic Algorithms (GA) and SA for solving phase equilibrium and stability problems. The algorithms are tested on several case studies, and the results show that both GA and SA perform well in solving the problems, with GA showing superior performance in some cases. In another work, Fernández-Vargas et al.^60^ examined the performance of various stopping criteria for population-based metaheuristics in global optimization for phase equilibrium calculations and modeling. Their results provided valuable insights for improving population-based metaheuristics in chemical engineering. Moodley et al.^61^ explored the application of the Krill Herd (KH) optimization technique to phase equilibrium calculations. Their proposed method was tested on several case studies and compared with other optimization techniques, demonstrating its superior accuracy and computational efficiency. Bamikole and Narasigadu^62^ investigated four different swarm-based stochastic optimization algorithms, including the recently emerged metaheuristics of the Honey Badger Algorithm (HBA), Pathfinder Algorithm (PFA), Horse Herd Optimization algorithm (HOA), and Red Fox Optimization (RFO) to solve various phase stability and equilibrium problems. Respective prediction results obtained from these competitive algorithms reveal that HBA reaches the best solution accuracy between the compared optimizers for given test cases.

As it is observed from the completed literature studies on solving chemical equilibrium problems utilizing stochastic methods, accurate minimization of the defined Gibbs Free Energy equation is the ruling factor. It plays a decisive role in obtaining the precise distribution of mole numbers in chemical products. This study proposes a novel thermodynamic model that concurrently employs the Stochiometric Equilibrium Equation and Atomic Mass Balance Equation to attain the correct mole numbers in the chemical equilibrium at the specific operation conditions. These complementary mathematical models are integrated into a set of nonlinear equations, and a metaheuristic-based solver is utilized to solve the defined set of nonlinear equations rather than conventional nonlinear equation solvers such as Newton–Raphson or Continuity methods. The following section explains the proposed mathematical model’s essential steps for finding the reacting components’ equilibrium points in the vapor phase.

## The proposed mathematical model for calculating the equilibrium point of reacting components

Calculating the products produced during a chemical reaction takes work to grasp. Only via experimentation and as a function of environmental factors like temperature, pressure, and time can the actual products of a chemical reaction be identified. Actual products may change as these parameters vary during the ongoing reaction process. Using the equilibrium notion is one method of determining what products will occur. When no outside force or energy is present, all physical systems try to reduce their energy levels. For instance, surface tension causes a bubble to assume a spherical shape with the smallest area possible for the present volume. When temperature and pressure remain constant and enough time is given, chemical reactions try to decrease their energy level. This state of reaction is called chemical equilibrium. The equilibrium equation can be expressed in general form by the following:

17 $$v_{A} A + v_{B} B \rightleftarrows v_{C} C + v_{D} D$$

where *A* and *B* are the reactants, and *C* and *D* are the products of the defined equilibrium reaction. The parameter *v* is the stoichiometric coefficient associated with the reactive system components of *A*, *B*, *C*, and *D*. The following equilibrium case is also valid for general expression.

18 $$- \frac{{dn_{A} }}{{v_{A} }} = - \frac{{dn_{B} }}{{v_{B} }} = \frac{{dn_{C} }}{{v_{C} }} = \frac{{dn_{D} }}{{v_{D} }} = d\varepsilon$$

where *dn*_A,*B*,*C*,*D*_ symbolizes the change of the particular components in the reactive system with increasing reaction time, *dε* is the proportionality factor. Equation (18) can be reformulated using the equations given below.

19 $$dn_{A} = - v_{A} d\varepsilon ,dn_{B} = - v_{B} d\varepsilon ,dn_{C} = - v_{C} d\varepsilon ,dn_{D} = - v_{D} d\varepsilon$$

For the defined case in Eq. (19), the Gibbs Free Energy equation takes the below-given form at the equilibrium condition.

20 $$dG\left( {T,P} \right) = - v_{A} \mu_{A} - v_{B} \mu_{B} + v_{C} \mu_{C} + v_{D} \mu_{D} = 0$$

where *µ* is the chemical potential of the respective system components (*A*, *B*, *C*, *D*) and can be formulated for the ideal gas mixture in the form of a specific Gibbs Free Energy function by the following equation

21 $$\mu_{i} = h_{i}^{o} + Ts_{i}^{o} + RT\ln \left( {\frac{{P_{i} }}{{P_{ref} }}} \right) = g_{i}^{o} + RT\ln \left( {\frac{{P_{i} }}{{P_{ref} }}} \right)$$

where *h*_i_^o^, *s*_i_^o^, and g_i_^o^ are the enthalpy, entropy, and Gibbs free energy value of the *i*th chemical system component at the ideal gas state; *P*_*i*_ is the partial pressure of the *i*th element in the ideal gas state. *R* is the universal gas constant; *T* is the working temperature; and *P*_*ref*_ is the reference pressure. Considering the ideal gas conditions, the partial pressure of the *i*th reactive system component can be expressed by the mole fraction of the respective element using the following equation.

22 $$P_{i} = \frac{{n_{i} }}{{n_{total} }}P$$

where *n*_*i*_ is the mole number of the *i*th system component; *n*_total_ stands for the total number of mole numbers in the reactive system; *P* is the operating pressure of the chemical system. Integrating Eq. (22) into Eq. (21) yields the below given formulation

23 $$\mu_{i} = g_{i}^{o} + RTln\left( {\frac{{n_{i} }}{{n_{total} }}\frac{P}{{P_{ref} }}} \right)$$

Then, the Gibbs Free Energy equation at the equilibrium point defined in Eq. (20) takes the final form by substituting Eq. (23) into the base equation.

24 $$\begin{gathered} dG\left( {T,P} \right) = - v_{A} \left( {g_{A}^{o} + RT\ln \left( {\frac{{n_{A} }}{{n_{total} }}\frac{P}{{P_{ref} }}} \right)} \right) - v_{B} \left( {g_{B}^{o} + RT\ln \left( {\frac{{n_{B} }}{{n_{total} }}\frac{P}{{P_{ref} }}} \right)} \right) \hfill \\ \quad \quad \quad \quad \quad + v_{C} \left( {g_{C}^{o} + RT\ln \left( {\frac{{n_{C} }}{{n_{total} }}\frac{P}{{P_{ref} }}} \right)} \right) + v_{D} \left( {g_{D}^{o} + RT\ln \left( {\frac{{n_{D} }}{{n_{total} }}\frac{P}{{P_{ref} }}} \right)} \right) = 0 \hfill \\ \end{gathered}$$

Knowing that

25 $$dG^{O} \left( {T,P} \right) = - v_{A} g_{A}^{o} - v_{B} g_{B}^{o} + v_{C} g_{C}^{o} + v_{D} g_{D}^{o} = 0$$

and integrating Eq. (25) into Eq. (24) yields to the following expression

26 $$\begin{gathered} dG\left( {T,P} \right) = dG^{O} \left( {T,P} \right) - RT\left( {v_{A} \ln \left( {\frac{{n_{A} }}{{n_{total} }}\frac{P}{{P_{ref} }}} \right)} \right) + v_{B} \ln \left( {\frac{{n_{B} }}{{n_{total} }}\frac{P}{{P_{ref} }}} \right) \hfill \\ \quad \quad \quad \quad \quad - v_{C} \ln \left( {\frac{{n_{C} }}{{n_{total} }}\frac{P}{{P_{ref} }}} \right) - v_{D} \ln \left( {\frac{{n_{D} }}{{n_{total} }}\frac{P}{{P_{ref} }}} \right) = 0 \hfill \\ \end{gathered}$$

The defined Eq. (25) can also be re-formulated by the below expression

27 $$\exp \left( { - \frac{{dG^{O} \left( {T,P} \right)}}{RT}} \right) = \left( {\frac{{n_{A}^{{v_{A} }} n_{B}^{{v_{B} }} }}{{n_{C}^{{v_{C} }} n_{D}^{{v_{D} }} }}} \right)\left( {\frac{P}{{n_{total} P_{ref} }}} \right)^{{v_{A} + v_{B} - v_{C} - v_{D} }}$$

The left-hand side of Eq. (27) is called Equilibrium Constant, $K\left( T \right) = \exp \left( { - \frac{{dG^{O} \left( {T,P} \right)}}{RT}} \right)$, which is the sole function of working temperature (*T*) for ideal gases. More than the stochiometric equilibrium equation is needed to ensure all molecular balance between the reacting components in the chemical system. Atomic mass balance is also required to obtain the reacting components’ accurate composition in the equilibrium state. The following expression can formulate an atomic mass balance equation.

28 $$\mathop \sum \limits_{j = 1}^{NS} A_{ij} n_{j - } b_{i}^{o} = 0 \left( {i = 1, \ldots ,NA} \right)$$

where *NA* is the number of chemical elements in the reaction; *NS* is the number of species in the reaction; *A*_*ij*_ is the number of kilogram atoms per kmole of species *j*; $b_{i}^{0}$ is the assigned number of kilogram atoms element *i* per kmol of total reactants; *n*_*j*_ is the mole number of the *j*th species in the chemical mixture. A descriptive example is provided below to explain Eq. (28) briefly. Assume that a chemical reaction occurs by the input moles of reactants such that one kmole CH_4_ and ten kmole H_2_O enter a chemical reaction at the defined operating temperature and pressure, and other chemicals which are expected to be formed (not existed yet) in the reaction are H_2_, CO_2_, CO, and O_2_. The representative chemical reaction for the given mole number of compounds can be expressed by the following

29 $${\text{CH}}_{4} + 10{\text{H}}_{2} {\text{O}} \to n_{1} {\text{CH}}_{4} + n_{2} {\text{H}}_{2} {\text{O}} + n_{3} {\text{H}}_{2} + n_{4} {\text{CO}}_{2} + n_{5} {\text{CO}} + n_{6} {\text{O}}_{2}$$

Following that, the “A” matrix defined in Eq. (28) can be constructed by the following

30 $$A = \left[ {\begin{array}{*{20}c} & {{\text{CH}}_{4} } & {{\text{H}}_{2} {\text{O}}} & {{\text{H}}_{2} } & {{\text{CO}}_{2} } & {{\text{CO}}} & {{\text{O}}_{2} } \\ {\text{H}} & 4 & 2 & 2 & 0 & 0 & 0 \\ {\text{C}} & 1 & 0 & 0 & 1 & 1 & 0 \\ {\text{O}} & 0 & 1 & 0 & 2 & 1 & 2 \\ \end{array} } \right]$$

*b*_*i*_^*0*^ is formed by the multiplication of the number of atoms with the inlet mole numbers of the *i*^th^ component and calculated for each inlet atom by the following equation

31 $$\begin{gathered} b_{H}^{o} = 1 \times 4 + 10 \times 2 + 0 \times 2 + 0 \times 0 + 0 \times 0 + 0 \times 0 = 24 \hfill \\ b_{C}^{o} = 1 \times 1 + 10 \times 0 + 0 \times 0 + 0 \times 1 + 0 \times 1 + 0 \times 0 = 1 \hfill \\ b_{D}^{o} = 1 \times 0 + 10 \times 1 + 0 \times 0 + 0 \times 2 + 0 \times 1 + 0 \times 2 = 10 \hfill \\ \end{gathered}$$

Substituting these obtained values in the Eq. (28) yields the following matrix expression.

32 $$\left[ {\begin{array}{*{20}c} 4 & 2 & 2 & 0 & 0 & 0 \\ 1 & 0 & 0 & 1 & 1 & 0 \\ 0 & 1 & 0 & 2 & 1 & 0 \\ \end{array} } \right]\left[ {\begin{array}{*{20}c} {n_{{{\text{CH}}_{4} }} } \\ {n_{{{\text{H}}_{2} {\text{O}}}} } \\ {n_{{{\text{H}}_{2} }} } \\ {n_{{{\text{CO}}_{2} }} } \\ {n_{{{\text{CO}}}} } \\ {n_{{{\text{O}}_{2} }} } \\ \end{array} } \right] = \left[ {\begin{array}{*{20}c} {24} \\ 1 \\ {10} \\ \end{array} } \right]$$

Combining atomic mass balance and stoichiometric equations forms a semi-definite system of equations. There must be more than atomic balance to solve the equation when you have more molecule outputs in equilibrium. For the remaining molecules, a stoichiometric equilibrium nonlinear equation is also required. Let us examine this formulation using another informative example. Suppose that one kmole O_2_ and one kmole CO attain an equilibrium condition at 100 kPa and 3000 K. It is investigated to find the distribution of the mole numbers of the formed products at the equilibrium point for the below given stoichiometric equilibrium equation.

33 $${\text{CO}} + 0.5{\text{O}}_{2} { \leftrightarrows }{\text{CO}}_{2}$$

where prescribed reference pressure *P*_*ref*_ for this equilibrium reaction is 100 kPa. The atomic mass balance for this reaction can be constructed by considering the input moles of 1 kmole *CO*, one kmole *O*_*2*_, and zero mole input of *CO*. Knowing that there are two different atoms, *C* and *O*, in the reaction pool, the following equation expresses atomic mass balance equation.

34 $$\mathop \sum \limits_{j = 1}^{2} A_{ij} n_{j} - b_{i}^{0} = 0\quad i \in \left( {{\text{CO}},{\text{O}}_{2} ,{\text{CO}}_{2} } \right)\quad j \in \left( {{\text{C}},{\text{O}}} \right)$$

Then, *A* matrix for the given equilibrium case can be constructed as

35 $$A = \left[ {\begin{array}{*{20}c} s & {{\text{CO}}} & {{\text{O}}_{2} } & {{\text{CO}}_{2} } \\ {\text{C}} & 1 & 0 & 1 \\ {\text{O}} & 1 & 2 & 2 \\ \end{array} } \right]$$

where *b*^0^ matrix is formed by using numeric values given in the A matrix and calculated as

36 $$b^{0} = \left[ {\begin{array}{*{20}c} 1 \\ 3 \\ \end{array} } \right]$$

Finally, the atomic balance equation is formulated in the matrix form by the following.

37 $$\left[ {\begin{array}{*{20}c} 1 & 0 & 1 \\ 1 & 2 & 2 \\ \end{array} } \right]\left[ {\begin{array}{*{20}c} {n_{{{\text{CO}}}} } \\ {n_{{{\text{O}}_{2} }} } \\ {n_{{{\text{CO}}_{2} }} } \\ \end{array} } \right] = \left[ {\begin{array}{*{20}c} 1 \\ 3 \\ \end{array} } \right]$$

And respective stoichiometric equilibrium equation of this case takes the final form of

38 $$K\left( T \right) = \exp \left( { - \frac{{dG^{0} \left( {T,P} \right)}}{RT}} \right) = \left( {\frac{{n_{{{\text{CO}}}} n_{{{\text{O}}_{2} }}^{\frac{1}{2}} }}{{n_{{{\text{CO}}_{2} }} }}} \right)\left( {\frac{{100\left( {@P_{react} } \right)}}{{\left( {n_{{{\text{CO}}}} + n_{{{\text{O}}_{2} + n_{{{\text{CO}}_{2} }} }} } \right)100\left( {@P_{ref} } \right)}}} \right)^{{1 + \frac{1}{2} - 1}}$$

There are three unknown parameters in the equation sets. Still, since two of them can be solved as linear solvers (expressed in Eq. 39), the defined function can have only one unknown, and two other variables are dependent on this unknown value. We can reformulate the above-described set of equations as a function of the mole number of CO_2_ (it can be any component in the chemical mixture; CO_2_ is haphazardly chosen) that is to be formed in the reaction by the below-given equation sets.

39 $$f_{1} \to \left[ {\begin{array}{*{20}c} 1 & 0 \\ 1 & 2 \\ \end{array} } \right]\left[ {\begin{array}{*{20}c} {n_{{{\text{CO}}}} } \\ {n_{{{\text{O}}_{2} }} } \\ \end{array} } \right] = \left[ {\begin{array}{*{20}c} 1 \\ 3 \\ \end{array} } \right] - \left[ {\begin{array}{*{20}c} 1 \\ 2 \\ \end{array} } \right]n_{{{\text{CO}}_{2} }}$$

40 $$f_{2} \to \left( {\frac{{n_{{{\text{CO}}}} n_{{{\text{O}}_{2} }}^{\frac{1}{2}} }}{{n_{{{\text{CO}}_{2} }} }}} \right)\left( {\frac{1}{{\left( {n_{{{\text{CO}}}} + n_{{{\text{O}}_{2} }} + n_{{{\text{CO}}_{2} }} } \right)}}} \right)^{{1 + \frac{1}{2} - 1}} - k\left( T \right) = 0$$

Considering ideal gas conditions for the reacting components, equilibrium constant *K*(*T*) is the sole function of the operating temperature *T* and can be calculated in advance. The primary aim should be to solve f_1_ and f_2_ concurrently to obtain accurate CO, O_2_, and CO_2_ mole numbers in the reactive mixture. With the known value of $n_{{{\text{CO}}_{2} }}$, remaining unknown mole numbers, $n_{{{\text{CO}}}}$ and $n_{{{\text{O}}_{2} }}$ can be easily calculated by advanced mathematical tools to solve a set of linear equations. In addition, they must satisfy the defined equality constraint given in Eq. (40) to eliminate infeasible solutions. Above defined set of equations composed of a linear set of equations and stoichiometric equilibrium equation expressed in the form of equality constraint can be converted into a nonlinear system of equations, defined in the form of *f*_*1*_ and *f*_*2*_, and accurately solved by well-devised equation solvers. Retaining the accurate solutions of nonlinear systems of equations requires advanced algorithmic procedures that can be categorized into two main branches: conventional and stochastic methods. Traditional problem solvers, such as Newton-type methods, are skillful operators. However, they need a good initial guess to obtain successful answers, and wrong initial points may lead to deceptive results. In addition, these Newton-type methods need accurate derivative information of the solution space, which may not always be possible as discontinuous regions may occur, collapsing the entire iterative process of the responsible solver. To overcome these structural algorithmic drawbacks, stochastic optimization methods such as metaheuristics have been frequently utilized in the near-past literature studies for solving nonlinear systems of equations as they are derivative-free and require a small amount of computational budget compared to conventional analytic solvers^63,64^. Here in this research study, the proposed HMRFO algorithm is put into practice to attain the accurate distribution of the reactive components in a chemical system by solving the above-defined system of nonlinear equations. Within its general form, the system of nonlinear equation sets can be described by the following mathematical expression,

41 $$\begin{gathered} f_{1} \left( {x_{1} ,x_{2} ,x_{3} , \ldots ,x_{n} } \right) = 0 \hfill \\ f_{2} \left( {x_{1} ,x_{2} ,x_{3} , \ldots ,x_{n} } \right) = 0 \hfill \\ \vdots \quad \quad \quad \; \vdots \quad \quad \quad \; \vdots \quad \quad \;\;\; \vdots \hfill \\ f_{m} \left( {x_{1} ,x_{2} ,x_{3} , \ldots ,x_{n} } \right) = 0 \hfill \\ \end{gathered}$$

where each function *f*_*i*_* i* = 1,2,…, *m* is a nonlinear equation, mapping the solution vector $\overrightarrow{x}=\left({x}_{1},{x}_{2},\dots ,{x}_{n}\right)$ into n-dimensional solution space of real numbers *R*^*n*^. Some functions in the equation set may be linear, and others nonlinear, depending on the mathematical characteristic of the system. A successful solution to this defined system given in Eq. (41) requires finding the accurate values of variables in a vector that makes each function in the nonlinear system equal to zero. That is, if *f*_*i*_ = 0 *i* = 1,2,… *m*, then the corresponding solution ${\overrightarrow{x}}^{*}=\left({x}_{1}^{*},{x}_{2}^{*},\dots ,{x}_{n}^{*}\right)$ becomes the optimal solution of the problem. To solve a nonlinear system of equations through metaheuristic optimizers, nonlinear equation sets are transformed into a definite single objective unconstrained optimization problem, whose problem objective is represented by the sum of the squared residuals of nonlinear equations *f*_*i*_ = *i* = 1,2,…, *m*. This converted optimization problem can be formulated by

42 $$\min F\left( {\mathop{x}\limits^{\rightharpoonup} } \right) = \min\left( {\sqrt {\mathop \sum \limits_{i = 1}^{m} f_{i}^{2} \left( {\mathop{x}\limits^{\rightharpoonup} } \right)} } \right)$$

where $F(\vec{x})$ is the objective function of the minimization problem. The following section will evaluate the optimization performance of the proposed HMRFO algorithm by solving four different chemical equilibrium problems.

## Discussion on the comparative performance of the proposed HMRFO over chemical equilibrium problems

This section assesses the prediction accuracy of the HMRFO algorithm over four challenging chemical equilibrium problems. Respective estimation results obtained for these problems by the developed HMRFO are benchmarked against those acquired by the recently developed metaheuristic algorithms of Firefly Optimizer (FIREFLY)^13^, PSO, Thermal Exchange Optimization (THERM)^65^, HARRIS , Poor and Rich Optimization (PRO)^66^, Reptile Search Algorithm (REPTILE)^67^, EQUIL, JAYA , Spotted Hyena Optimizer (SPOTTED)^68^, Aquila Optimizer (AQUILA)^69^, Crow Search (CROW)^70^, African Vultures Optimization (AFRICAN)^71^, Runge–Kutta Optimizer (RUNGE)^72^, and Gradient-based Optimizer (GRAD)^73^. All these optimization algorithms are run 100 times for varying numbers of function evaluations, and obtained results are evaluated using statistical measures. In this concept, the algorithm yielding the minimum fitness function value retained after consecutive algorithm runs provides the most accurate distribution of mole numbers of the system components in the reactive mixture.

### Case study 1: glucose steam gasification process^74^

The chemical system contains six species formed from Carbon, Hydrogen, and Oxygen. The following is the set of equilibrium reactions describing the Glucose Steam Gasification Process.

43 $$\begin{gathered} {\text{C}} + {\text{H}}_{2} {\text{O}}{ \leftrightarrows }{\text{H}}_{2} + {\text{CO}} \hfill \\ {\text{CO}} + 3{\text{H}}_{2} { \leftrightarrows }{\text{CH}}_{4} + {\text{H}}_{2} {\text{O}} \hfill \\ {\text{CO}} + {\text{H}}_{2} {\text{O}}{ \leftrightarrows }{\text{H}}_{2} + {\text{CO}}_{2} \hfill \\ \end{gathered}$$

The aim is to investigate the actual mole number values of each chemical system component at the equilibrium point under the operational conditions covering 1 bar working pressure and 2000 K reaction temperature. The chemical equation representing the inlet mole numbers of the reactive components can be given as

44 $${\text{CH}}_{4} + {\text{CO}}_{2} + {\text{H}}_{2} {\text{O}} + {\text{CO}} + {\text{C}} + 3{\text{H}}_{2} \to n_{{{\text{CH}}_{4} }} {\text{CH}}_{4} + n_{{{\text{CO}}_{2} }} {\text{CO}}_{2} + n_{{{\text{H}}_{2} {\text{O}}}} {\text{H}}_{2} {\text{O}} + n_{{{\text{CO}}}} {\text{CO}} + n_{{\text{C}}} {\text{C}} + n_{{{\text{H}}_{2} }} {\text{H}}_{2}$$

**Fig. 22:**
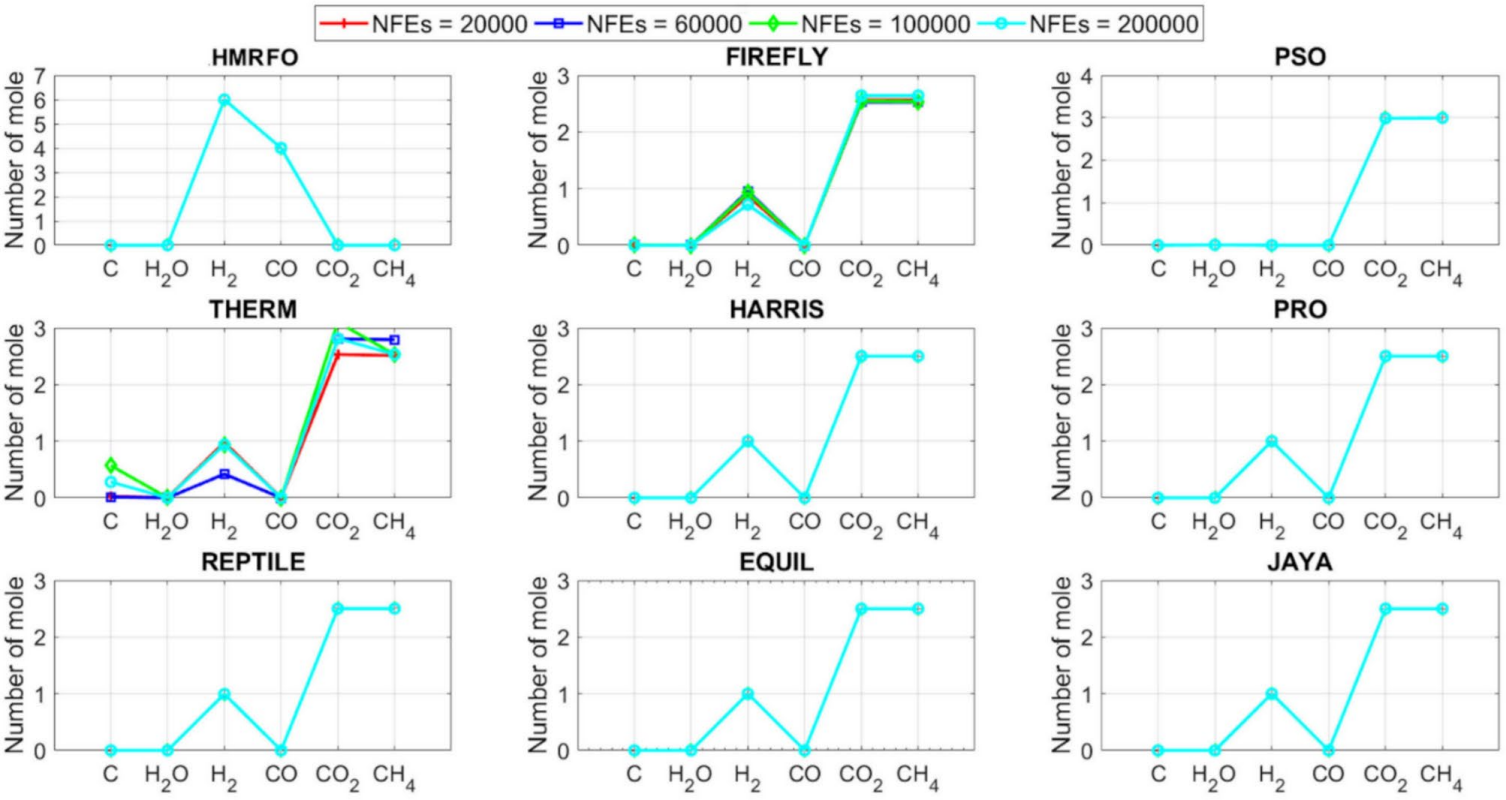
Optimal values of the mole numbers of the reacting components at the equilibrium point found by HMRFO, FIREFLY, PSO, THERM, HARRIS, PRO, REPTILE, EQUIL, and JAYA algorithms for the Glucose Steam Gasification Process.

**Fig. 23:**
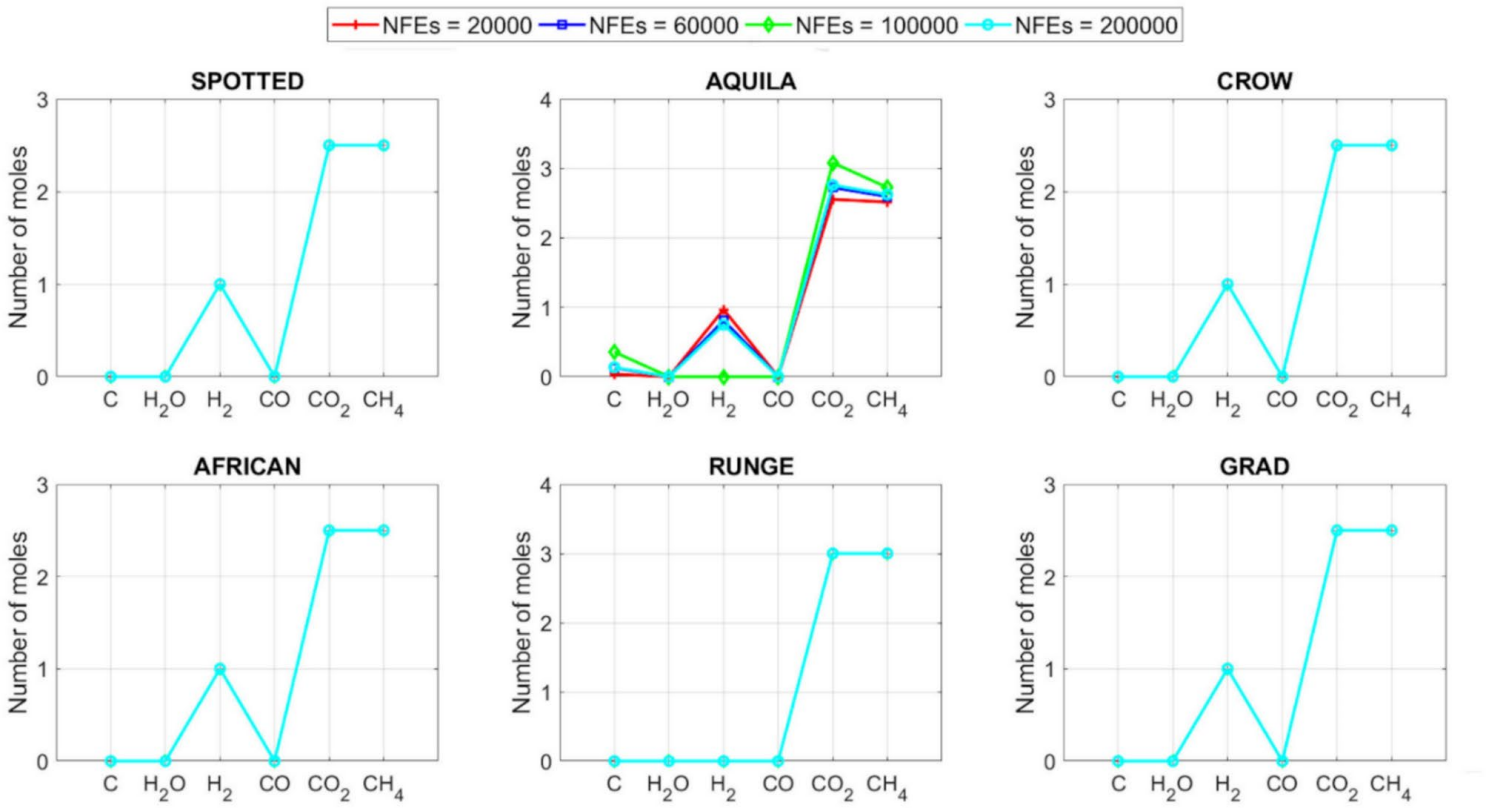
Optimal distribution of the reactive components in the chemical mixture at the equilibrium conditions for the Glucose Steam Gasification Process.

**Fig. 24:**
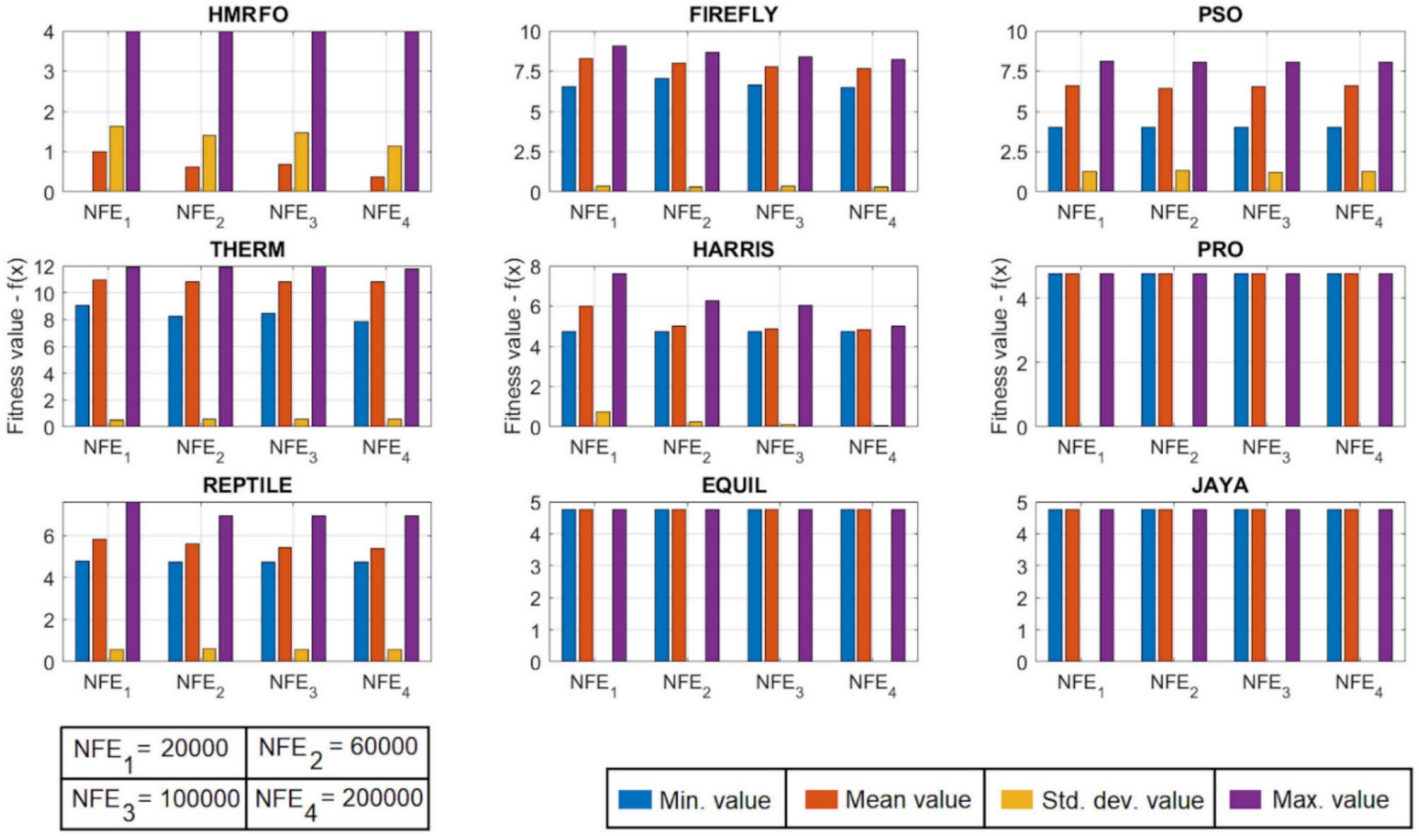
Statistical results obtained for varying numbers of function evaluations found by HMRFO, FIREFLY, PSO, THERM, HARRIS, PRO, REPTILE, EQUIL, and JAYA algorithms.

**Fig. 25:**
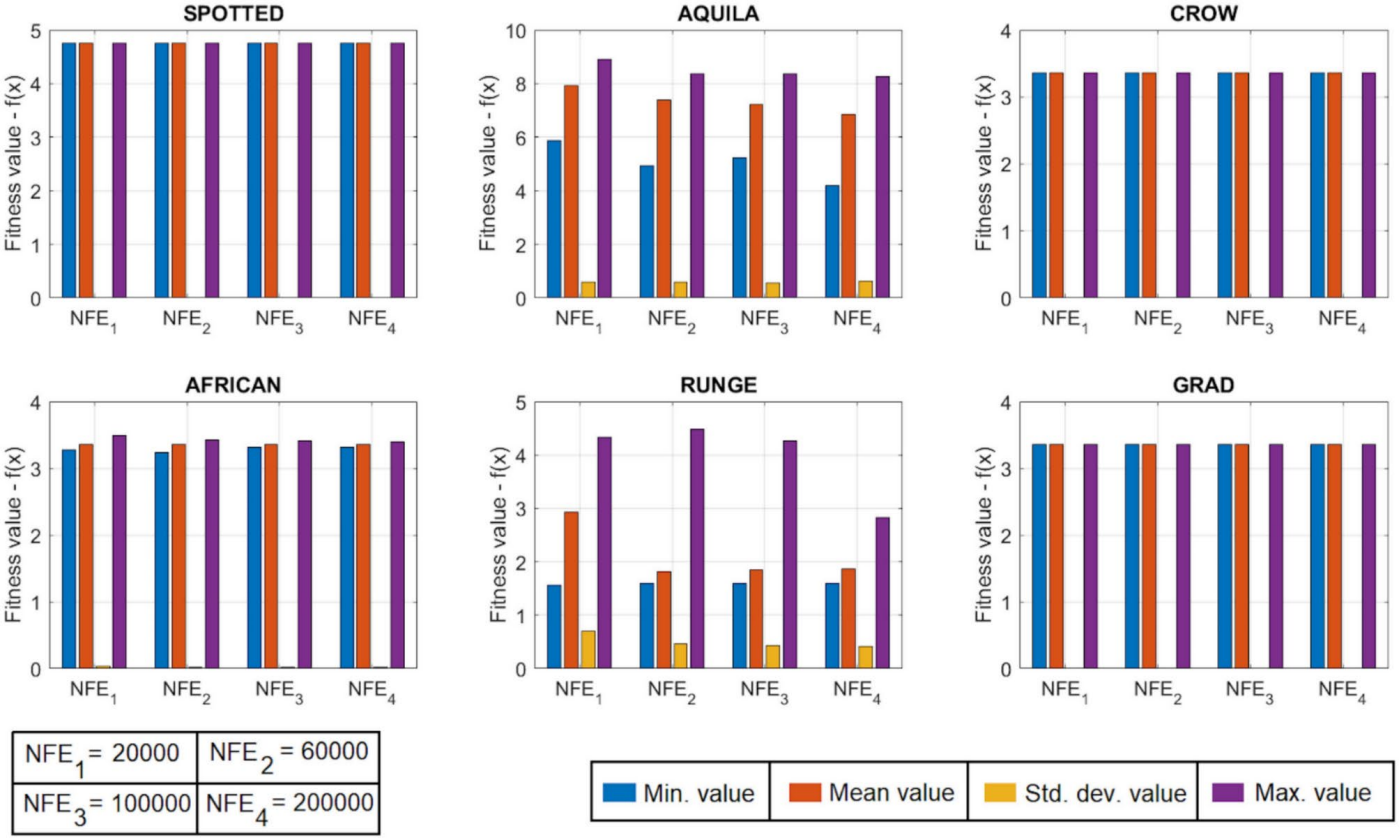
Comparison of the statistical results for the competitive algorithms of SPOTTED, AQUILA, CROW, AFRICAN, RUNGE, and GRAD for the case study 1.

Figures 22 and 23 visualize the optimal mole number values of the components at the equilibrium point for competitive algorithms for varying numbers of function evaluations. It is seen that increasing the number of iterations does not have any influential effect on the distribution of mole numbers in the reaction pool; according to the statistical results represented in the bar charts given in Figs. 24 and 25, HMRFO provides the most accurate prediction for a varying number of functional evaluation levels. Statistic results obtained for NFEs = 200,000 for the compared algorithms reported in Table 17 also verify this conclusion that minimum fitness value f(x) = 3.14855E−13 retrieved by HMRFO is much better than those retained by the retained algorithms. This situation can be comprehended that all available CH_4_, CO_2_, H_2_O, and C are depleted in the reaction, and 5.997968112 mol H_2_ and 3.999202825 mol CO are formed at the equilibrium point. For this case, HMRFO outperforms the remaining algorithms, considering the best fitness value and persistent solution robustness, which is evident when statistical results are carefully examined in Table 17.

**Table 17:** Comparison of the optimal mole number of the chemical mixture components found by the competitive algorithms.

	HMRFO	FIREFLY	PSO	THERM	HARRIS
*n*_C_	0.000000000	0.000186322	0.000000000	0.279737354	0.000000000
$n_{{{\text{H}}_{2} {\text{O}}}}$	0.000000000	0.000021941	0.008725113	0.000007236	0.000077742
$n_{{{\text{H}}_{2} }}$	5.997968112	0.717132001	0.000000000	0.936968044	1.000000000
*n*_CO_	3.999202825	0.000000000	0.000000000	0.000000000	0.000000000
$n_{{{\text{CO}}_{2} }}$	0.000089917	2.641587410	2.986912042	2.811241981	2.499883382
$n_{{{\text{CH}}_{4} }}$	0.000707257	2.641423028	2.995637349	2.531512193	2.499961128
*f*_*min*_	**3.14855E−13**	6.461944482	4.024924499	7.859255052	4.750241892
*f*_*aver*_	0.374803521	7.655037574	6.586915256	10.84015933	4.815100693
*f*_*std*_	1.143130773	0.301645231	1.263529163	0.549816362	0.058880829
*f*_*max*_	3.991293222	8.205319195	8.087835342	11.78474478	5.011506816

	PRO	REPTILE	EQUIL	JAYA	SPOTTED
*n*_C_	0.000000000	0.000000000	0.000000000	0.000000000	0.000000000
$n_{{{\text{H}}_{2} {\text{O}}}}$	0.000000000	0.000080751	0.000080225	0.000080222	0.000080225
$n_{{{\text{H}}_{2} }}$	1.000000000	0.995043233	1.000000000	1.000000000	1.000000000
*n*_CO_	0.000000000	0.000000000	0.000000000	0.000000000	0.000000000
$n_{{{\text{CO}}_{2} }}$	2.499879862	2.502357235	2.499879662	2.499879623	2.499879682
$n_{{{\text{CH}}_{4} }}$	2.499959992	2.502437986	2.499959883	2.499959882	2.499959882
*f*_*min*_	4.750201823	4.753038083	4.750201709	4.750201701	4.750201701
*f*_*aver*_	4.750210742	5.387791546	4.750201709	4.750201701	4.750201701
*f*_*std*_	1.19439E−05	0.569648436	2.03976E−14	2.04238E−14	7.43732E−11
*f*_*max*_	4.750280194	6.916075235	4.750201709	4.750201701	4.750201711

	AQUILA	CROW	AFRICAN	RUNGE	GRAD
*n*_C_	0.136898922	0.000000000	0.000000000	0.000000000	0.000000000
$n_{{{\text{H}}_{2} {\text{O}}}}$	0.000000000	0.000080225	0.000069776	0.000056792	0.000080225
$n_{{{\text{H}}_{2} }}$	0.750047777	1.000000000	1.000000000	0.000000000	1.000000000
*n*_CO_	0.000000000	0.000000000	0.000000000	0.000000000	0.000000000
$n_{{{\text{CO}}_{2} }}$	2.761874999	2.499879667	2.499895332	2.999914811	2.499879662
$n_{{{\text{CH}}_{4} }}$	2.624976022	2.499959887	2.499965114	2.999971623	2.499959887
*f*_*min*_	4.205330122	3.359007599	3.312828155	1.598606105	3.359007224
*f*_*aver*_	6.846307377	3.359007632	3.356383772	1.864339695	3.359007626
*f*_*std*_	0.628990654	1.17617E−08	0.015757213	0.410451373	4.03051E−08
*f*_*max*_	8.277821655	3.359007652	3.401098912	2.823652506	3.359007709

Significant values are in [bold].

### Case study 2: oxidation of propane^75^

The present reaction illustrates the combustion of propane with pure oxygen at operation temperatures of 2500 K and 2 bar system pressure. The chemical mixture comprises gaseous products involving ten species (CO_2_, CO, O_2_, H_2_, H_2_O, OH, H, O, N_2_, NO) formed from four organic elements (C, H, O, N). A set of equilibrium reactions representing the basic steps of combustion of propane is expressed by the following

45 $$\begin{gathered} {\text{CO}}_{2} { \leftrightarrows }{\text{CO}} + 0.5{\text{O}}_{2} \hfill \\ {\text{H}}_{2} {\text{O}}{ \leftrightarrows }{\text{H}}_{2} + 0.5{\text{O}}_{2} \hfill \\ {\text{H}}_{2} {\text{O}}{ \leftrightarrows }0.5{\text{H}}_{2} + {\text{OH}} \hfill \\ 0.5{\text{H}}_{2} { \leftrightarrows }{\text{H}} \hfill \\ 0.5{\text{O}}_{2} { \leftrightarrows }{\text{O}} \hfill \\ 0.5{\text{N}}_{2} + 0.5{\text{O}}_{2} { \leftrightarrows }{\text{NO}} \hfill \\ \end{gathered}$$

The optimization problem involves determining the correct mole amounts of the reacting components, which consist of ten species in gas form, and the total number of moles in the gas mixture. The expression below represents the initial mole numbers of the reactants at the specified operational conditions within the structure of the chemical reaction equation.

46 $$\begin{gathered} {\text{CO}}_{2} + {\text{CO}} + {\text{O}}_{2} + {\text{H}}_{2} + {\text{H}}_{2} {\text{O}} + {\text{OH}} + {\text{H}} + {\text{O}} + {\text{N}}_{2} + {\text{NO}} \to n_{{{\text{CO}}_{2} }} {\text{CO}}_{2} + n_{{{\text{CO}}}} {\text{CO}} \hfill \\ \quad + n_{{{\text{O}}_{2} }} {\text{O}}_{2} + n_{{{\text{H}}_{2} }} {\text{H}}_{2} + n_{{{\text{H}}_{2} {\text{O}}}} {\text{H}}_{2} {\text{O}} + + n_{{{\text{OH}}}} {\text{OH}} + n_{{\text{H}}} {\text{H}} + n_{{\text{O}}} {\text{O}} + n_{{{\text{N}}_{2} }} {\text{N}}_{2} + n_{{{\text{NO}}}} {\text{NO}} \hfill \\ \end{gathered}$$

**Fig. 26:**
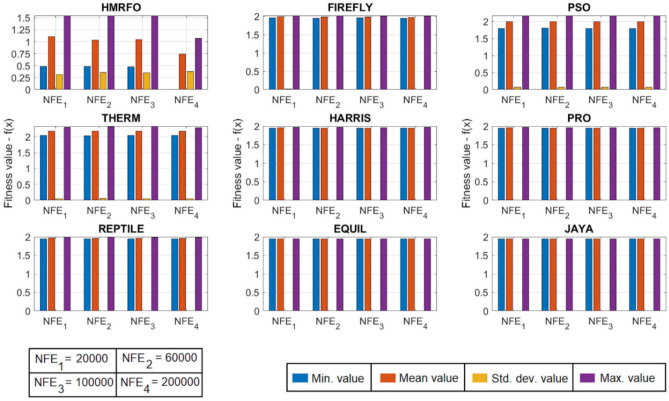
Statistical comparison of the fitness values for varying numbers of function evaluations for the compared algorithms.

**Fig. 27:**
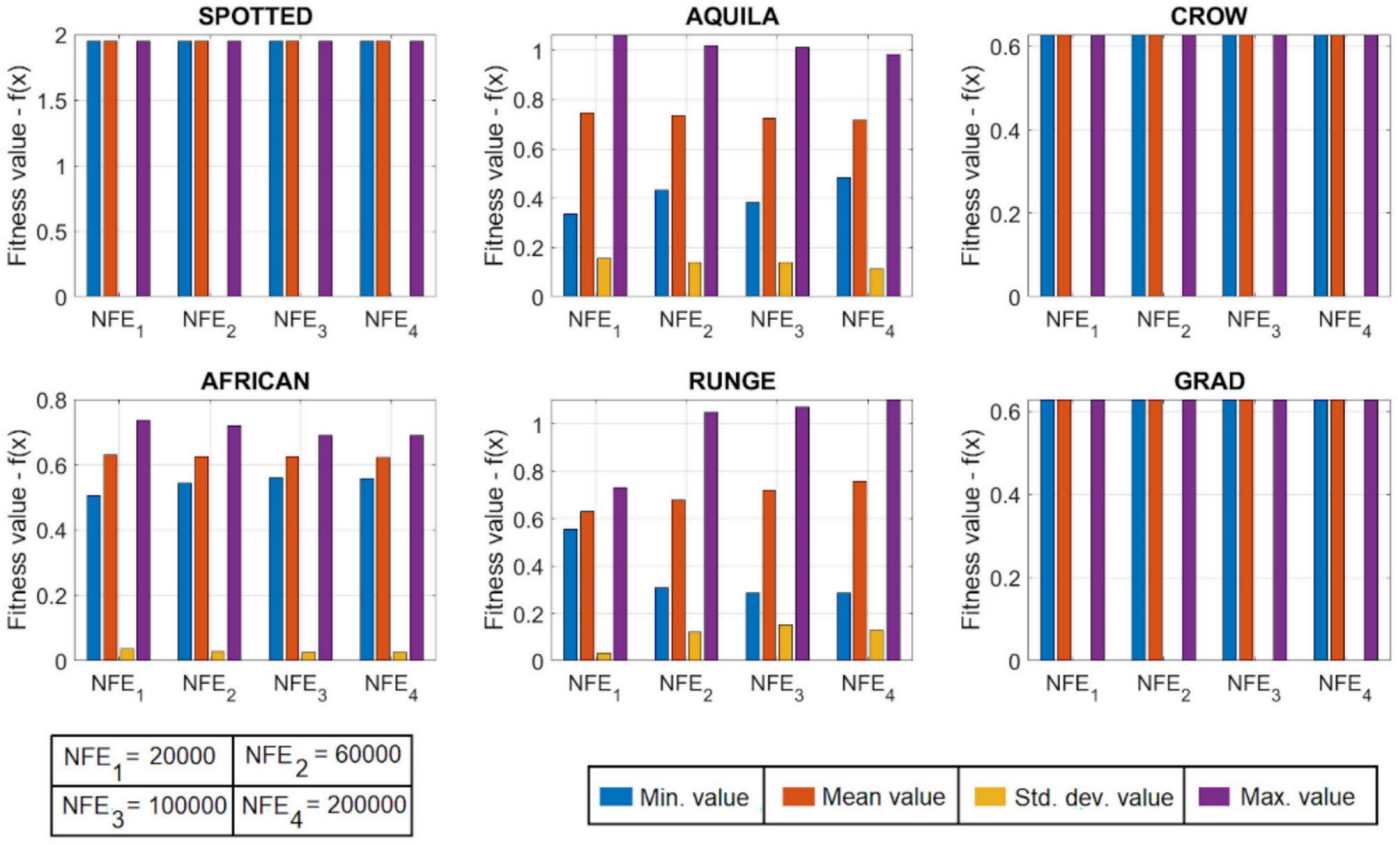
Statistical results obtained for the second case study for the compared algorithms.

**Fig. 28:**
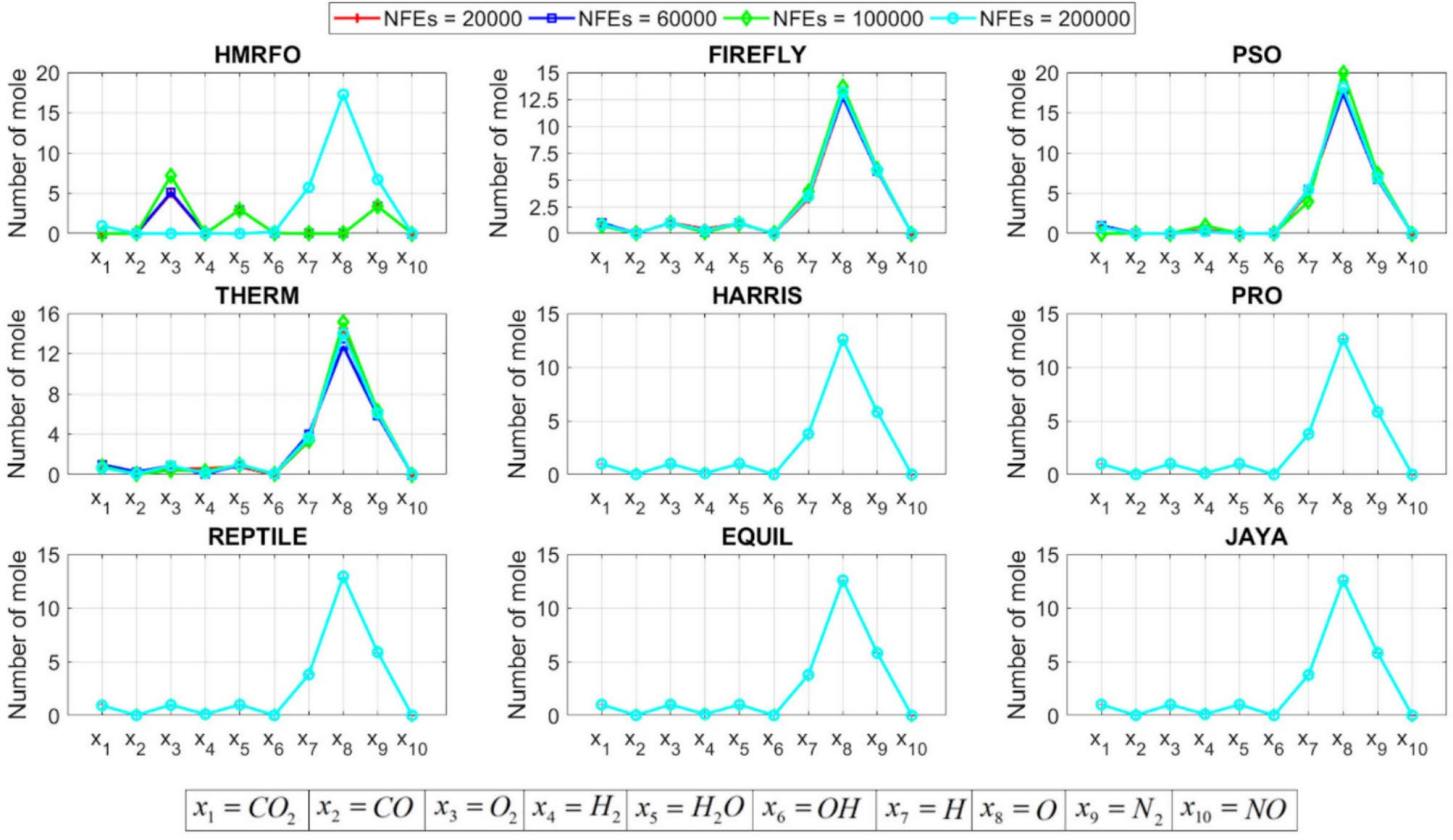
Optimal values of the numbers of the reactive compounds in the chemical mixture.

**Fig. 29:**
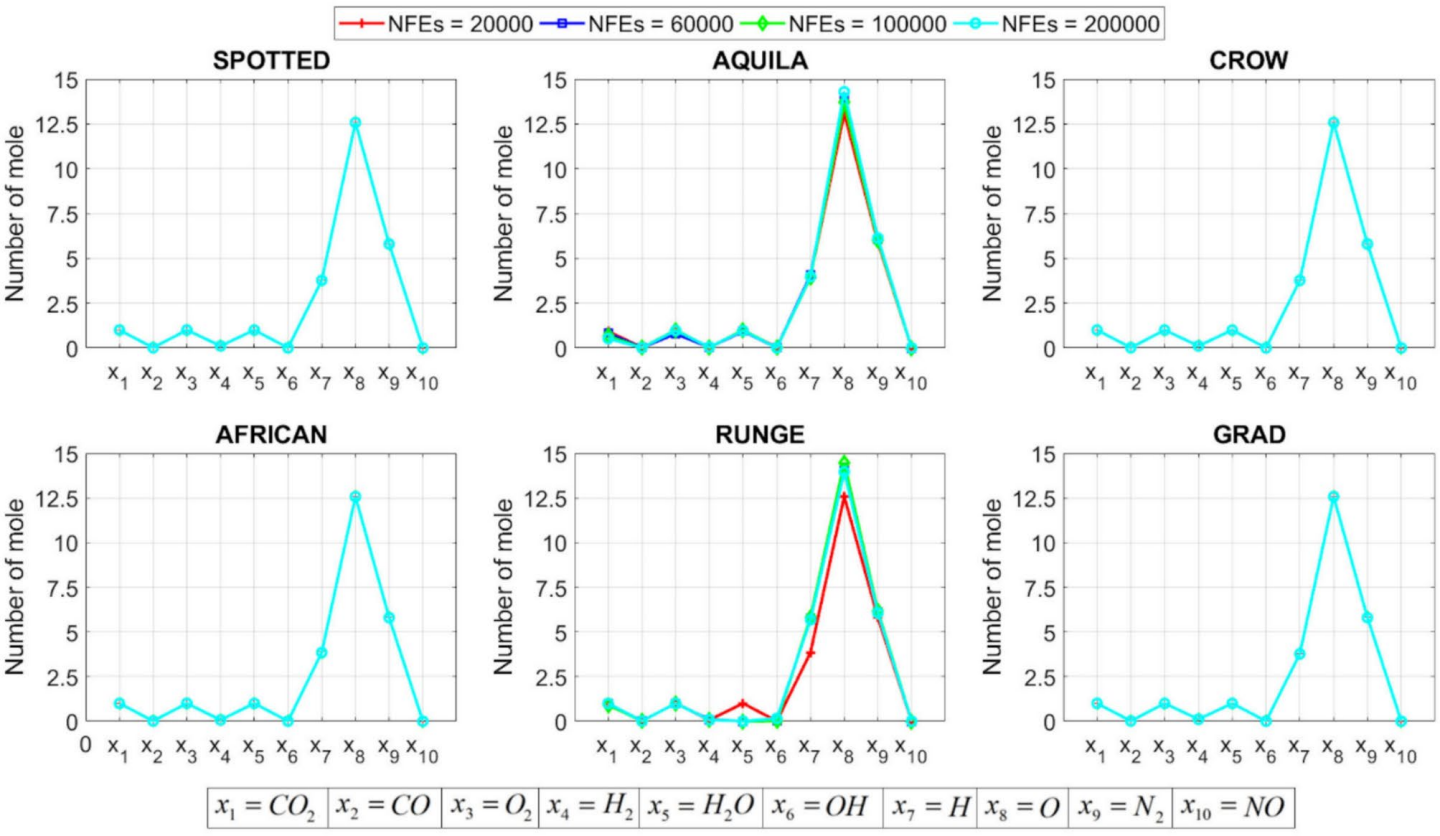
Optimal equilibrium compositions of the product gases at the equilibrium point.

Figures 26 and 27 show the statistical results of the predictions performed by the compared algorithms for varying numbers of function evaluations. When considering lower function evaluations, AQUILA provides better estimations regarding best results than the other methods, including the proposed HMRFO. However, it is significantly surpassed by HMRFO when 200,000 function evaluations are considered for the termination criterion for the compared algorithms. Figures 28 and 29 show the distribution of the reacting components in the chemical mixture obtained by contestant metaheuristic optimizers. For different cases of function evaluations, similar distributions of mole numbers in the chemical mixture have been received by the employed algorithms, which also indicates that stagnation in fitness value evolution is evident for the compared methods, except for HMRFO, as no apparent improvement in objective function values is observed even with increasing number iterations. One of the main reasons for this deficiency is the imbalance between the exploration and exploitation mechanisms inherent in these competitive optimizers, which causes feasible and erroneous predictions to be yielded. HMRFO conducts better communication with these complementary search mechanisms, enabling it to reach much better forecasts than the compared algorithms for the equilibrium case. This behavior is also verified for the statistical results in Table 18, obtained for NFEs = 200,000. Table 18 also reports the respective mole number of the reactive components acquired for the minimum fitness values for each competitive algorithm. Clear dominancy of HMRFO is evident for this case with a corresponding minimum objective function value of *f*(*x*) = 0.00420111957, which is much better than that of the remaining algorithms. It is also interesting to see that EQUIL, JAYA, and SPOTTED reach almost the same fitness values of *f*(*x*) = 1.946645 in nearly every algorithm run, explaining that these optimizers are entrapped in the local solutions and are not able to improve even after 200,000 number of function evaluations. When examining the mole number compositions in the mixture found by HMRFO in Table 18, complete depletion of O_2_, H_2_O, and NO is observed, which brings about the formation of 0.9648 mol CO_2_, 0.0058 mol CO, 0.0343 mol H_2_, 0.2020 mol OH, 5.730 mol H, 17.2674 mol O, and 6.702 mol N_2_.

**Table 18:** Comparison of the mole number of the reacting components at the equilibrium point by the competing algorithms.

	HMRFO	FIREFLY	PSO	THERM	HARRIS
$n_{{{\text{CO}}_{2} }}$	0.964819722	0.850693316	0.778563631	0.653233669	1.000000000
$n_{{{\text{CO}}}}$	0.005766169	0.012978480	0.004849554	0.079221398	0.013587093
$n_{{{\text{O}}_{2} }}$	0.000000000	0.981891341	0.000000000	0.853968724	1.000000000
$n_{{{\text{H}}_{2} }}$	0.034353095	0.284510608	0.269054304	0.140952569	0.107078591
$n_{{{\text{H}}_{2} {\text{O}}}}$	0.000000000	0.985445280	0.000000000	0.998708965	1.000000000
*n*_OH_	0.201981275	0.004899022	0.025952701	0.074489872	0.005447627
*n*_H_	5.729312533	3.455189199	5.435938689	3.646187058	3.781147754
*n*_O_	17.26739901	13.14799324	18.16164784	13.96446276	12.57832424
$n_{{{\text{N}}_{2} }}$	6.702392953	5.908242671	6.874788682	6.065643892	5.798420327
*n*_NO_	0.000000000	0.000000000	0.000000000	0.000000000	0.000000000
*f*_*min*_	**0.004201119**	1.958045549	1.803687864	2.043626183	1.946700761
*f*_*aver*_	0.746572797	1.981337013	1.997249031	2.175828908	1.952031443
*f*_*std*_	0.378063213	0.008158171	0.071754464	0.047461721	0.004427850
*f*_*max*_	1.068965012	2.009973348	2.179167457	2.284854286	1.970916113

	PRO	REPTILE	EQUIL	JAYA	SPOTTED
$n_{{{\text{CO}}_{2} }}$	0.997484023	0.921860696	1.000000000	1.000000000	1.000000000
$n_{{{\text{CO}}}}$	0.012901609	0.012806496	0.013639207	0.013639207	0.013638625
$n_{{{\text{O}}_{2} }}$	1.000000000	0.974778570	1.000000000	1.000000000	1.000000000
$n_{{{\text{H}}_{2} }}$	0.111472203	0.108855185	0.111037668	0.111037596	0.111040288
$n_{{{\text{H}}_{2} {\text{O}}}}$	1.000000000	0.991153808	1.000000000	1.000000000	1.000000000
*n*_OH_	0.005022429	0.005690088	0.005363375	0.005363377	0.005363732
*n*_H_	3.772180728	3.794291922	3.772561287	3.772561430	3.772555690
*n*_O_	12.58966925	12.92919560	12.57753299	12.57753299	12.57753336
$n_{{{\text{N}}_{2} }}$	5.800607449	5.866062266	5.798267788	5.798267788	5.798267859
*n*_NO_	0.000000000	0.000000000	0.000000000	0.000000000	0.000000000
*f*_*min*_	1.946793247	1.950849979	1.946645595	1.946645595	1.946645595
*f*_*aver*_	1.949624977	1.960485278	1.946645595	1.946645595	1.946645597
*f*_*std*_	0.001813126	0.005116473	3.03851E−11	9.32587E−15	1.70564E−09
*f*_*max*_	1.957039534	1.977350970	1.946645595	1.946645595	1.946645606

	AQUILA	CROW	AFRICAN	RUNGE	GRAD
$n_{{{\text{CO}}_{2} }}$	0.525627212	1.000000000	1.000000000	1.000000000	1.000000000
$n_{{{\text{CO}}}}$	0.008089167	0.013639207	0.014732825	0.011179979	0.013639207
$n_{{{\text{O}}_{2} }}$	0.968029380	1.000000000	1.000000000	0.998948908	1.000000000
$n_{{{\text{H}}_{2} }}$	0.046986634	0.111037588	0.072563363	0.083113532	0.111037588
$n_{{{\text{H}}_{2} {\text{O}}}}$	0.957485455	1.000000000	1.000000000	0.000000000	1.000000000
*n*_OH_	0.009955315	0.005363377	0.006727056	0.159218270	0.005363377
*n*_H_	3.981100505	3.772561444	3.848157959	5.674554664	3.772561446
*n*_O_	14.29644588	12.57753299	12.57378881	13.96527704	12.57753299
$n_{{{\text{N}}_{2} }}$	6.129644505	5.798267788	5.797545975	6.065800872	5.798267787
*n*_NO_	0.000000000	0.000000000	0.000000000	0.000000000	0.000000000
*f*_*min*_	0.484349569	0.628211660	0.557377597	0.287360480	0.628211659
*f*_*aver*_	0.718109625	0.628211684	0.623337959	0.757900897	0.628211684
*f*_*std*_	0.114984968	6.44116E−09	0.025696650	0.128163676	1.15333E−08
*f*_*max*_	0.984302318	0.628211707	0.690653235	1.104280947	0.628211711

Significant values are in [bold].

### Case study 3: combustion of methane^74^

The set of reactions defined below represents possible routes for the combustion of methane in the gas state at the operational conditions covering the working temperature of 2500 K and pressure of 2 bar. The chemical mixture of the products in the gaseous phase involves different species, from molecular compounds to atomic elements. The composition mixture of the reaction product at the equilibrium point includes eleven various system components such as CH_4_, H_2_, H, O_2_, O, OH, N_2_, NO, H_2_O, CO, and CO_2_ formed out of four atomic particles (C, H, O, N). The given set of equilibrium reactions below describes the main reactions involved in the combustion of methane.

$$0.5{\text{H}}_{2} \rightleftarrows {\text{H}}$$

$$0.5{\text{O}}_{2} \rightleftarrows {\text{O}}$$

$$0.5{\text{O}}_{2} + 0.5{\text{H}}_{2} \rightleftarrows {\text{OH}}$$

$$0.5{\text{N}}_{2} + 0.5{\text{O}}_{2} \rightleftarrows {\text{NO}}$$

$${\text{H}}_{2} + 0.5{\text{O}}_{2} \rightleftarrows {\text{H}}_{2} {\text{O}}$$

$${\text{CO}} + 0.5{\text{O}}_{2} \rightleftarrows {\text{CO}}_{2}$$

47 $${\text{CH}}_{4} + 2{\text{O}}_{2} \rightleftarrows 2{\text{HO}}_{2} + {\text{CO}}_{2}$$

In this case, the complete oxidation of methane is realized by a stoichiometric amount of air composed of 21% O_2_ and 79% N on a volumetric scale. The following chemical equation represents the inlet moles number of the reactive components in the chemical mixture.

48 $$\begin{gathered} {\text{CH}}_{4} + 2{\text{O}}_{2} + 7.52{\text{N}}_{2} \to n_{{{\text{CH}}_{4} }} {\text{CH}}_{4} + n_{{{\text{H}}_{2} }} {\text{H}}_{2} + n_{{\text{H}}} {\text{H}} + n_{{{\text{O}}_{2} }} {\text{O}}_{2} \hfill \\ + n_{{\text{O}}} {\text{O}} + n_{{{\text{OH}}}} {\text{OH}} + n_{{{\text{N}}_{2} }} {\text{N}}_{2} + n_{{{\text{NO}}}} {\text{NO}} + n_{{{\text{H}}_{2} {\text{O}}}} {\text{H}}_{2} {\text{O}} + n_{{{\text{CO}}}} {\text{CO}} + n_{{{\text{CO}}_{2} }} {\text{CO}}_{2} \hfill \\ \end{gathered}$$

**Fig. 30:**
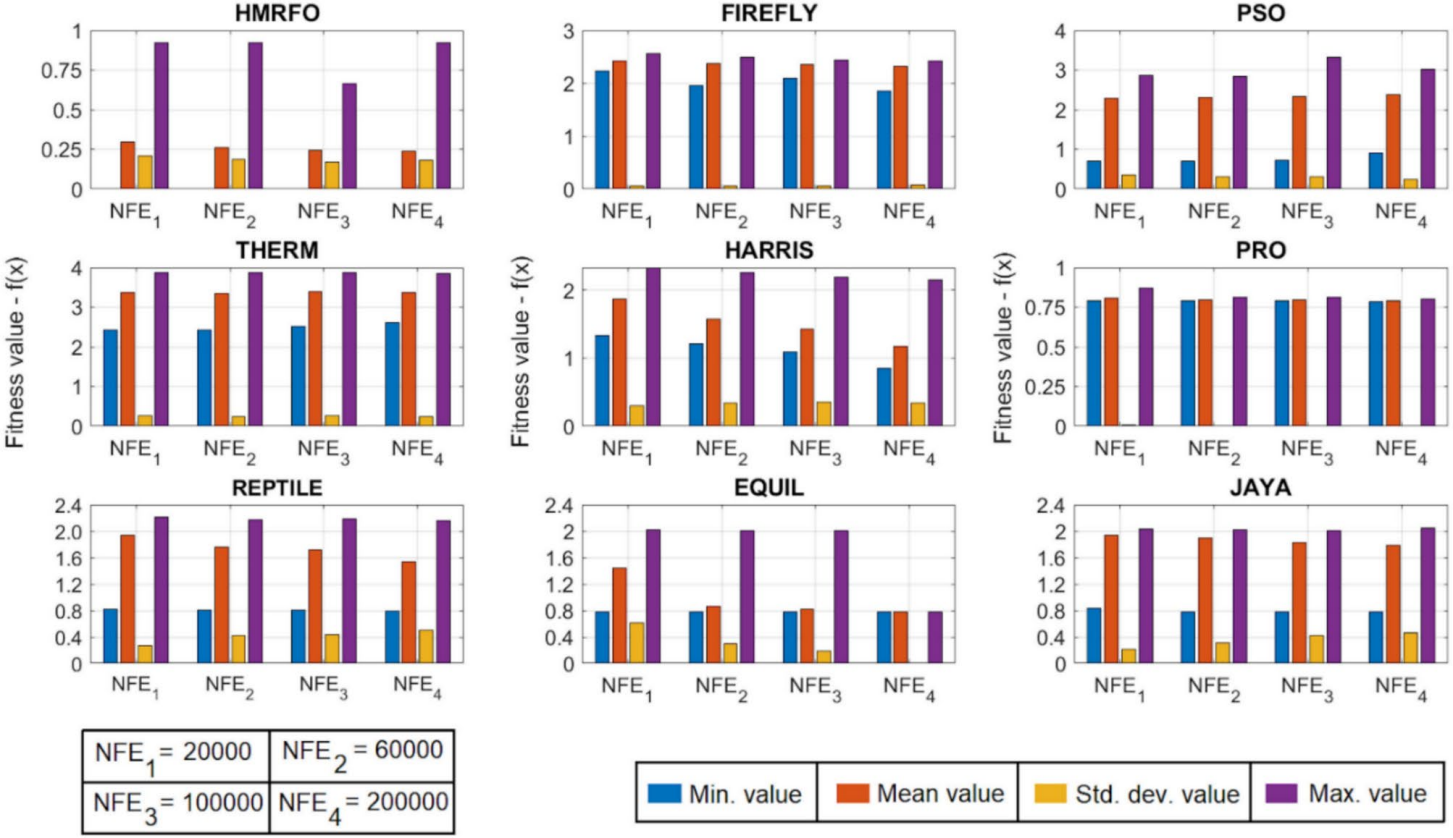
Statistical analysis of the retained objective function values for the third case study.

**Fig. 31:**
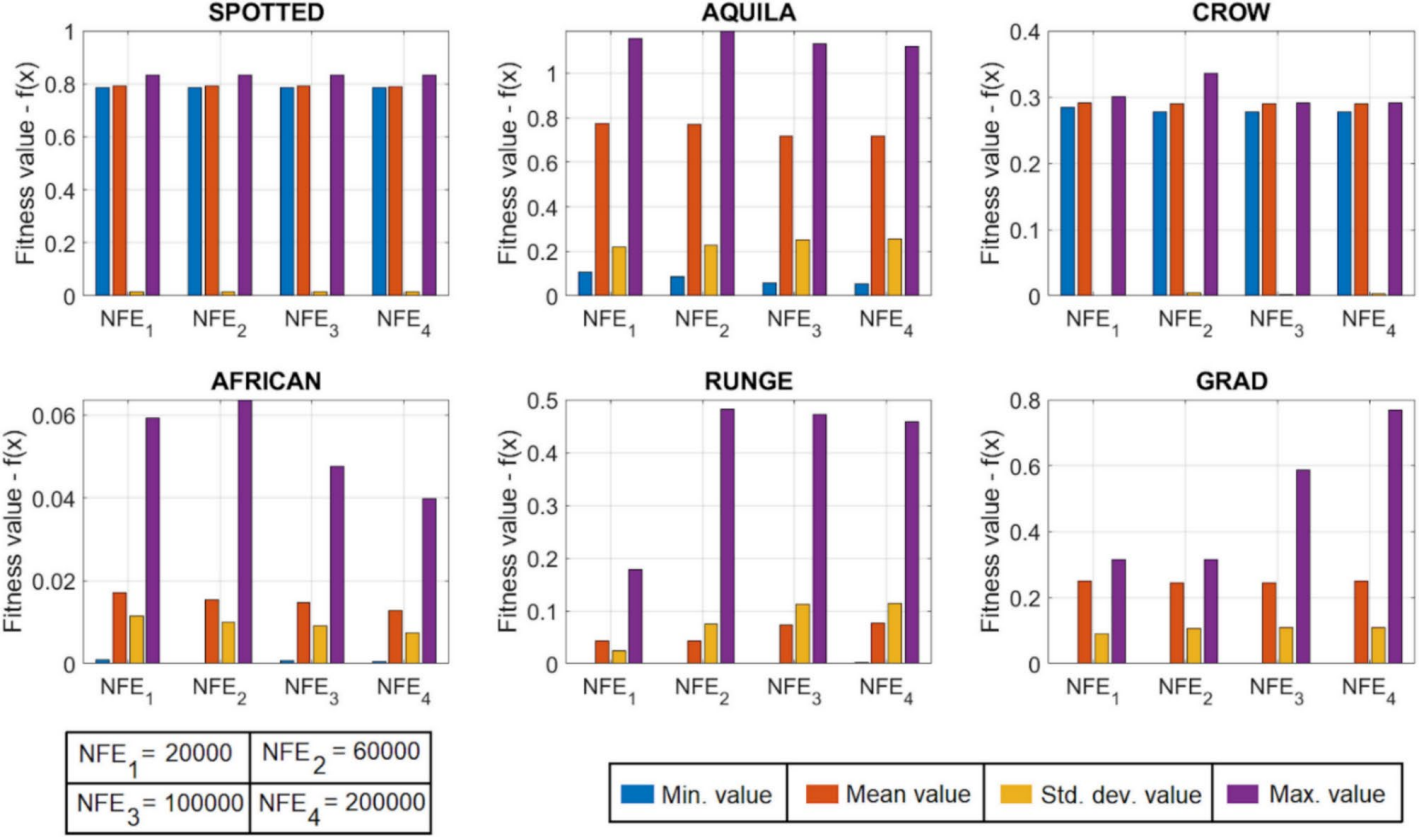
Comparative analysis between the competitive algorithms in terms of their respective statistical results.

**Fig. 32:**
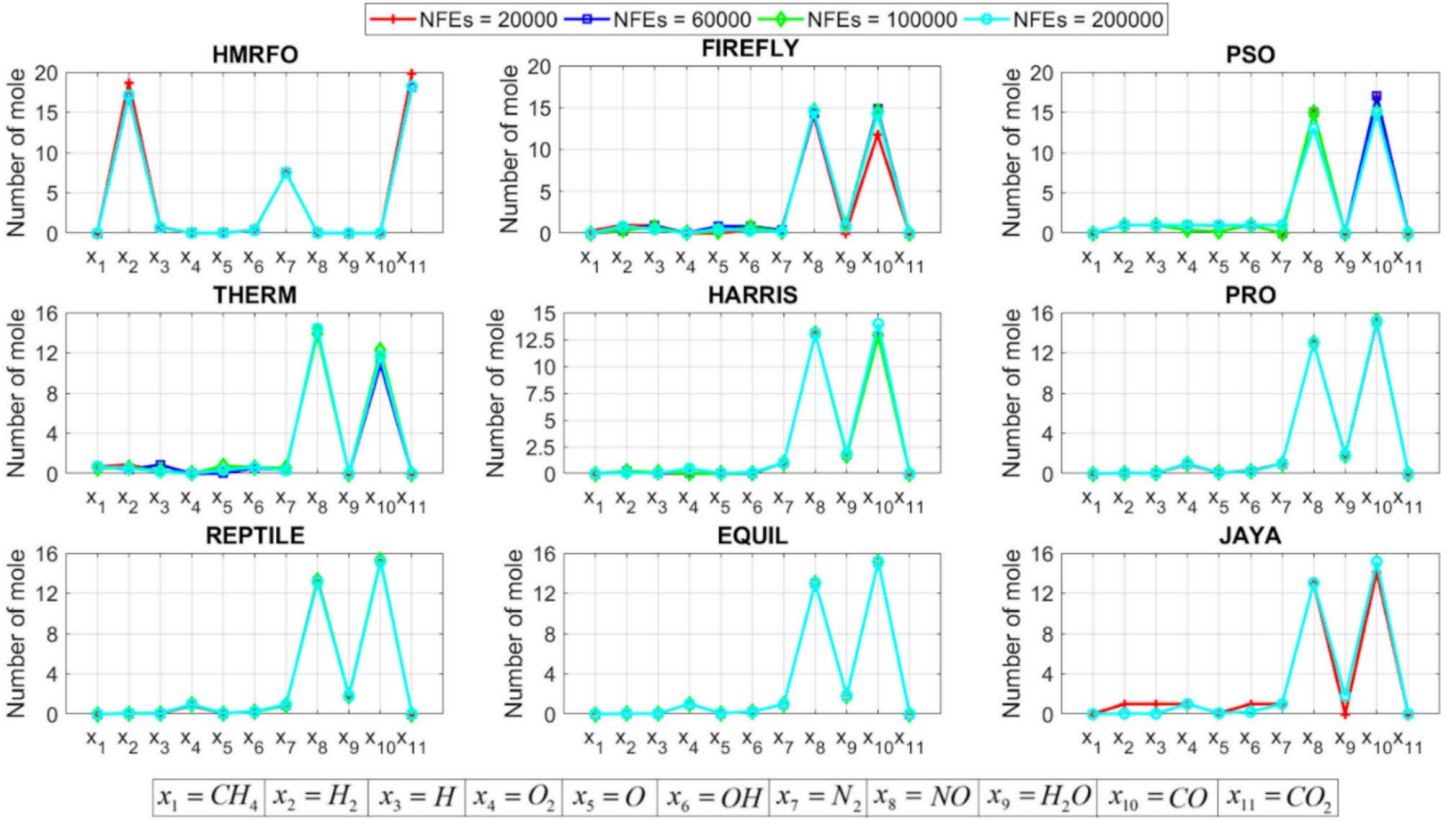
Optimal composition of reactive components in the chemical mixture for the third case study.

**Fig. 33:**
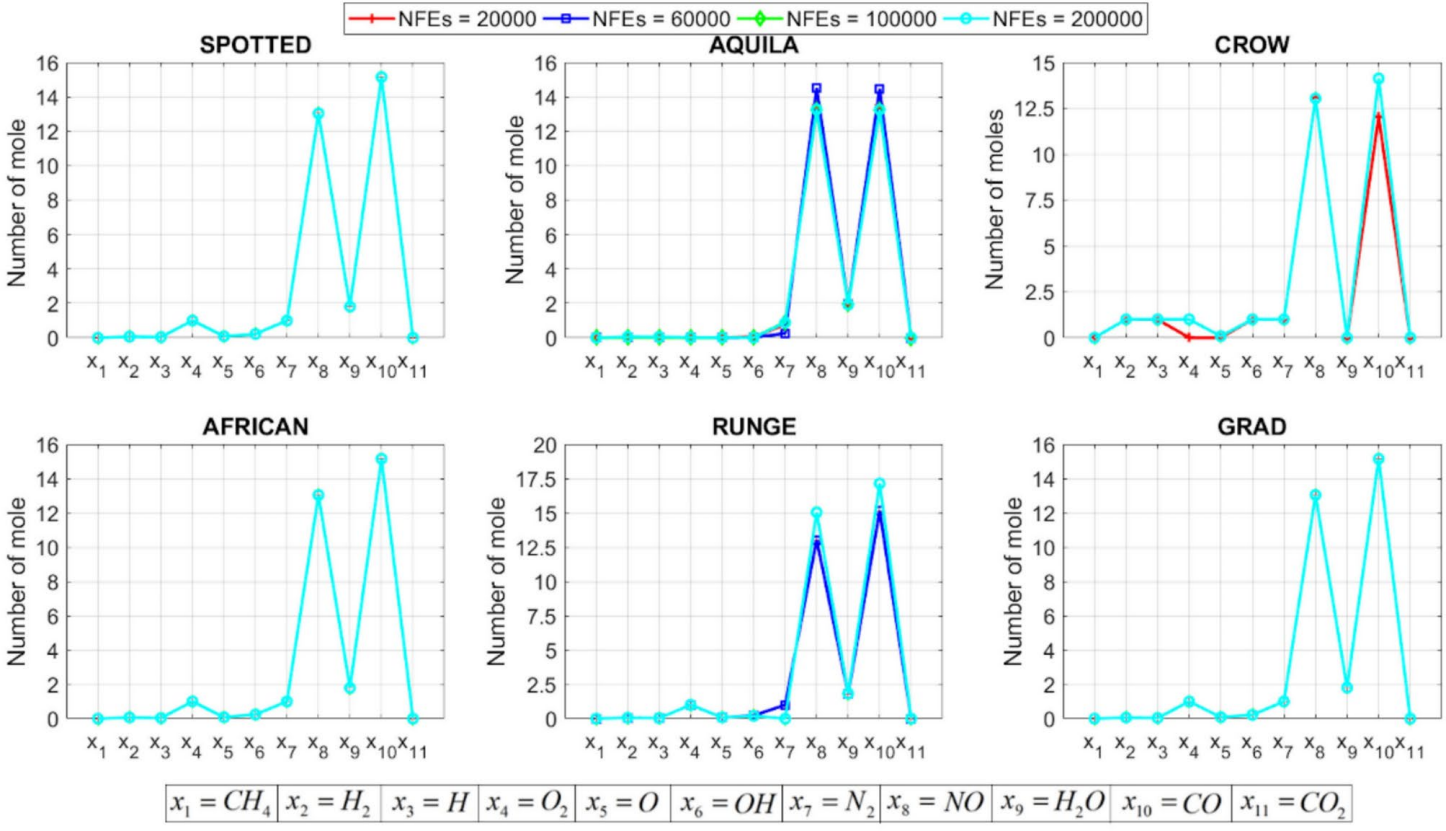
Composition of the product gases at the equilibrium condition obtained for competing algorithms.

Figures 30 and 31, respectively, compare the statistical results obtained after consecutive runs for the compared algorithm for the third case study of methane combustion. The statistics show that the AFRICAN algorithm has the best overall solution consistency among the compared algorithms, which can reach the minimum objective value on the order of 0.001 for each function evaluation case. However, observing the stagnation in the numerical value of the best fitness values with increasing function evaluation considerations is interesting. RUNGE and GRAD algorithms also provide promising predictions, yielding the best objective function values lower than 0.005 for each case. Another interesting point is that the GRAD algorithm finds the same best minimum solution of *f*(*x*) = 2.8057E−04 for each function evaluation case. AQUILA also obtains satisfactory predictions concerning best results, which is on the order of 0.05, and becomes one of the prolific methods for providing accurate predictions. Regarding solution persistence, the HMRFO algorithm is one step behind the AFRICAN, RUNGE, and GRAD algorithms. However, it obtains the best fitness value of *f*(*x*) = 9.52823E−10 when NFE is 200,000, which is far better than those obtained for the compared methods. Figures 32 and 33 compare the optimal compositions of the product gases at the equilibrium state obtained for varying function evaluations for different contestant algorithms. Similar distributions of molecular compounds have been observed in other algorithms, even with increasing numbers of iterations considered for termination criterion, except the proposed HMRFO algorithm. The equilibrium composition of the reacting components for NFEs = 200,000 is reported in Table 19. It can be understood from the best predictions results of the HMRFO given in Table 19 that all available CH_4_ in the vapor mixture is depleted, which causes the formation of 0.6901 mol of H, 0.3666 mol of OH, 17.1175 mol of H_2_, 18.2334 mol of CO_2_ is formed. While 0.0102 mol O_2_ remains in the mixture, 0.0097 mol of O atom is formed. No formation of H_2_O and CO is observed. There is no significant depletion in the mole number values of N_2_ molecule, yet minor amounts (0.0157) of NO are produced. Table 19 shows that EQUIL, SPOTTED, and JAYA algorithms reach the same best minimum fitness value of *f*(*x*) = 0.7857.

**Table 19:** Comparison of the optimum compositions of reactive components at the equilibrium condition.

	HMRFO	FIREFLY	PSO	THERM	HARRIS
$n_{{{\text{CH}}_{4} }}$	0.000000000	0.000000000	0.00000000	0.701701619	0.000000000
$n_{{{\text{H}}_{2} }}$	17.117434986	0.809224804	1.00000000	0.502677829	0.067599788
*n*_H_	0.690841701	0.442020014	1.00000000	0.193612283	0.037110896
$n_{{{\text{O}}_{2} }}$	0.010222176	0.002164740	1.00000000	0.000680384	0.463555903
*n*_O_	0.009707619	0.492869520	1.00000000	0.344683585	0.037898577
*n*_OH_	0.366566905	0.253899843	1.00000000	0.616663198	0.105264882
$n_{{{\text{N}}_{2} }}$	7.512167346	0.193683886	1.00000000	0.303712557	0.999997629
*n*_NO_	0.015665307	14.652632226	13.04000000	14.432574884	13.040004740
$n_{{{\text{H}}_{2} {\text{O}}}}$	0.000000000	0.842815060	0.00000000	0.000000000	1.861212322
*n*_CO_	0.000000000	14.246545925	15.04000000	11.680660389	13.971492330
$n_{{{\text{CO}}_{2} }}$	18.233755133	0.000000000	0.00000000	0.000000000	0.000000000
*f*_*min*_	**9.52822E−10**	1.847463992	0.90292156	2.608437863	0.846488101
*f*_*aver*_	0.236983504	2.312183299	2.37443580	3.369222155	1.172932274
*f*_*std*_	0.180541902	0.069836891	0.23898720	0.251523302	0.342603923
*f*_*max*_	0.920967713	2.419462332	3.02356066	3.850325750	2.153073509

	PRO	REPTILE	EQUIL	JAYA	SPOTTED
$n_{{{\text{CH}}_{4} }}$	0.000000000	0.000000000	0.000000000	0.000000000	0.000000000
$n_{{{\text{H}}_{2} }}$	0.071546164	0.087785099	0.061409484	0.061401767	0.061397445
*n*_H_	0.044176704	0.054068248	0.035559228	0.035556574	0.035560192
$n_{{{\text{O}}_{2} }}$	0.999798155	0.966461914	0.999999999	0.999999999	0.999999999
*n*_O_	0.100117942	0.081439683	0.082587667	0.082581845	0.082584081
*n*_OH_	0.236662731	0.244793834	0.217642143	0.217624057	0.217620107
$n_{{{\text{N}}_{2} }}$	0.999960153	0.947159976	0.999999991	0.999999999	0.999999997
*n*_NO_	13.040079693	13.145680047	13.040000016	13.040000000	13.040000004
$n_{{{\text{H}}_{2} {\text{O}}}}$	1.788034117	1.762783858	1.811989829	1.812007917	1.812012405
*n*_CO_	15.164490795	15.167621253	15.152219656	15.152213819	15.152216597
$n_{{{\text{CO}}_{2} }}$	0.000000000	0.000000000	0.000000000	0.000000000	0.000000000
*f*_*min*_	0.786831428	0.795583221	0.785727534	0.785727534	0.785727534
*f*_*aver*_	0.792197317	1.532233110	0.785727827	1.780853942	0.790405348
*f*_*std*_	0.003173362	0.499164192	4.65221E−07	0.456285283	0.014033420
*f*_*max*_	0.803722497	2.155353380	0.785730685	2.045545676	0.832505617

	AQUILA	CROW	AFRICAN	RUNGE	GRAD
$n_{{{\text{CH}}_{4} }}$	0.000000000	0.000000000	0.000000000	0.00000000	0.000000000
$n_{{{\text{H}}_{2} }}$	0.050458811	1.000000000	0.067821660	0.06099763	0.061317180
*n*_H_	0.044745871	0.999999999	0.037402892	0.03663078	0.035523949
$n_{{{\text{O}}_{2} }}$	0.019145089	1.000000000	0.999982253	0.99996128	1.000000000
*n*_O_	0.010507677	0.082708218	0.082147437	0.08732520	0.082586172
*n*_OH_	0.006041172	0.999999999	0.241794314	0.19835936	0.217193785
$n_{{{\text{N}}_{2} }}$	0.879532839	1.000000000	0.999977255	0.00000000	1.000000000
*n*_NO_	13.280934323	13.040000000	13.040045488	15.040000000	13.040000000
$n_{{{\text{H}}_{2} {\text{O}}}}$	1.924142462	0.000000000	1.792579735	1.821507289	1.812323952
*n*_CO_	13.259910609	14.122708212	15.156531484	17.147114272	15.152103909
$n_{{{\text{CO}}_{2} }}$	0.000000000	0.000000000	0.000000000	0.000000000	0.000000000
*f*_*min*_	0.053979392	0.277830352	0.000579846	0.002414118	0.000280571
*f*_*aver*_	0.716462696	0.290114983	0.012857326	0.076312085	0.251071580
*f*_*std*_	0.256559138	0.003141283	0.007484197	0.113947228	0.109198993
*f*_*max*_	1.118162517	0.290935677	0.039807913	0.458734579	0.769630649

Significant values are in [bold].

### Case study 4

The system comprises four hydro-carbon elements C, H, O, and N, constituting eleven chemical components, including H_2_, H_2_O, CO, CO_2_, O_2_, N_2_, NO, OH, H, and N. The main aim in this case is to find the accurate distribution of the mole numbers of reacting chemical products at the equilibrium condition, which covers the working temperature of 2500 K and pressure of 1 bar. A set of eight independent equilibrium equations is utilized to chemically model the adopted case study from Holub and Vonka^75^.

$${\text{H}}_{2} + {\text{CO}}_{2} \rightleftarrows {\text{CO}} + {\text{H}}_{2} {\text{O}}$$

$${\text{H}}_{2} + 0.5{\text{O}}_{2} \rightleftarrows {\text{H}}_{2} {\text{O}}$$

$${\text{H}}_{2} + {\text{O}} \rightleftarrows {\text{OH}} + {\text{H}}$$

$${\text{H}}_{2} + 2{\text{N}} \rightleftarrows {\text{N}}_{2} + 2{\text{H}}$$

$${\text{H}}_{2} + {\text{O}} \rightleftarrows {\text{H}}_{2} {\text{O}}$$

$${\text{H}}_{2} \rightleftarrows 2{\text{H}}$$

49 $${\text{H}}_{2} + {\text{NO}} \rightleftarrows 0.5{\text{N}}_{2} + {\text{H}}_{2} {\text{O}}$$

The below-given form of a chemical equation expresses the initial mole numbers of the reactants before entering the reaction.

50 $$\begin{gathered} {\text{H}}_{2} + {\text{H}}_{2} {\text{O}} + {\text{CO}} + {\text{CO}}_{2} + {\text{O}}_{2} + {\text{N}}_{2} + {\text{NO}} + {\text{OH}} + {\text{H}} + {\text{O}} + {\text{N}} \to n_{{{\text{H}}_{2} }} {\text{H}}_{2} + n_{{{\text{H}}_{2} {\text{O}}}} {\text{H}}_{2} {\text{O}} \hfill \\ + n_{{{\text{CO}}}} {\text{CO}} + n_{{{\text{CO}}_{2} }} {\text{CO}}_{2} + n_{{{\text{O}}_{2} }} {\text{O}}_{2} + n_{{{\text{N}}_{2} }} {\text{N}}_{2} + n_{{{\text{NO}}}} {\text{NO}} + n_{{{\text{OH}}}} {\text{OH}} + n_{{\text{H}}} {\text{H}} + n_{{\text{O}}} {\text{O}} + n_{{\text{N}}} {\text{N}} \hfill \\ \end{gathered}$$

**Fig. 34:**
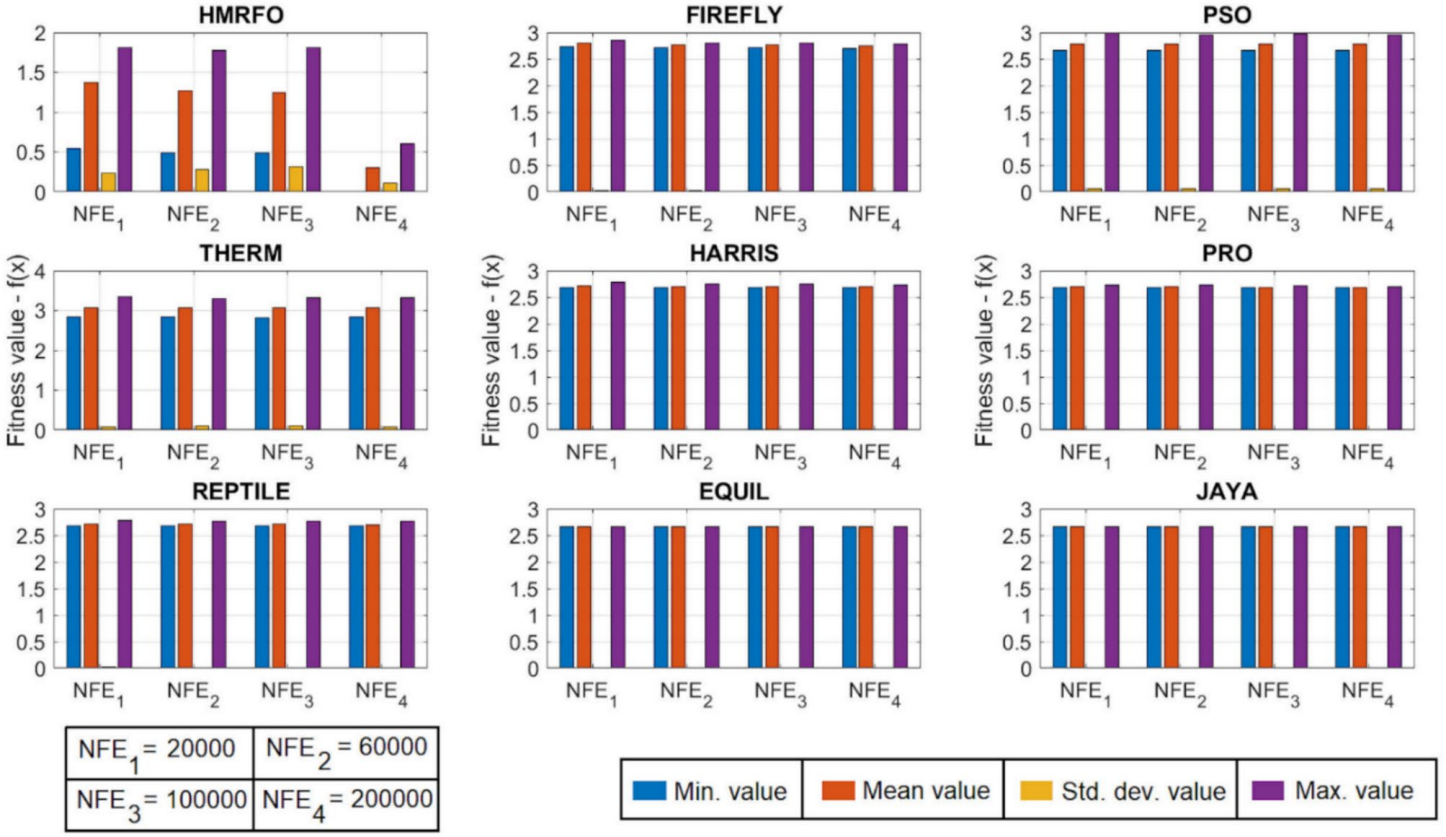
Comparison of the statistical results obtained from HMRFO, FIREFLY, PSO, THERM, HARRIS, PRO, REPTILE, EQUIL, and JAYA algorithms for the fourth case study.

**Fig. 35:**
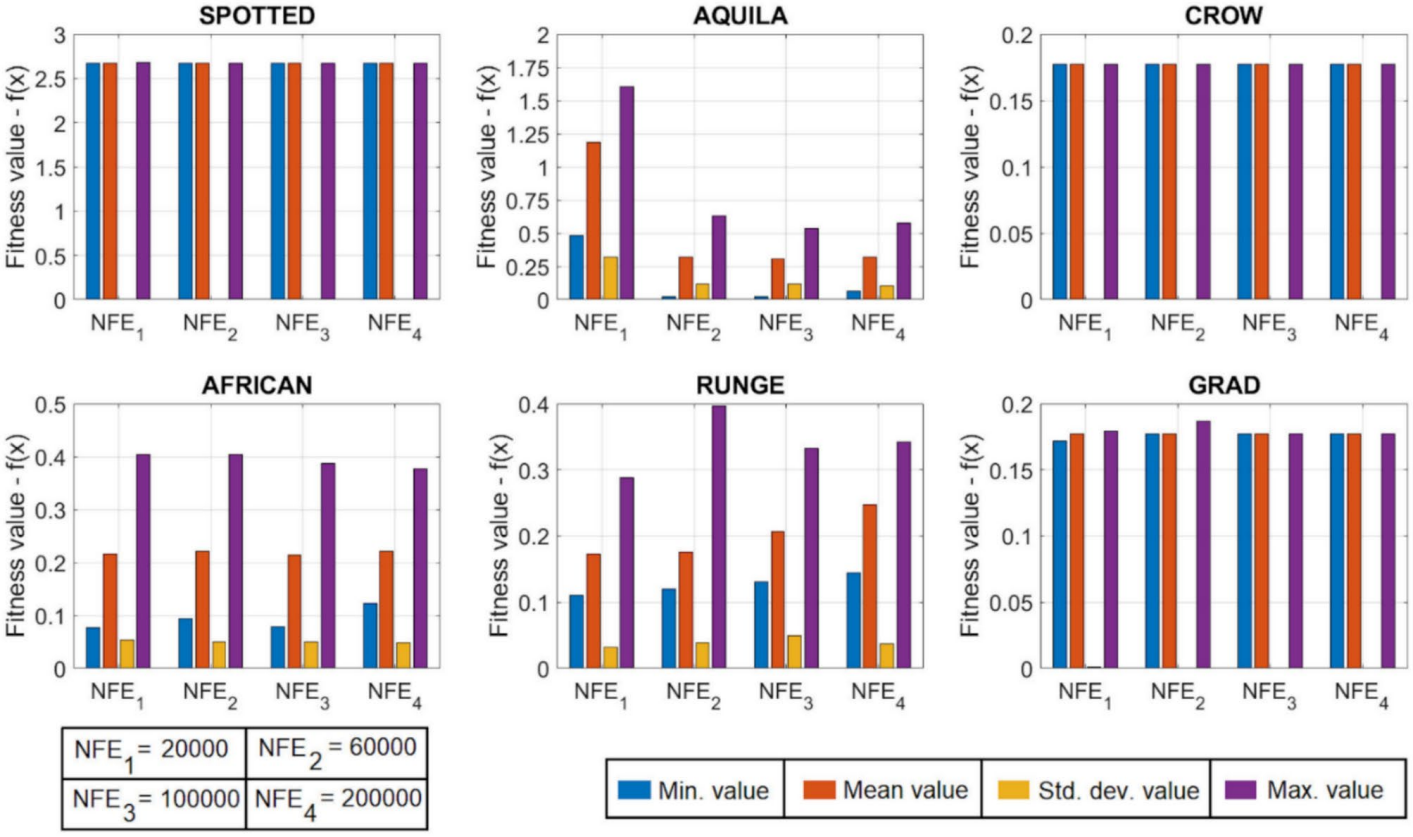
Statistical comparison of the collected results for the compared algorithms for varying numbers of function evaluations.

Figures 34 and 35 compare the statistical results obtained for the contestant algorithms for varying numbers of function evaluations for the fourth case study. There is no significant improvement in fitness qualities of the best predictions when the number of function evaluations is increased from 20,000 to 200,000 for most of the compared algorithms, except for HMRFO and AQUILA, which is an unexpected, even surprising situation in the concept of solution convergence tendencies. It was already mentioned in the second case study that algorithms experiencing problems in improving the solution qualities with an increasing number of iterations are the ones stuck on some of the troublesome local pitfalls over the search domain, posing difficulties to responsible mutation equations to make any tangible progress in altering the fitness rates. Despite their similar performance in successfully eliminating the local solutions encountered during the iteration, HMRFO reaches better qualities in objective function values with the best result of f(x) = 0.0066 when NFEs are considered 200,000.

**Fig. 36:**
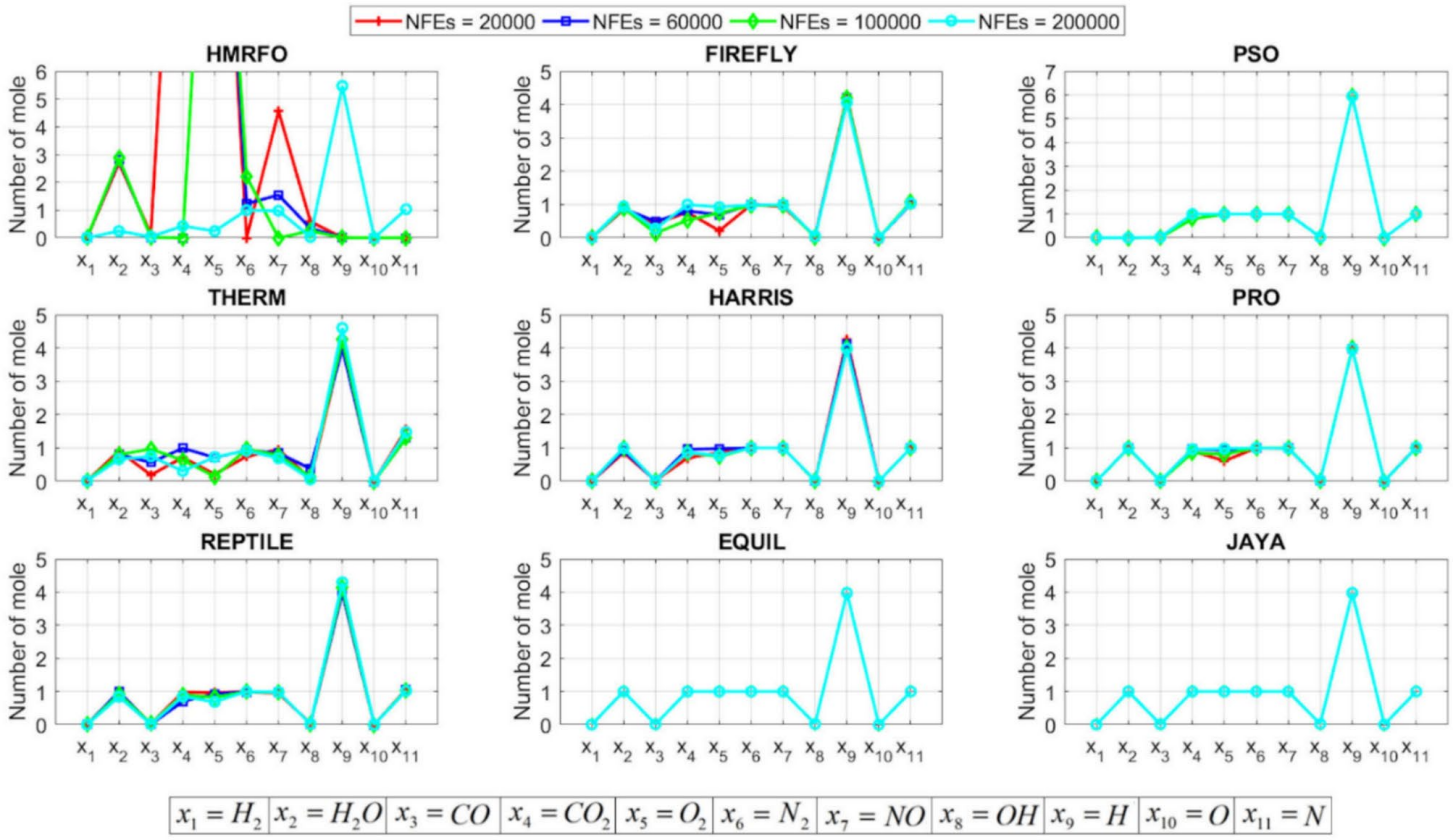
Optimal values of the reactive components in the chemical mixture for the fourth case study.

**Fig. 37:**
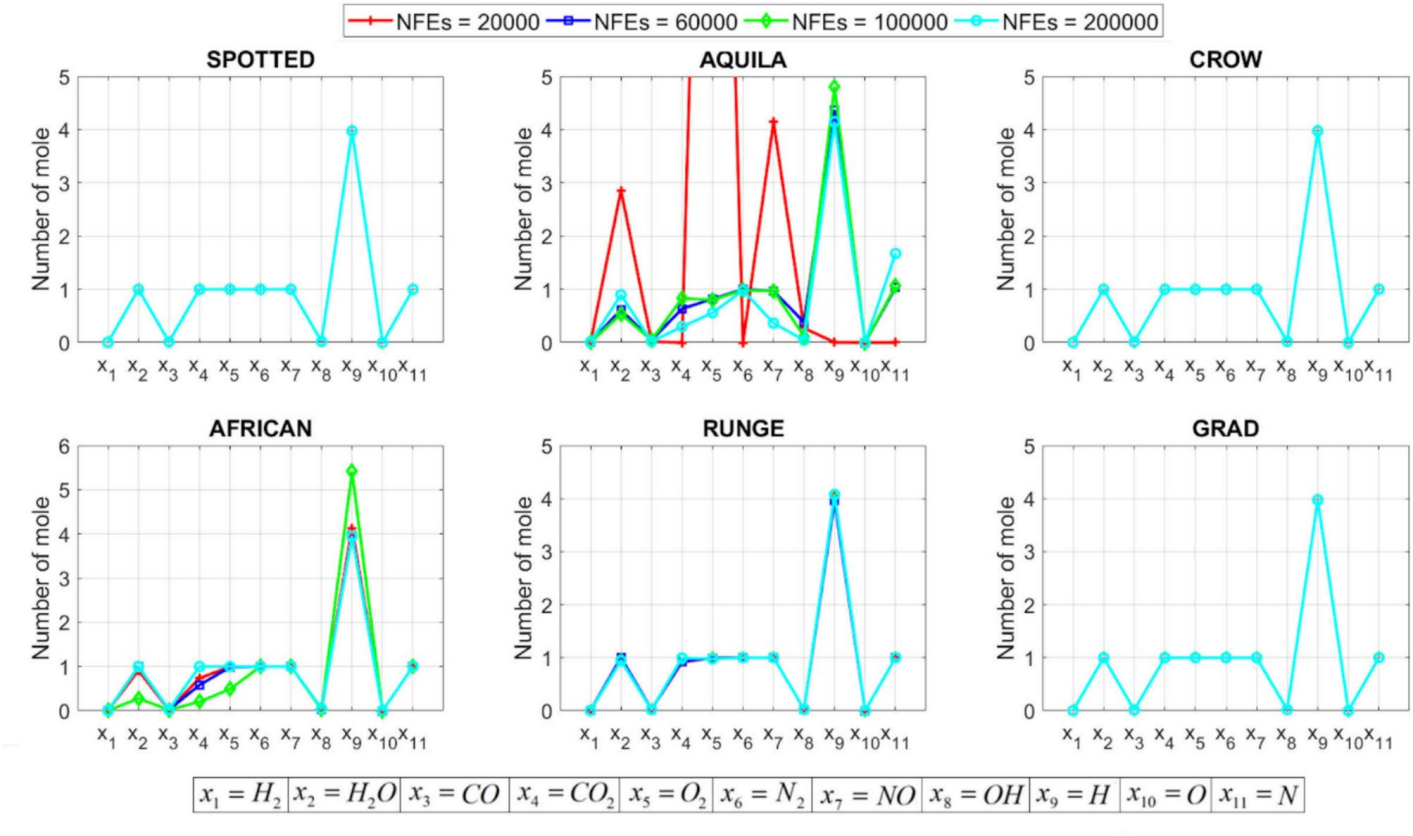
Composition of the final products in the gas phase chemical mixture.

Figures 36 and 37 schematically visualize the final equilibrium compositions of the reacting components obtained for their corresponding best fitness values by the competing metaheuristic algorithms. PSO, EQUIL, JAYA, SPOTTED, CROW, RUNGE, and GRAD algorithms find almost the same distribution reactive components in the gas mixture for varying NFEs, and no improvement in prediction accuracies is observed. Table 20 provides the equilibrium compositions of the products in the gaseous mixture according to the minimum objective function value of the employed algorithms when NFEs = 200,000.

**Table 20:** Final composition of the products acquired for the best fitness values of the competing algorithms.

	HMRFO	FIREFLY	PSO	THERM	HARRIS
$n_{{{\text{H}}_{2} }}$	0.003837337	0.004772450	0.005661051	0.012474490	0.002101710
$n_{{{\text{H}}_{2} {\text{O}}}}$	0.243722323	0.941040801	0.000000000	0.659747660	0.998592994
*n*_CO_	0.040376970	0.274191879	0.026753700	0.747860645	0.010864670
$n_{{{\text{CO}}_{2} }}$	0.424889313	0.995732719	1.000000000	0.305983804	0.865098639
$n_{{{\text{O}}_{2} }}$	0.245680220	0.923194348	1.000000000	0.725551484	0.770756318
$n_{{{\text{N}}_{2} }}$	0.996565402	0.984495518	1.000000000	0.916607245	0.999999999
*n*_NO_	0.974505836	0.994450691	1.000000000	0.690123816	0.997402617
*n*_OH_	0.033369374	0.047348665	0.035181235	0.055232453	0.023226205
*n*_H_	5.471511303	4.061024830	5.953496662	4.600323245	3.975384383
*n*_O_	0.000000000	0.000000000	0.000000000	0.000000000	0.000000000
*n*_N_	1.032363359	1.036558271	1.000000000	1.476661691	1.002597382
*f*_*min*_	**0.006564153**	2.703887695	2.662109646	2.833296168	2.674498322
*f*_*aver*_	0.302800131	2.756810520	2.789727841	3.078116508	2.696538683
*f*_*std*_	0.114442680	0.015785143	0.060470179	0.087679726	0.013037282
*f*_*max*_	0.608553649	2.791105130	2.950709995	3.317332033	2.727763544

	PRO	REPTILE	EQUIL	JAYA	SPOTTED
$n_{{{\text{H}}_{2} }}$	0.002174173	0.002250278	0.002031076	0.002031526	0.002031226
$n_{{{\text{H}}_{2} {\text{O}}}}$	0.999765684	0.840605309	0.999999734	0.999999999	0.999999999
*n*_CO_	0.012330779	0.014492331	0.012874341	0.012874698	0.012876801
$n_{{{\text{CO}}_{2} }}$	0.961855290	0.848407837	0.999999616	0.999999999	0.999999995
$n_{{{\text{O}}_{2} }}$	0.979865192	0.686241255	0.999999904	0.999999999	0.999999963
$n_{{{\text{N}}_{2} }}$	0.999525801	0.998319894	0.999999943	1.000000000	0.999999999
*n*_NO_	0.989838695	0.982147286	0.999999832	1.000000000	0.999999999
*n*_OH_	0.018672647	0.018621500	0.020835271	0.020831475	0.020825739
*n*_H_	3.977447636	4.295667323	3.975103104	3.975105470	3.975111808
*n*_O_	0.000000000	0.000000000	0.000000000	0.000000000	0.000000000
*n*_N_	1.011109701	1.021212924	1.000000279	1.000000000	1.000000000
*f*_*min*_	2.672978975	2.682314679	2.670308306	2.670308244	2.670308245
*f*_*aver*_	2.684980884	2.706022628	2.670309310	2.670308244	2.670308283
*f*_*std*_	0.006088179	0.014128386	7.19374E−15	6.67280E−15	4.40918E−08
*f*_*max*_	2.704336805	2.772610314	2.670313196	2.670308244	2.670308550

	AQUILA	CROW	AFRICAN	RUNGE	GRAD
$n_{{{\text{H}}_{2} }}$	0.006558640	0.002031526	0.003292812	0.002558456	0.002031596
$n_{{{\text{H}}_{2} {\text{O}}}}$	0.895967172	0.999999999	0.999930640	0.945528266	0.999999999
$n_{{{\text{CO}}}}$	0.019500532	0.012874699	0.026884638	0.019510171	0.012875152
$n_{{{\text{CO}}_{2} }}$	0.299324840	0.999999999	0.999810675	0.994976033	0.999999999
$n_{{{\text{O}}_{2} }}$	0.556563152	0.999999999	0.997489108	0.977145560	0.999999999
$n_{{{\text{N}}_{2} }}$	0.980710563	1.000000000	0.999998325	0.999999979	0.999999999
*n*_NO_	0.364330043	0.999999999	0.999999768	0.999999195	1.000000000
*n*_OH_	0.043359319	0.020831476	0.046612688	0.026413082	0.020831906
*n*_H_	4.151589056	3.975105470	3.946940405	4.077413471	3.975104900
*n*_O_	0.000000000	0.000000000	0.000000000	0.000000000	0.000000000
*n*_N_	1.674248829	1.000000000	1.000003579	1.000000846	1.000000000
*f*_*min*_	0.065444155	0.177497548	0.122315467	0.144640384	0.177493252
*f*_*aver*_	0.318954147	0.177497555	0.221497474	0.247315351	0.177497535
*f*_*std*_	0.107973320	3.11451E−09	0.048303041	0.037646421	3.05959E−07
*f*_*max*_	0.579790667	0.177497562	0.377798064	0.342050615	0.177498070

Significant values are in [bold].

## A comprehensive discussion of the optimization results and proposed method

This section provides a detailed discussion of the developed HMRFO algorithm concerning various optimization problems examined in this study. This section also explains why HMRFO has become the superior algorithm compared to its competitors in most test cases. The main reason that lies behind the success of the HMRFO algorithm in obtaining superior predictive solutions for a different type of optimization problems with varying dimensionalities is the developed hierarchy within the population through different search operators that can combine multiple search agents to balance between the diversification and intensification phase of the algorithm. The No Free Lunch Theorem^76^ states that no single algorithm can find the global optimum solution of all available optimization problems. A hierarchically constructed population enables manipulation by different responsible operators with varying search characteristics, which significantly increases the probability of reaching the optimum solution of the problem by enhancing diversity in the population compared to a population evolved by a single operator. Elite individuals in the hierarchy are updated by an operator most related to local exploitation. In contrast, the remaining individuals at the bottom section of the hierarchy are altered by global search agents responsible for emphasizing exploration. This synergetic interaction between the different levels of the hierarchy in the population considerably eliminates the probability of getting trapped in local solutions, prevents unexpected solution stagnation, and improves diversity in the population. Hierarchically structured population members can effectively adapt to dynamic variations in the solution environment, allowing them to switch between contradictive yet complementary exploration and exploitation search mechanisms. The inherent robustness and scalability of the hierarchical algorithms put them one step forward over standard metaheuristics, particularly for cases where search space complexity is the main issue. This behavior of structured population members is verified in the numerical experiments section, where 500D and 1000D unimodal and multimodal benchmark problems are solved. The proposed method is not intimidated by the jeopardizing influences of the curve of dimensionality and obtains promising predictions for high dimensional complex test problems.

Another interesting question is why metaheuristic algorithms are considered instead of conventional optimization methods for solving chemical equilibrium problems. Metaheuristic methods are versatile approaches that can find applications within various engineering fields thanks to their flexible and scalable features, which enable them to fit any design problem. First and foremost, metaheuristic methods offer several advantages over conventional gradient-based optimizers, which include effectively dealing with high complexities and nonlinearities inherent in chemical equilibrium problems. Traditional methods often struggle to find the optimum product distribution in the chemical mixture since they are stuck in local minimum solutions encountered in the iterative process. Derivative information of the domain is not necessary for metaheuristic problems, which may sometimes be challenging to compute for model parameters such as activity coefficients and fugacity of the gas phase component in the mixture. Compared to traditional methods, metaheuristics can handle discontinuous functions, such as uncertainties in reaction coefficients and phase transitions, without modifying the governing mathematical model. Newton-based optimization methods are prone to struggle with highly constrained chemical equilibrium problems or require complex constraint handling methods, whereas metaheuristics can efficiently work with standard constraint handling methods.

Hierarchical metaheuristic optimization methods extend standard metaheuristic algorithms by incorporating multiple levels of search mechanisms in a single population. Therefore, these algorithmic features mentioned in the first paragraph make them a favorable alternative for finding the correct number of chemical products in a reactive mixture for a chemical equilibrium problem. Faster convergence, reduced computational effort, and a reasonable balance between exploration and exploitation mechanisms are the primary superiority of hierarchical algorithms over standard metaheuristics. Furthermore, relying on the previous experiences of authors in solving chemical equilibrium problems through various metaheuristic algorithms, it is seen that the hierarchical algorithm can successfully handle restrictive model constraints such as mass, balance, charge balance, and activity coefficients compared to the standard metaheuristics. Although the time dependency of the governing chemical reaction is not considered in this research study, it is known that hierarchical methods can adapt well to the time-dependent and dynamic variations in the products. Despite their many advantages, metaheuristic algorithms may have some algorithmic drawbacks. Compared to traditional methods, they may require a relatively high number of function evaluations to reach global optimization solutions, and due to their inherent stochasticity, the optimum solution may not be achieved even after a considerable number of iterations.

When literature studies regarding solving phase equilibrium problems through metaheuristic algorithms are examined, no available research study applies a stochastic metaheuristic optimizer to solve the Gibbs Free Energy minimization method employing the proposed mathematical model. This is the primary novelty proposed in this research study that converts to the governing thermodynamical model to determine phase equilibrium point into a nonlinear set of equations, utilizing the proposed hierarchical algorithm to obtain the unknown parameters, which are, in essence, the respective mole numbers of the product components in the reactive mixture. Another novelty is associated with the hierarchical optimization method’s construction scheme. This innovative approach concurrently uses elite oppositional-based learning, dynamic oppositional-based learning, and the quantum mutation scheme in the constructed hierarchy. It is the first application of these search schemes into a single mutation to enhance solution diversity in the population. Although some of them use some variants of oppositional learning schemes^38,39^, none employs two or more variants in the hierarchical population, integrating them between a simple local or global search mechanism to enhance diversity in the population. Numerical results reveal that employing two different variants of oppositional learning search mechanisms supported by the search equations of the quantum mutation scheme significantly improves the solution quality without imposing much computational burden on the processors.

It can be reliably concluded that hierarchical metaheuristic algorithms have significant advantages over standard and conventional optimization methods for solving chemical equilibrium problems. Even though their application is restricted in this study, they can be utilized for a wide range of engineering fields, including solving single—or multi-objective optimization problems, relying on their algorithmic advantages previously discussed in this section.

## Conclusive remarks and possible future projections

This research study proposes a novel structured population-based metaheuristic algorithm for solving chemical equilibrium problems for reactive components in the gas phase. Another novelty in this theoretical work is developing an innovative mathematical model based on the governing formulations of the chemical equilibrium concept for finding the accurate number of reacting compounds in the chemical mixture at the equilibrium point.

A hierarchical population is introduced into Manta Ray Foraging Optimization (MRFO), which is comprised of intelligently devised mutation schemes, each taking a heavy responsibility to improve the solution accuracy and enhance population diversity as much as possible. Within the proposed structured hierarchical population, the Elite Opposition-based Learning mechanism updates individuals with better fitness value to intensify the promising regions previously explored. In contrast, the advanced search mechanisms of Dynamic Opposition-based Learning and Quantum-based Learning relocate average inferior solutions in the Manta ray population, entailing a considerable boost in the exploration search mechanism of the running MRFO algorithm. The proposed improved optimization method is employed on 30D, 500D, and 1000D standard optimization benchmark problems, and its search efficiency is verified based on the accuracy of the predictive results obtained for different cases. Artificially generated twenty-eight test cases composed of unimodal, multimodal, and composition benchmark problems used in 2013 competitions are utilized to evaluate further the optimization performance of the proposed HMRFO algorithm, whose respective estimations are comparatively assessed those obtained for some state-of-art optimizers. HRMFO outperforms the compared algorithm in fourteen out of twenty-eight cases, proving its superiority in solving challenging optimization problems. Finally, four different chemical equilibrium problems have been solved by the proposed HMRFO and some cutting-edge newly emerged metaheuristic optimizers utilizing the newly developed mathematical model for determining the equilibrium composition of reacting components in the gas phase mixture. HMRFO reaches the minimum objective value for each chemical equilibrium problem and surpasses the competitive algorithms used in the comparison, considering the accuracy of the solution and the best results.

It can be concluded from this comprehensive research study that taking advantage of the different mutation schemes within the fabric of the hierarchical population considerably enhances solution diversity throughout the population, thanks to the improved communication abilities of the population individuals belonging to the different levels of the constructed hierarchy. Future directions for the hierarchical-based optimizers should be associated with solving multi-dimensional complex real-world engineering design optimization cases, such as chemical equilibrium problems, which are inherently challenging. The novel developed a mathematical model for specifying the accurate amount of system components that should be applied to a more diverse set of equilibrium components with more compounds in the chemical mixture to assess its efficiency over muli-component reactive equilibrium problems. Furthermore, relying on its success in real-world complex design cases, the proposed method can be a favorable candidate for solving oil spill remediation problems^77^ and measuring the degree of conflicts between evidence^78^, which are hard-to-solve cases for conventional optimizers yet well-suited to metaheuristic algorithms for their successful solution.

## Data Availability

The datasets used during the current study are available from the corresponding author upon reasonable request.
